# Circulating inflammatory cytokines and risk of idiopathic pulmonary fibrosis: a Mendelian randomization study

**DOI:** 10.1186/s12890-023-02658-3

**Published:** 2023-10-03

**Authors:** Qinyao Jia, Yanmei Lei, Shaoping Chen, Shengming Liu, Tao Wang, Yao Cheng

**Affiliations:** 1 School of Pharmacy, North Sichuan Medical College, Nanchong, People’s Republic of China; 2 Department of Tuberculosis, Chengdu Public Health Clinical Medical Center, Chengdu, People’s Republic of China; 3 Department of Nuclear Medicine, Affiliated Hospital of Chengdu University of Traditional Chinese Medicine, Chengdu, People’s Republic of China; 4 Department of Pulmonary and Critical Care Medicine, Affiliated Hospital of North Sichuan Medical College, Nanchong, People’s Republic of China; 5 Department of Pulmonary and Critical Care Medicine, The First Affiliated Hospital of Jinan University, Guangzhou, People’s Republic of China; 6 Department of Pulmonary and Critical Care Medicine, University of Chinese Academy of Sciences Shenzhen Hospital, The first Affiliated Hospital of Jinan University, Shenzhen, Guangzhou, People’s Republic of China

**Keywords:** Circulating inflammatory cytokines, Idiopathic pulmonary fibrosis, Mendelian randomization, Genetics

## Abstract

**Background:**

The previous epidemiological and experimental evidence has implied the linkage between chronic inflammation to idiopathic pulmonary fibrosis (IPF). However, it was still unclear whether there were casual associations between circulating inflammatory cytokines and IPF development. The objective of present study was to examine whether altered genetically predicted concentration of circulating cytokines were associated with IPF development using a two-sample Mendelian randomization (MR) analysis.

**Materials and methods:**

The causal effects of 23 circulating inflammatory cytokines were evaluated on IPF using MR analysis. The primary approach of MR analysis was the inverse variance-weighted (IVW) method. The sensitivity analyses were conducted by simple median, weighted median, penalized weighted median and MR-Egger regression methods.

**Results:**

The present MR study found suggestive evidence that a higher circulating IL-14 level was associated with an increased risk of IPF (random effects IVW method: odds ratio: 1.001, 95% confidence interval: 1.000-1.001, P = 0.026). The sensitivity analysis yielded directionally similar results for IL-14. There was no significant association found between other circulating inflammatory cytokines and IPF.

**Conclusion:**

The high level of IL14 predicted by genes had a casual relationship with the increased risk of IPF. This finding provided epidemiological evidence for drug therapy targeting inflammatory factors in the prevention and treatment of IPF. It’s warranted further exploration to validate the clinical significance of IL14 associated with developmental risk of IPF.

**Supplementary Information:**

The online version contains supplementary material available at 10.1186/s12890-023-02658-3.

## Introduction

Idiopathic pulmonary fibrosis (IPF) is a prototype of chronic, progressive, and fibrotic lung disease with replacement of altered extracellular matrix and destroy of alveolar architecture, leading to decreased lung compliance, disrupted gas exchange, and ultimately respiratory failure and death [1]. Although IPF is the most common type of idiopathic interstitial pneumonia, the exact etiology is still unknown with diverse hypotheses. In recent years, dysfunction of alveolar epithelial type 2 cells has recently been recognized associated with the occurrence and progression of IPF, which is indispensable in the regeneration and lung surfactant secretion of alveolar epithelial cells [2–4]. A review conducted by Heukels et al. has indicated that both innate and adaptive immune systems play a vital role in the initiation and perpetuation of IPF pathobiology [5]. Multiple innate immune cells are involved in IPF, such as neutrophils and fibrocytes, leading to tissue remodeling and ongoing fibrosis. T-cells are present as representative of adaptive immune system involved in IPF that Tregs have a protective role to decrease fibrocyte accumulation and dampen inflammatory responses [6–8]. The presence of inflammation induced by epithelial-mesenchymal transition and genetic variations has been demonstrated associated with increased risk of developing IPF [9] [10, 11]. Subsequent studies on experimental animal models have confirmed that the progression of pulmonary fibrosis can be effectively blocked through inhibition of inflammatory response [12–15]. However, there has been no study to report whether the circulating inflammatory cytokines have causal relationships with the developmental risk of IPF.

Mendelian randomization (MR) is an observational study design that seeks to understand causal relationships between an exposure (e.g., a risk factor or biomarker) and an outcome (e.g., a disease or trait) by using genetic variants as instrumental variables, which has been widely applied to mitigate certain significant drawbacks encountered by conventional observational studies [16]. Specifically, MR approach utilizes germline genetic variations as instrumental variables (IV) to examine the causal relationship between exposure phenotype and outcome phenotype. MR analysis shows its methodological advantages by lending greater credence to causal claims compared to traditional observational approaches. In rare diseases like IPF, it may be difficult or unethical to conduct RCTs due to the limited number of affected individuals. MR studies can overcome this limitation by using genetic data available from large-scale population-based studies. MR studies also show strengths in examining the long-term effects of exposures on rare pathologies by analyzing genetic variants associated with exposure that are present from birth. This is particularly useful when studying diseases with a long latency period [17]. MR approach has potential to facilitate the utilization of publicly available data sourced from sweeping genome-wide association studies (GWASs), along with concurrently circumventing the typical pitfalls inherent to observational investigations [18]. GWAS encompasses datasets including both exposure and outcome metrics using regression estimation of the genetic variants. A previous study has employed MR analyses to evaluate the putative causal links between a diverse array of immunological proteins/traits and schizophrenia, major depression, and bipolar disorder [19]. Specifically, the study has reported evidence in support of potential causal associations of several immunological proteins/traits with rare pathologies, like schizophrenia, including pro-inflammatory cytokines and anti-inflammatory cytokines, in addition to acute-phase proteins and chemokines. This case of rare disease has raised the potential to transmit MR analysis integrated with GWAS into IPF by providing methodological reference. The present study aimed to apply a two-sample MR analysis to evaluate whether circulating inflammatory cytokines have causal associations with developmental risk of IPF, providing novel insights about how inflammation contributes to the initiation and progression of IPF.

## Materials and methods

### Study design and data sources

**Fig. 1:**
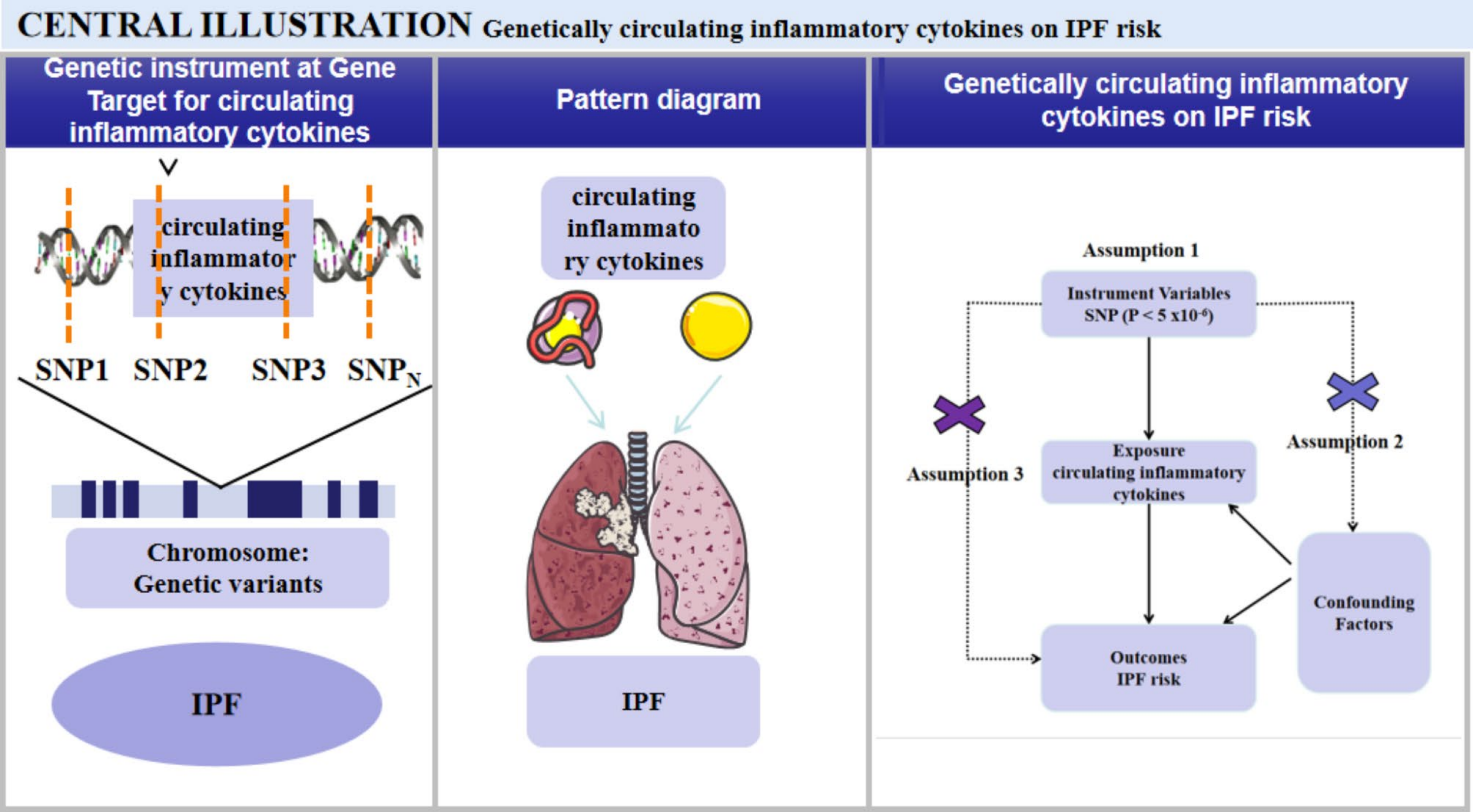
Illustrates the diagram of the study design. (1) Selection of genetic variants that were proxies of the effect of circulating inflammatory cytokines. (2) Selection of IPF as the outcomes. 4) Two-step MR analysis estimating the causal effects of circulating inflammatory cytokines on the IPF.

We employed a two-sample MR analysis model in order to assess the causal impact of circulating inflammatory cytokines on IPF, as depicted in Fig. 1. The utilization of MR analysis enables mitigation of unmeasured confounding variables and facilitates more compelling causal inferences. Hence, this method proves to be appropriate for determining whether an exposure factor can be attributed to causing the onset of a particular disease [20]. A total of seven MR analysis models were employed to investigate the causal influence of 23 circulating inflammatory cytokines (including CRP, MIP1a, MIP1b, IL-1ra, IL-2ra, IL-2, IL-6, IL-8, IL-10, IL-13, IL-14, IL-16, IL-17, IL-18, MCP1, MIF, Eotaxin, GROa, MIP1a, MIP1b, RANTES, TANLN, and CXCL9) on the development of IPF. In this study, we leveraged previously reported genetic variants that have been associated with the levels of circulating inflammatory cytokines. These genetic variants were sourced from the most comprehensive meta-analysis of GWAS focusing on cytokine-related traits across three separate cohorts - the Cardiovascular Risk in Young Finns Study (YFS), FINRISK 1997, and FINRISK 2002, with a cumulative participant count of up to 8,293 individuals of Finnish descent [21]. The primary outcome measure of this investigation focused on estimating the lifetime risk of developing IPF. To obtain the relevant summary statistics data for IPF, we relied on the results from the most recent and extensive GWAS available (reference number: ebi-a-GCST90018120). This particular GWAS meticulously evaluated the associations involving an extensive set of 16,137,102 genotyped single nucleotide polymorphisms (SNPs) in relation to IPF. The study encompassed a substantial sample of European descent, comprising 1,369 cases and 435,866 controls, totaling 45,1025 individuals. Our research adhered to all pertinent guidelines and protocols to ensure methodological rigor and ethical compliance. Further information and data for this GWAS can be accessed at the following location: https://gwas.mrcieu.ac.uk/datasets/ebi-a-GCST90018120/.

### SNPs selection

We employed a rigorous approach to identify SNPs that exhibit a significant association with circulating inflammatory cytokines. These identified SNPs were subsequently utilized as instrumental variables (IVs) in our analysis. The selection criteria for these IVs included a stringent significance threshold (P < 5 × 10 − 6), as well as specific considerations pertaining to linkage disequilibrium (LD), whereby the LD measure (r2) was required to be below 0.001 and the LD distance to exceed 10,000 kb. Subsequently, we endeavored to locate information regarding the aforementioned IV SNPs within the IPF dataset sourced from large-scale GWASs. Specifically, this IPF dataset was acquired from the prominent GWAS conducted by Duckworth, as identified by the unique identifier (id: ebi-a-GCST90018120). Readers looking to access the detailed data for IPF can refer to the following resource: https://gwas.mrcieu.ac.uk/datasets/ebi-a-GCST90018120/ [22]. The studies that provided data for the GWAS meta-analyses had undergone appropriate ethical review by relevant institutional review boards, thus ensuring compliance with ethical standards. For the purposes of our study, we solely extracted summarized data from these existing studies, thereby obviating the need for any supplemental ethical approvals. Comprehensive information pertaining to all SNPs employed in our investigation can be found in the Supplemental materials [23].

### Statistical analysis

In this study, a comprehensive array of seven MR analysis methods were employed. These methods encompassed the inverse-variance weighted (IVW) technique, both in the fixed-effect and random-effect frameworks, alongside the simple median, weighted median, penalized weighted median, MR Egger, and MR Egger (bootstrap) approaches. The IVW method, serving as the primary analytical tool, was utilized due to its ability to yield reliable estimates of causal effects, even in the presence of heterogeneity. To further ensure robustness and explore potential sources of bias, two additional methods, namely the weighted median estimator and MR-Egger, were implemented for sensitivity analyses. The weighted median estimator was particularly proficient in furnishing dependable causal assessments when a majority of the instrumental variables adhered to MR assumptions. On the other hand, the MR-Egger estimate remained unbiased under the proviso that the genetic instrument exhibited no dependency on pleiotropic effects. Moreover, in order to comprehensively evaluate the presence of pleiotropic effects and heterogeneity among individual SNPs, we employed the IVW method in conjunction with MR Egger intercept and Cochran’s Q statistics. The absence of pleiotropic effects was determined if the intercept did not significantly deviate from 0 (p > 0.05). Heterogeneity, on the other hand, was assessed based on the value of Cochrane’s Q statistic. When the p-value of this statistic was less than 0.05, the IVW method with a multiplicative random-effects model was selected as the primary outcome. Conversely, if the p-value was greater than or equal to 0.05, the IVW method with a fixed-effects model was considered the primary outcome. To further account for potential pleiotropy and derive a causal effect assessment while addressing the issue of directional horizontal pleiotropy, MR-Egger regression was also performed in this study. Furthermore, a leave-one-out analysis was conducted to assess the robustness of the MR analysis results by examining the influence of individual outlier SNPs. In line with a previous study, a causal relationship was deemed significant if three conditions were met: (1) the p-value of the IVW method was less than 0.05, (2) the estimates from the IVW, MR-Egger, and weighted median methods were consistent in direction, and (3) the p-value of the MR-Egger intercept test was greater than 0.05. All statistical analyses were undertaken using the “TwoSampleMR” package in R version 3.4.1 (R Foundation for Statistical Computing, Vienna, Austria), and a two-tailed p-value of less than 0.05 was considered statistically significant.

## Results

### The characteristics of instrumental variables

We included 555 SNPs significantly related to the circulating inflammatory cytokines variation as IVs for circulating inflammatory cytokines-IPF causal estimations. We used a 2-sample MR model to evaluate the causal effect of circulating inflammatory cytokines on IPF (Fig. 1). The general characteristics of IVs of circulating inflammatory cytokines and IPF are shown in the Supplementary Table 1.

### Circulating CRP on IPF

**Fig. 2:**
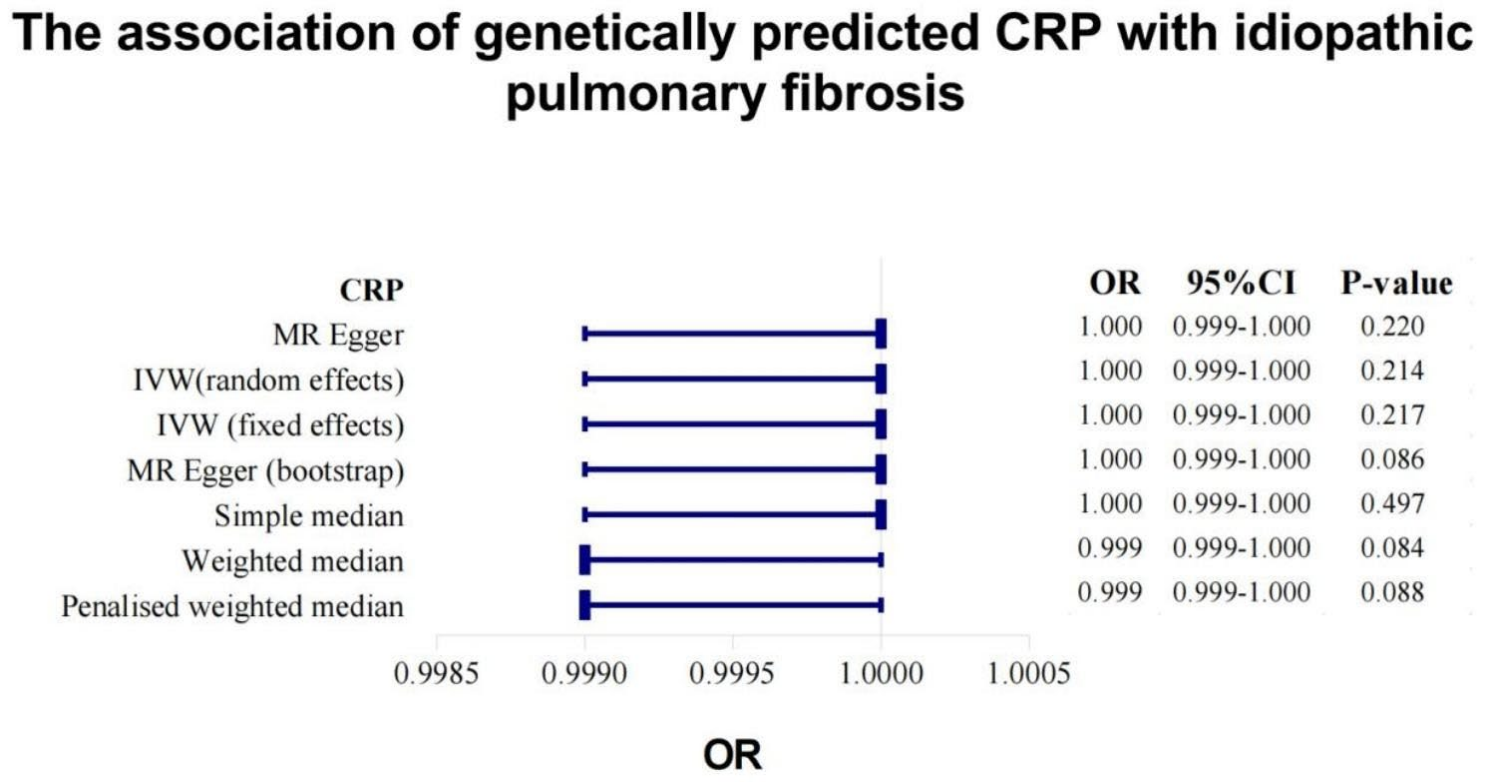
The forest plot for the causal effect of CRP on IPF by each common MR analytical methods

Genetically predicted higher plasma CRP levels showed a suggestive inverse association with IPF using IVW method (fixed-effect model: OR, 1.000; 95% CI, 0.999-1.000, P = 0.217; random-effect model: OR, 1.000; 95% CI, 0.999-1.000, P = 0.214) (Fig. 2). A similar finding was observed using the weighted median method (OR, 1.000; 95% CI, 0.999-1.000, P = 0.084) and MR-Egger regression method (OR, 1.000; 95% CI, 0.999-1.000, P = 0.220) (Fig. 2). The intercept didn’t indicate existence of pleiotropy (P = 0.586) (Supplementary Table 2). The results of Cochran’s Q test revealed no heterogeneity across the SNPs (P = 0.554) (Supplementary Table 2).

### Circulating inflammatory cytokines (IL family) on IPF

**Fig. 3:**
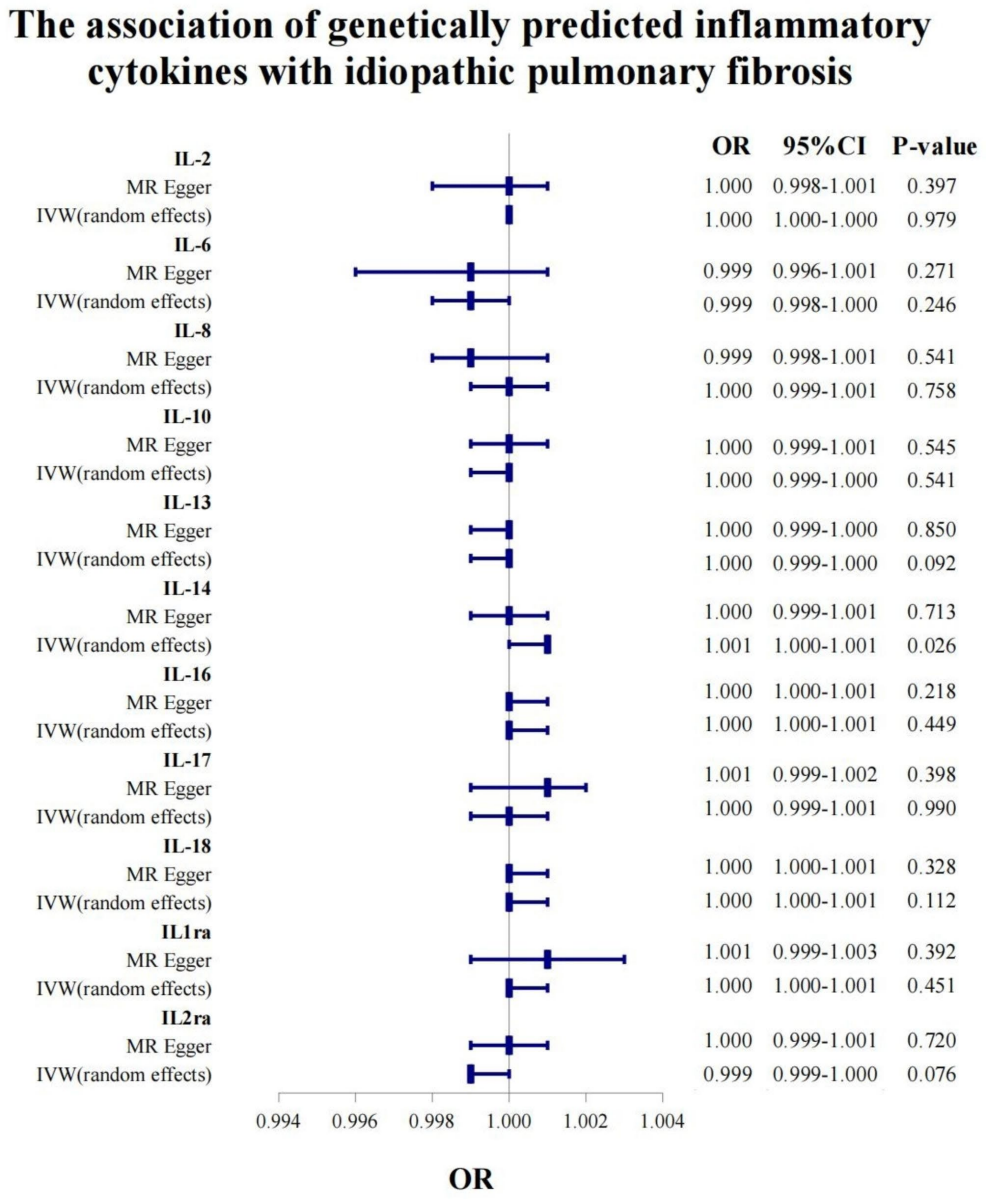
The forest plot for the causal effect of circulating interleukin family cytokines on IPF by IVW and MR Egger MR analytical methods

The IVW MR methods were performed to analyze the final results. The other MR analysis method (simple median, weighted median, penalized weighted median, MR Egger) were performed as the complement to IVW, confirming the robustness of the IVW analysis results. The MR analysis by MR Egger and IVW showed that IL2 was not significantly associated with risk of IPF (MR Egger: P = 0.397; random-effect model: P = 0.979) (Table 1; Fig. 3). However, IL2ra by IVW fixed-effect model was significantly associated with a decreased risk of IPF (P = 0.022) (Supplementary Table 2). Although there was a statistically significant difference in the causal effect of IL2ra on IPF, the OR value in MR Egger analysis was not support the above results, so we did not consider this result to have significant clinical significance based on the results of our previous methodology. We also performed MR analysis for other interleukin factors and found that only IL-14 had a significant causal effect on the increased risk of IPF (random-effect model IVW: OR, 1.001; 95% CI, 1.000-1.001, P = 0.026) (Table 1). Sensitivity analysis yielded directionally similar results for IL-14 (MR Egger: OR: 1.000, 95% CI: 0.999–1.001, P = 0.713; Weighted median: OR: 1.000, 95% CI: 0.999–1.001, P = 0.767). Except for IL-14, other inflammatory cytokines in the IL family were not significantly associated with developmental risk of IPF (P > 0.05) (Table 1). Moreover, there was no significant heterogeneity found in circulating inflammatory cytokines (Table 1).

**Table 1:** Effect estimates for association of genetically predicted circulating inflammatory cytokines (IL family) with idiopathic pulmonary fibrosis using MR Egger and random effects IVW methods

IL family	Methods	OR	95%CI	P-value	Cochran’s Q	P for Cochran’s Q
IL2	MR Egger	1.000	0.998–1.001	0.397	2.970	0.965
	IVW (random effects)	1.000	1.000–1.000	0.979	4.015	0.947
IL6	MR Egger	0.999	0.996–1.001	0.271	8.684	0.124
	IVW (random effects)	0.999	0.998-1.000	0.246	9.642	0.141
IL-8	MR Egger	0.999	0.998–1.001	0.541	3.609	0.165
	IVW (random effects)	1.000	0.999–1.001	0.758	4.495	0.213
IL-10	MR Egger	1.000	0.999–1.001	0.545	30.654	0.103
	IVW (random effects)	1.000	0.999-1.000	0.541	30.822	0.127
IL-13	MR Egger	1.000	0.999-1.000	0.850	9.635	0.210
	IVW (random effects)	1.000	0.999-1.000	0.092	10.892	0.208
IL-14	MR Egger	1.000	0.999–1.001	0.713	5.694	0.770
	IVW (random effects)	1.001	1.000-1.001	0.026	6.337	0.786
IL-16	MR Egger	1.000	1.000-1.001	0.218	9.601	0.384
	IVW (random effects)	1.000	1.000-1.001	0.449	10.853	0.369
IL-17	MR Egger	1.001	0.999–1.002	0.398	5.543	0.852
	IVW (random effects)	1.000	0.999–1.001	0.990	6.580	0.832
IL-18	MR Egger	1.000	1.000-1.001	0.328	8.004	0.889
	IVW (random effects)	1.000	1.000-1.001	0.112	8.222	0.915
IL1ra	MR Egger	1.001	0.999–1.003	0.392	2.486	0.870
	IVW (random effects)	1.000	1.000-1.001	0.451	3.119	0.874
IL2ra	MR Egger	1.000	0.999–1.001	0.720	10.293	0.113
	IVW (random effects)	0.999	0.999-1.000	0.076	11.640	0.113

Abbreviations: IL, interleukin; MR, Mendelian randomization; IVW, Inverse-variance weighted; OR, Odd ratio; CI, Confidence interval

### Circulating inflammatory cytokines (chemokine family) on IPF

**Fig. 4:**
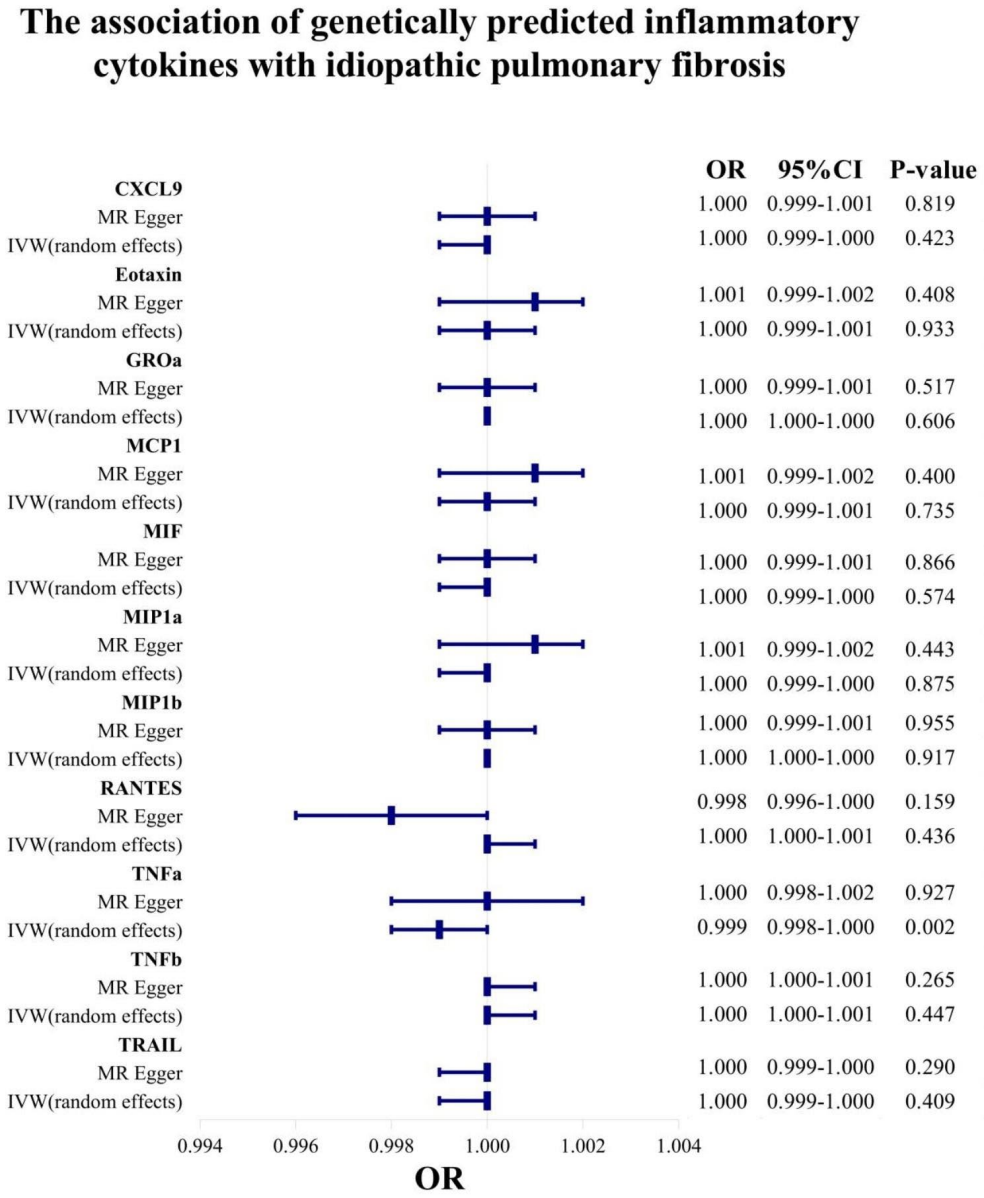
The forest plot for the causal effect of circulating chemokine on IPF by IVW and MR Egger MR analytical methods

The MR analysis by IVW models and other analytic methods (MR Egger, simple median, weighted median, penalised weighted median) showed that MCP1 was not significantly associated with development risk of IPF (P > 0.05) (Table 2; Fig. 4). TNFa was found significantly associated with a decreased risk of IPF by IVW models (OR, 0.999; 95% CI, 0.998-1.000, P = 0.002), whereas the opposite result was observed in the MR Egger method (OR, 1.000; 95% CI, 0.998–1.002, P = 0.927) (Fig. 4). Therefore, the results of present study couldn’t prove a causal relationship between TNFa and IPF. Except for MCP1 and TNFa, other circulating inflammatory cytokines in chemokine family were found not significantly associated with developmental risk of IPF (P > 0.05) (Table 2). Otherwise, the results suggested the existence of heterogeneity for RANTES (P = 0.040), Eotaxin (P = 0.007) and MCP1 (P = 0.012).

**Table 2:** Effect estimates for association of genetically predicted circulating inflammatory cytokines (chemokine family) with idiopathic pulmonary fibrosis using MR Egger and random effects IVW methods

Chemokine family	Methods	OR	95%CI	P-value	Cochran’s Q	P for Cochran’s Q
CXCL9	MR Egger	1.000	0.999–1.001	0.819	9.636	0.648
	IVW (random effects)	1.000	0.999-1.000	0.423	9.650	0.722
Eotaxin	MR Egger	1.001	0.999–1.002	0.408	31.465	0.008
	IVW (random effects)	1.000	0.999–1.001	0.933	33.399	0.007
GROa	MR Egger	1.000	0.999–1.001	0.517	5.736	0.571
	IVW (random effects)	1.000	1.000–1.000	0.606	6.630	0.644
MCP1	MR Egger	1.001	0.999–1.002	0.400	25.723	0.012
	IVW (random effects)	1.000	0.999–1.001	0.735	27.129	0.012
MIF	MR Egger	1.000	0.999–1.001	0.866	3.989	0.551
	IVW (random effects)	1.000	0.999-1.000	0.574	4.326	0.633
MIP1a	MR Egger	1.001	0.999–1.002	0.443	3.956	0.785
	IVW (random effects)	1.000	0.999-1.000	0.875	4.794	0.779
MIP1b	MR Egger	1.000	0.999–1.001	0.955	24.863	0.129
	IVW (random effects)	1.000	1.000–1.000	0.917	24.904	0.164
RANTES	MR Egger	0.998	0.996-1.000	0.159	11.708	0.165
	IVW (random effects)	1.000	1.000-1.001	0.436	17.618	0.040
TNFa	MR Egger	1.000	0.998–1.002	0.927	0.000	0.977
	IVW (random effects)	0.999	0.998-1.000	0.002	0.931	0.628
TNFb	MR Egger	1.000	1.000-1.001	0.265	1.888	0.389
	IVW (random effects)	1.000	1.000-1.001	0.447	3.653	0.301
TRAIL	MR Egger	1.000	0.999-1.000	0.290	21.717	0.196
	IVW (random effects)	1.000	0.999-1.000	0.409	22.401	0.215

Abbreviations: MR, Mendelian randomization; IVW, Inverse-variance weighted; OR, Odd ratio; CI, Confidence interval

### Sensitivity analyses

The present study carried out a series of sensitivity analyses to evaluate the potential horizontal pleiotropy. The results of MR-Egger Intercept-test indicated that there was not significantly impact caused by horizontal pleiotropy (P > 0.05). The results of the leave-one-out sensitivity analysis suggested that the association between inflammatory cytokines and IPF was not substantially driven by any individual SNP (available in Supplementary material).

## Discussion

The present study performed a two-sample MR analysis to explore genetic evidence to support causal relationships of circulating inflammatory cytokines with the risk of IPF. The current MR study demonstrated that genetically proxied higher circulating IL14 level was causally associated with an increased risk of IPF, whereas no significant associations were found between genetically higher exposures of other circulating inflammatory cytokines levels (circulating CRP, MIP1a, MIP1b, IL-1ra, IL-2ra, IL-2, IL-6, IL-8, IL-10, IL-13, IL-16, IL-17, IL-18, MCP1, MIF, Eotaxin, GROa, MIP1a, MIP1b, RANTES, TANLN, CXCL9, TNFa, TNFb) and IPF. These findings provided epidemiological evidence for drug therapy targeting inflammatory factors in the prevention and treatment of IPF.

IPF is characterized as a profoundly debilitating interstitial lung disease, with a rapid deterioration in lung function and a mortality rate of 50% within 3–5 years following diagnosis [23, 24]. While antifibrotic therapy, such as pirfenidone and nintedanib, has shown efficacy in slowing the decline of lung function in IPF patients, neither treatment has demonstrated a definitive impact on reducing mortality outcomes [25]. Additionally, the currently available therapeutic options for patients with confirmed IPF fail to consistently enhance their overall quality of life. The limited success of these therapeutic approaches can be attributed to the elusive understanding of the underlying pathological mechanisms responsible for fibrosis in IPF, which hampers the development of precise and potent treatment interventions [26, 27]. Thus, the elucidation of early pathological mechanisms in IPF is of paramount importance for unraveling the etiological basis of the disease and identifying potential therapeutic targets.

Inflammation, recognized as a critical component in numerous autoimmune disorders, is widely implicated in various significant pathogenic processes of IPF. These include epithelial-mesenchymal transition, epithelial cell apoptosis, impaired fibrinolytic system, and myofibroblast accumulation, which are considered pivotal in the pathophysiology of IPF [28–30]. The paradigm of IPF pathogenesis has shifted from a fibroblast-driven disease to an epithelium-driven disease. Upon recurrent microinjuries, alveolar type II epithelial cells dysfunction are not only unable to sustain physiological lung regeneration but also promote aberrant epithelial-mesenchymal crosstalk, resulting in a drift towards fibrosis rather than regeneration [2–4]. Multiple preclinical studies have consistently demonstrated a close relationship between the pathogenesis of IPF and inflammatory responses mediated by the transforming growth factor-β (TGF-β) signaling pathway. Accordingly, therapeutic interventions targeting the suppression of TGF-β signaling pathways have shown promising efficacy in ameliorating pulmonary fibrosis [31, 32]. A previous study conducted by Kim MS further reported that IL-37, an anti-inflammatory cytokine, exhibits inhibitory effects on TGF-β1 signaling and enhances autophagy in IPF fibroblasts, ultimately improving the condition of IPF pulmonary fibrosis [33]. Nevertheless, a fundamental question that remains elusive is whether the onset and unfavorable prognosis of IPF are determined by genetically predicted levels of serum inflammatory cytokines or if IPF itself triggers alterations in serum inflammatory cytokine levels. Therefore, to address this inquiry, we endeavored to establish supportive evidence at the genetic level using MR methods.

In our study, we successfully established a causal link between serum IL14 levels and an elevated risk of developing IPF. However, it is important to note that the incremental risk associated with IL14 levels was not substantial, and its clinical significance may be limited. As for the relationship between other inflammatory cytokines and IPF, no causal link has been established thus far. Therefore, based on the current body of research, it is our belief that the pathogenesis of IPF is not triggered by fluctuations in serum inflammatory cytokine levels. Instead, these fluctuations may be a consequence of the progression of IPF. Moreover, our study yielded robust null results for circulating inflammatory cytokines (IL-1ra, IL-2ra, IL-2, IL-6, IL-8, IL-10, IL-18, MIP1a, MIP1b, MCP1, RANTES, TANLN, and CXCL9), indicating that prolonged exposure to elevated levels of these circulating inflammatory cytokines does not increase the risk of IPF. Presently, the blocking of inflammatory cytokines appears to hold promising potential as a therapeutic approach in IPF drug therapy. A phase 2, randomized, double-blind, placebo-controlled trial conducted by Maher et al. in 2021 has recruited about 500 patients with IPF to evaluate the efficacy and safety of lebrikizumab (IL-13 monoclonal antibody), alone or with background pirfenidone therapy [34]. Interestingly, the results didn’t provide valid evidence to demonstrate the casual relationship between IL-13 and developmental risk of IL-13 that blocking IL-13 alone seemed not to achieve sufficient lung function benefits in patients with IPF. Another phase 2 randomized controlled study conducted by Parker et al. in 2018 has also investigated efficacy and safety of human anti-IL-13 monoclonal antibody, tralokinumab, in patients with mild to moderate IPF [35], which indicated an acceptable safety and tolerability profile of IL-13 monoclonal antibody but didn’t achieve key efficacy endpoints. The pathogenesis of IPF is intricate and involves a multitude of inflammatory cytokines. Employing the Mendelian randomization approach to demonstrate a causal relationship between specific serum inflammatory cytokine levels and a reduced risk of IPF would be of considerable interest, as subsequent animal experiments based on these findings could potentially yield novel treatment methods. In our investigation, we explored the causal relationship between the risk of developing IPF and the serum levels of inflammatory cytokines predicted by 24 common genes. Regrettably, we did not observe any individual inflammatory cytokine exhibiting a significant impact on the heightened risk of IPF. Nonetheless, our study furnishes valuable guidance for future drug research, whereby we can explore the causal association between disease and the levels of inflammatory cytokines predicted by genes, thereby allowing us to conduct subsequent experiments guided by the aforementioned screening results.

Several limitations warrant consideration in our study. Firstly, although no causal association was observed, it is possible that the effect size is too small to be detected comprehensively, although this cannot be definitively ruled out. Secondly, the lack of available data pertaining to the pathological classification of IPF hindered our ability to conduct further analyses specifically examining IPF subtypes. As such, further investigation into IPF subtype analysis is necessary to elucidate the potential correlation between levels of circulating inflammatory cytokines and IPF across different subtypes. Thirdly, the identification of IVs was conducted using a relatively stringent threshold (p < 5 × 10^− 6^), which may introduce bias when weak IVs are included. Fourthly, the scope of our study was limited to individuals of European descent, thereby limiting the generalizability of our findings to individuals of other ancestral backgrounds. Consequently, the present study does not provide support for a causal relationship between levels of 27 inflammatory cytokines and IPF. Considering the restricted dataset of inflammatory factors included in our analysis, alternative datasets examining inflammatory factors are needed to validate our findings. Therefore, we anticipate that future studies incorporating updated GWAS data on inflammatory factors will serve to further validate the results of our investigation.

## Conclusion

The high level of IL14 predicted by genes had a casual relationship with the increased risk of IPF. No significant associations were found between genetically higher exposures of other circulating inflammatory cytokines levels and IPF. These findings provided epidemiological evidence for drug therapy targeting inflammatory factors in the prevention and treatment of IPF. It’s still warranted further exploration to validate the clinical significance of IL14 associated with developmental risk of IPF.

### Electronic supplementary material

Below is the link to the electronic supplementary material.

Supplementary Material 1

Supplementary Material 2

Supplementary Material 3

Supplementary Material 4

## Data Availability

The datasets used and/or analysed during the current study are available from the corresponding author on reasonable request. All methods were carried out in accordance with the relevant guidelines and regulations.

## References

[1] Richeldi L, Collard HR, Jones MG (2017). Idiopathic pulmonary fibrosis. Lancet.

[2] Confalonieri P, Volpe MC, Jacob J et al. Regeneration or repair? The role of alveolar epithelial cells in the pathogenesis of idiopathic pulmonary fibrosis (IPF). Cells 2022;11. 10.3390/cells11132095 PMC9266271 35805179

[3] Lacedonia D, Scioscia G, Soccio P (2021). Downregulation of exosomal let-7d and miR-16 in idiopathic pulmonary fibrosis. BMC Pulm Med.

[4] Zhu W, Tan C, Zhang J (2022). Alveolar epithelial type 2 cell dysfunction in idiopathic pulmonary fibrosis. Lung.

[5] Heukels P, Moor CC, von der Thüsen JH (2019). Inflammation and immunity in IPF pathogenesis and treatment. Respir Med.

[6] O’Dwyer DN, Ashley SL, Gurczynski SJ (2019). Lung microbiota contribute to pulmonary inflammation and Disease Progression in Pulmonary Fibrosis. Am J Respir Crit Care Med.

[7] Zhao M, Wang L, Wang M (2022). Targeting fibrosis, mechanisms and cilinical trials. Signal Transduct Target Ther.

[8] Samarelli AV, Tonelli R, Marchioni A et al. Fibrotic idiopathic interstitial lung disease: the Molecular and Cellular Key Players. Int J Mol Sci 2021;22. 10.3390/ijms22168952 PMC8396471 34445658

[9] Salton F, Ruaro B, Confalonieri P et al. Epithelial-mesenchymal transition: a major pathogenic driver in idiopathic pulmonary fibrosis? Medicina (Kaunas) 2020;56. 10.3390/medicina56110608 PMC7697350 33202716

[10] Baratella E, Ruaro B, Giudici F et al. Evaluation of correlations between genetic variants and high-resolution computed tomography patterns in idiopathic pulmonary fibrosis. Diagnostics (Basel) 2021;11. 10.3390/diagnostics11050762 PMC8146750 33922858

[11] Lee JH, Park HJ, Kim S (2023). Epidemiology and comorbidities in idiopathic pulmonary fibrosis: a nationwide cohort study. BMC Pulm Med.

[12] Ma WH, Li M, Ma HF (2020). Protective effects of GHK-Cu in bleomycin-induced pulmonary fibrosis via anti-oxidative stress and anti-inflammation pathways. Life Sci.

[13] Doni A, Mantovani A, Bottazzi B (2021). PTX3 regulation of inflammation, hemostatic response, tissue repair, and Resolution of Fibrosis favors a role in limiting idiopathic pulmonary fibrosis. Front Immunol.

[14] Xiong Y, Cui X, Zhou Y (2021). Dehydrocostus lactone inhibits BLM-induced pulmonary fibrosis and inflammation in mice via the JNK and p38 MAPK-mediated NF-κB signaling pathways. Int Immunopharmacol.

[15] Li S, Yang Q, Chen F (2022). The antifibrotic effect of pheretima protein is mediated by the TGF-β1/Smad2/3 pathway and attenuates inflammation in bleomycin-induced idiopathic pulmonary fibrosis. J Ethnopharmacol.

[16] Birney E. Mendelian randomization. Cold Spring Harb Perspect Med 2022;12. 10.1101/cshperspect.a041302 PMC9121891 34872952

[17] Zhu Q, Nguyen DT, Sid E (2020). Leveraging the UMLS as a Data Standard for Rare Disease Data normalization and harmonization. Methods Inf Med.

[18] Smith GD, Ebrahim S (2003). Mendelian randomization’: can genetic epidemiology contribute to understanding environmental determinants of disease?. Int J Epidemiol.

[19] Perry BI, Upthegrove R, Kappelmann N (2021). Associations of immunological proteins/traits with schizophrenia, major depression and bipolar disorder: a bi-directional two-sample mendelian randomization study. Brain Behav Immun.

[20] Sekula P, Del Greco MF, Pattaro C (2016). Mendelian randomization as an Approach to assess causality using Observational Data. J Am Soc Nephrol.

[21] Ahola-Olli AV, Würtz P, Havulinna AS (2017). Genome-wide Association Study identifies 27 loci influencing concentrations of circulating cytokines and growth factors. Am J Hum Genet.

[22] Duckworth A, Gibbons MA, Allen RJ (2021). Telomere length and risk of idiopathic pulmonary fibrosis and chronic obstructive pulmonary disease: a mendelian randomisation study. Lancet Respir Med.

[23] Raghu G, Remy-Jardin M, Myers JL (2018). Diagnosis of idiopathic pulmonary fibrosis. An Official ATS/ERS/JRS/ALAT Clinical Practice Guideline. Am J Respir Crit Care Med.

[24] Ley B, Collard HR, King TE (2011). Clinical course and prediction of survival in idiopathic pulmonary fibrosis. Am J Respir Crit Care Med.

[25] Canestaro WJ, Forrester SH, Raghu G (2016). Drug Treatment of Idiopathic Pulmonary Fibrosis: systematic review and network Meta-analysis. Chest.

[26] Noble PW, Albera C, Bradford WZ (2011). Pirfenidone in patients with idiopathic pulmonary fibrosis (CAPACITY): two randomised trials. Lancet.

[27] Richeldi L, du Bois RM, Raghu G (2014). Efficacy and safety of nintedanib in idiopathic pulmonary fibrosis. N Engl J Med.

[28] Bringardner BD, Baran CP, Eubank TD (2008). The role of inflammation in the pathogenesis of idiopathic pulmonary fibrosis. Antioxid Redox Signal.

[29] Fathimath Muneesa M, Shaikh SB, Jeena TM (2021). Inflammatory mediators in various molecular pathways involved in the development of pulmonary fibrosis. Int Immunopharmacol.

[30] Guo X, Sunil C, Qian G (2021). Obesity and the development of lung fibrosis. Front Pharmacol.

[31] Dai WJ, Qiu J, Sun J (2019). Downregulation of microRNA-9 reduces inflammatory response and fibroblast proliferation in mice with idiopathic pulmonary fibrosis through the ANO1-mediated TGF-β-Smad3 pathway. J Cell Physiol.

[32] Park SJ, Kim TH, Lee K et al. Kurarinone attenuates BLM-Induced Pulmonary Fibrosis via inhibiting TGF-β signaling pathways. Int J Mol Sci 2021;22. 10.3390/ijms22168388 PMC8395032 34445094

[33] Kim MS, Baek AR, Lee JH (2019). IL-37 attenuates Lung Fibrosis by Inducing Autophagy and regulating TGF-β1 production in mice. J Immunol.

[34] Maher TM, Costabel U, Glassberg MK et al. Phase 2 trial to assess lebrikizumab in patients with idiopathic pulmonary fibrosis. Eur Respir J 2021;57. 10.1183/13993003.02442-2019 PMC7859504 33008934

[35] Parker JM, Glaspole IN, Lancaster LH (2018). A phase 2 Randomized Controlled Study of Tralokinumab in subjects with idiopathic pulmonary fibrosis. Am J Respir Crit Care Med.

