# Supplementary material for: Circulating inflammatory cytokines and risk of idiopathic pulmonary fibrosis: a Mendelian randomization study
Source: BMC Pulm Med. 2023 Oct 3;23:369. doi: 10.1186/s12890-023-02658-3 (PMC10548733; doi:10.1186/s12890-023-02658-3)
Supplement: Supplementary file 1 — Supplementary Material 1 [file 12890_2023_2658_MOESM1_ESM.docx]

**Supplementary material**

**IL-2**

**Scatter plot to visualize the causal effect of IL-2 on idiopathic pulmonary fibrosis. The slope of the straight line indicates the magnitude of the causal association. IVW indicates inverse-variance weighted, and MR, Mendelian randomization.**

**
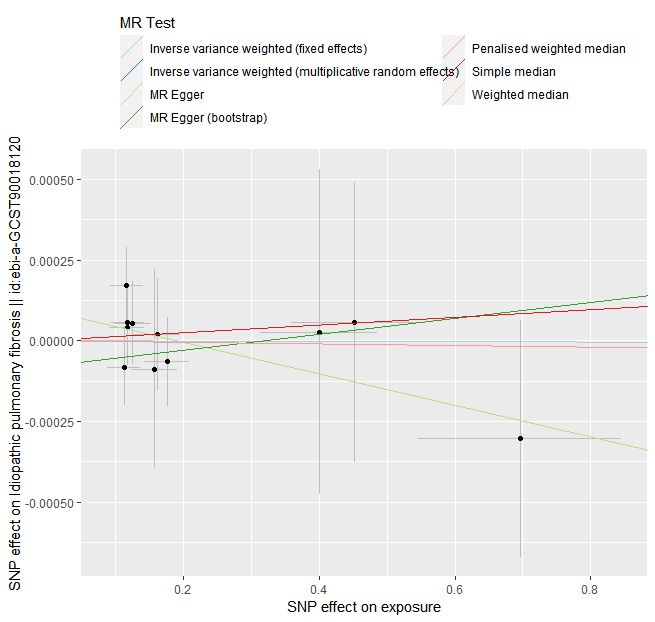
**

**Fixed-effect IVW analysis and of the causal association of IL-2 with idiopathic pulmonary fibrosis. The black dots and bars indicated the causal estimate and 95% CI using each SNP. The red dot and bar indicated the overall estimate and 95% CI meta-analyzed by fixed-effect inverse variance weighted** **method and MR-Egger method.**

**
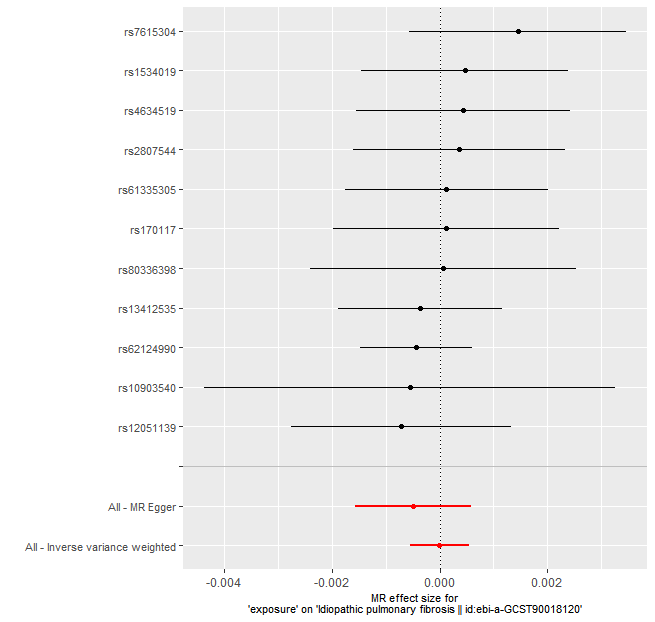
**

**MR leave-one-out sensitivity analysis for** **IL-2 on idiopathic pulmonary fibrosis. Circles indicate MR estimates for IL2 on idiopathic pulmonary fibrosis using inverse-variance weighted fixed-effect method if each single nucleotide polymorphism was omitted. The bars indicate the CI. MR indicates Mendelian randomization.**

**
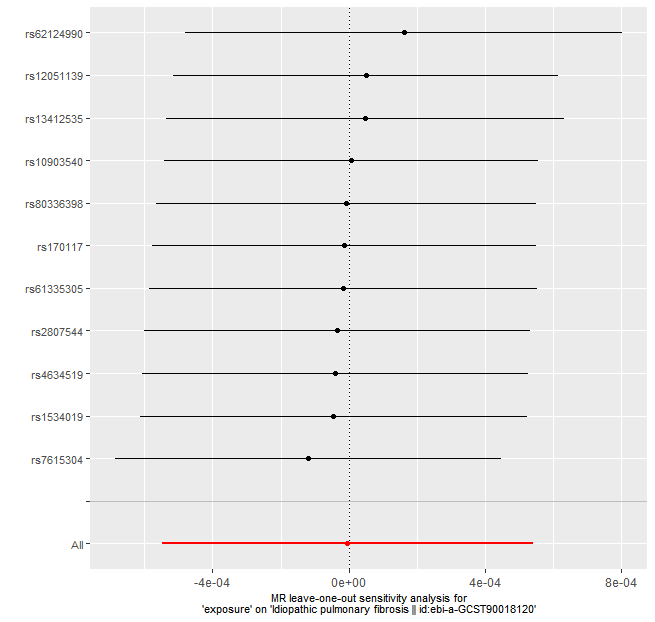
**

****IL-6****

**Scatter plot to visualize the causal effect of **IL-6** on idiopathic pulmonary fibrosis. The slope of the straight line indicates the magnitude of the causal association. IVW indicates inverse-variance weighted, and MR, Mendelian randomization.**

**
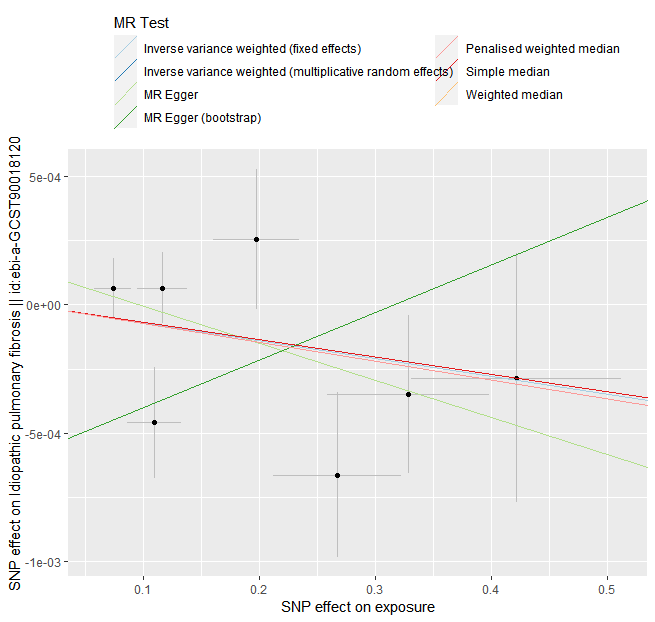
**

**Fixed-effect IVW analysis and of the causal association of IL-6 with idiopathic pulmonary fibrosis. The black dots and bars indicated the causal estimate and 95% CI using each SNP. The red dot and bar indicated the overall estimate and 95% CI meta-analyzed by fixed-effect inverse variance weighted method and MR-Egger method**

**
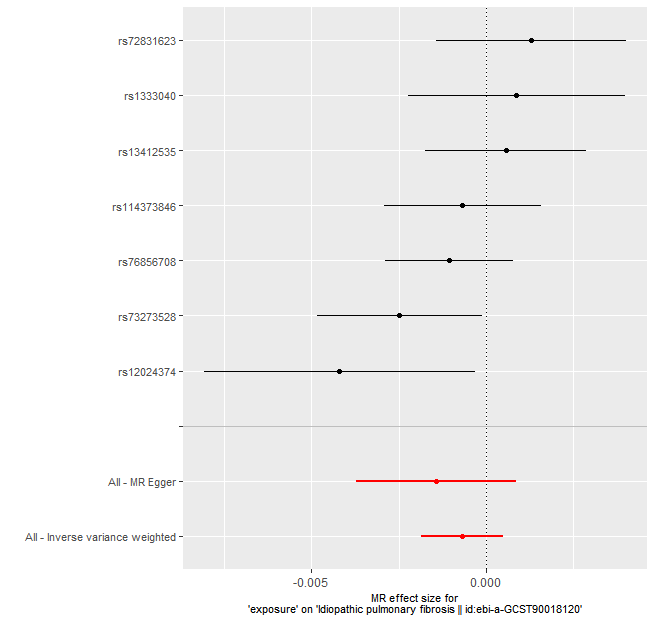
**

**MR leave-one-out sensitivity analysis for IL-6 on idiopathic pulmonary fibrosis. Circles indicate MR estimates for IL-6 on idiopathic pulmonary fibrosis using inverse-variance weighted fixed-effect method if each single nucleotide polymorphism was omitted. The bars indicate the CI. MR indicates Mendelian randomization.**

**
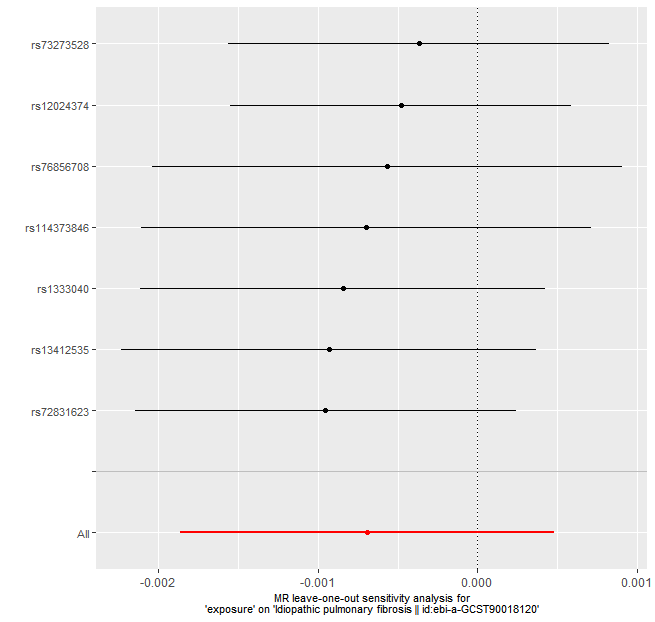
**

**Scatter plot to visualize the causal effect of **IL-8** on idiopathic pulmonary fibrosis. The slope of the straight line indicates the magnitude of the causal association. IVW indicates inverse-variance weighted, and MR, Mendelian randomization.**

**
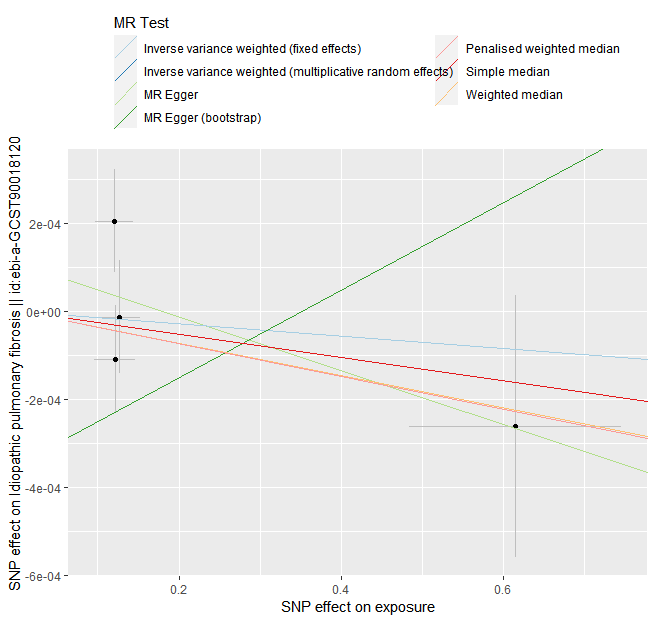
**

**Fixed-effect IVW analysis and of the causal association of IL-8 with idiopathic pulmonary fibrosis. The black dots and bars indicated the causal estimate and 95% CI using each SNP. The red dot and bar indicated the overall estimate and 95% CI meta-analyzed by fixed-effect inverse variance weighted method and MR-Egger method**

**
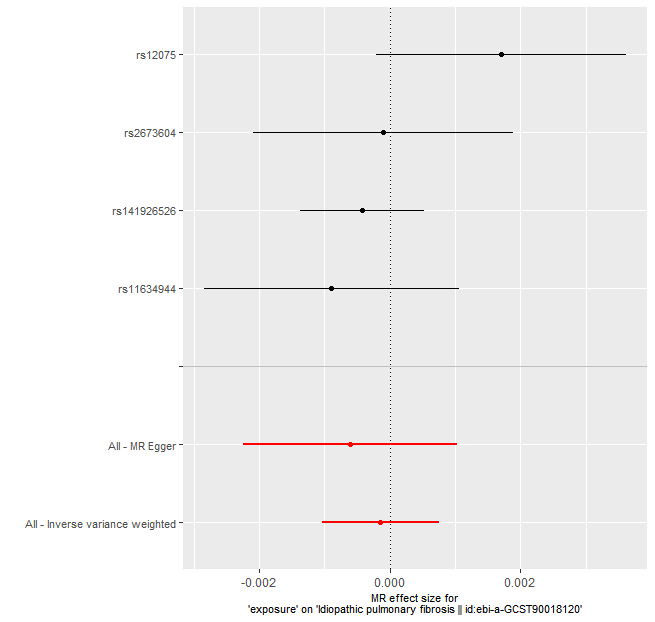
**

**MR leave-one-out sensitivity analysis for IL-8 on idiopathic pulmonary fibrosis. Circles indicate MR estimates for IL-8 on idiopathic pulmonary fibrosis using inverse-variance weighted fixed-effect method if each single nucleotide polymorphism was omitted. The bars indicate the CI. MR indicates Mendelian randomization.**

**
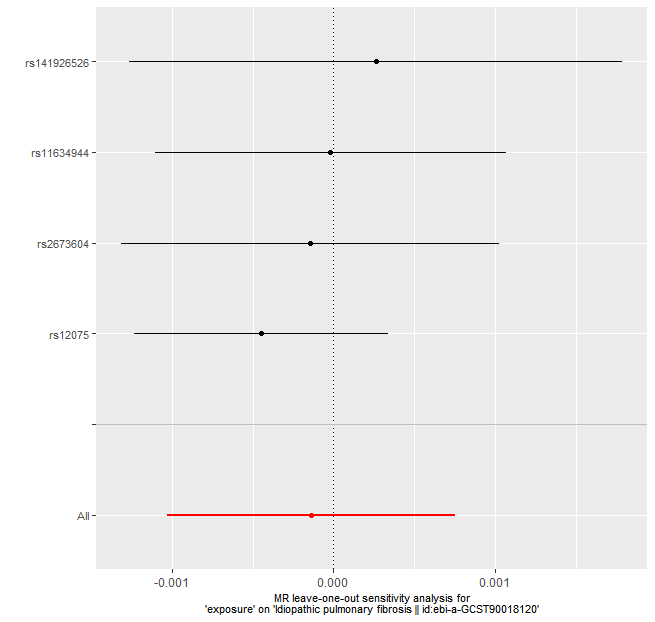
**

**Scatter plot to visualize the causal effect of **IL-10** on idiopathic pulmonary fibrosis. The slope of the straight line indicates the magnitude of the causal association. IVW indicates inverse-variance weighted, and MR, Mendelian randomization.**

**
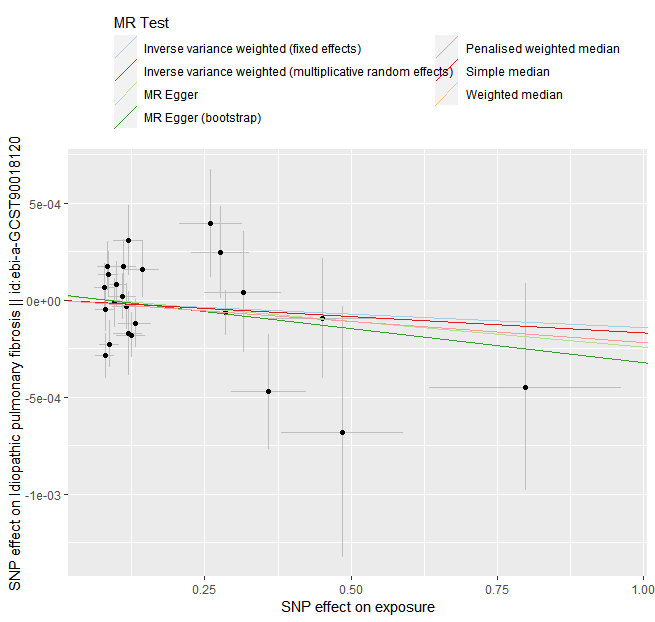
**

**Fixed-effect IVW analysis and of the causal association of IL-10 with idiopathic pulmonary fibrosis. The black dots and bars indicated the causal estimate and 95% CI using each SNP. The red dot and bar indicated the overall estimate and 95% CI meta-analyzed by fixed-effect inverse variance weighted method and MR-Egger method**

**
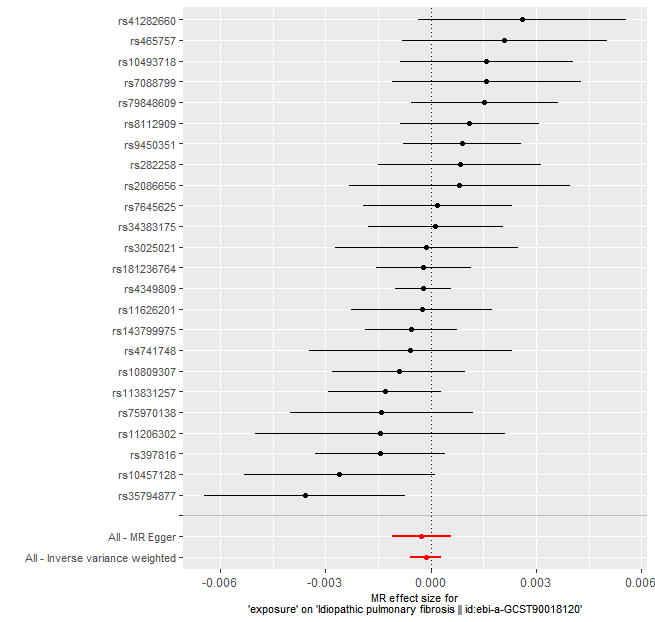
**

**MR leave-one-out sensitivity analysis for IL-10 on idiopathic pulmonary fibrosis. Circles indicate MR estimates for IL-10 on idiopathic pulmonary fibrosis using inverse-variance weighted fixed-effect method if each single nucleotide polymorphism was omitted. The bars indicate the CI. MR indicates Mendelian randomization.**

**
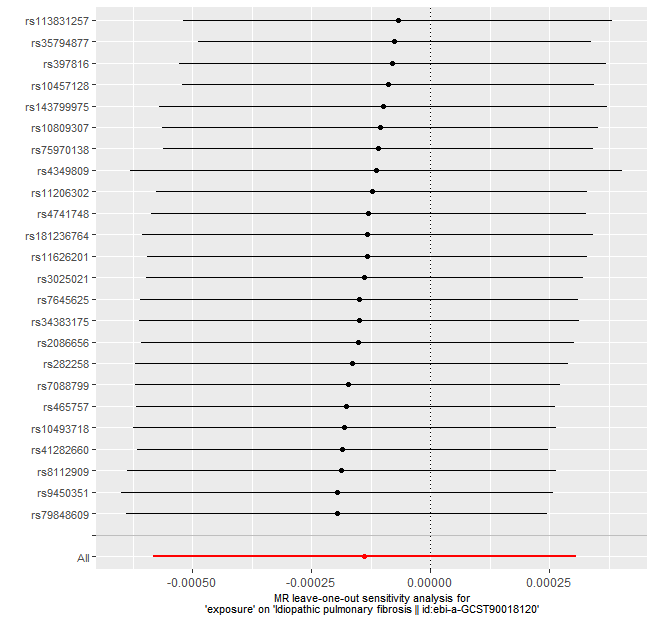
**

**Scatter plot to visualize the causal effect of **IL-13** on idiopathic pulmonary fibrosis. The slope of the straight line indicates the magnitude of the causal association. IVW indicates inverse-variance weighted, and MR, Mendelian randomization.**

**
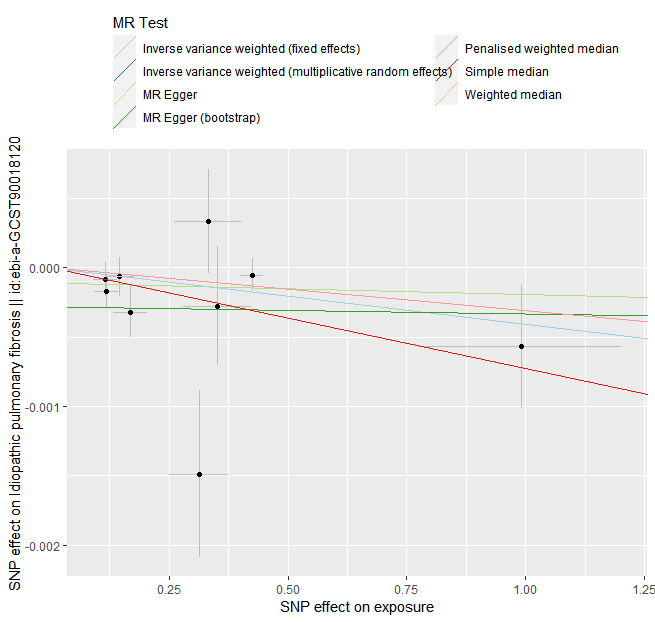
**

**Fixed-effect IVW analysis and of the causal association of IL-13 with idiopathic pulmonary fibrosis. The black dots and bars indicated the causal estimate and 95% CI using each SNP. The red dot and bar indicated the overall estimate and 95% CI meta-analyzed by fixed-effect inverse variance weighted method and MR-Egger method**

**
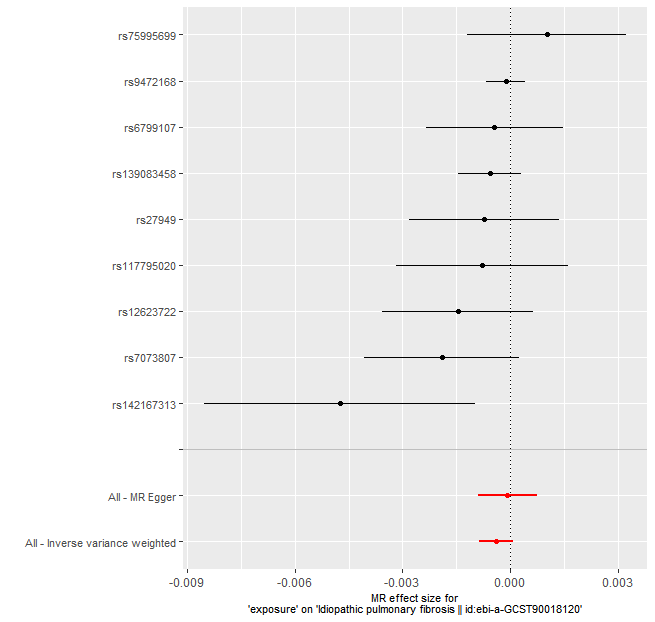
**

**MR leave-one-out sensitivity analysis for IL-13 on idiopathic pulmonary fibrosis. Circles indicate MR estimates for IL-13 on idiopathic pulmonary fibrosis using inverse-variance weighted fixed-effect method if each single nucleotide polymorphism was omitted. The bars indicate the CI. MR indicates Mendelian randomization.**

**
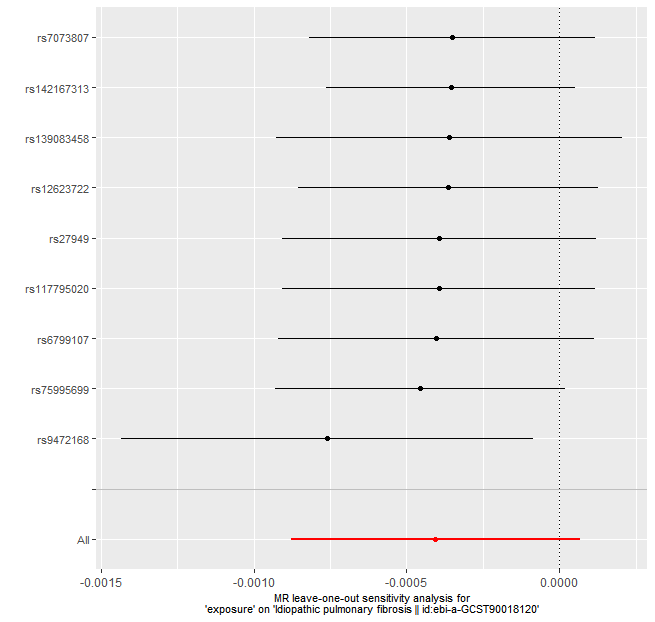
**

**Scatter plot to visualize the causal effect of **IL-14** on idiopathic pulmonary fibrosis. The slope of the straight line indicates the magnitude of the causal association. IVW indicates inverse-variance weighted, and MR, Mendelian randomization.**

**
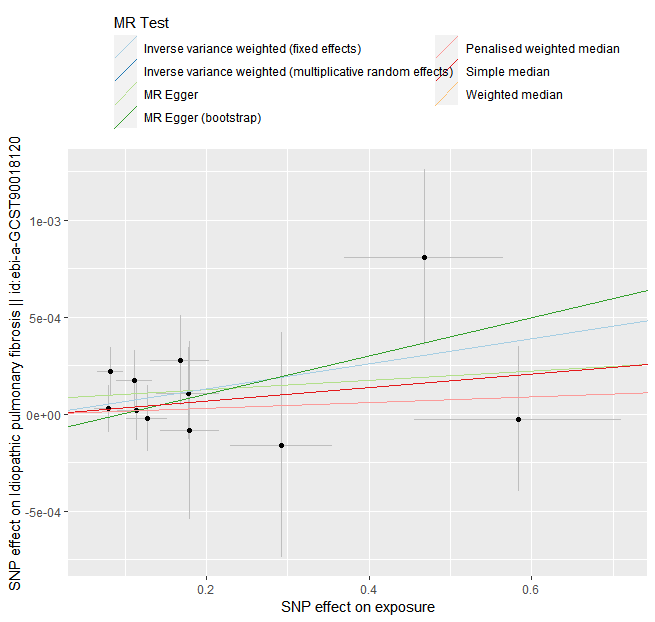
**

**Fixed-effect IVW analysis and of the causal association of IL-14 with idiopathic pulmonary fibrosis. The black dots and bars indicated the causal estimate and 95% CI using each SNP. The red dot and bar indicated the overall estimate and 95% CI meta-analyzed by fixed-effect inverse variance weighted method and MR-Egger method**

**
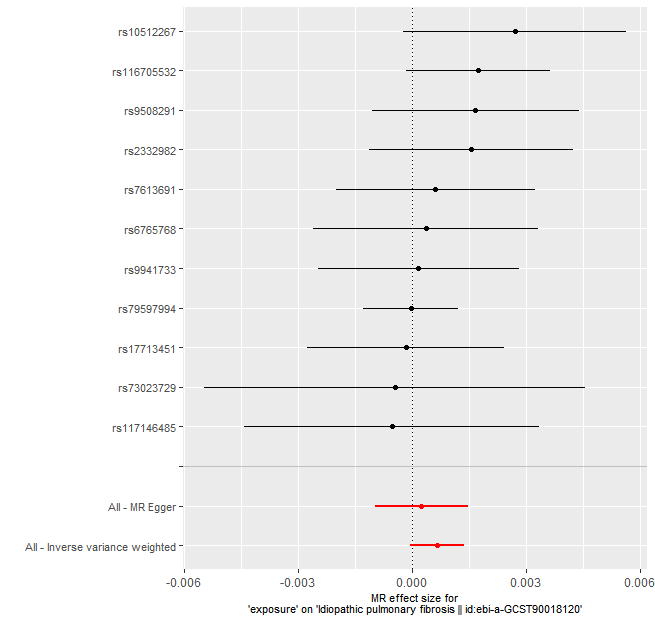
**

**MR leave-one-out sensitivity analysis for IL-14 on idiopathic pulmonary fibrosis. Circles indicate MR estimates for IL-14 on idiopathic pulmonary fibrosis using inverse-variance weighted fixed-effect method if each single nucleotide polymorphism was omitted. The bars indicate the CI. MR indicates Mendelian randomization.**

**
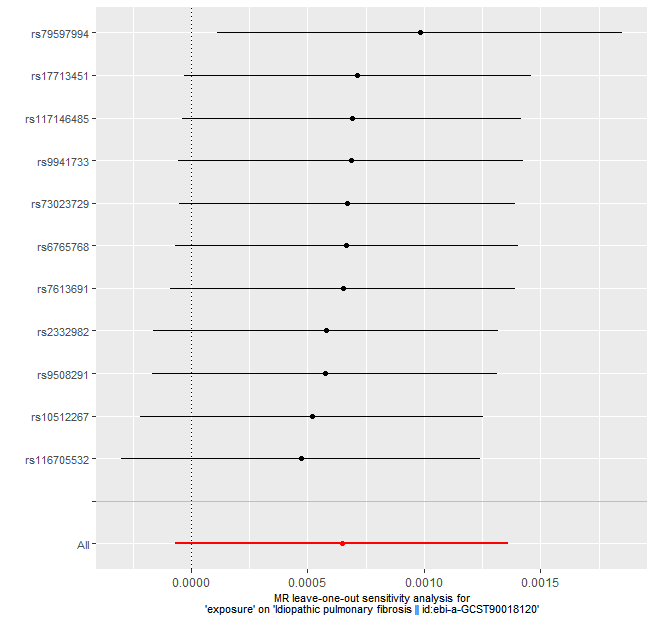
**

**Scatter plot to visualize the causal effect of **IL-16** on idiopathic pulmonary fibrosis. The slope of the straight line indicates the magnitude of the causal association. IVW indicates inverse-variance weighted, and MR, Mendelian randomization.**

**
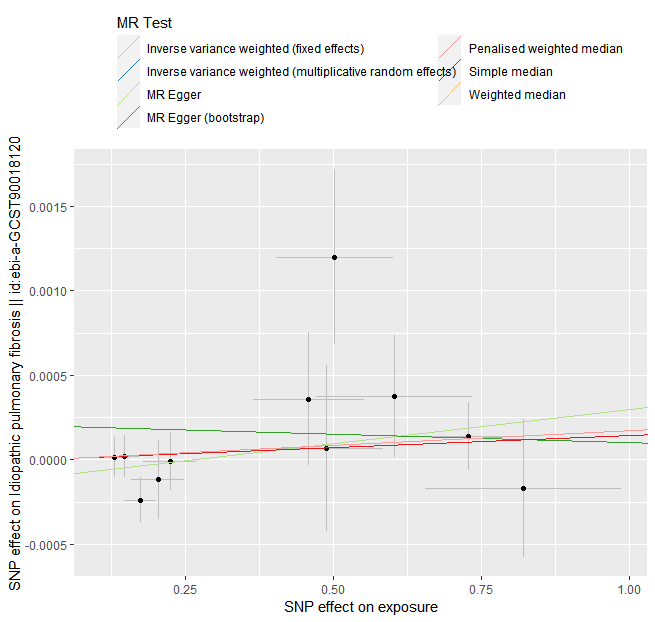
**

**Fixed-effect IVW analysis and of the causal association of IL-16 with idiopathic pulmonary fibrosis. The black dots and bars indicated the causal estimate and 95% CI using each SNP. The red dot and bar indicated the overall estimate and 95% CI meta-analyzed by fixed-effect inverse variance weighted method and MR-Egger method**

**
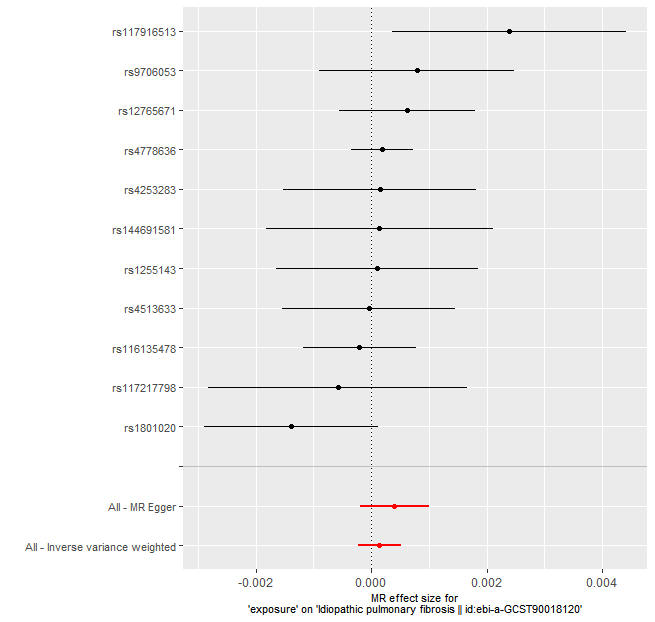
**

**MR leave-one-out sensitivity analysis for IL-16 on idiopathic pulmonary fibrosis. Circles indicate MR estimates for IL-16 on idiopathic pulmonary fibrosis using inverse-variance weighted fixed-effect method if each single nucleotide polymorphism was omitted. The bars indicate the CI. MR indicates Mendelian randomization.**

**
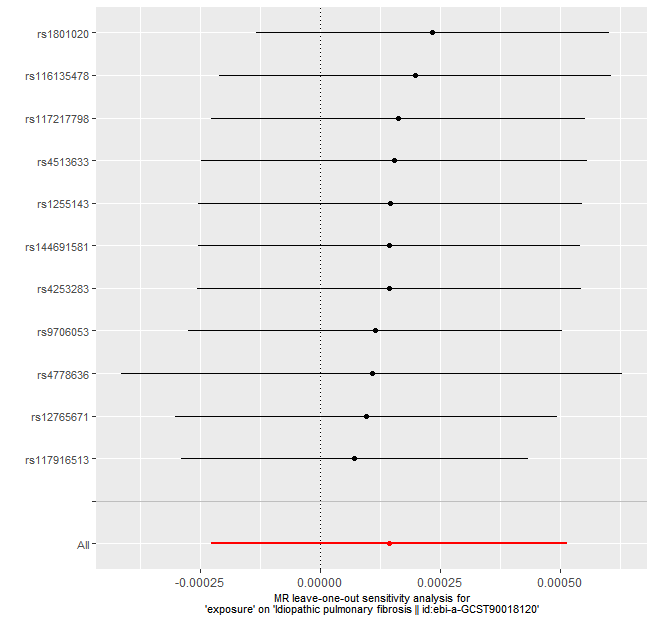
**

**Scatter plot to visualize the causal effect of **IL-17** on idiopathic pulmonary fibrosis. The slope of the straight line indicates the magnitude of the causal association. IVW indicates inverse-variance weighted, and MR, Mendelian randomization.**

**
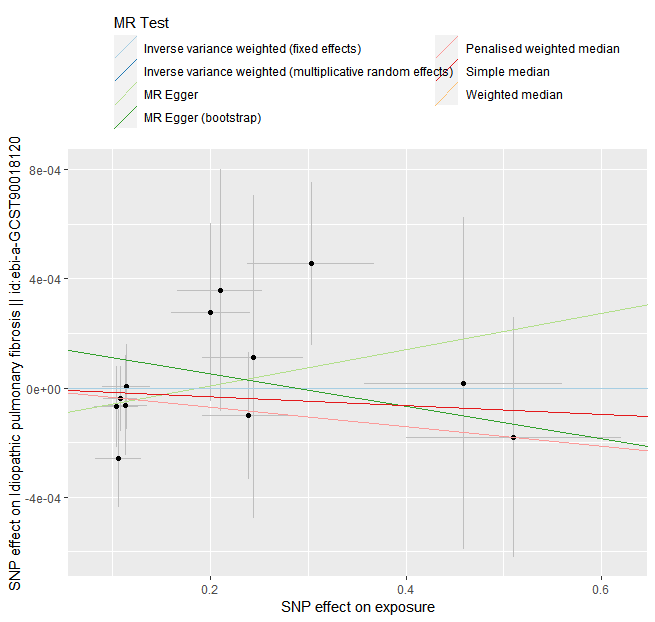
**

**Fixed-effect IVW analysis and of the causal association of IL-17 with idiopathic pulmonary fibrosis. The black dots and bars indicated the causal estimate and 95% CI using each SNP. The red dot and bar indicated the overall estimate and 95% CI meta-analyzed by fixed-effect inverse variance weighted method and MR-Egger method**

**
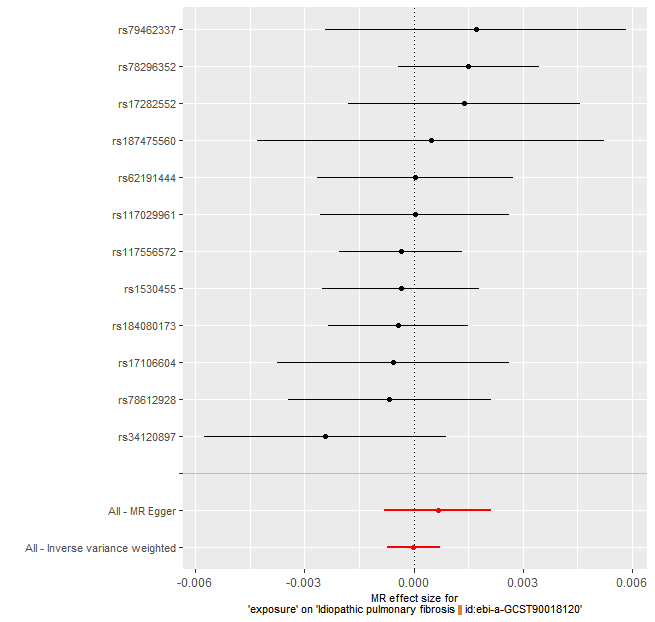
**

**MR leave-one-out sensitivity analysis for IL-17 on idiopathic pulmonary fibrosis. Circles indicate MR estimates for IL-17 on idiopathic pulmonary fibrosis using inverse-variance weighted fixed-effect method if each single nucleotide polymorphism was omitted. The bars indicate the CI. MR indicates Mendelian randomization.**

**
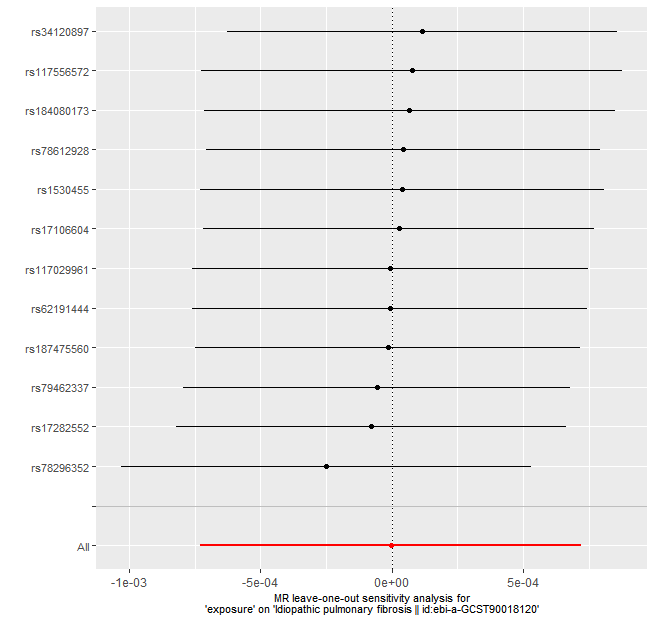
**

**Scatter plot to visualize the causal effect of **IL-18** on idiopathic pulmonary fibrosis. The slope of the straight line indicates the magnitude of the causal association. IVW indicates inverse-variance weighted, and MR, Mendelian randomization.**

**
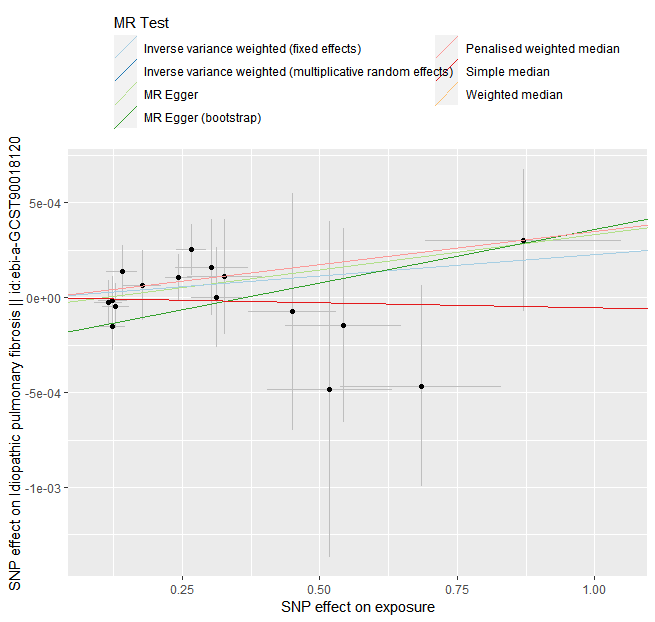
**

**Fixed-effect IVW analysis and of the causal association of IL-18 with idiopathic pulmonary fibrosis. The black dots and bars indicated the causal estimate and 95% CI using each SNP. The red dot and bar indicated the overall estimate and 95% CI meta-analyzed by fixed-effect inverse variance weighted method and MR-Egger method**

**
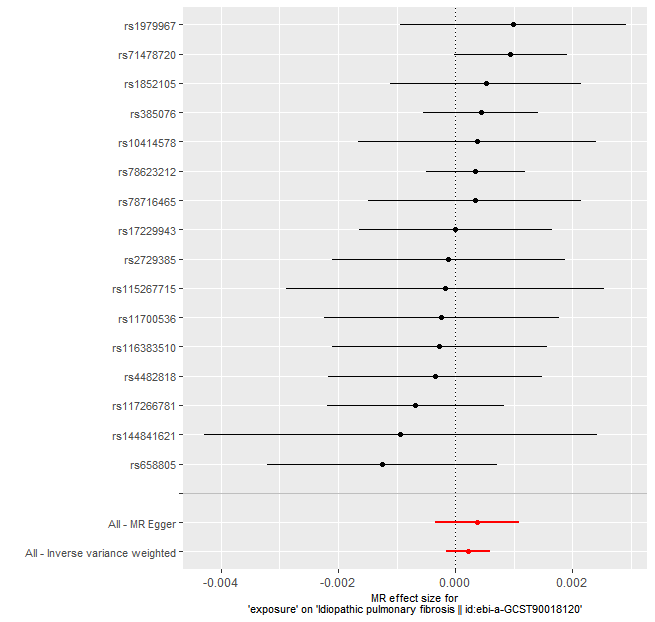
**

**MR leave-one-out sensitivity analysis for IL-18 on idiopathic pulmonary fibrosis. Circles indicate MR estimates for IL-18 on idiopathic pulmonary fibrosis using inverse-variance weighted fixed-effect method if each single nucleotide polymorphism was omitted. The bars indicate the CI. MR indicates Mendelian randomization.**

**
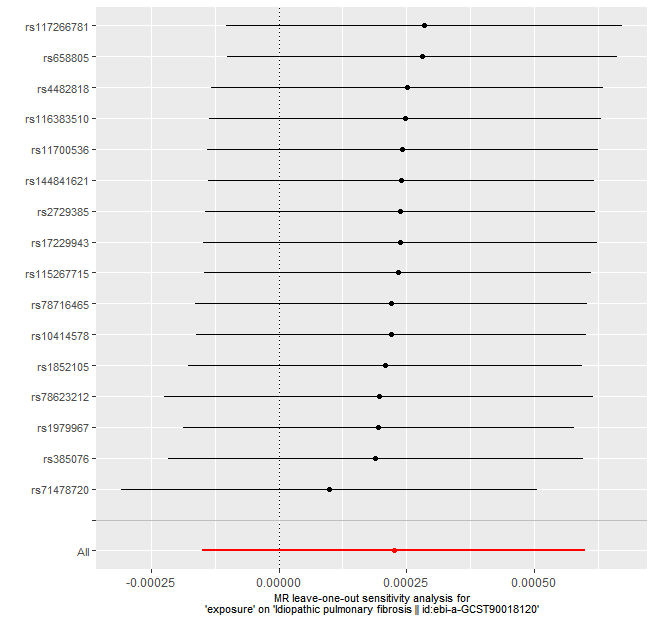
**

**Scatter plot to visualize the causal effect of **IL1ra** on idiopathic pulmonary fibrosis. The slope of the straight line indicates the magnitude of the causal association. IVW indicates inverse-variance weighted, and MR, Mendelian randomization.**

**
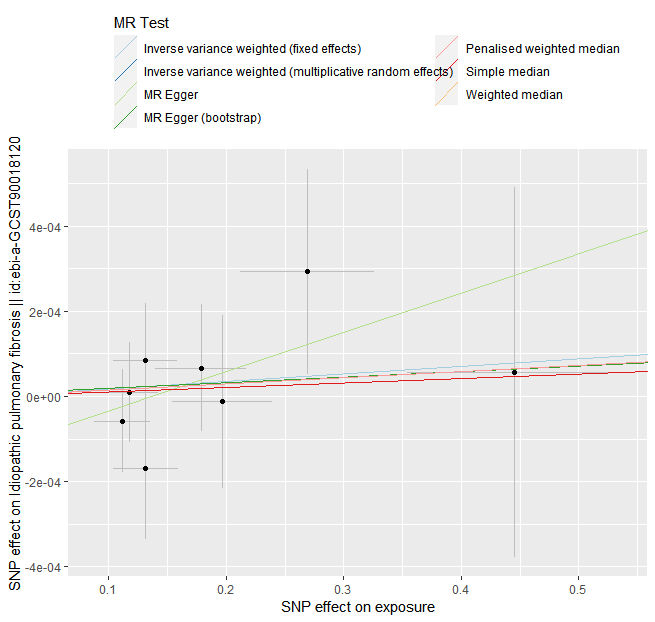
**

**Fixed-effect IVW analysis and of the causal association of IL1ra with idiopathic pulmonary fibrosis. The black dots and bars indicated the causal estimate and 95% CI using each SNP. The red dot and bar indicated the overall estimate and 95% CI meta-analyzed by fixed-effect inverse variance weighted method and MR-Egger method**

**
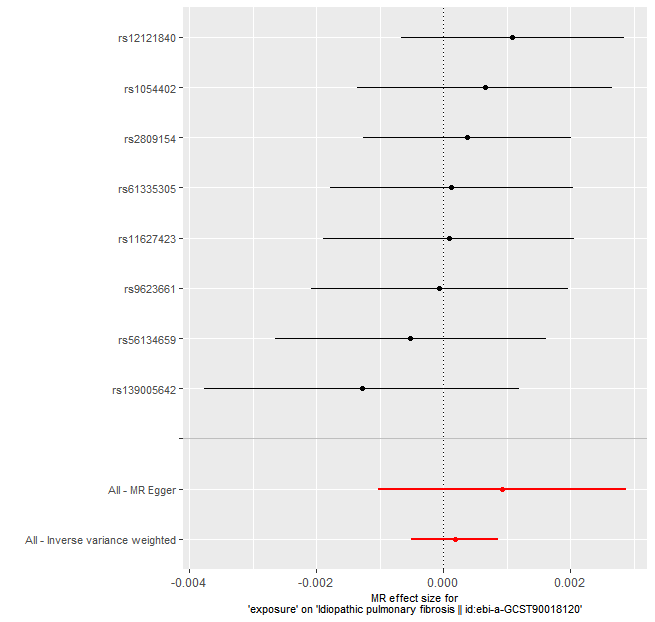
**

**MR leave-one-out sensitivity analysis for IL1ra on idiopathic pulmonary fibrosis. Circles indicate MR estimates for IL1ra on idiopathic pulmonary fibrosis using inverse-variance weighted fixed-effect method if each single nucleotide polymorphism was omitted. The bars indicate the CI. MR indicates Mendelian randomization.**

**
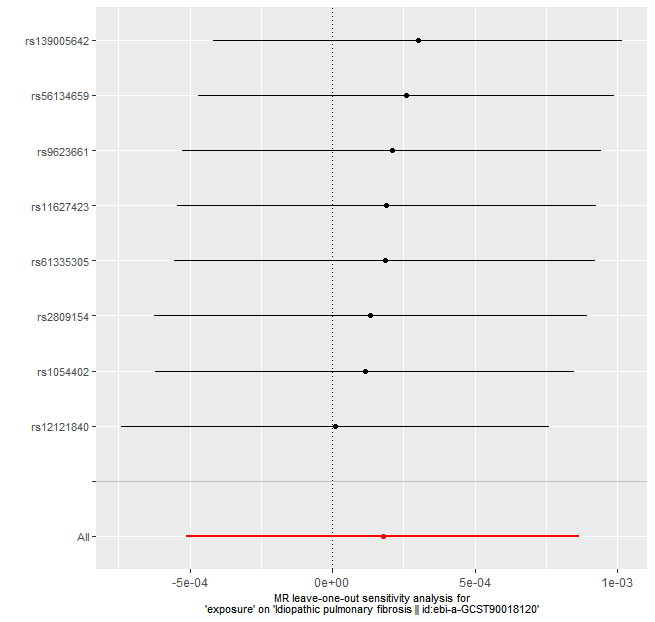
**

**Scatter plot to visualize the causal effect of **IL2ra** on idiopathic pulmonary fibrosis. The slope of the straight line indicates the magnitude of the causal association. IVW indicates inverse-variance weighted, and MR, Mendelian randomization.**

**
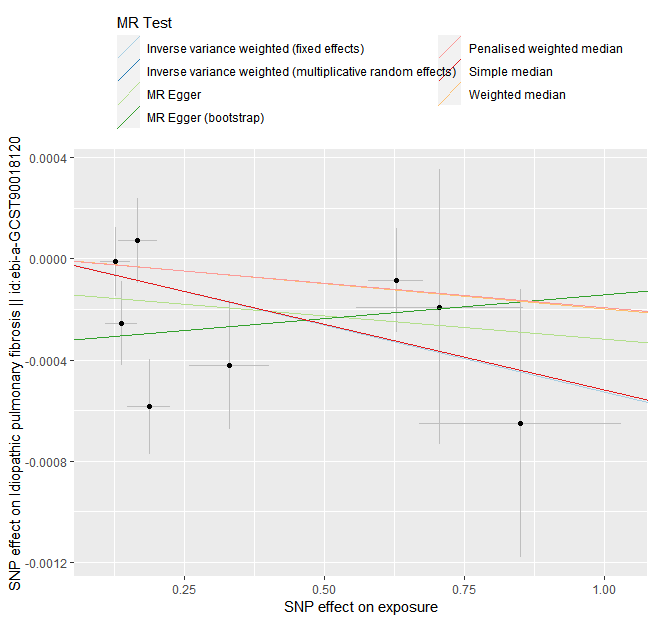
**

**Fixed-effect IVW analysis and of the causal association of **IL2ra**  with idiopathic pulmonary fibrosis. The black dots and bars indicated the causal estimate and 95% CI using each SNP. The red dot and bar indicated the overall estimate and 95% CI meta-analyzed by fixed-effect inverse variance weighted method and MR-Egger method**

**
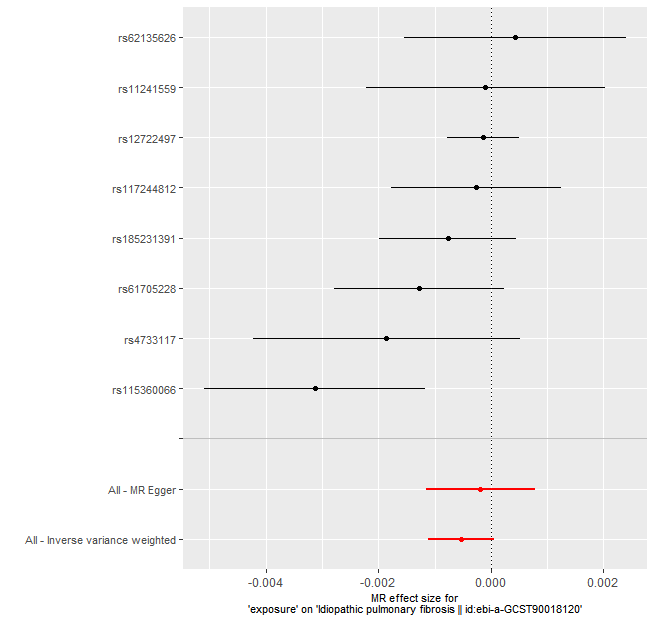
**

**MR leave-one-out sensitivity analysis for **IL2ra**  on idiopathic pulmonary fibrosis. Circles indicate MR estimates for IL2ra on idiopathic pulmonary fibrosis using inverse-variance weighted fixed-effect method if each single nucleotide polymorphism was omitted. The bars indicate the CI. MR indicates Mendelian randomization.**

**
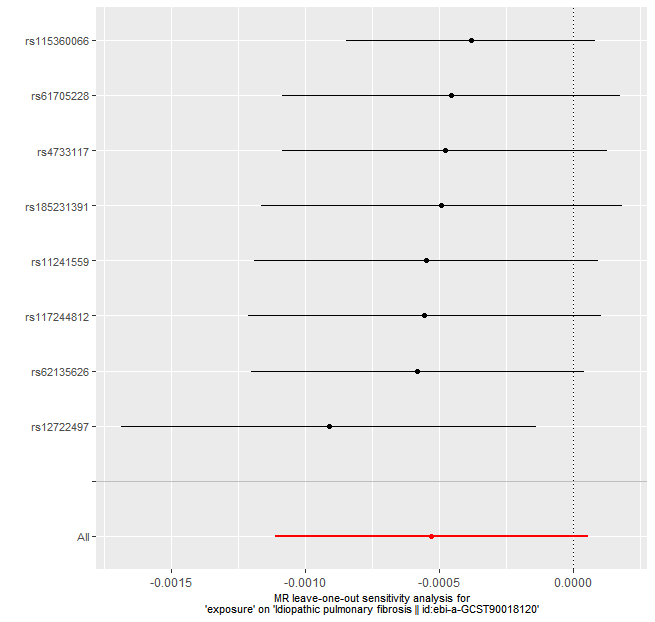
**

**Scatter plot to visualize the causal effect of **CRP** on idiopathic pulmonary fibrosis. The slope of the straight line indicates the magnitude of the causal association. IVW indicates inverse-variance weighted, and MR, Mendelian randomization.**

**
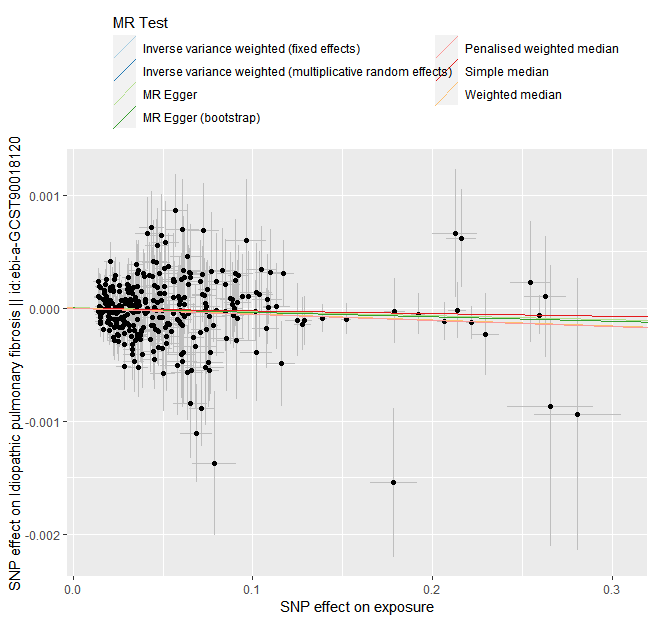
**

**Fixed-effect IVW analysis and of the causal association of **CRP** with idiopathic pulmonary fibrosis. The black dots and bars indicated the causal estimate and 95% CI using each SNP. The red dot and bar indicated the overall estimate and 95% CI meta-analyzed by fixed-effect inverse variance weighted method and MR-Egger method**

**
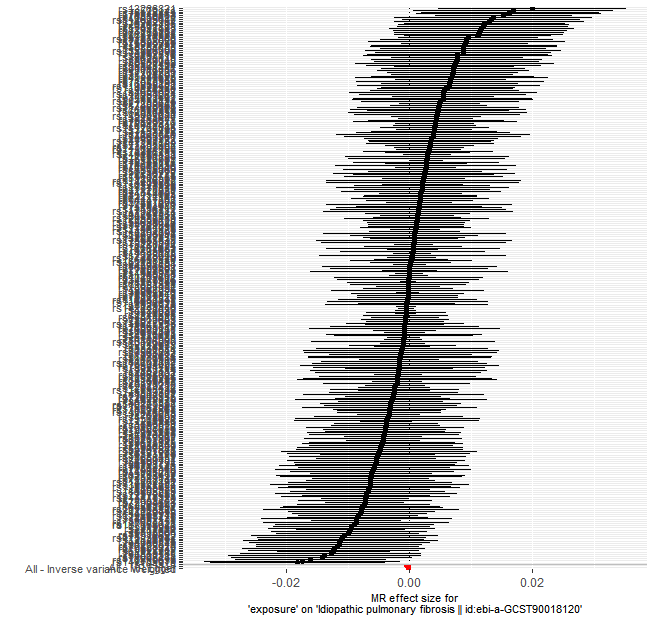
**

**MR leave-one-out sensitivity analysis for **CRP**  on idiopathic pulmonary fibrosis. Circles indicate MR estimates for CRP on idiopathic pulmonary fibrosis using inverse-variance weighted fixed-effect method if each single nucleotide polymorphism was omitted. The bars indicate the CI. MR indicates Mendelian randomization.**

**
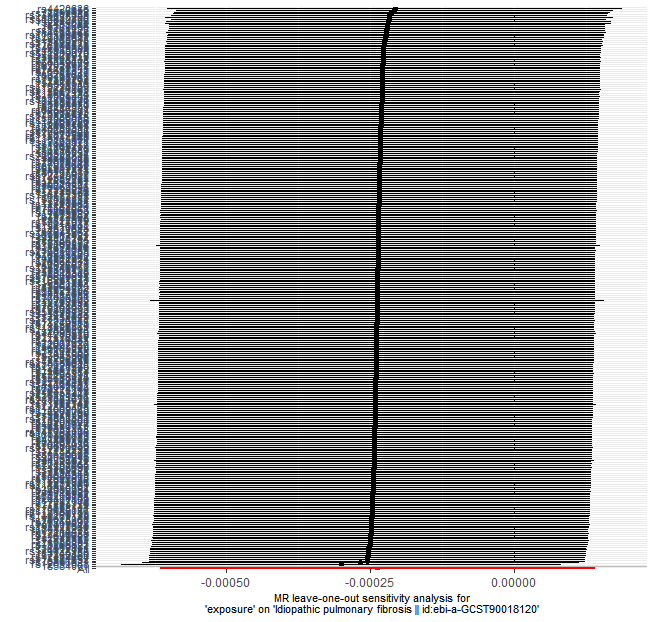
**

**Scatter plot to visualize the causal effect of **CXCL9** on idiopathic pulmonary fibrosis. The slope of the straight line indicates the magnitude of the causal association. IVW indicates inverse-variance weighted, and MR, Mendelian randomization.**

**
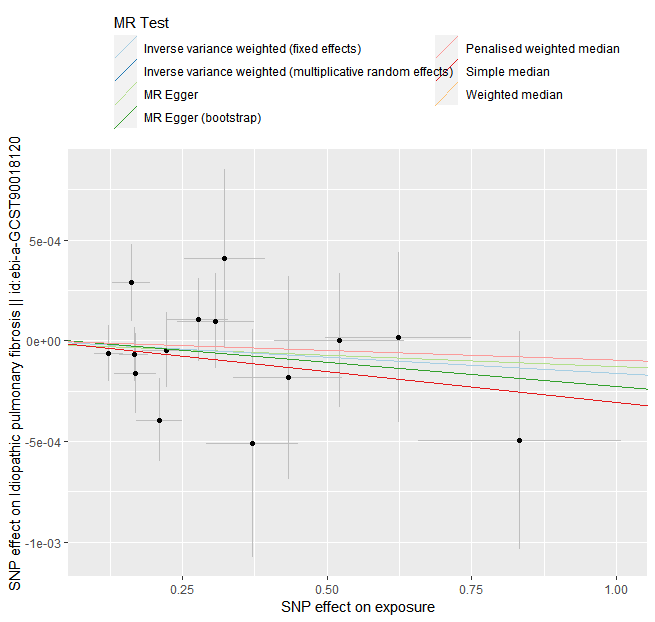
**

**Fixed-effect IVW analysis and of the causal association of **CXCL9** with idiopathic pulmonary fibrosis. The black dots and bars indicated the causal estimate and 95% CI using each SNP. The red dot and bar indicated the overall estimate and 95% CI meta-analyzed by fixed-effect inverse variance weighted method and MR-Egger method**

**
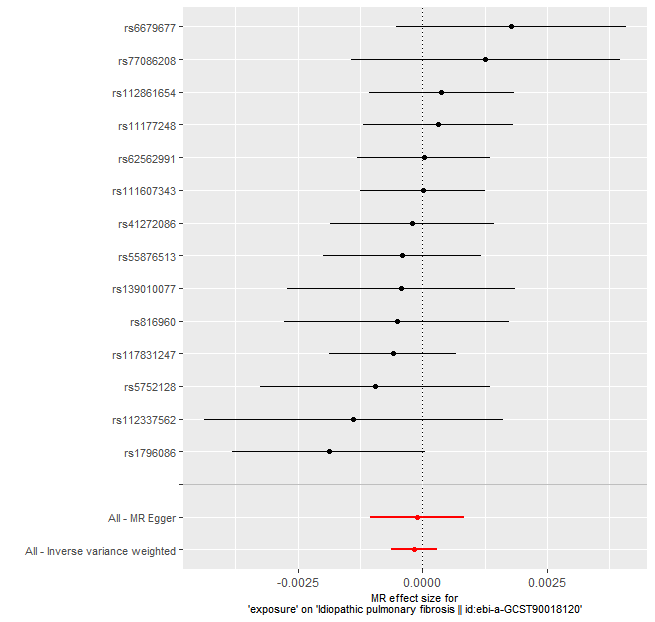
**

**MR leave-one-out sensitivity analysis for **CXCL9**  on idiopathic pulmonary fibrosis. Circles indicate MR estimates for CXCL9 on idiopathic pulmonary fibrosis using inverse-variance weighted fixed-effect method if each single nucleotide polymorphism was omitted. The bars indicate the CI. MR indicates Mendelian randomization.**

**
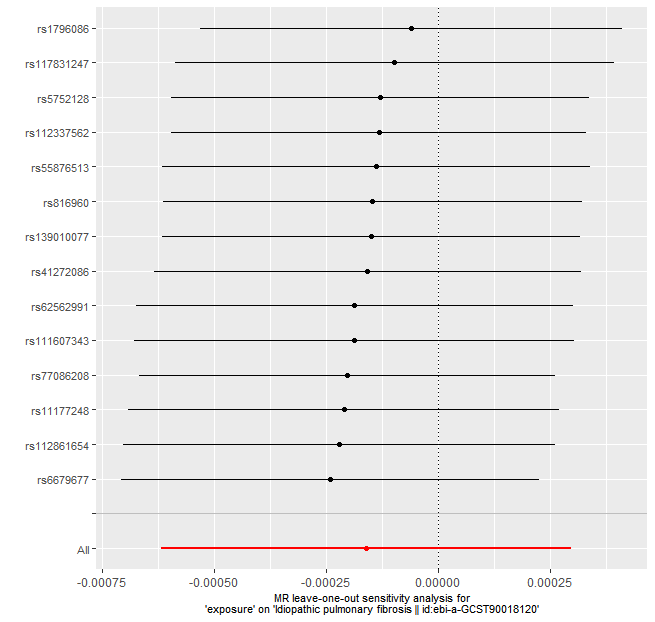
**

**Scatter plot to visualize the causal effect of **Eotaxin** on idiopathic pulmonary fibrosis. The slope of the straight line indicates the magnitude of the causal association. IVW indicates inverse-variance weighted, and MR, Mendelian randomization.**

**
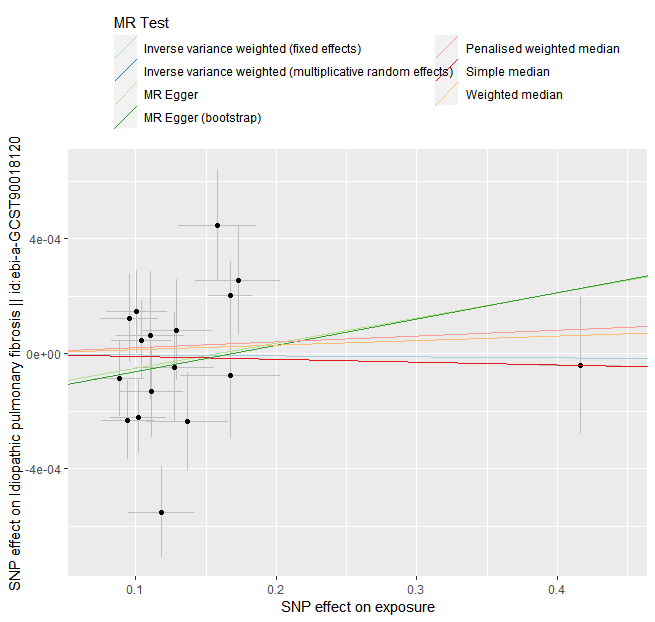
**

**Fixed-effect IVW analysis and of the causal association of **Eotaxin** with idiopathic pulmonary fibrosis. The black dots and bars indicated the causal estimate and 95% CI using each SNP. The red dot and bar indicated the overall estimate and 95% CI meta-analyzed by fixed-effect inverse variance weighted method and MR-Egger method**

**
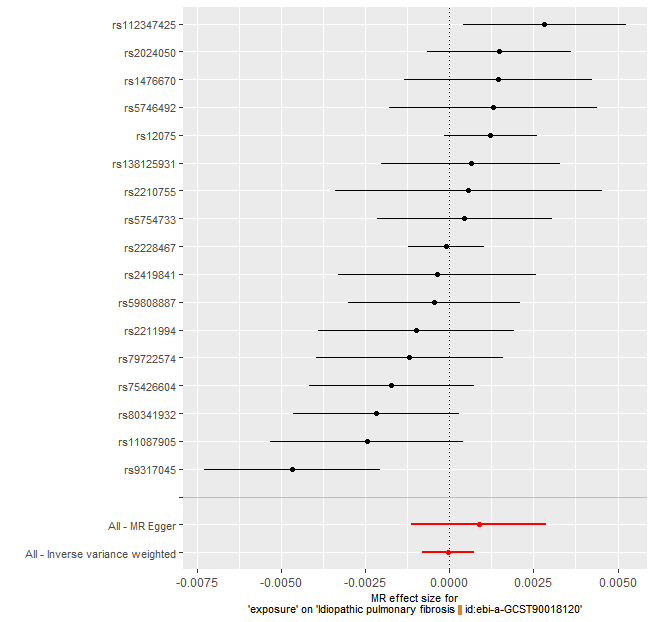
**

**MR leave-one-out sensitivity analysis for **Eotaxin**  on idiopathic pulmonary fibrosis. Circles indicate MR estimates for Eotaxin on idiopathic pulmonary fibrosis using inverse-variance weighted fixed-effect method if each single nucleotide polymorphism was omitted. The bars indicate the CI. MR indicates Mendelian randomization.**

**
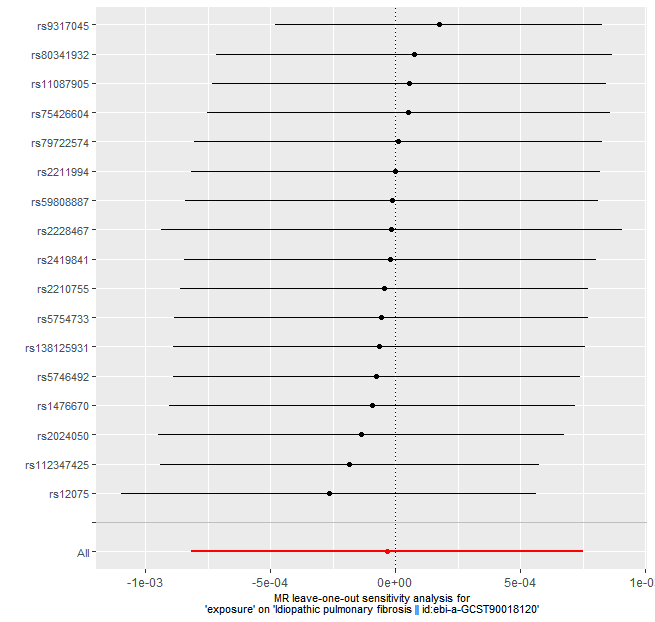
**

**Scatter plot to visualize the causal effect of **GROa** on idiopathic pulmonary fibrosis. The slope of the straight line indicates the magnitude of the causal association. IVW indicates inverse-variance weighted, and MR, Mendelian randomization.**

**
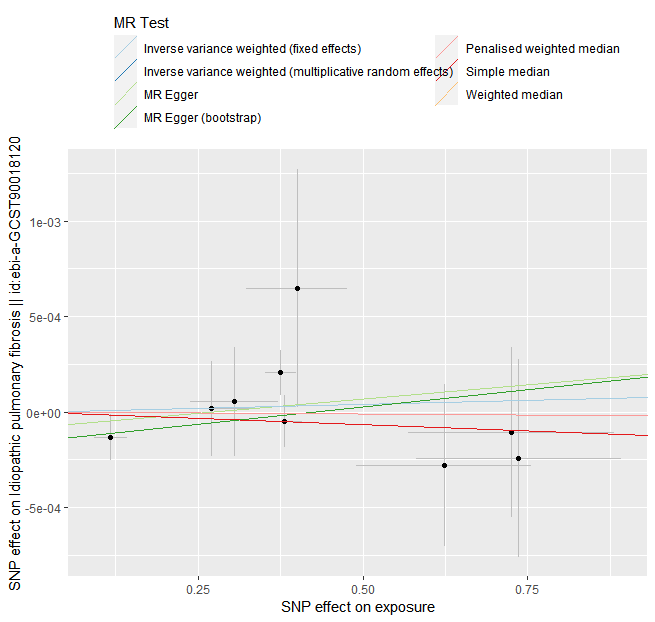
**

**Fixed-effect IVW analysis and of the causal association of **GROa** with idiopathic pulmonary fibrosis. The black dots and bars indicated the causal estimate and 95% CI using each SNP. The red dot and bar indicated the overall estimate and 95% CI meta-analyzed by fixed-effect inverse variance weighted method and MR-Egger method**

**
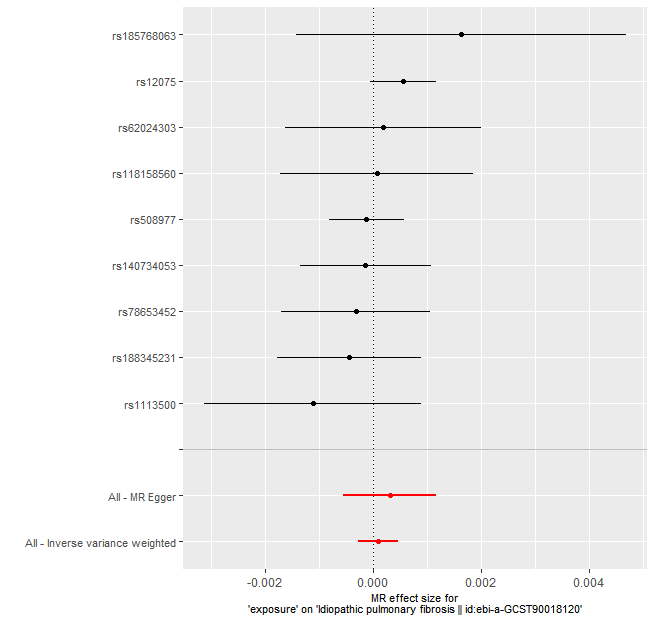
**

**MR leave-one-out sensitivity analysis for **GROa** on idiopathic pulmonary fibrosis. Circles indicate MR estimates for GROa on idiopathic pulmonary fibrosis using inverse-variance weighted fixed-effect method if each single nucleotide polymorphism was omitted. The bars indicate the CI. MR indicates Mendelian randomization.**

**
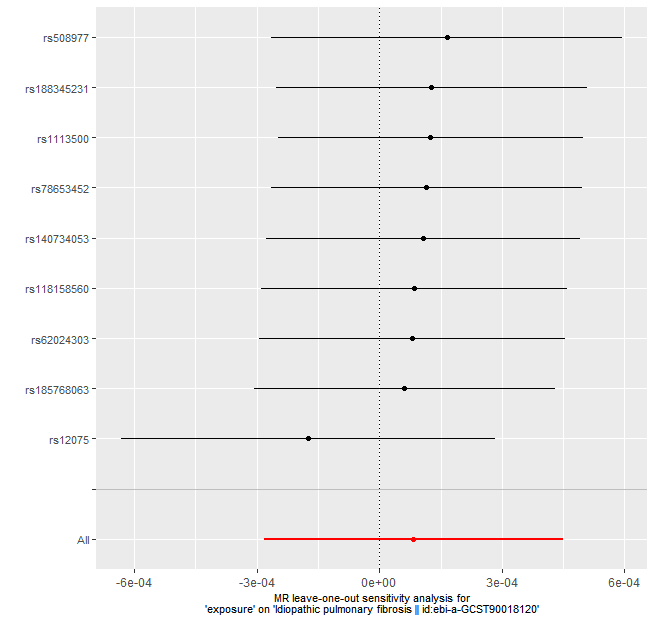
**

**Scatter plot to visualize the causal effect of **MCP1** on idiopathic pulmonary fibrosis. The slope of the straight line indicates the magnitude of the causal association. IVW indicates inverse-variance weighted, and MR, Mendelian randomization.**

**
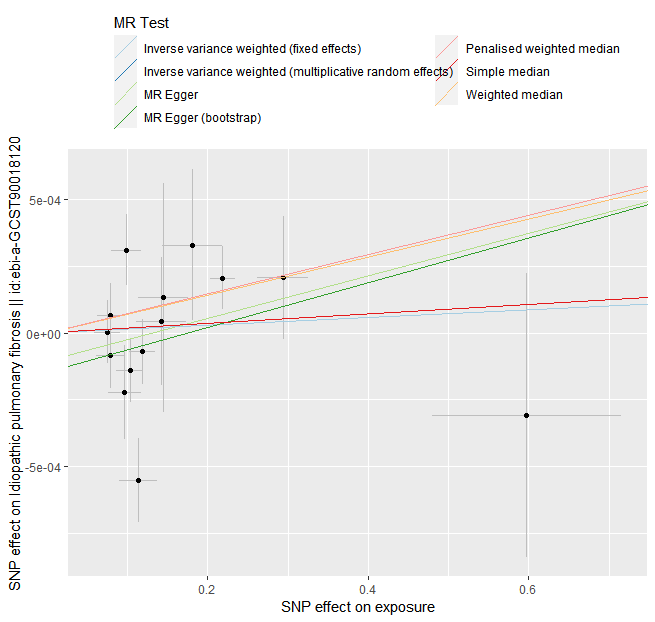
**

**Fixed-effect IVW analysis and of the causal association of **MCP1** with idiopathic pulmonary fibrosis. The black dots and bars indicated the causal estimate and 95% CI using each SNP. The red dot and bar indicated the overall estimate and 95% CI meta-analyzed by fixed-effect inverse variance weighted method and MR-Egger method**

**
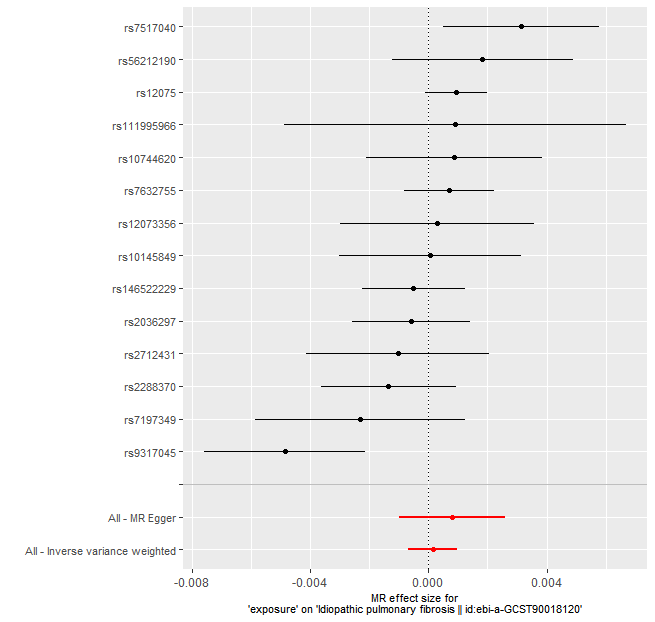
**

**MR leave-one-out sensitivity analysis for **MCP1**  on idiopathic pulmonary fibrosis. Circles indicate MR estimates for MCP1 on idiopathic pulmonary fibrosis using inverse-variance weighted fixed-effect method if each single nucleotide polymorphism was omitted. The bars indicate the CI. MR indicates Mendelian randomization**

**
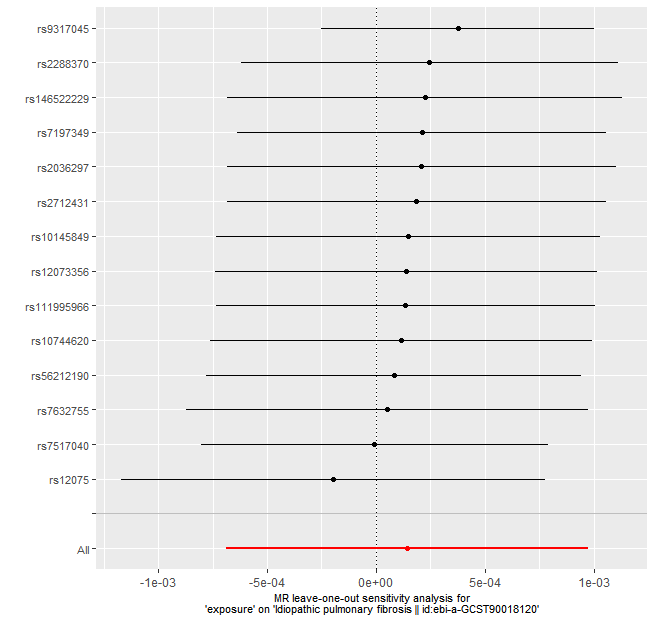
**

**Scatter plot to visualize the causal effect of **MIF** on idiopathic pulmonary fibrosis. The slope of the straight line indicates the magnitude of the causal association. IVW indicates inverse-variance weighted, and MR, Mendelian randomization.**

**
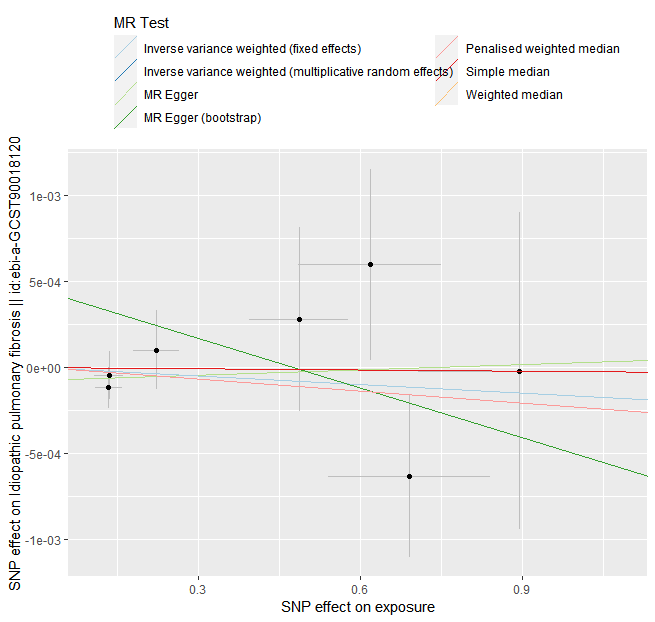
**

**Fixed-effect IVW analysis and of the causal association of **MIF** with idiopathic pulmonary fibrosis. The black dots and bars indicated the causal estimate and 95% CI using each SNP. The red dot and bar indicated the overall estimate and 95% CI meta-analyzed by fixed-effect inverse variance weighted method and MR-Egger method**

**
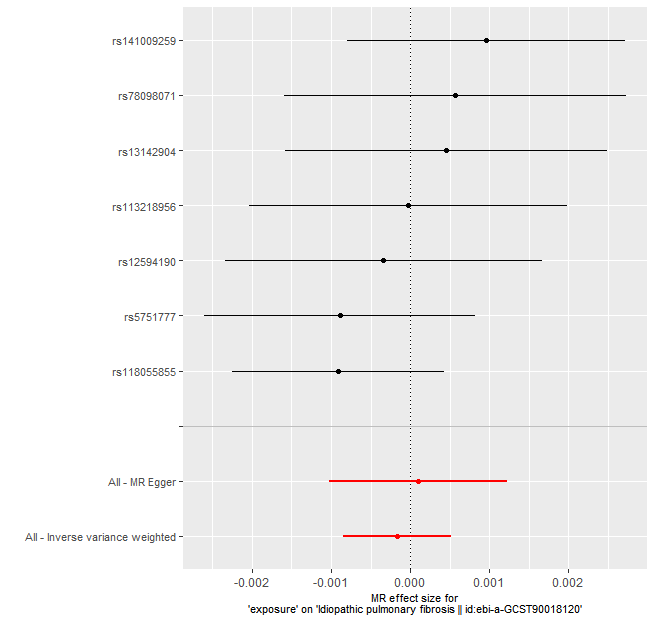
**

**MR leave-one-out sensitivity analysis for **MIF**  on idiopathic pulmonary fibrosis. Circles indicate MR estimates for MIF on idiopathic pulmonary fibrosis using inverse-variance weighted fixed-effect method if each single nucleotide polymorphism was omitted. The bars indicate the CI. MR indicates Mendelian randomization**

**
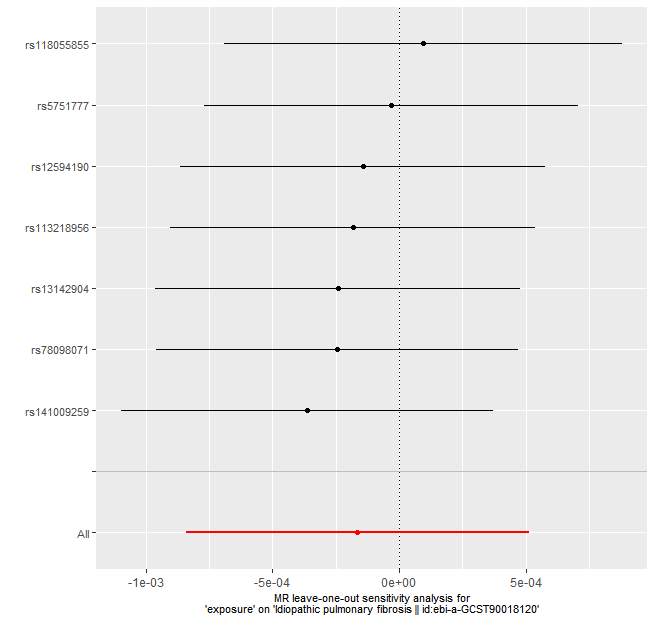
**

**Scatter plot to visualize the causal effect of **MIP1a** on idiopathic pulmonary fibrosis. The slope of the straight line indicates the magnitude of the causal association. IVW indicates inverse-variance weighted, and MR, Mendelian randomization.**

**
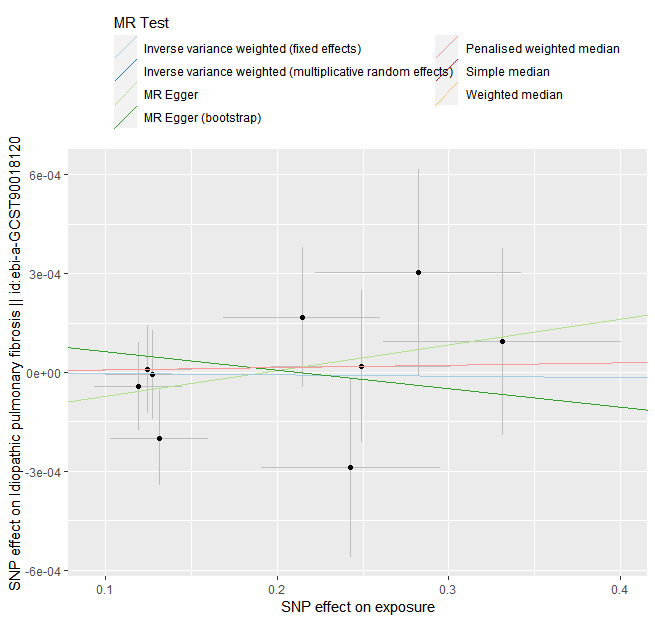
**

**Fixed-effect IVW analysis and of the causal association of **MIP1a** with idiopathic pulmonary fibrosis. The black dots and bars indicated the causal estimate and 95% CI using each SNP. The red dot and bar indicated the overall estimate and 95% CI meta-analyzed by fixed-effect inverse variance weighted method and MR-Egger method**

**
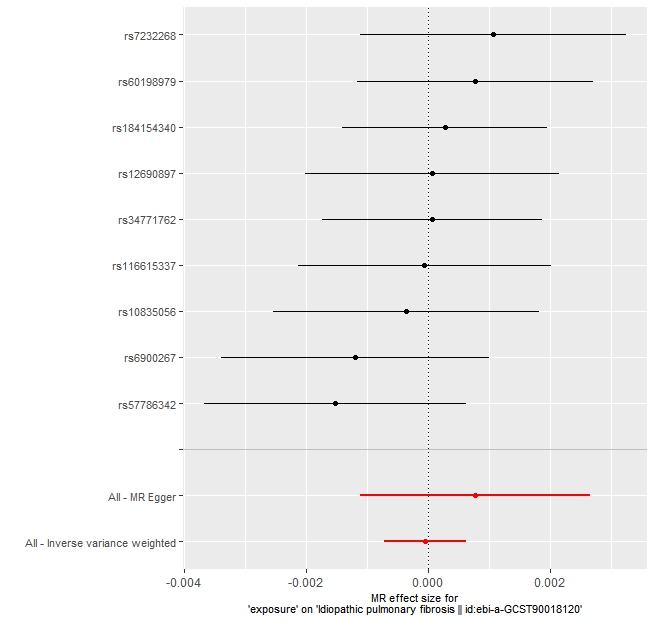
**

**MR leave-one-out sensitivity analysis for **MIP1a**  on idiopathic pulmonary fibrosis. Circles indicate MR estimates for MIP1a on idiopathic pulmonary fibrosis using inverse-variance weighted fixed-effect method if each single nucleotide polymorphism was omitted. The bars indicate the CI. MR indicates Mendelian randomization**

**
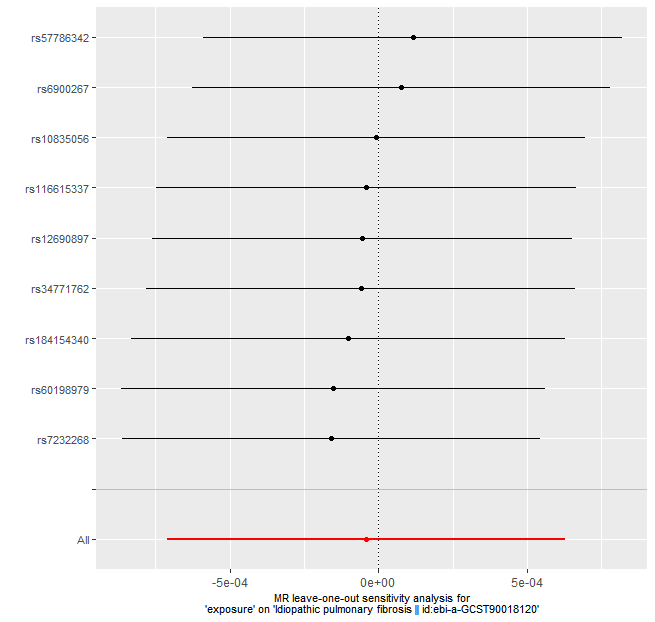
**

**Scatter plot to visualize the causal effect of **MIP1b** on idiopathic pulmonary fibrosis. The slope of the straight line indicates the magnitude of the causal association. IVW indicates inverse-variance weighted, and MR, Mendelian randomization.**

**
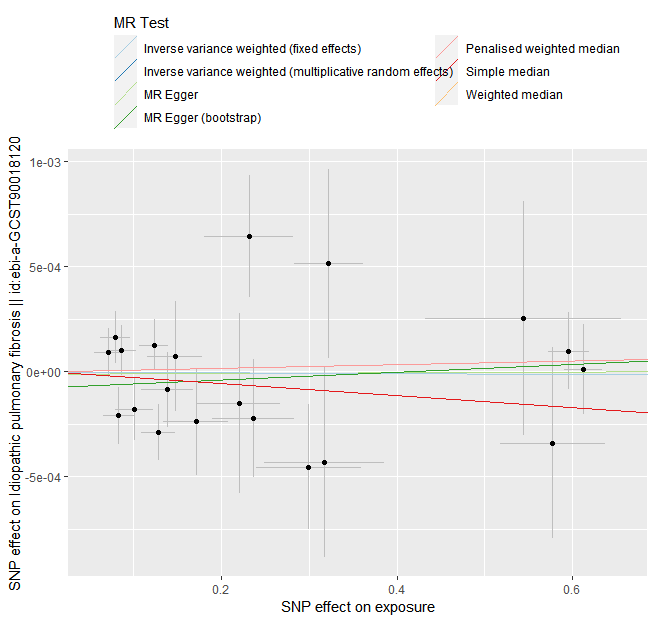
**

**Fixed-effect IVW analysis and of the causal association of **MIP1b** with idiopathic pulmonary fibrosis. The black dots and bars indicated the causal estimate and 95% CI using each SNP. The red dot and bar indicated the overall estimate and 95% CI meta-analyzed by fixed-effect inverse variance weighted method and MR-Egger method**

**
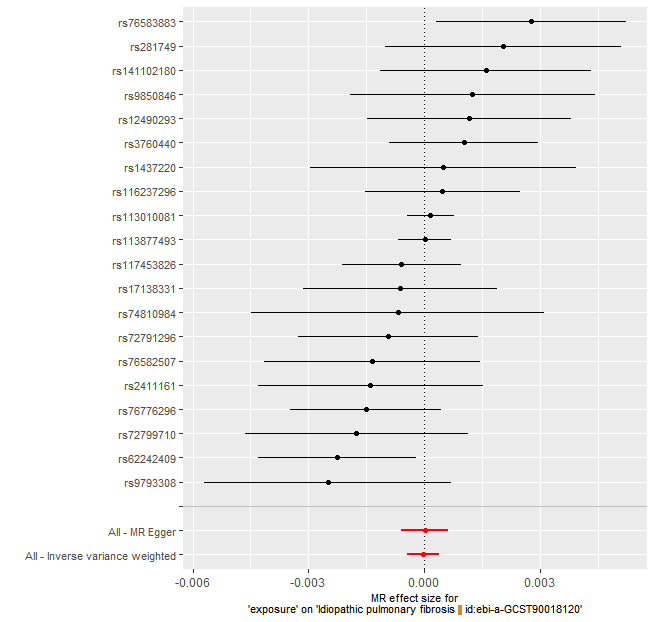
**

**MR leave-one-out sensitivity analysis for **MIP1b**  on idiopathic pulmonary fibrosis. Circles indicate MR estimates for MIP1b on idiopathic pulmonary fibrosis using inverse-variance weighted fixed-effect method if each single nucleotide polymorphism was omitted. The bars indicate the CI. MR indicates Mendelian randomization**

**
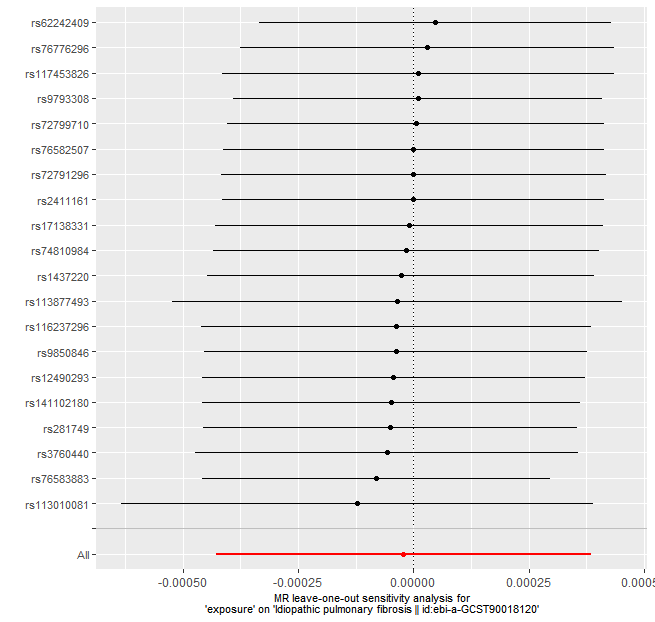
**

**Scatter plot to visualize the causal effect of **RANTES** on idiopathic pulmonary fibrosis. The slope of the straight line indicates the magnitude of the causal association. IVW indicates inverse-variance weighted, and MR, Mendelian randomization.**

**
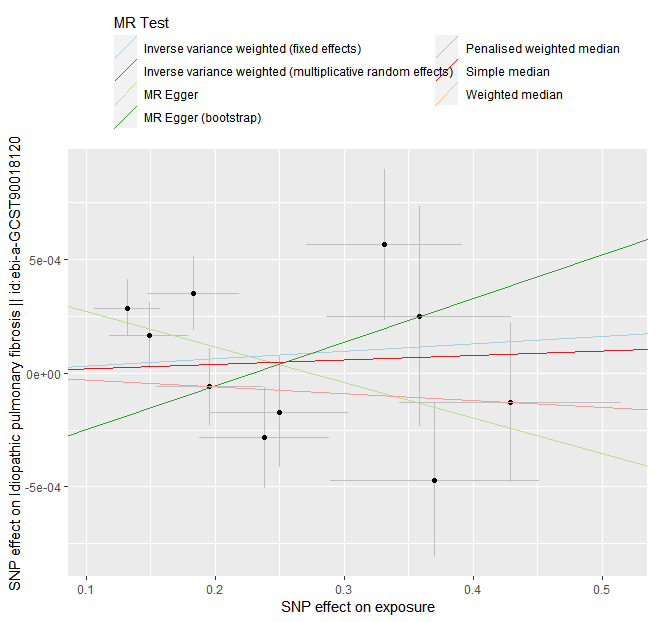
**

**Fixed-effect IVW analysis and of the causal association of **RANTES** with idiopathic pulmonary fibrosis. The black dots and bars indicated the causal estimate and 95% CI using each SNP. The red dot and bar indicated the overall estimate and 95% CI meta-analyzed by fixed-effect inverse variance weighted method and MR-Egger method**

**
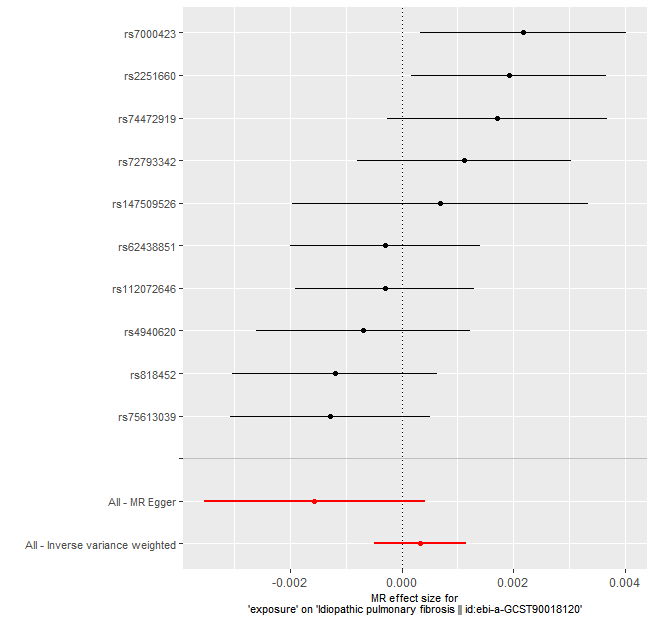
**

**MR leave-one-out sensitivity analysis for **RANTES** on idiopathic pulmonary fibrosis. Circles indicate MR estimates for RANTES on idiopathic pulmonary fibrosis using inverse-variance weighted fixed-effect method if each single nucleotide polymorphism was omitted. The bars indicate the CI. MR indicates Mendelian randomization**

**
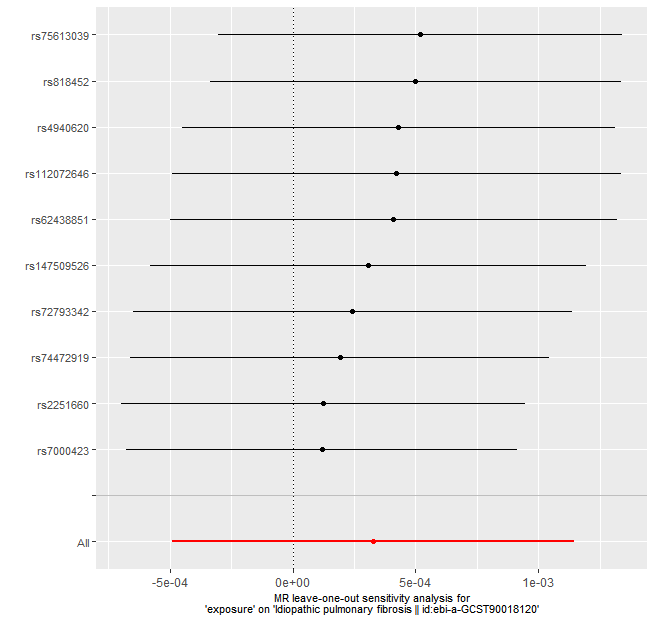
**

**Scatter plot to visualize the causal effect of **TNFa** on idiopathic pulmonary fibrosis. The slope of the straight line indicates the magnitude of the causal association. IVW indicates inverse-variance weighted, and MR, Mendelian randomization.**

**
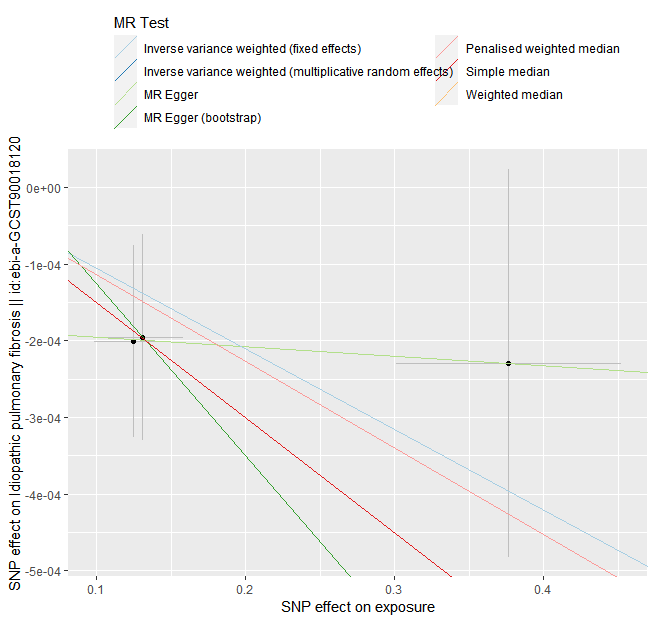
**

**Fixed-effect IVW analysis and of the causal association of **TNFa** with idiopathic pulmonary fibrosis. The black dots and bars indicated the causal estimate and 95% CI using each SNP. The red dot and bar indicated the overall estimate and 95% CI meta-analyzed by fixed-effect inverse variance weighted method and MR-Egger method**

**
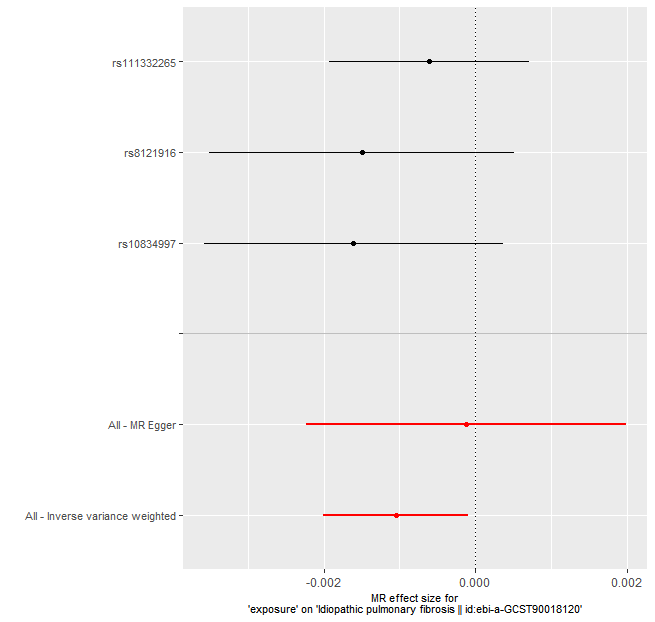
**

**MR leave-one-out sensitivity analysis for **TNFa** on idiopathic pulmonary fibrosis. Circles indicate MR estimates for RANTES on idiopathic pulmonary fibrosis using inverse-variance weighted fixed-effect method if each single nucleotide polymorphism was omitted. The bars indicate the CI. MR indicates Mendelian randomization**

**
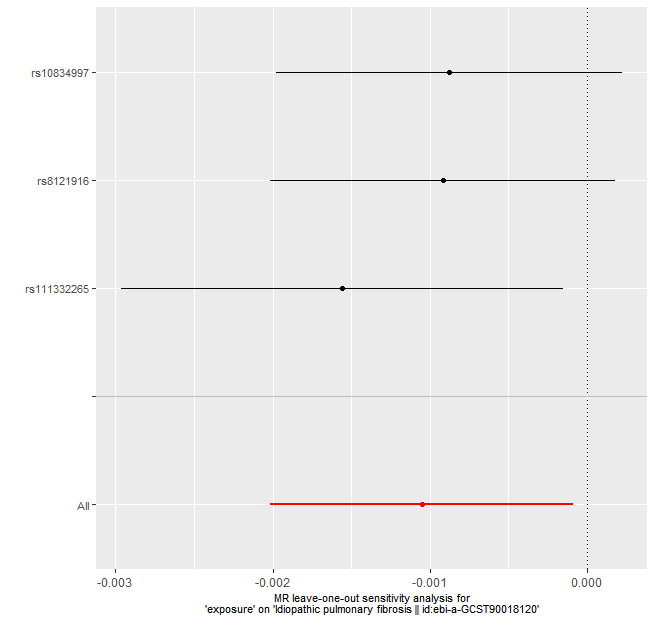
**

**Scatter plot to visualize the causal effect of **TNFb** on idiopathic pulmonary fibrosis. The slope of the straight line indicates the magnitude of the causal association. IVW indicates inverse-variance weighted, and MR, Mendelian randomization.**

**
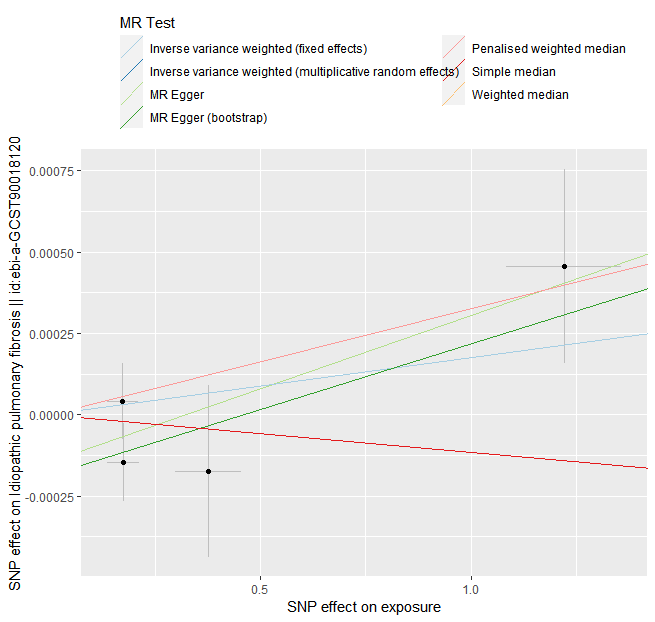
**

**Fixed-effect IVW analysis and of the causal association of **TNFb** with idiopathic pulmonary fibrosis. The black dots and bars indicated the causal estimate and 95% CI using each SNP. The red dot and bar indicated the overall estimate and 95% CI meta-analyzed by fixed-effect inverse variance weighted method and MR-Egger method**

**
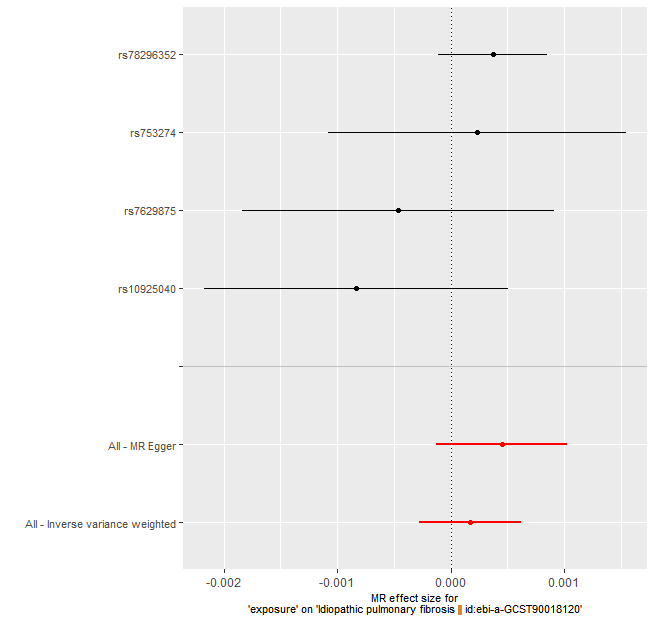
**

**MR leave-one-out sensitivity analysis for **TNFb** on idiopathic pulmonary fibrosis. Circles indicate MR estimates for RANTES on idiopathic pulmonary fibrosis using inverse-variance weighted fixed-effect method if each single nucleotide polymorphism was omitted. The bars indicate the CI. MR indicates Mendelian randomization**

**
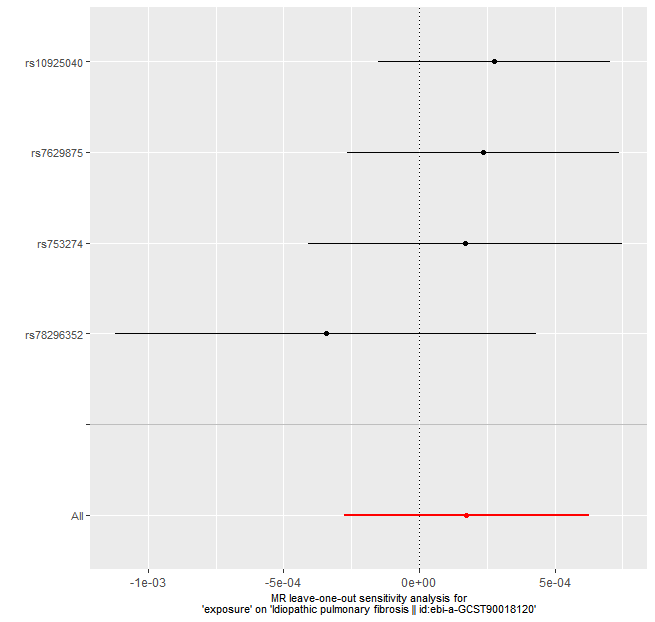
**

**Scatter plot to visualize the causal effect of **TRAIL** on idiopathic pulmonary fibrosis. The slope of the straight line indicates the magnitude of the causal association. IVW indicates inverse-variance weighted, and MR, Mendelian randomization.**

**
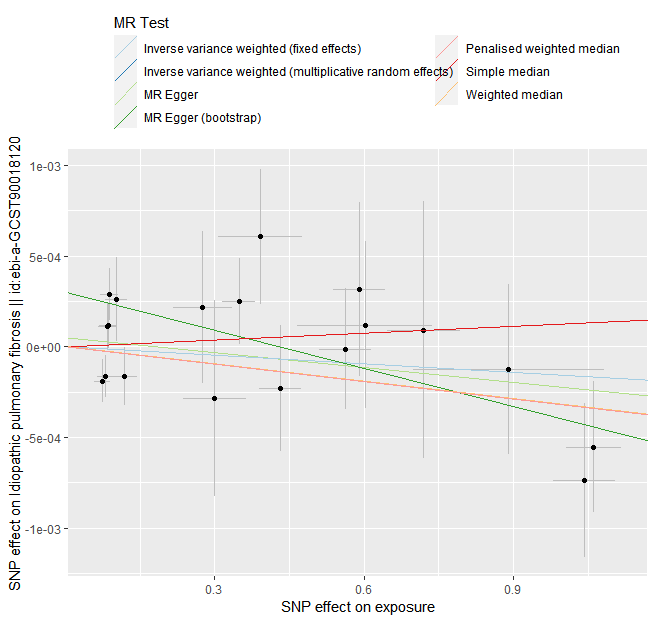
**

**Fixed-effect IVW analysis and of the causal association of **TRAIL** with idiopathic pulmonary fibrosis. The black dots and bars indicated the causal estimate and 95% CI using each SNP. The red dot and bar indicated the overall estimate and 95% CI meta-analyzed by fixed-effect inverse variance weighted method and MR-Egger method**

**
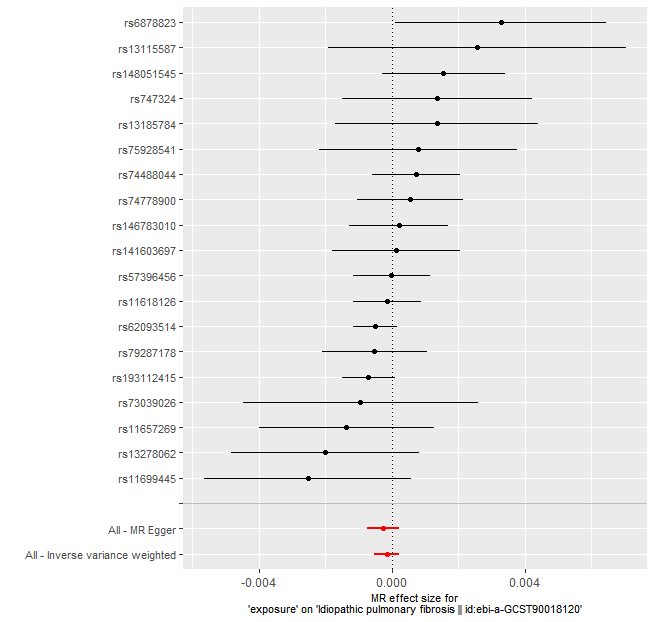
**

**MR leave-one-out sensitivity analysis for **TRAIL** on idiopathic pulmonary fibrosis. Circles indicate MR estimates for RANTES on idiopathic pulmonary fibrosis using inverse-variance weighted fixed-effect method if each single nucleotide polymorphism was omitted. The bars indicate the CI. MR indicates Mendelian randomization**

**
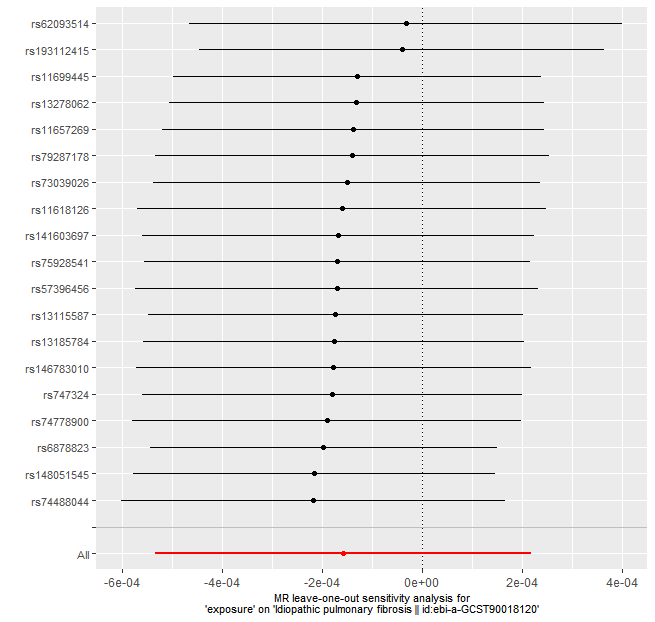
**
