# Supplementary material for: Circulating inflammatory cytokines and risk of idiopathic pulmonary fibrosis: a Mendelian randomization study
Source: BMC Pulm Med. 2023 Oct 3;23:369. doi: 10.1186/s12890-023-02658-3 (PMC10548733; doi:10.1186/s12890-023-02658-3)
Supplement: Supplementary file 2 — Supplementary Material 2 [file 12890_2023_2658_MOESM2_ESM.docx]

**Supplementary Table 1. General characteristics of IVs of circulating inflammatory cytokines and IPF.**

|  |  | SNP | effect_allele.exposure | other_allele.exposure | effect_allele.outcome | other_allele.outcome | beta.exposure | beta.outcome | eaf.exposure | eaf.outcome | remove | palindromic | ambiguous | id.outcome | chr | pos | se.outcome | samplesize.outcome | pval.outcome | outcome | originalname.outcome | outcome.deprecated | mr_keep.outcome | data_source.outcome | se.exposure | pval.exposure | exposure | mr_keep.exposure | pval_origin.exposure | id.exposure | data_source.exposure | action | mr_keep |
| --- | --- | --- | --- | --- | --- | --- | --- | --- | --- | --- | --- | --- | --- | --- | --- | --- | --- | --- | --- | --- | --- | --- | --- | --- | --- | --- | --- | --- | --- | --- | --- | --- | --- |
|  | 1 | rs10903540 | G | A | G | A | 0.1581 | -8.78E-05 | 0.0338 | 0.046093 | FALSE | FALSE | FALSE | ebi-a-GCST90018120 | 10 | 1717507 | 0.000308399 | 437235 | 0.780001 | Idiopathic pulmonary fibrosis \|\| id:ebi-a-GCST90018120 | Idiopathic pulmonary fibrosis | Idiopathic pulmonary fibrosis \|\| \|\| | TRUE | igd | 0.0343 | 4.19E-06 | exposure | TRUE | reported | 3Xa7th | textfile | 2 | TRUE |
|  | 2 | rs12051139 | C | T | C | T | 0.1131 | -8.11E-05 | 0.4006 | 0.409066 | FALSE | FALSE | FALSE | ebi-a-GCST90018120 | 16 | 86918674 | 0.000118344 | 437235 | 0.49 | Idiopathic pulmonary fibrosis \|\| id:ebi-a-GCST90018120 | Idiopathic pulmonary fibrosis | Idiopathic pulmonary fibrosis \|\| \|\| | TRUE | igd | 0.0247 | 4.76E-06 | exposure | TRUE | reported | 3Xa7th | textfile | 2 | TRUE |
|  | 3 | rs13412535 | G | A | G | A | -0.1764 | 6.47E-05 | 0.7744 | 0.76812 | FALSE | FALSE | FALSE | ebi-a-GCST90018120 | 2 | 224874874 | 0.000137289 | 437235 | 0.64 | Idiopathic pulmonary fibrosis \|\| id:ebi-a-GCST90018120 | Idiopathic pulmonary fibrosis | Idiopathic pulmonary fibrosis \|\| \|\| | TRUE | igd | 0.0332 | 1.18E-07 | exposure | TRUE | reported | 3Xa7th | textfile | 2 | TRUE |
|  | 4 | rs1534019 | C | T | C | T | 0.1187 | 5.55E-05 | 0.5557 | 0.563349 | FALSE | FALSE | FALSE | ebi-a-GCST90018120 | 7 | 120879318 | 0.000116893 | 437235 | 0.64 | Idiopathic pulmonary fibrosis \|\| id:ebi-a-GCST90018120 | Idiopathic pulmonary fibrosis | Idiopathic pulmonary fibrosis \|\| \|\| | TRUE | igd | 0.0248 | 1.58E-06 | exposure | TRUE | reported | 3Xa7th | textfile | 2 | TRUE |
|  | 5 | rs170117 | C | T | C | T | 0.1617 | 1.92E-05 | 0.8797 | 0.870555 | FALSE | FALSE | FALSE | ebi-a-GCST90018120 | 4 | 55390380 | 0.000172757 | 437235 | 0.91 | Idiopathic pulmonary fibrosis \|\| id:ebi-a-GCST90018120 | Idiopathic pulmonary fibrosis | Idiopathic pulmonary fibrosis \|\| \|\| | TRUE | igd | 0.0349 | 3.87E-06 | exposure | TRUE | reported | 3Xa7th | textfile | 2 | TRUE |
|  | 6 | rs2807544 | G | A | G | A | -0.1175 | -4.26E-05 | 0.5656 | 0.551724 | FALSE | FALSE | FALSE | ebi-a-GCST90018120 | 1 | 15204245 | 0.000117815 | 437235 | 0.719999 | Idiopathic pulmonary fibrosis \|\| id:ebi-a-GCST90018120 | Idiopathic pulmonary fibrosis | Idiopathic pulmonary fibrosis \|\| \|\| | TRUE | igd | 0.0253 | 3.41E-06 | exposure | TRUE | reported | 3Xa7th | textfile | 2 | TRUE |
|  | 7 | rs4634519 | G | A | G | A | 0.1261 | 5.46E-05 | 0.3121 | 0.284762 | FALSE | FALSE | FALSE | ebi-a-GCST90018120 | 7 | 67192928 | 0.000128251 | 437235 | 0.67 | Idiopathic pulmonary fibrosis \|\| id:ebi-a-GCST90018120 | Idiopathic pulmonary fibrosis | Idiopathic pulmonary fibrosis \|\| \|\| | TRUE | igd | 0.0269 | 2.77E-06 | exposure | TRUE | reported | 3Xa7th | textfile | 2 | TRUE |
|  | 8 | rs61335305 | C | A | C | A | -0.4514 | -5.68E-05 | 0.9891 | 0.982026 | FALSE | FALSE | FALSE | ebi-a-GCST90018120 | 15 | 66453074 | 0.000434857 | 437235 | 0.9 | Idiopathic pulmonary fibrosis \|\| id:ebi-a-GCST90018120 | Idiopathic pulmonary fibrosis | Idiopathic pulmonary fibrosis \|\| \|\| | TRUE | igd | 0.0918 | 7.32E-07 | exposure | TRUE | reported | 3Xa7th | textfile | 2 | TRUE |
|  | 9 | rs62124990 | G | T | G | T | 0.6961 | -0.00030407 | 0.9841 | 0.973775 | FALSE | FALSE | FALSE | ebi-a-GCST90018120 | 2 | 19238636 | 0.000367595 | 437235 | 0.41 | Idiopathic pulmonary fibrosis \|\| id:ebi-a-GCST90018120 | Idiopathic pulmonary fibrosis | Idiopathic pulmonary fibrosis \|\| \|\| | TRUE | igd | 0.1495 | 3.22E-06 | exposure | TRUE | reported | 3Xa7th | textfile | 2 | TRUE |
|  | 10 | rs7615304 | G | A | G | A | 0.1172 | 0.000170172 | 0.6511 | 0.64058 | FALSE | FALSE | FALSE | ebi-a-GCST90018120 | 3 | 156675703 | 0.000120668 | 437235 | 0.16 | Idiopathic pulmonary fibrosis \|\| id:ebi-a-GCST90018120 | Idiopathic pulmonary fibrosis | Idiopathic pulmonary fibrosis \|\| \|\| | TRUE | igd | 0.0242 | 1.21E-06 | exposure | TRUE | reported | 3Xa7th | textfile | 2 | TRUE |
|  | 11 | rs80336398 | C | T | C | T | -0.4001 | -2.71E-05 | 0.0209 | 0.01473 | FALSE | FALSE | FALSE | ebi-a-GCST90018120 | 3 | 64060934 | 0.000504354 | 437235 | 0.96 | Idiopathic pulmonary fibrosis \|\| id:ebi-a-GCST90018120 | Idiopathic pulmonary fibrosis | Idiopathic pulmonary fibrosis \|\| \|\| | TRUE | igd | 0.0858 | 2.82E-06 | exposure | TRUE | reported | 3Xa7th | textfile | 2 | TRUE |
|  |  |  |  |  |  |  |  |  |  |  |  |  |  |  |  |  |  |  |  |  |  |  |  |  |  |  |  |  |  |  |  |  |  |
| IL-6 |  | SNP | effect_allele.exposure | other_allele.exposure | effect_allele.outcome | other_allele.outcome | beta.exposure | beta.outcome | eaf.exposure | eaf.outcome | remove | palindromic | ambiguous | id.outcome | chr | pos | se.outcome | samplesize.outcome | pval.outcome | outcome | originalname.outcome | outcome.deprecated | mr_keep.outcome | data_source.outcome | se.exposure | pval.exposure | exposure | mr_keep.exposure | pval_origin.exposure | id.exposure | data_source.exposure | action | mr_keep |
|  | 1 | rs114373846 | C | T | C | T | -0.422 | 0.000285638 | 0.9871 | 0.984077 | FALSE | FALSE | FALSE | ebi-a-GCST90018120 | 3 | 152566063 | 0.00048399 | 437235 | 0.55 | Idiopathic pulmonary fibrosis \|\| id:ebi-a-GCST90018120 | Idiopathic pulmonary fibrosis | Idiopathic pulmonary fibrosis \|\| \|\| | TRUE | igd | 0.0904 | 3.32E-06 | exposure | TRUE | reported | TXm2eH | textfile | 2 | TRUE |
|  | 2 | rs12024374 | G | A | G | A | -0.1096 | 0.000460229 | 0.8837 | 0.922548 | FALSE | FALSE | FALSE | ebi-a-GCST90018120 | 1 | 234384658 | 0.000216523 | 437235 | 0.0340001 | Idiopathic pulmonary fibrosis \|\| id:ebi-a-GCST90018120 | Idiopathic pulmonary fibrosis | Idiopathic pulmonary fibrosis \|\| \|\| | TRUE | igd | 0.0236 | 3.35E-06 | exposure | TRUE | reported | TXm2eH | textfile | 2 | TRUE |
|  | 3 | rs1333040 | C | T | C | T | -0.0738 | -6.38E-05 | 0.4264 | 0.415419 | FALSE | FALSE | FALSE | ebi-a-GCST90018120 | 9 | 22083404 | 0.000117216 | 437235 | 0.58 | Idiopathic pulmonary fibrosis \|\| id:ebi-a-GCST90018120 | Idiopathic pulmonary fibrosis | Idiopathic pulmonary fibrosis \|\| \|\| | TRUE | igd | 0.0158 | 3.17E-06 | exposure | TRUE | reported | TXm2eH | textfile | 2 | TRUE |
|  | 4 | rs13412535 | G | A | G | A | 0.1164 | 6.47E-05 | 0.7744 | 0.76812 | FALSE | FALSE | FALSE | ebi-a-GCST90018120 | 2 | 224874874 | 0.000137289 | 437235 | 0.64 | Idiopathic pulmonary fibrosis \|\| id:ebi-a-GCST90018120 | Idiopathic pulmonary fibrosis | Idiopathic pulmonary fibrosis \|\| \|\| | TRUE | igd | 0.0215 | 7.34E-08 | exposure | TRUE | reported | TXm2eH | textfile | 2 | TRUE |
|  | 5 | rs72831623 | G | A | G | A | -0.1973 | -0.000254027 | 0.9473 | 0.937332 | FALSE | FALSE | FALSE | ebi-a-GCST90018120 | 17 | 45722293 | 0.000273596 | 437235 | 0.35 | Idiopathic pulmonary fibrosis \|\| id:ebi-a-GCST90018120 | Idiopathic pulmonary fibrosis | Idiopathic pulmonary fibrosis \|\| \|\| | TRUE | igd | 0.0372 | 1.08E-07 | exposure | TRUE | reported | TXm2eH | textfile | 2 | TRUE |
|  | 6 | rs73273528 | C | T | C | T | -0.2672 | 0.000663067 | 0.9662 | 0.966029 | FALSE | FALSE | FALSE | ebi-a-GCST90018120 | 20 | 50431113 | 0.000321351 | 437235 | 0.0389996 | Idiopathic pulmonary fibrosis \|\| id:ebi-a-GCST90018120 | Idiopathic pulmonary fibrosis | Idiopathic pulmonary fibrosis \|\| \|\| | TRUE | igd | 0.0553 | 9.58E-07 | exposure | TRUE | reported | TXm2eH | textfile | 2 | TRUE |
|  | 7 | rs76856708 | C | T | C | T | -0.3289 | 0.000349484 | 0.0378 | 0.038241 | FALSE | FALSE | FALSE | ebi-a-GCST90018120 | 16 | 80729043 | 0.000306839 | 437235 | 0.25 | Idiopathic pulmonary fibrosis \|\| id:ebi-a-GCST90018120 | Idiopathic pulmonary fibrosis | Idiopathic pulmonary fibrosis \|\| \|\| | TRUE | igd | 0.07 | 2.61E-06 | exposure | TRUE | reported | TXm2eH | textfile | 2 | TRUE |
|  |  |  |  |  |  |  |  |  |  |  |  |  |  |  |  |  |  |  |  |  |  |  |  |  |  |  |  |  |  |  |  |  |  |
| IL-8 |  | SNP | effect_allele.exposure | other_allele.exposure | effect_allele.outcome | other_allele.outcome | beta.exposure | beta.outcome | eaf.exposure | eaf.outcome | remove | palindromic | ambiguous | id.outcome | chr | pos | se.outcome | samplesize.outcome | pval.outcome | outcome | originalname.outcome | outcome.deprecated | mr_keep.outcome | data_source.outcome | se.exposure | pval.exposure | exposure | mr_keep.exposure | pval_origin.exposure | id.exposure | data_source.exposure | action | mr_keep |
|  | 1 | rs11634944 | C | T | C | T | 0.1214 | -0.000108118 | 0.3469 | 0.366259 | FALSE | FALSE | FALSE | ebi-a-GCST90018120 | 15 | 25183093 | 0.0001206 | 437235 | 0.37 | Idiopathic pulmonary fibrosis \|\| id:ebi-a-GCST90018120 | Idiopathic pulmonary fibrosis | Idiopathic pulmonary fibrosis \|\| \|\| | TRUE | igd | 0.0252 | 1.29E-06 | exposure | TRUE | reported | 083CZH | textfile | 2 | TRUE |
|  | 2 | rs12075 | G | A | G | A | -0.12 | -0.000204697 | 0.3976 | 0.420187 | FALSE | FALSE | FALSE | ebi-a-GCST90018120 | 1 | 159175354 | 0.000117093 | 437235 | 0.08 | Idiopathic pulmonary fibrosis \|\| id:ebi-a-GCST90018120 | Idiopathic pulmonary fibrosis | Idiopathic pulmonary fibrosis \|\| \|\| | TRUE | igd | 0.0236 | 3.88E-07 | exposure | TRUE | reported | 083CZH | textfile | 2 | TRUE |
|  | 3 | rs141926526 | C | A | C | A | 0.6149 | -0.000260737 | 0.0477 | 0.039601 | FALSE | FALSE | FALSE | ebi-a-GCST90018120 | 7 | 32848640 | 0.000297601 | 437235 | 0.38 | Idiopathic pulmonary fibrosis \|\| id:ebi-a-GCST90018120 | Idiopathic pulmonary fibrosis | Idiopathic pulmonary fibrosis \|\| \|\| | TRUE | igd | 0.1308 | 2.57E-06 | exposure | TRUE | reported | 083CZH | textfile | 2 | TRUE |
|  | 4 | rs2673604 | C | A | C | A | 0.1266 | -1.29E-05 | 0.2992 | 0.281278 | FALSE | FALSE | FALSE | ebi-a-GCST90018120 | 8 | 133411607 | 0.000128645 | 437235 | 0.92 | Idiopathic pulmonary fibrosis \|\| id:ebi-a-GCST90018120 | Idiopathic pulmonary fibrosis | Idiopathic pulmonary fibrosis \|\| \|\| | TRUE | igd | 0.0255 | 7.02E-07 | exposure | TRUE | reported | 083CZH | textfile | 2 | TRUE |
|  |  |  |  |  |  |  |  |  |  |  |  |  |  |  |  |  |  |  |  |  |  |  |  |  |  |  |  |  |  |  |  |  |  |
| IL-10 |  | SNP | effect_allele.exposure | other_allele.exposure | effect_allele.outcome | other_allele.outcome | beta.exposure | beta.outcome | eaf.exposure | eaf.outcome | remove | palindromic | ambiguous | id.outcome | chr | pos | se.outcome | samplesize.outcome | pval.outcome | outcome | originalname.outcome | outcome.deprecated | mr_keep.outcome | data_source.outcome | se.exposure | pval.exposure | exposure | mr_keep.exposure | pval_origin.exposure | id.exposure | data_source.exposure | action | mr_keep |
|  | 1 | rs10457128 | G | A | G | A | 0.0865 | -0.000224881 | 0.3608 | 0.372245 | FALSE | FALSE | FALSE | ebi-a-GCST90018120 | 6 | 106017976 | 0.000120477 | 437235 | 0.0619998 | Idiopathic pulmonary fibrosis \|\| id:ebi-a-GCST90018120 | Idiopathic pulmonary fibrosis | Idiopathic pulmonary fibrosis \|\| \|\| | TRUE | igd | 0.0172 | 5.24E-07 | exposure | TRUE | reported | 8LkfUb | textfile | 2 | TRUE |
|  | 2 | rs10493718 | C | A | C | A | 0.11 | 0.000174905 | 0.7087 | 0.775808 | FALSE | FALSE | FALSE | ebi-a-GCST90018120 | 1 | 83062933 | 0.000138522 | 437235 | 0.21 | Idiopathic pulmonary fibrosis \|\| id:ebi-a-GCST90018120 | Idiopathic pulmonary fibrosis | Idiopathic pulmonary fibrosis \|\| \|\| | TRUE | igd | 0.0222 | 7.16E-07 | exposure | TRUE | reported | 8LkfUb | textfile | 2 | TRUE |
|  | 3 | rs10809307 | C | T | C | T | -0.1305 | 0.000119339 | 0.6958 | 0.688723 | FALSE | FALSE | FALSE | ebi-a-GCST90018120 | 9 | 11045908 | 0.000125586 | 437235 | 0.34 | Idiopathic pulmonary fibrosis \|\| id:ebi-a-GCST90018120 | Idiopathic pulmonary fibrosis | Idiopathic pulmonary fibrosis \|\| \|\| | TRUE | igd | 0.0282 | 3.64E-06 | exposure | TRUE | reported | 8LkfUb | textfile | 2 | TRUE |
|  | 4 | rs11206302 | C | T | C | T | 0.1189 | -0.000170849 | 0.0795 | 0.078031 | FALSE | FALSE | FALSE | ebi-a-GCST90018120 | 1 | 54673943 | 0.000215847 | 437235 | 0.43 | Idiopathic pulmonary fibrosis \|\| id:ebi-a-GCST90018120 | Idiopathic pulmonary fibrosis | Idiopathic pulmonary fibrosis \|\| \|\| | TRUE | igd | 0.0251 | 2.20E-06 | exposure | TRUE | reported | 8LkfUb | textfile | 2 | TRUE |
|  | 5 | rs113831257 | G | A | G | A | -0.3592 | 0.000471911 | 0.9583 | 0.957776 | FALSE | FALSE | FALSE | ebi-a-GCST90018120 | 4 | 76159521 | 0.000297154 | 437235 | 0.11 | Idiopathic pulmonary fibrosis \|\| id:ebi-a-GCST90018120 | Idiopathic pulmonary fibrosis | Idiopathic pulmonary fibrosis \|\| \|\| | TRUE | igd | 0.0644 | 2.53E-08 | exposure | TRUE | reported | 8LkfUb | textfile | 2 | TRUE |
|  | 6 | rs11626201 | C | A | C | A | -0.1162 | 3.03E-05 | 0.3658 | 0.407556 | FALSE | FALSE | FALSE | ebi-a-GCST90018120 | 14 | 36980700 | 0.000118515 | 437235 | 0.8 | Idiopathic pulmonary fibrosis \|\| id:ebi-a-GCST90018120 | Idiopathic pulmonary fibrosis | Idiopathic pulmonary fibrosis \|\| \|\| | TRUE | igd | 0.0245 | 1.93E-06 | exposure | TRUE | reported | 8LkfUb | textfile | 2 | TRUE |
|  | 7 | rs143799975 | G | A | G | A | 0.7984 | -0.000447534 | 0.0129 | 0.011923 | FALSE | FALSE | FALSE | ebi-a-GCST90018120 | 4 | 76807015 | 0.000533453 | 437235 | 0.4 | Idiopathic pulmonary fibrosis \|\| id:ebi-a-GCST90018120 | Idiopathic pulmonary fibrosis | Idiopathic pulmonary fibrosis \|\| \|\| | TRUE | igd | 0.1637 | 1.00E-06 | exposure | TRUE | reported | 8LkfUb | textfile | 2 | TRUE |
|  | 8 | rs181236764 | C | T | C | T | -0.4504 | 9.35E-05 | 0.9573 | 0.963252 | FALSE | FALSE | FALSE | ebi-a-GCST90018120 | 5 | 73103249 | 0.000309177 | 437235 | 0.760001 | Idiopathic pulmonary fibrosis \|\| id:ebi-a-GCST90018120 | Idiopathic pulmonary fibrosis | Idiopathic pulmonary fibrosis \|\| \|\| | TRUE | igd | 0.0964 | 3.22E-06 | exposure | TRUE | reported | 8LkfUb | textfile | 2 | TRUE |
|  | 9 | rs2086656 | C | T | C | T | 0.0789 | 6.46E-05 | 0.3052 | 0.295892 | FALSE | FALSE | FALSE | ebi-a-GCST90018120 | 4 | 60498473 | 0.000126895 | 437235 | 0.61 | Idiopathic pulmonary fibrosis \|\| id:ebi-a-GCST90018120 | Idiopathic pulmonary fibrosis | Idiopathic pulmonary fibrosis \|\| \|\| | TRUE | igd | 0.0171 | 3.78E-06 | exposure | TRUE | reported | 8LkfUb | textfile | 2 | TRUE |
|  | 10 | rs282258 | C | T | C | T | -0.0992 | -8.17E-05 | 0.5746 | 0.56974 | FALSE | FALSE | FALSE | ebi-a-GCST90018120 | 2 | 224914800 | 0.000117197 | 437235 | 0.48 | Idiopathic pulmonary fibrosis \|\| id:ebi-a-GCST90018120 | Idiopathic pulmonary fibrosis | Idiopathic pulmonary fibrosis \|\| \|\| | TRUE | igd | 0.0162 | 1.00E-09 | exposure | TRUE | reported | 8LkfUb | textfile | 2 | TRUE |
|  | 11 | rs3025021 | C | T | C | T | -0.0947 | 1.21E-05 | 0.6322 | 0.666371 | FALSE | FALSE | FALSE | ebi-a-GCST90018120 | 6 | 43749163 | 0.000126117 | 437235 | 0.93 | Idiopathic pulmonary fibrosis \|\| id:ebi-a-GCST90018120 | Idiopathic pulmonary fibrosis | Idiopathic pulmonary fibrosis \|\| \|\| | TRUE | igd | 0.0195 | 1.46E-06 | exposure | TRUE | reported | 8LkfUb | textfile | 2 | TRUE |
|  | 12 | rs34383175 | C | T | C | T | 0.3153 | 4.29E-05 | 0.9732 | 0.964237 | FALSE | FALSE | FALSE | ebi-a-GCST90018120 | 8 | 145584694 | 0.000311423 | 437235 | 0.89 | Idiopathic pulmonary fibrosis \|\| id:ebi-a-GCST90018120 | Idiopathic pulmonary fibrosis | Idiopathic pulmonary fibrosis \|\| \|\| | TRUE | igd | 0.0657 | 1.51E-06 | exposure | TRUE | reported | 8LkfUb | textfile | 2 | TRUE |
|  | 13 | rs35794877 | C | A | C | A | -0.0794 | 0.00028497 | 0.508 | 0.485 | FALSE | FALSE | FALSE | ebi-a-GCST90018120 | 4 | 7708698 | 0.000116102 | 437235 | 0.0140001 | Idiopathic pulmonary fibrosis \|\| id:ebi-a-GCST90018120 | Idiopathic pulmonary fibrosis | Idiopathic pulmonary fibrosis \|\| \|\| | TRUE | igd | 0.0167 | 1.80E-06 | exposure | TRUE | reported | 8LkfUb | textfile | 2 | TRUE |
|  | 14 | rs397816 | C | T | C | T | -0.1237 | 0.00017904 | 0.4264 | 0.444563 | FALSE | FALSE | FALSE | ebi-a-GCST90018120 | 22 | 22728326 | 0.000117076 | 437235 | 0.13 | Idiopathic pulmonary fibrosis \|\| id:ebi-a-GCST90018120 | Idiopathic pulmonary fibrosis | Idiopathic pulmonary fibrosis \|\| \|\| | TRUE | igd | 0.0249 | 7.90E-07 | exposure | TRUE | reported | 8LkfUb | textfile | 2 | TRUE |
|  | 15 | rs41282660 | G | A | G | A | 0.1194 | 0.000311228 | 0.1322 | 0.116065 | FALSE | FALSE | FALSE | ebi-a-GCST90018120 | 6 | 44197006 | 0.000180396 | 437235 | 0.0840001 | Idiopathic pulmonary fibrosis \|\| id:ebi-a-GCST90018120 | Idiopathic pulmonary fibrosis | Idiopathic pulmonary fibrosis \|\| \|\| | TRUE | igd | 0.0255 | 3.72E-06 | exposure | TRUE | reported | 8LkfUb | textfile | 2 | TRUE |
|  | 16 | rs4349809 | G | T | G | T | -0.2853 | 6.33E-05 | 0.4513 | 0.478674 | FALSE | FALSE | FALSE | ebi-a-GCST90018120 | 6 | 43924830 | 0.000116344 | 437235 | 0.59 | Idiopathic pulmonary fibrosis \|\| id:ebi-a-GCST90018120 | Idiopathic pulmonary fibrosis | Idiopathic pulmonary fibrosis \|\| \|\| | TRUE | igd | 0.0165 | 5.77E-67 | exposure | TRUE | reported | 8LkfUb | textfile | 2 | TRUE |
|  | 17 | rs465757 | G | A | G | A | -0.084 | -0.000176631 | 0.333 | 0.317127 | FALSE | FALSE | FALSE | ebi-a-GCST90018120 | 20 | 15580283 | 0.000125697 | 437235 | 0.16 | Idiopathic pulmonary fibrosis \|\| id:ebi-a-GCST90018120 | Idiopathic pulmonary fibrosis | Idiopathic pulmonary fibrosis \|\| \|\| | TRUE | igd | 0.0174 | 1.17E-06 | exposure | TRUE | reported | 8LkfUb | textfile | 2 | TRUE |
|  | 18 | rs4741748 | G | A | G | A | 0.0793 | -4.57E-05 | 0.4284 | 0.444436 | FALSE | FALSE | FALSE | ebi-a-GCST90018120 | 9 | 2626851 | 0.000116965 | 437235 | 0.69 | Idiopathic pulmonary fibrosis \|\| id:ebi-a-GCST90018120 | Idiopathic pulmonary fibrosis | Idiopathic pulmonary fibrosis \|\| \|\| | TRUE | igd | 0.017 | 2.79E-06 | exposure | TRUE | reported | 8LkfUb | textfile | 2 | TRUE |
|  | 19 | rs7088799 | G | T | G | T | 0.0852 | 0.000135379 | 0.4344 | 0.416531 | FALSE | FALSE | FALSE | ebi-a-GCST90018120 | 10 | 65016174 | 0.000117436 | 437235 | 0.25 | Idiopathic pulmonary fibrosis \|\| id:ebi-a-GCST90018120 | Idiopathic pulmonary fibrosis | Idiopathic pulmonary fibrosis \|\| \|\| | TRUE | igd | 0.0167 | 3.23E-07 | exposure | TRUE | reported | 8LkfUb | textfile | 2 | TRUE |
|  | 20 | rs75970138 | G | A | G | A | 0.485 | -0.000679849 | 0.9871 | 0.991931 | FALSE | FALSE | FALSE | ebi-a-GCST90018120 | 9 | 122576276 | 0.000646025 | 437235 | 0.29 | Idiopathic pulmonary fibrosis \|\| id:ebi-a-GCST90018120 | Idiopathic pulmonary fibrosis | Idiopathic pulmonary fibrosis \|\| \|\| | TRUE | igd | 0.104 | 1.53E-06 | exposure | TRUE | reported | 8LkfUb | textfile | 2 | TRUE |
|  | 21 | rs7645625 | G | T | G | T | 0.1086 | 2.02E-05 | 0.4324 | 0.431601 | FALSE | FALSE | FALSE | ebi-a-GCST90018120 | 3 | 146574037 | 0.000117299 | 437235 | 0.86 | Idiopathic pulmonary fibrosis \|\| id:ebi-a-GCST90018120 | Idiopathic pulmonary fibrosis | Idiopathic pulmonary fibrosis \|\| \|\| | TRUE | igd | 0.0237 | 4.41E-06 | exposure | TRUE | reported | 8LkfUb | textfile | 2 | TRUE |
|  | 22 | rs79848609 | C | A | C | A | -0.2603 | -0.000397444 | 0.0318 | 0.048192 | FALSE | FALSE | FALSE | ebi-a-GCST90018120 | 15 | 87316165 | 0.000277815 | 437235 | 0.15 | Idiopathic pulmonary fibrosis \|\| id:ebi-a-GCST90018120 | Idiopathic pulmonary fibrosis | Idiopathic pulmonary fibrosis \|\| \|\| | TRUE | igd | 0.0537 | 8.75E-07 | exposure | TRUE | reported | 8LkfUb | textfile | 2 | TRUE |
|  | 23 | rs8112909 | G | A | G | A | 0.1426 | 0.000157482 | 0.1918 | 0.202265 | FALSE | FALSE | FALSE | ebi-a-GCST90018120 | 19 | 46413408 | 0.000144255 | 437235 | 0.27 | Idiopathic pulmonary fibrosis \|\| id:ebi-a-GCST90018120 | Idiopathic pulmonary fibrosis | Idiopathic pulmonary fibrosis \|\| \|\| | TRUE | igd | 0.0299 | 1.94E-06 | exposure | TRUE | reported | 8LkfUb | textfile | 2 | TRUE |
|  | 24 | rs9450351 | C | T | C | T | 0.2768 | 0.000246804 | 0.0616 | 0.073059 | FALSE | FALSE | FALSE | ebi-a-GCST90018120 | 6 | 86624320 | 0.000236266 | 437235 | 0.3 | Idiopathic pulmonary fibrosis \|\| id:ebi-a-GCST90018120 | Idiopathic pulmonary fibrosis | Idiopathic pulmonary fibrosis \|\| \|\| | TRUE | igd | 0.0489 | 1.48E-08 | exposure | TRUE | reported | 8LkfUb | textfile | 2 | TRUE |
|  |  |  |  |  |  |  |  |  |  |  |  |  |  |  |  |  |  |  |  |  |  |  |  |  |  |  |  |  |  |  |  |  |  |
| IL-13 |  | SNP | effect_allele.exposure | other_allele.exposure | effect_allele.outcome | other_allele.outcome | beta.exposure | beta.outcome | eaf.exposure | eaf.outcome | remove | palindromic | ambiguous | id.outcome | chr | pos | se.outcome | samplesize.outcome | pval.outcome | outcome | originalname.outcome | outcome.deprecated | mr_keep.outcome | data_source.outcome | se.exposure | pval.exposure | exposure | mr_keep.exposure | pval_origin.exposure | id.exposure | data_source.exposure | action | mr_keep |
|  | 1 | rs117795020 | G | A | G | A | 0.3522 | -0.000277853 | 0.9821 | 0.981505 | FALSE | FALSE | FALSE | ebi-a-GCST90018120 | 9 | 90084152 | 0.000429303 | 437235 | 0.52 | Idiopathic pulmonary fibrosis \|\| id:ebi-a-GCST90018120 | Idiopathic pulmonary fibrosis | Idiopathic pulmonary fibrosis \|\| \|\| | TRUE | igd | 0.0716 | 9.86E-07 | exposure | TRUE | reported | j1b8JP | textfile | 2 | TRUE |
|  | 2 | rs12623722 | G | A | G | A | 0.1185 | -0.000173645 | 0.7177 | 0.705233 | FALSE | FALSE | FALSE | ebi-a-GCST90018120 | 2 | 23178683 | 0.000127164 | 437235 | 0.17 | Idiopathic pulmonary fibrosis \|\| id:ebi-a-GCST90018120 | Idiopathic pulmonary fibrosis | Idiopathic pulmonary fibrosis \|\| \|\| | TRUE | igd | 0.0258 | 4.19E-06 | exposure | TRUE | reported | j1b8JP | textfile | 2 | TRUE |
|  | 3 | rs139083458 | C | T | C | T | -0.9902 | 0.000568763 | 0.9841 | 0.98213 | FALSE | FALSE | FALSE | ebi-a-GCST90018120 | 5 | 26160518 | 0.000442517 | 437235 | 0.2 | Idiopathic pulmonary fibrosis \|\| id:ebi-a-GCST90018120 | Idiopathic pulmonary fibrosis | Idiopathic pulmonary fibrosis \|\| \|\| | TRUE | igd | 0.2107 | 2.81E-06 | exposure | TRUE | reported | j1b8JP | textfile | 2 | TRUE |
|  | 4 | rs142167313 | C | T | C | T | 0.313 | -0.00148768 | 0.0189 | 0.010191 | FALSE | FALSE | FALSE | ebi-a-GCST90018120 | 6 | 44172097 | 0.000601918 | 437235 | 0.0140001 | Idiopathic pulmonary fibrosis \|\| id:ebi-a-GCST90018120 | Idiopathic pulmonary fibrosis | Idiopathic pulmonary fibrosis \|\| \|\| | TRUE | igd | 0.0617 | 3.98E-07 | exposure | TRUE | reported | j1b8JP | textfile | 2 | TRUE |
|  | 5 | rs27949 | C | T | C | T | 0.1168 | -8.48E-05 | 0.3688 | 0.317661 | FALSE | FALSE | FALSE | ebi-a-GCST90018120 | 5 | 58550823 | 0.000124502 | 437235 | 0.5 | Idiopathic pulmonary fibrosis \|\| id:ebi-a-GCST90018120 | Idiopathic pulmonary fibrosis | Idiopathic pulmonary fibrosis \|\| \|\| | TRUE | igd | 0.0252 | 3.43E-06 | exposure | TRUE | reported | j1b8JP | textfile | 2 | TRUE |
|  | 6 | rs6799107 | C | T | C | T | 0.1459 | -6.47E-05 | 0.2137 | 0.207956 | FALSE | FALSE | FALSE | ebi-a-GCST90018120 | 3 | 127057018 | 0.000142407 | 437235 | 0.649999 | Idiopathic pulmonary fibrosis \|\| id:ebi-a-GCST90018120 | Idiopathic pulmonary fibrosis | Idiopathic pulmonary fibrosis \|\| \|\| | TRUE | igd | 0.0301 | 1.25E-06 | exposure | TRUE | reported | j1b8JP | textfile | 2 | TRUE |
|  | 7 | rs7073807 | C | T | C | T | -0.1682 | 0.000321374 | 0.8827 | 0.887815 | FALSE | FALSE | FALSE | ebi-a-GCST90018120 | 10 | 69153428 | 0.000184513 | 437235 | 0.0810009 | Idiopathic pulmonary fibrosis \|\| id:ebi-a-GCST90018120 | Idiopathic pulmonary fibrosis | Idiopathic pulmonary fibrosis \|\| \|\| | TRUE | igd | 0.0356 | 2.37E-06 | exposure | TRUE | reported | j1b8JP | textfile | 2 | TRUE |
|  | 8 | rs75995699 | G | A | G | A | -0.3319 | -0.000334957 | 0.9761 | 0.975133 | FALSE | FALSE | FALSE | ebi-a-GCST90018120 | 6 | 5140856 | 0.000375225 | 437235 | 0.37 | Idiopathic pulmonary fibrosis \|\| id:ebi-a-GCST90018120 | Idiopathic pulmonary fibrosis | Idiopathic pulmonary fibrosis \|\| \|\| | TRUE | igd | 0.0698 | 2.64E-06 | exposure | TRUE | reported | j1b8JP | textfile | 2 | TRUE |
|  | 9 | rs9472168 | G | A | G | A | -0.4244 | 5.31E-05 | 0.4493 | 0.478933 | FALSE | FALSE | FALSE | ebi-a-GCST90018120 | 6 | 43928985 | 0.000117577 | 437235 | 0.649999 | Idiopathic pulmonary fibrosis \|\| id:ebi-a-GCST90018120 | Idiopathic pulmonary fibrosis | Idiopathic pulmonary fibrosis \|\| \|\| | TRUE | igd | 0.0248 | 1.08E-65 | exposure | TRUE | reported | j1b8JP | textfile | 2 | TRUE |
|  |  |  |  |  |  |  |  |  |  |  |  |  |  |  |  |  |  |  |  |  |  |  |  |  |  |  |  |  |  |  |  |  |  |
| IL-14 |  | SNP | effect_allele.exposure | other_allele.exposure | effect_allele.outcome | other_allele.outcome | beta.exposure | beta.outcome | eaf.exposure | eaf.outcome | remove | palindromic | ambiguous | id.outcome | chr | pos | se.outcome | samplesize.outcome | pval.outcome | outcome | originalname.outcome | outcome.deprecated | mr_keep.outcome | data_source.outcome | se.exposure | pval.exposure | exposure | mr_keep.exposure | pval_origin.exposure | id.exposure | data_source.exposure | action | mr_keep |
|  | 1 | rs10512267 | C | T | C | T | 0.0824 | 0.000221829 | 0.33 | 0.332301 | FALSE | FALSE | FALSE | ebi-a-GCST90018120 | 9 | 102190129 | 0.00012338 | 437235 | 0.0719996 | Idiopathic pulmonary fibrosis \|\| id:ebi-a-GCST90018120 | Idiopathic pulmonary fibrosis | Idiopathic pulmonary fibrosis \|\| \|\| | TRUE | igd | 0.0161 | 2.94E-07 | exposure | TRUE | reported | TJb989 | textfile | 2 | TRUE |
|  | 2 | rs116705532 | G | T | G | T | 0.4678 | 0.000808914 | 0.0169 | 0.017066 | FALSE | FALSE | FALSE | ebi-a-GCST90018120 | 1 | 113705169 | 0.000454408 | 437235 | 0.0749998 | Idiopathic pulmonary fibrosis \|\| id:ebi-a-GCST90018120 | Idiopathic pulmonary fibrosis | Idiopathic pulmonary fibrosis \|\| \|\| | TRUE | igd | 0.0978 | 1.76E-06 | exposure | TRUE | reported | TJb989 | textfile | 2 | TRUE |
|  | 3 | rs117146485 | C | T | C | T | 0.2924 | -0.000159692 | 0.0149 | 0.01004 | FALSE | FALSE | FALSE | ebi-a-GCST90018120 | 9 | 138824257 | 0.000579852 | 437235 | 0.780001 | Idiopathic pulmonary fibrosis \|\| id:ebi-a-GCST90018120 | Idiopathic pulmonary fibrosis | Idiopathic pulmonary fibrosis \|\| \|\| | TRUE | igd | 0.0629 | 2.71E-06 | exposure | TRUE | reported | TJb989 | textfile | 2 | TRUE |
|  | 4 | rs17713451 | G | A | G | A | -0.1274 | 2.19E-05 | 0.835 | 0.863236 | FALSE | FALSE | FALSE | ebi-a-GCST90018120 | 7 | 151162472 | 0.000168487 | 437235 | 0.9 | Idiopathic pulmonary fibrosis \|\| id:ebi-a-GCST90018120 | Idiopathic pulmonary fibrosis | Idiopathic pulmonary fibrosis \|\| \|\| | TRUE | igd | 0.0253 | 4.97E-07 | exposure | TRUE | reported | TJb989 | textfile | 2 | TRUE |
|  | 5 | rs2332982 | G | A | G | A | -0.1118 | -0.000173205 | 0.1421 | 0.172968 | FALSE | FALSE | FALSE | ebi-a-GCST90018120 | 4 | 175953663 | 0.000153507 | 437235 | 0.26 | Idiopathic pulmonary fibrosis \|\| id:ebi-a-GCST90018120 | Idiopathic pulmonary fibrosis | Idiopathic pulmonary fibrosis \|\| \|\| | TRUE | igd | 0.0219 | 3.20E-07 | exposure | TRUE | reported | TJb989 | textfile | 2 | TRUE |
|  | 6 | rs6765768 | G | A | G | A | -0.0795 | -2.76E-05 | 0.3917 | 0.377833 | FALSE | FALSE | FALSE | ebi-a-GCST90018120 | 3 | 11704807 | 0.000119755 | 437235 | 0.82 | Idiopathic pulmonary fibrosis \|\| id:ebi-a-GCST90018120 | Idiopathic pulmonary fibrosis | Idiopathic pulmonary fibrosis \|\| \|\| | TRUE | igd | 0.0167 | 2.00E-06 | exposure | TRUE | reported | TJb989 | textfile | 2 | TRUE |
|  | 7 | rs73023729 | G | A | G | A | 0.1796 | -8.38E-05 | 0.9771 | 0.983918 | FALSE | FALSE | FALSE | ebi-a-GCST90018120 | 6 | 159654030 | 0.000459622 | 437235 | 0.86 | Idiopathic pulmonary fibrosis \|\| id:ebi-a-GCST90018120 | Idiopathic pulmonary fibrosis | Idiopathic pulmonary fibrosis \|\| \|\| | TRUE | igd | 0.0366 | 9.03E-07 | exposure | TRUE | reported | TJb989 | textfile | 2 | TRUE |
|  | 8 | rs7613691 | G | A | G | A | -0.1775 | -0.000107774 | 0.0527 | 0.062906 | FALSE | FALSE | FALSE | ebi-a-GCST90018120 | 3 | 147653591 | 0.000237903 | 437235 | 0.649999 | Idiopathic pulmonary fibrosis \|\| id:ebi-a-GCST90018120 | Idiopathic pulmonary fibrosis | Idiopathic pulmonary fibrosis \|\| \|\| | TRUE | igd | 0.0384 | 4.05E-06 | exposure | TRUE | reported | TJb989 | textfile | 2 | TRUE |
|  | 9 | rs79597994 | C | T | C | T | 0.5831 | -2.49E-05 | 0.9722 | 0.972905 | FALSE | FALSE | FALSE | ebi-a-GCST90018120 | 1 | 151803047 | 0.000372247 | 437235 | 0.95 | Idiopathic pulmonary fibrosis \|\| id:ebi-a-GCST90018120 | Idiopathic pulmonary fibrosis | Idiopathic pulmonary fibrosis \|\| \|\| | TRUE | igd | 0.127 | 4.32E-06 | exposure | TRUE | reported | TJb989 | textfile | 2 | TRUE |
|  | 10 | rs9508291 | C | T | C | T | 0.1676 | 0.000277145 | 0.0646 | 0.066126 | FALSE | FALSE | FALSE | ebi-a-GCST90018120 | 13 | 29710620 | 0.000232841 | 437235 | 0.23 | Idiopathic pulmonary fibrosis \|\| id:ebi-a-GCST90018120 | Idiopathic pulmonary fibrosis | Idiopathic pulmonary fibrosis \|\| \|\| | TRUE | igd | 0.0359 | 3.03E-06 | exposure | TRUE | reported | TJb989 | textfile | 2 | TRUE |
|  | 11 | rs9941733 | G | A | G | A | -0.114 | -1.79E-05 | 0.175 | 0.170796 | FALSE | FALSE | FALSE | ebi-a-GCST90018120 | 20 | 374061 | 0.000154427 | 437235 | 0.91 | Idiopathic pulmonary fibrosis \|\| id:ebi-a-GCST90018120 | Idiopathic pulmonary fibrosis | Idiopathic pulmonary fibrosis \|\| \|\| | TRUE | igd | 0.0229 | 6.88E-07 | exposure | TRUE | reported | TJb989 | textfile | 2 | TRUE |
|  |  |  |  |  |  |  |  |  |  |  |  |  |  |  |  |  |  |  |  |  |  |  |  |  |  |  |  |  |  |  |  |  |  |
| IL-16 |  | SNP | effect_allele.exposure | other_allele.exposure | effect_allele.outcome | other_allele.outcome | beta.exposure | beta.outcome | eaf.exposure | eaf.outcome | remove | palindromic | ambiguous | id.outcome | chr | pos | se.outcome | samplesize.outcome | pval.outcome | outcome | originalname.outcome | outcome.deprecated | mr_keep.outcome | data_source.outcome | se.exposure | pval.exposure | exposure | mr_keep.exposure | pval_origin.exposure | id.exposure | data_source.exposure | action | mr_keep |
|  | 1 | rs116135478 | G | A | G | A | -0.8206 | 0.000170153 | 0.0179 | 0.020532 | FALSE | FALSE | FALSE | ebi-a-GCST90018120 | 9 | 120519761 | 0.000408809 | 437235 | 0.68 | Idiopathic pulmonary fibrosis \|\| id:ebi-a-GCST90018120 | Idiopathic pulmonary fibrosis | Idiopathic pulmonary fibrosis \|\| \|\| | TRUE | igd | 0.166 | 3.66E-06 | exposure | TRUE | reported | Wmt3Pp | textfile | 2 | TRUE |
|  | 2 | rs117217798 | C | T | C | T | 0.2036 | -0.000118232 | 0.9314 | 0.931253 | FALSE | FALSE | FALSE | ebi-a-GCST90018120 | 17 | 31483233 | 0.000233593 | 437235 | 0.61 | Idiopathic pulmonary fibrosis \|\| id:ebi-a-GCST90018120 | Idiopathic pulmonary fibrosis | Idiopathic pulmonary fibrosis \|\| \|\| | TRUE | igd | 0.0444 | 4.15E-06 | exposure | TRUE | reported | Wmt3Pp | textfile | 2 | TRUE |
|  | 3 | rs117916513 | G | A | G | A | 0.502 | 0.00120103 | 0.9791 | 0.987196 | FALSE | FALSE | FALSE | ebi-a-GCST90018120 | 11 | 121264274 | 0.000518903 | 437235 | 0.021 | Idiopathic pulmonary fibrosis \|\| id:ebi-a-GCST90018120 | Idiopathic pulmonary fibrosis | Idiopathic pulmonary fibrosis \|\| \|\| | TRUE | igd | 0.0986 | 3.79E-07 | exposure | TRUE | reported | Wmt3Pp | textfile | 2 | TRUE |
|  | 4 | rs1255143 | C | T | C | T | -0.1306 | -1.29E-05 | 0.4344 | 0.460488 | FALSE | FALSE | FALSE | ebi-a-GCST90018120 | 10 | 130052200 | 0.000116655 | 437235 | 0.91 | Idiopathic pulmonary fibrosis \|\| id:ebi-a-GCST90018120 | Idiopathic pulmonary fibrosis | Idiopathic pulmonary fibrosis \|\| \|\| | TRUE | igd | 0.0242 | 7.10E-08 | exposure | TRUE | reported | Wmt3Pp | textfile | 2 | TRUE |
|  | 5 | rs12765671 | G | A | G | A | 0.6023 | 0.000374244 | 0.9831 | 0.973665 | FALSE | FALSE | FALSE | ebi-a-GCST90018120 | 10 | 106684169 | 0.000361596 | 437235 | 0.3 | Idiopathic pulmonary fibrosis \|\| id:ebi-a-GCST90018120 | Idiopathic pulmonary fibrosis | Idiopathic pulmonary fibrosis \|\| \|\| | TRUE | igd | 0.1318 | 4.84E-06 | exposure | TRUE | reported | Wmt3Pp | textfile | 2 | TRUE |
|  | 6 | rs144691581 | G | A | G | A | -0.4882 | -6.94E-05 | 0.9851 | 0.984696 | FALSE | FALSE | FALSE | ebi-a-GCST90018120 | 15 | 96953325 | 0.000491357 | 437235 | 0.89 | Idiopathic pulmonary fibrosis \|\| id:ebi-a-GCST90018120 | Idiopathic pulmonary fibrosis | Idiopathic pulmonary fibrosis \|\| \|\| | TRUE | igd | 0.0967 | 4.20E-07 | exposure | TRUE | reported | Wmt3Pp | textfile | 2 | TRUE |
|  | 7 | rs1801020 | G | A | G | A | -0.1733 | 0.000240206 | 0.7753 | 0.744931 | FALSE | FALSE | FALSE | ebi-a-GCST90018120 | 5 | 176836532 | 0.000133457 | 437235 | 0.0719996 | Idiopathic pulmonary fibrosis \|\| id:ebi-a-GCST90018120 | Idiopathic pulmonary fibrosis | Idiopathic pulmonary fibrosis \|\| \|\| | TRUE | igd | 0.0272 | 4.53E-10 | exposure | TRUE | reported | Wmt3Pp | textfile | 2 | TRUE |
|  | 8 | rs4253283 | C | T | C | T | -0.146 | -2.20E-05 | 0.6998 | 0.684038 | FALSE | FALSE | FALSE | ebi-a-GCST90018120 | 4 | 187165211 | 0.00012444 | 437235 | 0.86 | Idiopathic pulmonary fibrosis \|\| id:ebi-a-GCST90018120 | Idiopathic pulmonary fibrosis | Idiopathic pulmonary fibrosis \|\| \|\| | TRUE | igd | 0.0262 | 1.75E-08 | exposure | TRUE | reported | Wmt3Pp | textfile | 2 | TRUE |
|  | 9 | rs4513633 | C | A | C | A | 0.2239 | -8.97E-06 | 0.1243 | 0.132165 | FALSE | FALSE | FALSE | ebi-a-GCST90018120 | 4 | 113570639 | 0.000171252 | 437235 | 0.96 | Idiopathic pulmonary fibrosis \|\| id:ebi-a-GCST90018120 | Idiopathic pulmonary fibrosis | Idiopathic pulmonary fibrosis \|\| \|\| | TRUE | igd | 0.0453 | 7.44E-07 | exposure | TRUE | reported | Wmt3Pp | textfile | 2 | TRUE |
|  | 10 | rs4778636 | G | A | G | A | 0.7272 | 0.000138144 | 0.9254 | 0.907789 | FALSE | FALSE | FALSE | ebi-a-GCST90018120 | 15 | 81591639 | 0.000199685 | 437235 | 0.49 | Idiopathic pulmonary fibrosis \|\| id:ebi-a-GCST90018120 | Idiopathic pulmonary fibrosis | Idiopathic pulmonary fibrosis \|\| \|\| | TRUE | igd | 0.0633 | 1.11E-30 | exposure | TRUE | reported | Wmt3Pp | textfile | 2 | TRUE |
|  | 11 | rs9706053 | C | T | C | T | -0.4582 | -0.000359958 | 0.9841 | 0.97716 | FALSE | FALSE | FALSE | ebi-a-GCST90018120 | 12 | 66376310 | 0.000394073 | 437235 | 0.36 | Idiopathic pulmonary fibrosis \|\| id:ebi-a-GCST90018120 | Idiopathic pulmonary fibrosis | Idiopathic pulmonary fibrosis \|\| \|\| | TRUE | igd | 0.0932 | 7.01E-07 | exposure | TRUE | reported | Wmt3Pp | textfile | 2 | TRUE |
|  |  |  |  |  |  |  |  |  |  |  |  |  |  |  |  |  |  |  |  |  |  |  |  |  |  |  |  |  |  |  |  |  |  |
| IL-17 |  | SNP | effect_allele.exposure | other_allele.exposure | effect_allele.outcome | other_allele.outcome | beta.exposure | beta.outcome | eaf.exposure | eaf.outcome | remove | palindromic | ambiguous | id.outcome | chr | pos | se.outcome | samplesize.outcome | pval.outcome | outcome | originalname.outcome | outcome.deprecated | mr_keep.outcome | data_source.outcome | se.exposure | pval.exposure | exposure | mr_keep.exposure | pval_origin.exposure | id.exposure | data_source.exposure | action | mr_keep |
|  | 1 | rs117029961 | G | A | G | A | -0.4585 | -1.64E-05 | 0.9891 | 0.990785 | FALSE | FALSE | FALSE | ebi-a-GCST90018120 | 10 | 37436581 | 0.000607238 | 437235 | 0.98 | Idiopathic pulmonary fibrosis \|\| id:ebi-a-GCST90018120 | Idiopathic pulmonary fibrosis | Idiopathic pulmonary fibrosis \|\| \|\| | TRUE | igd | 0.1015 | 4.94E-06 | exposure | TRUE | reported | QhiCLK | textfile | 2 | TRUE |
|  | 2 | rs117556572 | C | T | C | T | 0.5102 | -0.00018108 | 0.9891 | 0.982421 | FALSE | FALSE | FALSE | ebi-a-GCST90018120 | 13 | 105088917 | 0.000439434 | 437235 | 0.68 | Idiopathic pulmonary fibrosis \|\| id:ebi-a-GCST90018120 | Idiopathic pulmonary fibrosis | Idiopathic pulmonary fibrosis \|\| \|\| | TRUE | igd | 0.1099 | 3.28E-06 | exposure | TRUE | reported | QhiCLK | textfile | 2 | TRUE |
|  | 3 | rs1530455 | C | T | C | T | -0.108 | 3.84E-05 | 0.5915 | 0.596242 | FALSE | FALSE | FALSE | ebi-a-GCST90018120 | 3 | 122854899 | 0.000119117 | 437235 | 0.75 | Idiopathic pulmonary fibrosis \|\| id:ebi-a-GCST90018120 | Idiopathic pulmonary fibrosis | Idiopathic pulmonary fibrosis \|\| \|\| | TRUE | igd | 0.0173 | 4.87E-10 | exposure | TRUE | reported | QhiCLK | textfile | 2 | TRUE |
|  | 4 | rs17106604 | C | T | C | T | -0.1129 | 6.26E-05 | 0.8757 | 0.888494 | FALSE | FALSE | FALSE | ebi-a-GCST90018120 | 14 | 78379156 | 0.000183812 | 437235 | 0.73 | Idiopathic pulmonary fibrosis \|\| id:ebi-a-GCST90018120 | Idiopathic pulmonary fibrosis | Idiopathic pulmonary fibrosis \|\| \|\| | TRUE | igd | 0.0225 | 6.37E-07 | exposure | TRUE | reported | QhiCLK | textfile | 2 | TRUE |
|  | 5 | rs17282552 | C | T | C | T | 0.2001 | 0.000277868 | 0.0268 | 0.032555 | FALSE | FALSE | FALSE | ebi-a-GCST90018120 | 2 | 207973815 | 0.000326299 | 437235 | 0.39 | Idiopathic pulmonary fibrosis \|\| id:ebi-a-GCST90018120 | Idiopathic pulmonary fibrosis | Idiopathic pulmonary fibrosis \|\| \|\| | TRUE | igd | 0.0405 | 8.21E-07 | exposure | TRUE | reported | QhiCLK | textfile | 2 | TRUE |
|  | 6 | rs184080173 | C | T | C | T | -0.2384 | 0.000102014 | 0.0626 | 0.066342 | FALSE | FALSE | FALSE | ebi-a-GCST90018120 | 12 | 77725204 | 0.000232942 | 437235 | 0.66 | Idiopathic pulmonary fibrosis \|\| id:ebi-a-GCST90018120 | Idiopathic pulmonary fibrosis | Idiopathic pulmonary fibrosis \|\| \|\| | TRUE | igd | 0.0471 | 4.19E-07 | exposure | TRUE | reported | QhiCLK | textfile | 2 | TRUE |
|  | 7 | rs187475560 | C | T | C | T | 0.2434 | 0.000113877 | 0.9881 | 0.989704 | FALSE | FALSE | FALSE | ebi-a-GCST90018120 | 4 | 161274563 | 0.000592113 | 437235 | 0.85 | Idiopathic pulmonary fibrosis \|\| id:ebi-a-GCST90018120 | Idiopathic pulmonary fibrosis | Idiopathic pulmonary fibrosis \|\| \|\| | TRUE | igd | 0.052 | 3.29E-06 | exposure | TRUE | reported | QhiCLK | textfile | 2 | TRUE |
|  | 8 | rs34120897 | C | A | C | A | 0.1055 | -0.000256264 | 0.0974 | 0.117925 | FALSE | FALSE | FALSE | ebi-a-GCST90018120 | 16 | 89795813 | 0.000179332 | 437235 | 0.15 | Idiopathic pulmonary fibrosis \|\| id:ebi-a-GCST90018120 | Idiopathic pulmonary fibrosis | Idiopathic pulmonary fibrosis \|\| \|\| | TRUE | igd | 0.0232 | 3.63E-06 | exposure | TRUE | reported | QhiCLK | textfile | 2 | TRUE |
|  | 9 | rs62191444 | G | T | G | T | 0.1136 | 5.06E-06 | 0.834 | 0.835496 | FALSE | FALSE | FALSE | ebi-a-GCST90018120 | 20 | 373667 | 0.000155931 | 437235 | 0.97 | Idiopathic pulmonary fibrosis \|\| id:ebi-a-GCST90018120 | Idiopathic pulmonary fibrosis | Idiopathic pulmonary fibrosis \|\| \|\| | TRUE | igd | 0.0247 | 4.22E-06 | exposure | TRUE | reported | QhiCLK | textfile | 2 | TRUE |
|  | 10 | rs78296352 | G | T | G | T | -0.3027 | -0.000455665 | 0.9712 | 0.961226 | FALSE | FALSE | FALSE | ebi-a-GCST90018120 | 1 | 22821844 | 0.000298985 | 437235 | 0.13 | Idiopathic pulmonary fibrosis \|\| id:ebi-a-GCST90018120 | Idiopathic pulmonary fibrosis | Idiopathic pulmonary fibrosis \|\| \|\| | TRUE | igd | 0.0646 | 4.27E-06 | exposure | TRUE | reported | QhiCLK | textfile | 2 | TRUE |
|  | 11 | rs78612928 | C | T | C | T | -0.1037 | 6.88E-05 | 0.1968 | 0.190562 | FALSE | FALSE | FALSE | ebi-a-GCST90018120 | 4 | 29813914 | 0.000147664 | 437235 | 0.64 | Idiopathic pulmonary fibrosis \|\| id:ebi-a-GCST90018120 | Idiopathic pulmonary fibrosis | Idiopathic pulmonary fibrosis \|\| \|\| | TRUE | igd | 0.0222 | 2.62E-06 | exposure | TRUE | reported | QhiCLK | textfile | 2 | TRUE |
|  | 12 | rs79462337 | G | T | G | T | -0.2097 | -0.000357911 | 0.0199 | 0.020464 | FALSE | FALSE | FALSE | ebi-a-GCST90018120 | 6 | 145508129 | 0.000443184 | 437235 | 0.42 | Idiopathic pulmonary fibrosis \|\| id:ebi-a-GCST90018120 | Idiopathic pulmonary fibrosis | Idiopathic pulmonary fibrosis \|\| \|\| | TRUE | igd | 0.0435 | 1.41E-06 | exposure | TRUE | reported | QhiCLK | textfile | 2 | TRUE |
|  |  |  |  |  |  |  |  |  |  |  |  |  |  |  |  |  |  |  |  |  |  |  |  |  |  |  |  |  |  |  |  |  |  |
| IL-18 |  | SNP | effect_allele.exposure | other_allele.exposure | effect_allele.outcome | other_allele.outcome | beta.exposure | beta.outcome | eaf.exposure | eaf.outcome | remove | palindromic | ambiguous | id.outcome | chr | pos | se.outcome | samplesize.outcome | pval.outcome | outcome | originalname.outcome | outcome.deprecated | mr_keep.outcome | data_source.outcome | se.exposure | pval.exposure | exposure | mr_keep.exposure | pval_origin.exposure | id.exposure | data_source.exposure | action | mr_keep |
|  | 1 | rs10414578 | C | T | C | T | 0.1771 | 6.52E-05 | 0.8787 | 0.887954 | FALSE | FALSE | FALSE | ebi-a-GCST90018120 | 19 | 55146070 | 0.000183515 | 437235 | 0.719999 | Idiopathic pulmonary fibrosis \|\| id:ebi-a-GCST90018120 | Idiopathic pulmonary fibrosis | Idiopathic pulmonary fibrosis \|\| \|\| | TRUE | igd | 0.035 | 4.16E-07 | exposure | TRUE | reported | hIkZCF | textfile | 2 | TRUE |
|  | 2 | rs115267715 | C | T | C | T | -0.4508 | 7.50E-05 | 0.9821 | 0.991332 | FALSE | FALSE | FALSE | ebi-a-GCST90018120 | 5 | 68535015 | 0.000624035 | 437235 | 0.91 | Idiopathic pulmonary fibrosis \|\| id:ebi-a-GCST90018120 | Idiopathic pulmonary fibrosis | Idiopathic pulmonary fibrosis \|\| \|\| | TRUE | igd | 0.08 | 1.72E-08 | exposure | TRUE | reported | hIkZCF | textfile | 2 | TRUE |
|  | 3 | rs116383510 | C | A | C | A | 0.5426 | -0.000146846 | 0.0199 | 0.013876 | FALSE | FALSE | FALSE | ebi-a-GCST90018120 | 5 | 2545650 | 0.000509155 | 437235 | 0.77 | Idiopathic pulmonary fibrosis \|\| id:ebi-a-GCST90018120 | Idiopathic pulmonary fibrosis | Idiopathic pulmonary fibrosis \|\| \|\| | TRUE | igd | 0.1056 | 3.00E-07 | exposure | TRUE | reported | hIkZCF | textfile | 2 | TRUE |
|  | 4 | rs11700536 | C | T | C | T | -0.1156 | 2.73E-05 | 0.6382 | 0.592025 | FALSE | FALSE | FALSE | ebi-a-GCST90018120 | 21 | 44558687 | 0.000118038 | 437235 | 0.82 | Idiopathic pulmonary fibrosis \|\| id:ebi-a-GCST90018120 | Idiopathic pulmonary fibrosis | Idiopathic pulmonary fibrosis \|\| \|\| | TRUE | igd | 0.025 | 4.21E-06 | exposure | TRUE | reported | hIkZCF | textfile | 2 | TRUE |
|  | 5 | rs117266781 | C | T | C | T | -0.6841 | 0.000465438 | 0.9891 | 0.987841 | FALSE | FALSE | FALSE | ebi-a-GCST90018120 | 7 | 41301020 | 0.000527414 | 437235 | 0.38 | Idiopathic pulmonary fibrosis \|\| id:ebi-a-GCST90018120 | Idiopathic pulmonary fibrosis | Idiopathic pulmonary fibrosis \|\| \|\| | TRUE | igd | 0.1468 | 3.15E-06 | exposure | TRUE | reported | hIkZCF | textfile | 2 | TRUE |
|  | 6 | rs144841621 | C | T | C | T | -0.518 | 0.000483518 | 0.9891 | 0.995369 | FALSE | FALSE | FALSE | ebi-a-GCST90018120 | 10 | 71681557 | 0.000885881 | 437235 | 0.59 | Idiopathic pulmonary fibrosis \|\| id:ebi-a-GCST90018120 | Idiopathic pulmonary fibrosis | Idiopathic pulmonary fibrosis \|\| \|\| | TRUE | igd | 0.1141 | 3.81E-06 | exposure | TRUE | reported | hIkZCF | textfile | 2 | TRUE |
|  | 7 | rs17229943 | C | A | C | A | 0.312 | 1.69E-06 | 0.0497 | 0.050998 | FALSE | FALSE | FALSE | ebi-a-GCST90018120 | 5 | 68682536 | 0.000262875 | 437235 | 0.99 | Idiopathic pulmonary fibrosis \|\| id:ebi-a-GCST90018120 | Idiopathic pulmonary fibrosis | Idiopathic pulmonary fibrosis \|\| \|\| | TRUE | igd | 0.0463 | 1.62E-11 | exposure | TRUE | reported | hIkZCF | textfile | 2 | TRUE |
|  | 8 | rs1852105 | C | T | C | T | -0.3036 | -0.000159015 | 0.9513 | 0.944323 | FALSE | FALSE | FALSE | ebi-a-GCST90018120 | 7 | 63725595 | 0.000252451 | 437235 | 0.53 | Idiopathic pulmonary fibrosis \|\| id:ebi-a-GCST90018120 | Idiopathic pulmonary fibrosis | Idiopathic pulmonary fibrosis \|\| \|\| | TRUE | igd | 0.0661 | 4.32E-06 | exposure | TRUE | reported | hIkZCF | textfile | 2 | TRUE |
|  | 9 | rs1979967 | C | T | C | T | -0.1402 | -0.000138627 | 0.7406 | 0.76824 | FALSE | FALSE | FALSE | ebi-a-GCST90018120 | 15 | 79659613 | 0.000137985 | 437235 | 0.31 | Idiopathic pulmonary fibrosis \|\| id:ebi-a-GCST90018120 | Idiopathic pulmonary fibrosis | Idiopathic pulmonary fibrosis \|\| \|\| | TRUE | igd | 0.0286 | 9.45E-07 | exposure | TRUE | reported | hIkZCF | textfile | 2 | TRUE |
|  | 10 | rs2729385 | G | A | G | A | -0.1231 | 1.41E-05 | 0.675 | 0.689112 | FALSE | FALSE | FALSE | ebi-a-GCST90018120 | 11 | 57262993 | 0.000124934 | 437235 | 0.91 | Idiopathic pulmonary fibrosis \|\| id:ebi-a-GCST90018120 | Idiopathic pulmonary fibrosis | Idiopathic pulmonary fibrosis \|\| \|\| | TRUE | igd | 0.0262 | 3.79E-06 | exposure | TRUE | reported | hIkZCF | textfile | 2 | TRUE |
|  | 11 | rs385076 | C | T | C | T | 0.2432 | 0.00010584 | 0.6471 | 0.644763 | FALSE | FALSE | FALSE | ebi-a-GCST90018120 | 2 | 32489851 | 0.000121768 | 437235 | 0.39 | Idiopathic pulmonary fibrosis \|\| id:ebi-a-GCST90018120 | Idiopathic pulmonary fibrosis | Idiopathic pulmonary fibrosis \|\| \|\| | TRUE | igd | 0.0248 | 1.66E-22 | exposure | TRUE | reported | hIkZCF | textfile | 2 | TRUE |
|  | 12 | rs4482818 | G | A | G | A | -0.1286 | 4.47E-05 | 0.3588 | 0.369726 | FALSE | FALSE | FALSE | ebi-a-GCST90018120 | 4 | 65928497 | 0.000119841 | 437235 | 0.709999 | Idiopathic pulmonary fibrosis \|\| id:ebi-a-GCST90018120 | Idiopathic pulmonary fibrosis | Idiopathic pulmonary fibrosis \|\| \|\| | TRUE | igd | 0.0244 | 1.45E-07 | exposure | TRUE | reported | hIkZCF | textfile | 2 | TRUE |
|  | 13 | rs658805 | G | A | G | A | -0.1226 | 0.000152845 | 0.6869 | 0.66968 | FALSE | FALSE | FALSE | ebi-a-GCST90018120 | 6 | 70909073 | 0.000123175 | 437235 | 0.21 | Idiopathic pulmonary fibrosis \|\| id:ebi-a-GCST90018120 | Idiopathic pulmonary fibrosis | Idiopathic pulmonary fibrosis \|\| \|\| | TRUE | igd | 0.0244 | 4.94E-07 | exposure | TRUE | reported | hIkZCF | textfile | 2 | TRUE |
|  | 14 | rs71478720 | C | T | C | T | 0.2669 | 0.000251642 | 0.7823 | 0.740939 | FALSE | FALSE | FALSE | ebi-a-GCST90018120 | 11 | 112009605 | 0.000132239 | 437235 | 0.0569994 | Idiopathic pulmonary fibrosis \|\| id:ebi-a-GCST90018120 | Idiopathic pulmonary fibrosis | Idiopathic pulmonary fibrosis \|\| \|\| | TRUE | igd | 0.0276 | 3.71E-22 | exposure | TRUE | reported | hIkZCF | textfile | 2 | TRUE |
|  | 15 | rs78623212 | C | T | C | T | -0.8705 | -0.000300642 | 0.9761 | 0.975198 | FALSE | FALSE | FALSE | ebi-a-GCST90018120 | 7 | 103307627 | 0.000375291 | 437235 | 0.42 | Idiopathic pulmonary fibrosis \|\| id:ebi-a-GCST90018120 | Idiopathic pulmonary fibrosis | Idiopathic pulmonary fibrosis \|\| \|\| | TRUE | igd | 0.1778 | 6.71E-07 | exposure | TRUE | reported | hIkZCF | textfile | 2 | TRUE |
|  | 16 | rs78716465 | G | A | G | A | -0.3265 | -0.000109635 | 0.9622 | 0.96029 | FALSE | FALSE | FALSE | ebi-a-GCST90018120 | 20 | 40643726 | 0.000302894 | 437235 | 0.719999 | Idiopathic pulmonary fibrosis \|\| id:ebi-a-GCST90018120 | Idiopathic pulmonary fibrosis | Idiopathic pulmonary fibrosis \|\| \|\| | TRUE | igd | 0.0682 | 1.63E-06 | exposure | TRUE | reported | hIkZCF | textfile | 2 | TRUE |
|  |  |  |  |  |  |  |  |  |  |  |  |  |  |  |  |  |  |  |  |  |  |  |  |  |  |  |  |  |  |  |  |  |  |
| IL1ra |  | SNP | effect_allele.exposure | other_allele.exposure | effect_allele.outcome | other_allele.outcome | beta.exposure | beta.outcome | eaf.exposure | eaf.outcome | remove | palindromic | ambiguous | id.outcome | chr | pos | se.outcome | samplesize.outcome | pval.outcome | outcome | originalname.outcome | outcome.deprecated | mr_keep.outcome | data_source.outcome | se.exposure | pval.exposure | exposure | mr_keep.exposure | pval_origin.exposure | id.exposure | data_source.exposure | action | mr_keep |
|  | 1 | rs1054402 | C | T | C | T | -0.1311 | -8.51E-05 | 0.7336 | 0.752136 | FALSE | FALSE | FALSE | ebi-a-GCST90018120 | 9 | 119163509 | 0.000134531 | 437235 | 0.52 | Idiopathic pulmonary fibrosis \|\| id:ebi-a-GCST90018120 | Idiopathic pulmonary fibrosis | Idiopathic pulmonary fibrosis \|\| \|\| | TRUE | igd | 0.027 | 1.13E-06 | exposure | TRUE | reported | XKPshm | textfile | 2 | TRUE |
|  | 2 | rs11627423 | C | A | C | A | -0.1171 | -9.51E-06 | 0.3897 | 0.407228 | FALSE | FALSE | FALSE | ebi-a-GCST90018120 | 14 | 33200623 | 0.000118083 | 437235 | 0.94 | Idiopathic pulmonary fibrosis \|\| id:ebi-a-GCST90018120 | Idiopathic pulmonary fibrosis | Idiopathic pulmonary fibrosis \|\| \|\| | TRUE | igd | 0.0247 | 2.12E-06 | exposure | TRUE | reported | XKPshm | textfile | 2 | TRUE |
|  | 3 | rs12121840 | C | T | C | T | -0.2692 | -0.000292901 | 0.9155 | 0.936099 | FALSE | FALSE | FALSE | ebi-a-GCST90018120 | 1 | 165541642 | 0.00024046 | 437235 | 0.22 | Idiopathic pulmonary fibrosis \|\| id:ebi-a-GCST90018120 | Idiopathic pulmonary fibrosis | Idiopathic pulmonary fibrosis \|\| \|\| | TRUE | igd | 0.0571 | 2.43E-06 | exposure | TRUE | reported | XKPshm | textfile | 2 | TRUE |
|  | 4 | rs139005642 | AAAAG | A | AAAAG | A | -0.1315 | 0.000168976 | 0.1471 | 0.144086 | FALSE | FALSE | FALSE | ebi-a-GCST90018120 | 22 | 22615606 | 0.000166359 | 437235 | 0.31 | Idiopathic pulmonary fibrosis \|\| id:ebi-a-GCST90018120 | Idiopathic pulmonary fibrosis | Idiopathic pulmonary fibrosis \|\| \|\| | TRUE | igd | 0.0277 | 1.97E-06 | exposure | TRUE | reported | XKPshm | textfile | 2 | TRUE |
|  | 5 | rs2809154 | C | T | C | T | 0.1786 | 6.70E-05 | 0.838 | 0.81535 | FALSE | FALSE | FALSE | ebi-a-GCST90018120 | 13 | 84727524 | 0.000149025 | 437235 | 0.649999 | Idiopathic pulmonary fibrosis \|\| id:ebi-a-GCST90018120 | Idiopathic pulmonary fibrosis | Idiopathic pulmonary fibrosis \|\| \|\| | TRUE | igd | 0.0388 | 3.74E-06 | exposure | TRUE | reported | XKPshm | textfile | 2 | TRUE |
|  | 6 | rs56134659 | G | A | G | A | 0.1117 | -5.79E-05 | 0.4742 | 0.441025 | FALSE | FALSE | FALSE | ebi-a-GCST90018120 | 3 | 129058985 | 0.000121239 | 437235 | 0.630001 | Idiopathic pulmonary fibrosis \|\| id:ebi-a-GCST90018120 | Idiopathic pulmonary fibrosis | Idiopathic pulmonary fibrosis \|\| \|\| | TRUE | igd | 0.0237 | 2.44E-06 | exposure | TRUE | reported | XKPshm | textfile | 2 | TRUE |
|  | 7 | rs61335305 | C | A | C | A | -0.4453 | -5.68E-05 | 0.9891 | 0.982026 | FALSE | FALSE | FALSE | ebi-a-GCST90018120 | 15 | 66453074 | 0.000434857 | 437235 | 0.9 | Idiopathic pulmonary fibrosis \|\| id:ebi-a-GCST90018120 | Idiopathic pulmonary fibrosis | Idiopathic pulmonary fibrosis \|\| \|\| | TRUE | igd | 0.0908 | 1.00E-06 | exposure | TRUE | reported | XKPshm | textfile | 2 | TRUE |
|  | 8 | rs9623661 | C | T | C | T | 0.1966 | -1.22E-05 | 0.9076 | 0.907625 | FALSE | FALSE | FALSE | ebi-a-GCST90018120 | 22 | 43093376 | 0.00020334 | 437235 | 0.95 | Idiopathic pulmonary fibrosis \|\| id:ebi-a-GCST90018120 | Idiopathic pulmonary fibrosis | Idiopathic pulmonary fibrosis \|\| \|\| | TRUE | igd | 0.0426 | 3.86E-06 | exposure | TRUE | reported | XKPshm | textfile | 2 | TRUE |
|  |  |  |  |  |  |  |  |  |  |  |  |  |  |  |  |  |  |  |  |  |  |  |  |  |  |  |  |  |  |  |  |  |  |
| IL2ra |  | SNP | effect_allele.exposure | other_allele.exposure | effect_allele.outcome | other_allele.outcome | beta.exposure | beta.outcome | eaf.exposure | eaf.outcome | remove | palindromic | ambiguous | id.outcome | chr | pos | se.outcome | samplesize.outcome | pval.outcome | outcome | originalname.outcome | outcome.deprecated | mr_keep.outcome | data_source.outcome | se.exposure | pval.exposure | exposure | mr_keep.exposure | pval_origin.exposure | id.exposure | data_source.exposure | action | mr_keep |
|  | 1 | rs11241559 | G | T | G | T | 0.1264 | -1.23E-05 | 0.7813 | 0.766098 | FALSE | FALSE | FALSE | ebi-a-GCST90018120 | 5 | 119976700 | 0.000136577 | 437235 | 0.93 | Idiopathic pulmonary fibrosis \|\| id:ebi-a-GCST90018120 | Idiopathic pulmonary fibrosis | Idiopathic pulmonary fibrosis \|\| \|\| | TRUE | igd | 0.0266 | 2.00E-06 | exposure | TRUE | reported | YxQJz2 | textfile | 2 | TRUE |
|  | 2 | rs115360066 | G | A | G | A | -0.1867 | 0.00058462 | 0.1103 | 0.119237 | FALSE | FALSE | FALSE | ebi-a-GCST90018120 | 5 | 9823768 | 0.000186788 | 437235 | 0.00179999 | Idiopathic pulmonary fibrosis \|\| id:ebi-a-GCST90018120 | Idiopathic pulmonary fibrosis | Idiopathic pulmonary fibrosis \|\| \|\| | TRUE | igd | 0.0379 | 8.06E-07 | exposure | TRUE | reported | YxQJz2 | textfile | 2 | TRUE |
|  | 3 | rs117244812 | G | A | G | A | 0.7064 | -0.000190565 | 0.9861 | 0.987938 | FALSE | FALSE | FALSE | ebi-a-GCST90018120 | 17 | 6443310 | 0.000543805 | 437235 | 0.73 | Idiopathic pulmonary fibrosis \|\| id:ebi-a-GCST90018120 | Idiopathic pulmonary fibrosis | Idiopathic pulmonary fibrosis \|\| \|\| | TRUE | igd | 0.1488 | 2.10E-06 | exposure | TRUE | reported | YxQJz2 | textfile | 2 | TRUE |
|  | 4 | rs12722497 | C | A | C | A | -0.6279 | 8.69E-05 | 0.8559 | 0.910894 | FALSE | FALSE | FALSE | ebi-a-GCST90018120 | 10 | 6095928 | 0.000206026 | 437235 | 0.67 | Idiopathic pulmonary fibrosis \|\| id:ebi-a-GCST90018120 | Idiopathic pulmonary fibrosis | Idiopathic pulmonary fibrosis \|\| \|\| | TRUE | igd | 0.0485 | 1.57E-38 | exposure | TRUE | reported | YxQJz2 | textfile | 2 | TRUE |
|  | 5 | rs185231391 | C | T | C | T | -0.8503 | 0.000650563 | 0.0149 | 0.012741 | FALSE | FALSE | FALSE | ebi-a-GCST90018120 | 3 | 59359679 | 0.000529741 | 437235 | 0.22 | Idiopathic pulmonary fibrosis \|\| id:ebi-a-GCST90018120 | Idiopathic pulmonary fibrosis | Idiopathic pulmonary fibrosis \|\| \|\| | TRUE | igd | 0.1809 | 1.47E-06 | exposure | TRUE | reported | YxQJz2 | textfile | 2 | TRUE |
|  | 6 | rs4733117 | C | A | C | A | -0.1369 | 0.000254696 | 0.164 | 0.144501 | FALSE | FALSE | FALSE | ebi-a-GCST90018120 | 8 | 32137610 | 0.000165705 | 437235 | 0.12 | Idiopathic pulmonary fibrosis \|\| id:ebi-a-GCST90018120 | Idiopathic pulmonary fibrosis | Idiopathic pulmonary fibrosis \|\| \|\| | TRUE | igd | 0.0292 | 2.63E-06 | exposure | TRUE | reported | YxQJz2 | textfile | 2 | TRUE |
|  | 7 | rs61705228 | C | T | C | T | -0.3303 | 0.000420518 | 0.9632 | 0.944928 | FALSE | FALSE | FALSE | ebi-a-GCST90018120 | 4 | 101196302 | 0.000254956 | 437235 | 0.0990011 | Idiopathic pulmonary fibrosis \|\| id:ebi-a-GCST90018120 | Idiopathic pulmonary fibrosis | Idiopathic pulmonary fibrosis \|\| \|\| | TRUE | igd | 0.0716 | 3.99E-06 | exposure | TRUE | reported | YxQJz2 | textfile | 2 | TRUE |
|  | 8 | rs62135626 | C | T | C | T | 0.1661 | 7.10E-05 | 0.8847 | 0.861822 | FALSE | FALSE | FALSE | ebi-a-GCST90018120 | 2 | 37911175 | 0.000167419 | 437235 | 0.67 | Idiopathic pulmonary fibrosis \|\| id:ebi-a-GCST90018120 | Idiopathic pulmonary fibrosis | Idiopathic pulmonary fibrosis \|\| \|\| | TRUE | igd | 0.035 | 1.99E-06 | exposure | TRUE | reported | YxQJz2 | textfile | 2 | TRUE |
|  |  |  |  |  |  |  |  |  |  |  |  |  |  |  |  |  |  |  |  |  |  |  |  |  |  |  |  |  |  |  |  |  |  |
| CRP |  | SNP | effect_allele.exposure | other_allele.exposure | effect_allele.outcome | other_allele.outcome | beta.exposure | beta.outcome | eaf.exposure | eaf.outcome | remove | palindromic | ambiguous | id.outcome | chr | pos | se.outcome | samplesize.outcome | pval.outcome | outcome | originalname.outcome | outcome.deprecated | mr_keep.outcome | data_source.outcome | se.exposure | pval.exposure | exposure | mr_keep.exposure | pval_origin.exposure | id.exposure | data_source.exposure | action | mr_keep |
|  | 1 | rs10169482 | G | T | G | T | -0.0143 | 2.85E-05 | 0.399612 | 0.609417 | FALSE | FALSE | FALSE | ebi-a-GCST90018120 | 2 | 61486628 | 0.000118603 | 437235 | 0.81 | Idiopathic pulmonary fibrosis \|\| id:ebi-a-GCST90018120 | Idiopathic pulmonary fibrosis | Idiopathic pulmonary fibrosis \|\| \|\| | TRUE | igd | 0.0024 | 4.05E-09 | exposure | TRUE | reported | MpDfko | textfile | 2 | TRUE |
|  | 2 | rs10408993 | T | C | T | C | 0.0332 | 2.71E-05 | 0.055316621 | 0.944203 | FALSE | FALSE | FALSE | ebi-a-GCST90018120 | 19 | 45305753 | 0.000251765 | 437235 | 0.91 | Idiopathic pulmonary fibrosis \|\| id:ebi-a-GCST90018120 | Idiopathic pulmonary fibrosis | Idiopathic pulmonary fibrosis \|\| \|\| | TRUE | igd | 0.0051 | 4.95E-11 | exposure | TRUE | reported | MpDfko | textfile | 2 | TRUE |
|  | 3 | rs10415983 | C | T | C | T | -0.024 | 4.73E-05 | 0.162925049 | 0.836533 | FALSE | FALSE | FALSE | ebi-a-GCST90018120 | 19 | 45711598 | 0.000156213 | 437235 | 0.760001 | Idiopathic pulmonary fibrosis \|\| id:ebi-a-GCST90018120 | Idiopathic pulmonary fibrosis | Idiopathic pulmonary fibrosis \|\| \|\| | TRUE | igd | 0.0032 | 3.82E-14 | exposure | TRUE | reported | MpDfko | textfile | 2 | TRUE |
|  | 4 | rs10417602 | C | T | C | T | -0.0354 | 5.49E-06 | 0.076251441 | 0.924138 | FALSE | FALSE | FALSE | ebi-a-GCST90018120 | 19 | 45660639 | 0.000218473 | 437235 | 0.98 | Idiopathic pulmonary fibrosis \|\| id:ebi-a-GCST90018120 | Idiopathic pulmonary fibrosis | Idiopathic pulmonary fibrosis \|\| \|\| | TRUE | igd | 0.0045 | 2.14E-15 | exposure | TRUE | reported | MpDfko | textfile | 2 | TRUE |
|  | 5 | rs10493377 | A | G | A | G | -0.0209 | -7.27E-05 | 0.45522931 | 0.545803 | FALSE | FALSE | FALSE | ebi-a-GCST90018120 | 1 | 65879252 | 0.000116085 | 437235 | 0.53 | Idiopathic pulmonary fibrosis \|\| id:ebi-a-GCST90018120 | Idiopathic pulmonary fibrosis | Idiopathic pulmonary fibrosis \|\| \|\| | TRUE | igd | 0.0024 | 1.57E-18 | exposure | TRUE | reported | MpDfko | textfile | 2 | TRUE |
|  | 6 | rs10501349 | G | A | G | A | -0.023 | 0.000283822 | 0.125954915 | 0.874674 | FALSE | FALSE | FALSE | ebi-a-GCST90018120 | 11 | 56021284 | 0.000174617 | 437235 | 0.1 | Idiopathic pulmonary fibrosis \|\| id:ebi-a-GCST90018120 | Idiopathic pulmonary fibrosis | Idiopathic pulmonary fibrosis \|\| \|\| | TRUE | igd | 0.0037 | 4.31E-10 | exposure | TRUE | reported | MpDfko | textfile | 2 | TRUE |
|  | 7 | rs10521222 | C | T | C | T | -0.1014 | 2.47E-05 | 0.047815444 | 0.951927 | FALSE | FALSE | FALSE | ebi-a-GCST90018120 | 16 | 51158710 | 0.000270364 | 437235 | 0.93 | Idiopathic pulmonary fibrosis \|\| id:ebi-a-GCST90018120 | Idiopathic pulmonary fibrosis | Idiopathic pulmonary fibrosis \|\| \|\| | TRUE | igd | 0.0056 | 9.19E-73 | exposure | TRUE | reported | MpDfko | textfile | 2 | TRUE |
|  | 8 | rs1052373 | C | T | C | T | -0.024 | 0.000161442 | 0.322462752 | 0.677553 | FALSE | FALSE | FALSE | ebi-a-GCST90018120 | 11 | 47354787 | 0.000123658 | 437235 | 0.19 | Idiopathic pulmonary fibrosis \|\| id:ebi-a-GCST90018120 | Idiopathic pulmonary fibrosis | Idiopathic pulmonary fibrosis \|\| \|\| | TRUE | igd | 0.0026 | 7.16E-21 | exposure | TRUE | reported | MpDfko | textfile | 2 | TRUE |
|  | 9 | rs1064725 | T | G | T | G | 0.0472 | 0.000181249 | 0.041512472 | 0.958695 | FALSE | FALSE | FALSE | ebi-a-GCST90018120 | 19 | 45422561 | 0.000301248 | 437235 | 0.55 | Idiopathic pulmonary fibrosis \|\| id:ebi-a-GCST90018120 | Idiopathic pulmonary fibrosis | Idiopathic pulmonary fibrosis \|\| \|\| | TRUE | igd | 0.0062 | 4.08E-14 | exposure | TRUE | reported | MpDfko | textfile | 2 | TRUE |
|  | 10 | rs10810455 | C | G | C | G | -0.0145 | -0.000239844 | 0.433762 | 0.441207 | FALSE | TRUE | TRUE | ebi-a-GCST90018120 | 9 | 15866156 | 0.000116637 | 437235 | 0.04 | Idiopathic pulmonary fibrosis \|\| id:ebi-a-GCST90018120 | Idiopathic pulmonary fibrosis | Idiopathic pulmonary fibrosis \|\| \|\| | TRUE | igd | 0.0024 | 1.90E-09 | exposure | TRUE | reported | MpDfko | textfile | 2 | FALSE |
|  | 11 | rs10849772 | G | A | G | A | -0.0759 | 0.000550923 | 0.044945088 | 0.954596 | FALSE | FALSE | FALSE | ebi-a-GCST90018120 | 12 | 121070251 | 0.000277832 | 437235 | 0.0470002 | Idiopathic pulmonary fibrosis \|\| id:ebi-a-GCST90018120 | Idiopathic pulmonary fibrosis | Idiopathic pulmonary fibrosis \|\| \|\| | TRUE | igd | 0.0055 | 1.16E-43 | exposure | TRUE | reported | MpDfko | textfile | 2 | TRUE |
|  | 12 | rs10851685 | T | A | T | A | 0.0219 | 0.000192173 | 0.133916308 | 0.13501 | FALSE | TRUE | FALSE | ebi-a-GCST90018120 | 15 | 60855200 | 0.00016925 | 437235 | 0.26 | Idiopathic pulmonary fibrosis \|\| id:ebi-a-GCST90018120 | Idiopathic pulmonary fibrosis | Idiopathic pulmonary fibrosis \|\| \|\| | TRUE | igd | 0.0035 | 3.05E-10 | exposure | TRUE | reported | MpDfko | textfile | 2 | TRUE |
|  | 13 | rs10951261 | G | A | G | A | -0.0187 | -0.000101595 | 0.275415416 | 0.726595 | FALSE | FALSE | FALSE | ebi-a-GCST90018120 | 7 | 998802 | 0.00012968 | 437235 | 0.43 | Idiopathic pulmonary fibrosis \|\| id:ebi-a-GCST90018120 | Idiopathic pulmonary fibrosis | Idiopathic pulmonary fibrosis \|\| \|\| | TRUE | igd | 0.0027 | 3.96E-12 | exposure | TRUE | reported | MpDfko | textfile | 2 | TRUE |
|  | 14 | rs11039798 | G | A | G | A | -0.0284 | 0.000223006 | 0.129007194 | 0.871891 | FALSE | FALSE | FALSE | ebi-a-GCST90018120 | 11 | 48540223 | 0.000172946 | 437235 | 0.2 | Idiopathic pulmonary fibrosis \|\| id:ebi-a-GCST90018120 | Idiopathic pulmonary fibrosis | Idiopathic pulmonary fibrosis \|\| \|\| | TRUE | igd | 0.0036 | 6.32E-15 | exposure | TRUE | reported | MpDfko | textfile | 2 | TRUE |
|  | 15 | rs11065079 | C | T | C | T | -0.0876 | -8.77E-05 | 0.020523562 | 0.979208 | FALSE | FALSE | FALSE | ebi-a-GCST90018120 | 12 | 120752247 | 0.000405142 | 437235 | 0.83 | Idiopathic pulmonary fibrosis \|\| id:ebi-a-GCST90018120 | Idiopathic pulmonary fibrosis | Idiopathic pulmonary fibrosis \|\| \|\| | TRUE | igd | 0.0085 | 9.59E-25 | exposure | TRUE | reported | MpDfko | textfile | 2 | TRUE |
|  | 16 | rs11065219 | C | T | C | T | -0.0277 | 0.000169876 | 0.104504537 | 0.895178 | FALSE | FALSE | FALSE | ebi-a-GCST90018120 | 12 | 121146219 | 0.000188564 | 437235 | 0.37 | Idiopathic pulmonary fibrosis \|\| id:ebi-a-GCST90018120 | Idiopathic pulmonary fibrosis | Idiopathic pulmonary fibrosis \|\| \|\| | TRUE | igd | 0.0039 | 1.55E-12 | exposure | TRUE | reported | MpDfko | textfile | 2 | TRUE |
|  | 17 | rs11065387 | G | C | G | C | -0.116 | 0.000491346 | 0.023751646 | 0.024396 | FALSE | TRUE | FALSE | ebi-a-GCST90018120 | 12 | 121427653 | 0.000374958 | 437235 | 0.19 | Idiopathic pulmonary fibrosis \|\| id:ebi-a-GCST90018120 | Idiopathic pulmonary fibrosis | Idiopathic pulmonary fibrosis \|\| \|\| | TRUE | igd | 0.0079 | 5.93E-49 | exposure | TRUE | reported | MpDfko | textfile | 2 | TRUE |
|  | 18 | rs11077359 | T | C | T | C | 0.0307 | 0.000204371 | 0.208813495 | 0.209539 | FALSE | FALSE | FALSE | ebi-a-GCST90018120 | 17 | 76343961 | 0.000141993 | 437235 | 0.15 | Idiopathic pulmonary fibrosis \|\| id:ebi-a-GCST90018120 | Idiopathic pulmonary fibrosis | Idiopathic pulmonary fibrosis \|\| \|\| | TRUE | igd | 0.0029 | 5.53E-26 | exposure | TRUE | reported | MpDfko | textfile | 2 | TRUE |
|  | 19 | rs111231035 | T | C | T | C | 0.0829 | 0.000333176 | 0.024181426 | 0.975713 | FALSE | FALSE | FALSE | ebi-a-GCST90018120 | 1 | 159253133 | 0.00037585 | 437235 | 0.38 | Idiopathic pulmonary fibrosis \|\| id:ebi-a-GCST90018120 | Idiopathic pulmonary fibrosis | Idiopathic pulmonary fibrosis \|\| \|\| | TRUE | igd | 0.0078 | 3.34E-26 | exposure | TRUE | reported | MpDfko | textfile | 2 | TRUE |
|  | 20 | rs11203042 | T | C | T | C | -0.0145 | -9.85E-05 | 0.447186265 | 0.447241 | FALSE | FALSE | FALSE | ebi-a-GCST90018120 | 10 | 90989109 | 0.000116327 | 437235 | 0.4 | Idiopathic pulmonary fibrosis \|\| id:ebi-a-GCST90018120 | Idiopathic pulmonary fibrosis | Idiopathic pulmonary fibrosis \|\| \|\| | TRUE | igd | 0.0024 | 1.29E-09 | exposure | TRUE | reported | MpDfko | textfile | 2 | TRUE |
|  | 21 | rs11208629 | G | A | G | A | -0.0323 | -0.000158754 | 0.080783679 | 0.918884 | FALSE | FALSE | FALSE | ebi-a-GCST90018120 | 1 | 65781901 | 0.000211792 | 437235 | 0.450001 | Idiopathic pulmonary fibrosis \|\| id:ebi-a-GCST90018120 | Idiopathic pulmonary fibrosis | Idiopathic pulmonary fibrosis \|\| \|\| | TRUE | igd | 0.0044 | 2.40E-13 | exposure | TRUE | reported | MpDfko | textfile | 2 | TRUE |
|  | 22 | rs11249926 | C | T | C | T | 0.0151 | -5.69E-05 | 0.489001561 | 0.489849 | FALSE | FALSE | FALSE | ebi-a-GCST90018120 | 8 | 9355784 | 0.000115698 | 437235 | 0.62 | Idiopathic pulmonary fibrosis \|\| id:ebi-a-GCST90018120 | Idiopathic pulmonary fibrosis | Idiopathic pulmonary fibrosis \|\| \|\| | TRUE | igd | 0.0024 | 3.32E-10 | exposure | TRUE | reported | MpDfko | textfile | 2 | TRUE |
|  | 23 | rs112585178 | G | T | G | T | -0.0658 | 0.000547176 | 0.03237234 | 0.967658 | FALSE | FALSE | FALSE | ebi-a-GCST90018120 | 1 | 66154563 | 0.000326687 | 437235 | 0.0940005 | Idiopathic pulmonary fibrosis \|\| id:ebi-a-GCST90018120 | Idiopathic pulmonary fibrosis | Idiopathic pulmonary fibrosis \|\| \|\| | TRUE | igd | 0.0068 | 2.65E-22 | exposure | TRUE | reported | MpDfko | textfile | 2 | TRUE |
|  | 24 | rs11265260 | A | G | A | G | 0.2137 | -2.14E-05 | 0.057145209 | 0.942715 | FALSE | FALSE | FALSE | ebi-a-GCST90018120 | 1 | 159700039 | 0.000248704 | 437235 | 0.93 | Idiopathic pulmonary fibrosis \|\| id:ebi-a-GCST90018120 | Idiopathic pulmonary fibrosis | Idiopathic pulmonary fibrosis \|\| \|\| | TRUE | igd | 0.0051 | 1.00E-200 | exposure | TRUE | reported | MpDfko | textfile | 2 | TRUE |
|  | 25 | rs11265608 | G | A | G | A | 0.0438 | -0.000146935 | 0.103417781 | 0.896975 | FALSE | FALSE | FALSE | ebi-a-GCST90018120 | 1 | 154364140 | 0.000189985 | 437235 | 0.44 | Idiopathic pulmonary fibrosis \|\| id:ebi-a-GCST90018120 | Idiopathic pulmonary fibrosis | Idiopathic pulmonary fibrosis \|\| \|\| | TRUE | igd | 0.0039 | 6.39E-29 | exposure | TRUE | reported | MpDfko | textfile | 2 | TRUE |
|  | 26 | rs112749520 | C | A | C | A | -0.0853 | 0.000264493 | 0.015738535 | 0.984534 | FALSE | FALSE | FALSE | ebi-a-GCST90018120 | 1 | 153823728 | 0.00046816 | 437235 | 0.57 | Idiopathic pulmonary fibrosis \|\| id:ebi-a-GCST90018120 | Idiopathic pulmonary fibrosis | Idiopathic pulmonary fibrosis \|\| \|\| | TRUE | igd | 0.0098 | 2.75E-18 | exposure | TRUE | reported | MpDfko | textfile | 2 | TRUE |
|  | 27 | rs113116967 | G | T | G | T | -0.0336 | 3.56E-05 | 0.054529339 | 0.946327 | FALSE | FALSE | FALSE | ebi-a-GCST90018120 | 12 | 120430074 | 0.000256332 | 437235 | 0.89 | Idiopathic pulmonary fibrosis \|\| id:ebi-a-GCST90018120 | Idiopathic pulmonary fibrosis | Idiopathic pulmonary fibrosis \|\| \|\| | TRUE | igd | 0.0053 | 2.63E-10 | exposure | TRUE | reported | MpDfko | textfile | 2 | TRUE |
|  | 28 | rs113188187 | C | T | C | T | 0.105 | 0.000343081 | 0.022636925 | 0.977201 | FALSE | FALSE | FALSE | ebi-a-GCST90018120 | 1 | 159677904 | 0.000386995 | 437235 | 0.37 | Idiopathic pulmonary fibrosis \|\| id:ebi-a-GCST90018120 | Idiopathic pulmonary fibrosis | Idiopathic pulmonary fibrosis \|\| \|\| | TRUE | igd | 0.008 | 2.94E-39 | exposure | TRUE | reported | MpDfko | textfile | 2 | TRUE |
|  | 29 | rs113401670 | C | T | C | T | -0.062 | -0.000275505 | 0.019112372 | 0.981231 | FALSE | FALSE | FALSE | ebi-a-GCST90018120 | 15 | 53675194 | 0.000426144 | 437235 | 0.52 | Idiopathic pulmonary fibrosis \|\| id:ebi-a-GCST90018120 | Idiopathic pulmonary fibrosis | Idiopathic pulmonary fibrosis \|\| \|\| | TRUE | igd | 0.0088 | 2.35E-12 | exposure | TRUE | reported | MpDfko | textfile | 2 | TRUE |
|  | 30 | rs113405869 | G | T | G | T | 0.0218 | 4.61E-05 | 0.126280178 | 0.873553 | FALSE | FALSE | FALSE | ebi-a-GCST90018120 | 19 | 45620513 | 0.000173911 | 437235 | 0.79 | Idiopathic pulmonary fibrosis \|\| id:ebi-a-GCST90018120 | Idiopathic pulmonary fibrosis | Idiopathic pulmonary fibrosis \|\| \|\| | TRUE | igd | 0.0036 | 1.82E-09 | exposure | TRUE | reported | MpDfko | textfile | 2 | TRUE |
|  | 31 | rs113467192 | C | T | C | T | 0.0754 | -0.000478942 | 0.021004176 | 0.978885 | FALSE | FALSE | FALSE | ebi-a-GCST90018120 | 1 | 66163796 | 0.000402329 | 437235 | 0.23 | Idiopathic pulmonary fibrosis \|\| id:ebi-a-GCST90018120 | Idiopathic pulmonary fibrosis | Idiopathic pulmonary fibrosis \|\| \|\| | TRUE | igd | 0.0083 | 7.02E-20 | exposure | TRUE | reported | MpDfko | textfile | 2 | TRUE |
|  | 32 | rs113838402 | C | T | C | T | -0.0483 | -0.000306831 | 0.025923091 | 0.974491 | FALSE | FALSE | FALSE | ebi-a-GCST90018120 | 12 | 121601748 | 0.0003666 | 437235 | 0.4 | Idiopathic pulmonary fibrosis \|\| id:ebi-a-GCST90018120 | Idiopathic pulmonary fibrosis | Idiopathic pulmonary fibrosis \|\| \|\| | TRUE | igd | 0.0075 | 9.27E-11 | exposure | TRUE | reported | MpDfko | textfile | 2 | TRUE |
|  | 33 | rs114272969 | G | A | G | A | 0.0962 | 0.000600998 | 0.011648158 | 0.988312 | FALSE | FALSE | FALSE | ebi-a-GCST90018120 | 1 | 159209865 | 0.000536931 | 437235 | 0.26 | Idiopathic pulmonary fibrosis \|\| id:ebi-a-GCST90018120 | Idiopathic pulmonary fibrosis | Idiopathic pulmonary fibrosis \|\| \|\| | TRUE | igd | 0.0113 | 1.59E-17 | exposure | TRUE | reported | MpDfko | textfile | 2 | TRUE |
|  | 34 | rs114530473 | C | T | C | T | 0.1034 | 0.000125933 | 0.023204184 | 0.976436 | FALSE | FALSE | FALSE | ebi-a-GCST90018120 | 1 | 159723800 | 0.000381047 | 437235 | 0.74 | Idiopathic pulmonary fibrosis \|\| id:ebi-a-GCST90018120 | Idiopathic pulmonary fibrosis | Idiopathic pulmonary fibrosis \|\| \|\| | TRUE | igd | 0.008 | 1.68E-38 | exposure | TRUE | reported | MpDfko | textfile | 2 | TRUE |
|  | 35 | rs114669363 | G | A | G | A | -0.0653 | -0.000306062 | 0.017724221 | 0.982403 | FALSE | FALSE | FALSE | ebi-a-GCST90018120 | 1 | 66516655 | 0.000439322 | 437235 | 0.49 | Idiopathic pulmonary fibrosis \|\| id:ebi-a-GCST90018120 | Idiopathic pulmonary fibrosis | Idiopathic pulmonary fibrosis \|\| \|\| | TRUE | igd | 0.0092 | 1.33E-12 | exposure | TRUE | reported | MpDfko | textfile | 2 | TRUE |
|  | 36 | rs114695117 | C | A | C | A | -0.049 | -0.000649899 | 0.026641338 | 0.973605 | FALSE | FALSE | FALSE | ebi-a-GCST90018120 | 1 | 65209111 | 0.000360625 | 437235 | 0.0710003 | Idiopathic pulmonary fibrosis \|\| id:ebi-a-GCST90018120 | Idiopathic pulmonary fibrosis | Idiopathic pulmonary fibrosis \|\| \|\| | TRUE | igd | 0.0075 | 7.97E-11 | exposure | TRUE | reported | MpDfko | textfile | 2 | TRUE |
|  | 37 | rs115165870 | G | A | G | A | 0.0431 | -0.000279235 | 0.041058624 | 0.959079 | FALSE | FALSE | FALSE | ebi-a-GCST90018120 | 1 | 154303228 | 0.000292147 | 437235 | 0.34 | Idiopathic pulmonary fibrosis \|\| id:ebi-a-GCST90018120 | Idiopathic pulmonary fibrosis | Idiopathic pulmonary fibrosis \|\| \|\| | TRUE | igd | 0.0061 | 1.49E-12 | exposure | TRUE | reported | MpDfko | textfile | 2 | TRUE |
|  | 38 | rs115236337 | C | T | C | T | 0.0329 | -0.000363393 | 0.067627139 | 0.932999 | FALSE | FALSE | FALSE | ebi-a-GCST90018120 | 1 | 39662602 | 0.000231306 | 437235 | 0.12 | Idiopathic pulmonary fibrosis \|\| id:ebi-a-GCST90018120 | Idiopathic pulmonary fibrosis | Idiopathic pulmonary fibrosis \|\| \|\| | TRUE | igd | 0.0048 | 9.52E-12 | exposure | TRUE | reported | MpDfko | textfile | 2 | TRUE |
|  | 39 | rs115368096 | T | C | T | C | -0.0466 | -0.00040555 | 0.023727777 | 0.97603 | FALSE | FALSE | FALSE | ebi-a-GCST90018120 | 1 | 159336134 | 0.000378233 | 437235 | 0.28 | Idiopathic pulmonary fibrosis \|\| id:ebi-a-GCST90018120 | Idiopathic pulmonary fibrosis | Idiopathic pulmonary fibrosis \|\| \|\| | TRUE | igd | 0.0078 | 2.03E-09 | exposure | TRUE | reported | MpDfko | textfile | 2 | TRUE |
|  | 40 | rs115381557 | C | T | C | T | 0.2123 | 0.000662143 | 0.009674794 | 0.989602 | FALSE | FALSE | FALSE | ebi-a-GCST90018120 | 1 | 159725183 | 0.000569641 | 437235 | 0.24 | Idiopathic pulmonary fibrosis \|\| id:ebi-a-GCST90018120 | Idiopathic pulmonary fibrosis | Idiopathic pulmonary fibrosis \|\| \|\| | TRUE | igd | 0.0123 | 2.24E-66 | exposure | TRUE | reported | MpDfko | textfile | 2 | TRUE |
|  | 41 | rs115585839 | G | T | G | T | 0.1081 | 7.69E-05 | 0.021857721 | 0.978449 | FALSE | FALSE | FALSE | ebi-a-GCST90018120 | 1 | 159726586 | 0.00039768 | 437235 | 0.85 | Idiopathic pulmonary fibrosis \|\| id:ebi-a-GCST90018120 | Idiopathic pulmonary fibrosis | Idiopathic pulmonary fibrosis \|\| \|\| | TRUE | igd | 0.0083 | 8.09E-39 | exposure | TRUE | reported | MpDfko | textfile | 2 | TRUE |
|  | 42 | rs115615753 | T | C | T | C | 0.0611 | -4.79E-05 | 0.038863558 | 0.960879 | FALSE | FALSE | FALSE | ebi-a-GCST90018120 | 1 | 159359030 | 0.000298311 | 437235 | 0.87 | Idiopathic pulmonary fibrosis \|\| id:ebi-a-GCST90018120 | Idiopathic pulmonary fibrosis | Idiopathic pulmonary fibrosis \|\| \|\| | TRUE | igd | 0.0063 | 2.18E-22 | exposure | TRUE | reported | MpDfko | textfile | 2 | TRUE |
|  | 43 | rs11570051 | G | A | G | A | -0.0361 | 0.000252987 | 0.055500324 | 0.948434 | FALSE | FALSE | FALSE | ebi-a-GCST90018120 | 11 | 47371442 | 0.000272258 | 437235 | 0.35 | Idiopathic pulmonary fibrosis \|\| id:ebi-a-GCST90018120 | Idiopathic pulmonary fibrosis | Idiopathic pulmonary fibrosis \|\| \|\| | TRUE | igd | 0.0056 | 1.09E-10 | exposure | TRUE | reported | MpDfko | textfile | 2 | TRUE |
|  | 44 | rs1160984 | C | T | C | T | 0.0722 | 0.000297589 | 0.058187034 | 0.941741 | FALSE | FALSE | FALSE | ebi-a-GCST90018120 | 19 | 45403924 | 0.000247808 | 437235 | 0.23 | Idiopathic pulmonary fibrosis \|\| id:ebi-a-GCST90018120 | Idiopathic pulmonary fibrosis | Idiopathic pulmonary fibrosis \|\| \|\| | TRUE | igd | 0.0054 | 4.52E-41 | exposure | TRUE | reported | MpDfko | textfile | 2 | TRUE |
|  | 45 | rs11611673 | A | G | A | G | -0.0915 | -5.22E-05 | 0.025068869 | 0.974749 | FALSE | FALSE | FALSE | ebi-a-GCST90018120 | 12 | 121451150 | 0.000368492 | 437235 | 0.89 | Idiopathic pulmonary fibrosis \|\| id:ebi-a-GCST90018120 | Idiopathic pulmonary fibrosis | Idiopathic pulmonary fibrosis \|\| \|\| | TRUE | igd | 0.0076 | 5.32E-33 | exposure | TRUE | reported | MpDfko | textfile | 2 | TRUE |
|  | 46 | rs11640223 | G | T | G | T | 0.0235 | 1.18E-05 | 0.209370688 | 0.788493 | FALSE | FALSE | FALSE | ebi-a-GCST90018120 | 16 | 51111246 | 0.000141338 | 437235 | 0.93 | Idiopathic pulmonary fibrosis \|\| id:ebi-a-GCST90018120 | Idiopathic pulmonary fibrosis | Idiopathic pulmonary fibrosis \|\| \|\| | TRUE | igd | 0.0029 | 1.52E-15 | exposure | TRUE | reported | MpDfko | textfile | 2 | TRUE |
|  | 47 | rs116509635 | C | T | C | T | 0.0686 | -0.00110773 | 0.018153693 | 0.981605 | FALSE | FALSE | FALSE | ebi-a-GCST90018120 | 1 | 65810601 | 0.000430303 | 437235 | 0.00990011 | Idiopathic pulmonary fibrosis \|\| id:ebi-a-GCST90018120 | Idiopathic pulmonary fibrosis | Idiopathic pulmonary fibrosis \|\| \|\| | TRUE | igd | 0.009 | 2.03E-14 | exposure | TRUE | reported | MpDfko | textfile | 2 | TRUE |
|  | 48 | rs116643857 | T | C | T | C | 0.0583 | -9.60E-05 | 0.023681397 | 0.976144 | FALSE | FALSE | FALSE | ebi-a-GCST90018120 | 1 | 66256127 | 0.000378462 | 437235 | 0.8 | Idiopathic pulmonary fibrosis \|\| id:ebi-a-GCST90018120 | Idiopathic pulmonary fibrosis | Idiopathic pulmonary fibrosis \|\| \|\| | TRUE | igd | 0.0079 | 1.92E-13 | exposure | TRUE | reported | MpDfko | textfile | 2 | TRUE |
|  | 49 | rs11665829 | G | A | G | A | -0.0366 | 2.98E-05 | 0.376599067 | 0.623472 | FALSE | FALSE | FALSE | ebi-a-GCST90018120 | 19 | 45365817 | 0.000119323 | 437235 | 0.8 | Idiopathic pulmonary fibrosis \|\| id:ebi-a-GCST90018120 | Idiopathic pulmonary fibrosis | Idiopathic pulmonary fibrosis \|\| \|\| | TRUE | igd | 0.0025 | 6.21E-50 | exposure | TRUE | reported | MpDfko | textfile | 2 | TRUE |
|  | 50 | rs11667234 | C | A | C | A | 0.034 | -0.000469973 | 0.0449433 | 0.952905 | FALSE | FALSE | FALSE | ebi-a-GCST90018120 | 19 | 11019581 | 0.000273048 | 437235 | 0.0850002 | Idiopathic pulmonary fibrosis \|\| id:ebi-a-GCST90018120 | Idiopathic pulmonary fibrosis | Idiopathic pulmonary fibrosis \|\| \|\| | TRUE | igd | 0.0057 | 2.83E-09 | exposure | TRUE | reported | MpDfko | textfile | 2 | TRUE |
|  | 51 | rs11668327 | G | C | G | C | 0.0778 | -0.000147733 | 0.175577856 | 0.175569 | FALSE | TRUE | FALSE | ebi-a-GCST90018120 | 19 | 45398633 | 0.00015392 | 437235 | 0.34 | Idiopathic pulmonary fibrosis \|\| id:ebi-a-GCST90018120 | Idiopathic pulmonary fibrosis | Idiopathic pulmonary fibrosis \|\| \|\| | TRUE | igd | 0.0033 | 7.05E-122 | exposure | TRUE | reported | MpDfko | textfile | 2 | TRUE |
|  | 52 | rs116699695 | C | A | C | A | -0.0599 | 2.26E-05 | 0.02117999 | 0.978586 | FALSE | FALSE | FALSE | ebi-a-GCST90018120 | 1 | 66242007 | 0.000400157 | 437235 | 0.96 | Idiopathic pulmonary fibrosis \|\| id:ebi-a-GCST90018120 | Idiopathic pulmonary fibrosis | Idiopathic pulmonary fibrosis \|\| \|\| | TRUE | igd | 0.0084 | 7.82E-13 | exposure | TRUE | reported | MpDfko | textfile | 2 | TRUE |
|  | 53 | rs11673516 | T | G | T | G | -0.0154 | 2.49E-05 | 0.432226393 | 0.570404 | FALSE | FALSE | FALSE | ebi-a-GCST90018120 | 19 | 46059364 | 0.000116744 | 437235 | 0.83 | Idiopathic pulmonary fibrosis \|\| id:ebi-a-GCST90018120 | Idiopathic pulmonary fibrosis | Idiopathic pulmonary fibrosis \|\| \|\| | TRUE | igd | 0.0024 | 1.78E-10 | exposure | TRUE | reported | MpDfko | textfile | 2 | TRUE |
|  | 54 | rs116805289 | A | C | A | C | 0.0601 | -0.000408247 | 0.023092635 | 0.977039 | FALSE | FALSE | FALSE | ebi-a-GCST90018120 | 1 | 154510155 | 0.000385804 | 437235 | 0.29 | Idiopathic pulmonary fibrosis \|\| id:ebi-a-GCST90018120 | Idiopathic pulmonary fibrosis | Idiopathic pulmonary fibrosis \|\| \|\| | TRUE | igd | 0.0081 | 1.03E-13 | exposure | TRUE | reported | MpDfko | textfile | 2 | TRUE |
|  | 55 | rs11682713 | T | C | T | C | -0.0322 | -7.48E-05 | 0.105913277 | 0.894766 | FALSE | FALSE | FALSE | ebi-a-GCST90018120 | 2 | 27491111 | 0.000188656 | 437235 | 0.69 | Idiopathic pulmonary fibrosis \|\| id:ebi-a-GCST90018120 | Idiopathic pulmonary fibrosis | Idiopathic pulmonary fibrosis \|\| \|\| | TRUE | igd | 0.0039 | 2.02E-16 | exposure | TRUE | reported | MpDfko | textfile | 2 | TRUE |
|  | 56 | rs116877397 | C | T | C | T | -0.0909 | 0.000281485 | 0.012059565 | 0.987927 | FALSE | FALSE | FALSE | ebi-a-GCST90018120 | 12 | 121162996 | 0.000529432 | 437235 | 0.6 | Idiopathic pulmonary fibrosis \|\| id:ebi-a-GCST90018120 | Idiopathic pulmonary fibrosis | Idiopathic pulmonary fibrosis \|\| \|\| | TRUE | igd | 0.0109 | 5.51E-17 | exposure | TRUE | reported | MpDfko | textfile | 2 | TRUE |
|  | 57 | rs116881341 | G | A | G | A | -0.0743 | 2.52E-05 | 0.018463807 | 0.98153 | FALSE | FALSE | FALSE | ebi-a-GCST90018120 | 12 | 120737796 | 0.000429241 | 437235 | 0.95 | Idiopathic pulmonary fibrosis \|\| id:ebi-a-GCST90018120 | Idiopathic pulmonary fibrosis | Idiopathic pulmonary fibrosis \|\| \|\| | TRUE | igd | 0.009 | 1.65E-16 | exposure | TRUE | reported | MpDfko | textfile | 2 | TRUE |
|  | 58 | rs1169282 | C | T | C | T | -0.1074 | 0.000176242 | 0.027675567 | 0.972898 | FALSE | FALSE | FALSE | ebi-a-GCST90018120 | 12 | 121420430 | 0.000356245 | 437235 | 0.62 | Idiopathic pulmonary fibrosis \|\| id:ebi-a-GCST90018120 | Idiopathic pulmonary fibrosis | Idiopathic pulmonary fibrosis \|\| \|\| | TRUE | igd | 0.0074 | 3.29E-47 | exposure | TRUE | reported | MpDfko | textfile | 2 | TRUE |
|  | 59 | rs1169721 | A | G | A | G | -0.0226 | -0.000158834 | 0.208958524 | 0.791144 | FALSE | FALSE | FALSE | ebi-a-GCST90018120 | 12 | 121641509 | 0.000142243 | 437235 | 0.26 | Idiopathic pulmonary fibrosis \|\| id:ebi-a-GCST90018120 | Idiopathic pulmonary fibrosis | Idiopathic pulmonary fibrosis \|\| \|\| | TRUE | igd | 0.0029 | 9.70E-15 | exposure | TRUE | reported | MpDfko | textfile | 2 | TRUE |
|  | 60 | rs117198034 | C | T | C | T | 0.074 | -0.000185601 | 0.022289958 | 0.978248 | FALSE | FALSE | FALSE | ebi-a-GCST90018120 | 19 | 45469902 | 0.000396084 | 437235 | 0.64 | Idiopathic pulmonary fibrosis \|\| id:ebi-a-GCST90018120 | Idiopathic pulmonary fibrosis | Idiopathic pulmonary fibrosis \|\| \|\| | TRUE | igd | 0.0083 | 3.13E-19 | exposure | TRUE | reported | MpDfko | textfile | 2 | TRUE |
|  | 61 | rs117264457 | G | A | G | A | 0.0944 | 0.000101238 | 0.025055015 | 0.975339 | FALSE | FALSE | FALSE | ebi-a-GCST90018120 | 19 | 45404432 | 0.000372793 | 437235 | 0.79 | Idiopathic pulmonary fibrosis \|\| id:ebi-a-GCST90018120 | Idiopathic pulmonary fibrosis | Idiopathic pulmonary fibrosis \|\| \|\| | TRUE | igd | 0.0078 | 5.80E-34 | exposure | TRUE | reported | MpDfko | textfile | 2 | TRUE |
|  | 62 | rs117310449 | C | T | C | T | -0.2624 | -0.000101315 | 0.012029524 | 0.987942 | FALSE | FALSE | FALSE | ebi-a-GCST90018120 | 19 | 45393516 | 0.00053302 | 437235 | 0.85 | Idiopathic pulmonary fibrosis \|\| id:ebi-a-GCST90018120 | Idiopathic pulmonary fibrosis | Idiopathic pulmonary fibrosis \|\| \|\| | TRUE | igd | 0.0117 | 3.81E-112 | exposure | TRUE | reported | MpDfko | textfile | 2 | TRUE |
|  | 63 | rs117326714 | A | G | A | G | 0.0494 | 6.89E-05 | 0.026542774 | 0.97359 | FALSE | FALSE | FALSE | ebi-a-GCST90018120 | 19 | 45287103 | 0.000360494 | 437235 | 0.85 | Idiopathic pulmonary fibrosis \|\| id:ebi-a-GCST90018120 | Idiopathic pulmonary fibrosis | Idiopathic pulmonary fibrosis \|\| \|\| | TRUE | igd | 0.0076 | 6.02E-11 | exposure | TRUE | reported | MpDfko | textfile | 2 | TRUE |
|  | 64 | rs117368206 | C | T | C | T | -0.0606 | 0.000470799 | 0.019873039 | 0.979834 | FALSE | FALSE | FALSE | ebi-a-GCST90018120 | 12 | 121028907 | 0.000410666 | 437235 | 0.25 | Idiopathic pulmonary fibrosis \|\| id:ebi-a-GCST90018120 | Idiopathic pulmonary fibrosis | Idiopathic pulmonary fibrosis \|\| \|\| | TRUE | igd | 0.0086 | 2.36E-12 | exposure | TRUE | reported | MpDfko | textfile | 2 | TRUE |
|  | 65 | rs117436012 | T | C | T | C | -0.0309 | -6.93E-05 | 0.056116629 | 0.943496 | FALSE | FALSE | FALSE | ebi-a-GCST90018120 | 8 | 9197359 | 0.000250341 | 437235 | 0.780001 | Idiopathic pulmonary fibrosis \|\| id:ebi-a-GCST90018120 | Idiopathic pulmonary fibrosis | Idiopathic pulmonary fibrosis \|\| \|\| | TRUE | igd | 0.0052 | 3.32E-09 | exposure | TRUE | reported | MpDfko | textfile | 2 | TRUE |
|  | 66 | rs117453824 | C | T | C | T | -0.0613 | -0.000337232 | 0.014860446 | 0.985175 | FALSE | FALSE | FALSE | ebi-a-GCST90018120 | 12 | 24223492 | 0.000477876 | 437235 | 0.48 | Idiopathic pulmonary fibrosis \|\| id:ebi-a-GCST90018120 | Idiopathic pulmonary fibrosis | Idiopathic pulmonary fibrosis \|\| \|\| | TRUE | igd | 0.0099 | 5.72E-10 | exposure | TRUE | reported | MpDfko | textfile | 2 | TRUE |
|  | 67 | rs117468623 | T | C | T | C | 0.0578 | -0.000503526 | 0.01732858 | 0.983026 | FALSE | FALSE | FALSE | ebi-a-GCST90018120 | 12 | 120688572 | 0.000447452 | 437235 | 0.26 | Idiopathic pulmonary fibrosis \|\| id:ebi-a-GCST90018120 | Idiopathic pulmonary fibrosis | Idiopathic pulmonary fibrosis \|\| \|\| | TRUE | igd | 0.0093 | 5.30E-10 | exposure | TRUE | reported | MpDfko | textfile | 2 | TRUE |
|  | 68 | rs117477328 | C | T | C | T | -0.0735 | 0.000524621 | 0.012522497 | 0.987248 | FALSE | FALSE | FALSE | ebi-a-GCST90018120 | 12 | 121273856 | 0.000515042 | 437235 | 0.31 | Idiopathic pulmonary fibrosis \|\| id:ebi-a-GCST90018120 | Idiopathic pulmonary fibrosis | Idiopathic pulmonary fibrosis \|\| \|\| | TRUE | igd | 0.0108 | 9.45E-12 | exposure | TRUE | reported | MpDfko | textfile | 2 | TRUE |
|  | 69 | rs11770879 | G | A | G | A | -0.0324 | -6.74E-05 | 0.065707259 | 0.934042 | FALSE | FALSE | FALSE | ebi-a-GCST90018120 | 7 | 23208043 | 0.000232724 | 437235 | 0.77 | Idiopathic pulmonary fibrosis \|\| id:ebi-a-GCST90018120 | Idiopathic pulmonary fibrosis | Idiopathic pulmonary fibrosis \|\| \|\| | TRUE | igd | 0.0049 | 3.76E-11 | exposure | TRUE | reported | MpDfko | textfile | 2 | TRUE |
|  | 70 | rs118106133 | C | T | C | T | -0.1095 | -0.000321274 | 0.023305821 | 0.97666 | FALSE | FALSE | FALSE | ebi-a-GCST90018120 | 16 | 51450938 | 0.000382316 | 437235 | 0.4 | Idiopathic pulmonary fibrosis \|\| id:ebi-a-GCST90018120 | Idiopathic pulmonary fibrosis | Idiopathic pulmonary fibrosis \|\| \|\| | TRUE | igd | 0.008 | 1.41E-42 | exposure | TRUE | reported | MpDfko | textfile | 2 | TRUE |
|  | 71 | rs12042284 | A | C | A | C | 0.0305 | -0.000135974 | 0.088417377 | 0.911433 | FALSE | FALSE | FALSE | ebi-a-GCST90018120 | 1 | 159132649 | 0.000203556 | 437235 | 0.5 | Idiopathic pulmonary fibrosis \|\| id:ebi-a-GCST90018120 | Idiopathic pulmonary fibrosis | Idiopathic pulmonary fibrosis \|\| \|\| | TRUE | igd | 0.0042 | 5.65E-13 | exposure | TRUE | reported | MpDfko | textfile | 2 | TRUE |
|  | 72 | rs12042349 | C | T | C | T | -0.0466 | 1.48E-05 | 0.096226163 | 0.903751 | FALSE | FALSE | FALSE | ebi-a-GCST90018120 | 1 | 159176490 | 0.000195776 | 437235 | 0.94 | Idiopathic pulmonary fibrosis \|\| id:ebi-a-GCST90018120 | Idiopathic pulmonary fibrosis | Idiopathic pulmonary fibrosis \|\| \|\| | TRUE | igd | 0.004 | 1.94E-31 | exposure | TRUE | reported | MpDfko | textfile | 2 | TRUE |
|  | 73 | rs12044132 | C | T | C | T | -0.0527 | 0.000130492 | 0.160510735 | 0.840479 | FALSE | FALSE | FALSE | ebi-a-GCST90018120 | 1 | 154462360 | 0.000157798 | 437235 | 0.41 | Idiopathic pulmonary fibrosis \|\| id:ebi-a-GCST90018120 | Idiopathic pulmonary fibrosis | Idiopathic pulmonary fibrosis \|\| \|\| | TRUE | igd | 0.0033 | 8.90E-59 | exposure | TRUE | reported | MpDfko | textfile | 2 | TRUE |
|  | 74 | rs12048215 | A | G | A | G | 0.0373 | -0.000236889 | 0.116356545 | 0.883837 | FALSE | FALSE | FALSE | ebi-a-GCST90018120 | 1 | 247584591 | 0.00018033 | 437235 | 0.19 | Idiopathic pulmonary fibrosis \|\| id:ebi-a-GCST90018120 | Idiopathic pulmonary fibrosis | Idiopathic pulmonary fibrosis \|\| \|\| | TRUE | igd | 0.0037 | 7.88E-24 | exposure | TRUE | reported | MpDfko | textfile | 2 | TRUE |
|  | 75 | rs12313817 | G | A | G | A | -0.047 | 0.000193348 | 0.038416646 | 0.961357 | FALSE | FALSE | FALSE | ebi-a-GCST90018120 | 12 | 24260884 | 0.000299574 | 437235 | 0.52 | Idiopathic pulmonary fibrosis \|\| id:ebi-a-GCST90018120 | Idiopathic pulmonary fibrosis | Idiopathic pulmonary fibrosis \|\| \|\| | TRUE | igd | 0.0061 | 8.73E-15 | exposure | TRUE | reported | MpDfko | textfile | 2 | TRUE |
|  | 76 | rs12328794 | T | C | T | C | 0.0168 | -0.000186569 | 0.488724634 | 0.511374 | FALSE | FALSE | FALSE | ebi-a-GCST90018120 | 2 | 169961897 | 0.000115731 | 437235 | 0.11 | Idiopathic pulmonary fibrosis \|\| id:ebi-a-GCST90018120 | Idiopathic pulmonary fibrosis | Idiopathic pulmonary fibrosis \|\| \|\| | TRUE | igd | 0.0024 | 1.92E-12 | exposure | TRUE | reported | MpDfko | textfile | 2 | TRUE |
|  | 77 | rs12421530 | C | G | C | G | 0.021 | 4.33E-05 | 0.38402725 | 0.384348 | FALSE | TRUE | FALSE | ebi-a-GCST90018120 | 11 | 13366722 | 0.000118875 | 437235 | 0.709999 | Idiopathic pulmonary fibrosis \|\| id:ebi-a-GCST90018120 | Idiopathic pulmonary fibrosis | Idiopathic pulmonary fibrosis \|\| \|\| | TRUE | igd | 0.0024 | 8.40E-18 | exposure | TRUE | reported | MpDfko | textfile | 2 | TRUE |
|  | 78 | rs12601655 | A | G | A | G | 0.0288 | -0.000115757 | 0.193763291 | 0.806055 | FALSE | FALSE | FALSE | ebi-a-GCST90018120 | 17 | 76371592 | 0.000146401 | 437235 | 0.43 | Idiopathic pulmonary fibrosis \|\| id:ebi-a-GCST90018120 | Idiopathic pulmonary fibrosis | Idiopathic pulmonary fibrosis \|\| \|\| | TRUE | igd | 0.003 | 1.01E-21 | exposure | TRUE | reported | MpDfko | textfile | 2 | TRUE |
|  | 79 | rs12610605 | G | A | G | A | 0.0414 | -2.93E-05 | 0.179574187 | 0.82043 | FALSE | FALSE | FALSE | ebi-a-GCST90018120 | 19 | 45370838 | 0.00015143 | 437235 | 0.85 | Idiopathic pulmonary fibrosis \|\| id:ebi-a-GCST90018120 | Idiopathic pulmonary fibrosis | Idiopathic pulmonary fibrosis \|\| \|\| | TRUE | igd | 0.0033 | 8.29E-37 | exposure | TRUE | reported | MpDfko | textfile | 2 | TRUE |
|  | 80 | rs12625630 | C | T | C | T | 0.0185 | 0.000251886 | 0.174355804 | 0.825543 | FALSE | FALSE | FALSE | ebi-a-GCST90018120 | 20 | 48984751 | 0.000152276 | 437235 | 0.0980009 | Idiopathic pulmonary fibrosis \|\| id:ebi-a-GCST90018120 | Idiopathic pulmonary fibrosis | Idiopathic pulmonary fibrosis \|\| \|\| | TRUE | igd | 0.0032 | 4.29E-09 | exposure | TRUE | reported | MpDfko | textfile | 2 | TRUE |
|  | 81 | rs12691088 | G | A | G | A | -0.2159 | -0.000620579 | 0.023730813 | 0.978894 | FALSE | FALSE | FALSE | ebi-a-GCST90018120 | 19 | 45418486 | 0.000431848 | 437235 | 0.15 | Idiopathic pulmonary fibrosis \|\| id:ebi-a-GCST90018120 | Idiopathic pulmonary fibrosis | Idiopathic pulmonary fibrosis \|\| \|\| | TRUE | igd | 0.0084 | 8.08E-145 | exposure | TRUE | reported | MpDfko | textfile | 2 | TRUE |
|  | 82 | rs12721063 | G | A | G | A | 0.0682 | -0.00033764 | 0.011039021 | 0.988987 | FALSE | FALSE | FALSE | ebi-a-GCST90018120 | 19 | 45449166 | 0.000553351 | 437235 | 0.54 | Idiopathic pulmonary fibrosis \|\| id:ebi-a-GCST90018120 | Idiopathic pulmonary fibrosis | Idiopathic pulmonary fibrosis \|\| \|\| | TRUE | igd | 0.0116 | 3.64E-09 | exposure | TRUE | reported | MpDfko | textfile | 2 | TRUE |
|  | 83 | rs12721109 | G | A | G | A | 0.0764 | -0.000394209 | 0.026031724 | 0.974191 | FALSE | FALSE | FALSE | ebi-a-GCST90018120 | 19 | 45447221 | 0.000364513 | 437235 | 0.28 | Idiopathic pulmonary fibrosis \|\| id:ebi-a-GCST90018120 | Idiopathic pulmonary fibrosis | Idiopathic pulmonary fibrosis \|\| \|\| | TRUE | igd | 0.0076 | 8.70E-24 | exposure | TRUE | reported | MpDfko | textfile | 2 | TRUE |
|  | 84 | rs12728998 | C | T | C | T | -0.0197 | -8.46E-06 | 0.172464835 | 0.82652 | FALSE | FALSE | FALSE | ebi-a-GCST90018120 | 1 | 247592175 | 0.000152766 | 437235 | 0.96 | Idiopathic pulmonary fibrosis \|\| id:ebi-a-GCST90018120 | Idiopathic pulmonary fibrosis | Idiopathic pulmonary fibrosis \|\| \|\| | TRUE | igd | 0.0031 | 3.84E-10 | exposure | TRUE | reported | MpDfko | textfile | 2 | TRUE |
|  | 85 | rs12745083 | C | T | C | T | 0.1132 | 1.71E-05 | 0.023184897 | 0.97676 | FALSE | FALSE | FALSE | ebi-a-GCST90018120 | 1 | 159550605 | 0.000383649 | 437235 | 0.97 | Idiopathic pulmonary fibrosis \|\| id:ebi-a-GCST90018120 | Idiopathic pulmonary fibrosis | Idiopathic pulmonary fibrosis \|\| \|\| | TRUE | igd | 0.0076 | 1.27E-50 | exposure | TRUE | reported | MpDfko | textfile | 2 | TRUE |
|  | 86 | rs12816397 | A | G | A | G | 0.075 | 0.000170336 | 0.053093304 | 0.947122 | FALSE | FALSE | FALSE | ebi-a-GCST90018120 | 12 | 121271706 | 0.000258264 | 437235 | 0.51 | Idiopathic pulmonary fibrosis \|\| id:ebi-a-GCST90018120 | Idiopathic pulmonary fibrosis | Idiopathic pulmonary fibrosis \|\| \|\| | TRUE | igd | 0.0054 | 1.60E-43 | exposure | TRUE | reported | MpDfko | textfile | 2 | TRUE |
|  | 87 | rs12819210 | C | T | C | T | 0.0596 | -0.000136049 | 0.18720866 | 0.812228 | FALSE | FALSE | FALSE | ebi-a-GCST90018120 | 12 | 121458400 | 0.000148051 | 437235 | 0.36 | Idiopathic pulmonary fibrosis \|\| id:ebi-a-GCST90018120 | Idiopathic pulmonary fibrosis | Idiopathic pulmonary fibrosis \|\| \|\| | TRUE | igd | 0.0031 | 4.79E-84 | exposure | TRUE | reported | MpDfko | textfile | 2 | TRUE |
|  | 88 | rs12823525 | C | G | C | G | -0.0164 | -9.50E-05 | 0.472957609 | 0.472824 | FALSE | TRUE | TRUE | ebi-a-GCST90018120 | 12 | 95930681 | 0.000115756 | 437235 | 0.41 | Idiopathic pulmonary fibrosis \|\| id:ebi-a-GCST90018120 | Idiopathic pulmonary fibrosis | Idiopathic pulmonary fibrosis \|\| \|\| | TRUE | igd | 0.0024 | 6.22E-12 | exposure | TRUE | reported | MpDfko | textfile | 2 | FALSE |
|  | 89 | rs12941913 | C | T | C | T | 0.0155 | 0.000180982 | 0.399204 | 0.392584 | FALSE | FALSE | FALSE | ebi-a-GCST90018120 | 17 | 1346417 | 0.000118629 | 437235 | 0.13 | Idiopathic pulmonary fibrosis \|\| id:ebi-a-GCST90018120 | Idiopathic pulmonary fibrosis | Idiopathic pulmonary fibrosis \|\| \|\| | TRUE | igd | 0.0024 | 2.02E-10 | exposure | TRUE | reported | MpDfko | textfile | 2 | TRUE |
|  | 90 | rs13016086 | T | C | T | C | -0.0302 | -0.00021379 | 0.119129384 | 0.882006 | FALSE | FALSE | FALSE | ebi-a-GCST90018120 | 2 | 28315770 | 0.000179515 | 437235 | 0.23 | Idiopathic pulmonary fibrosis \|\| id:ebi-a-GCST90018120 | Idiopathic pulmonary fibrosis | Idiopathic pulmonary fibrosis \|\| \|\| | TRUE | igd | 0.0038 | 1.00E-15 | exposure | TRUE | reported | MpDfko | textfile | 2 | TRUE |
|  | 91 | rs13266821 | C | T | C | T | -0.0209 | -0.000417158 | 0.147763149 | 0.852395 | FALSE | FALSE | FALSE | ebi-a-GCST90018120 | 8 | 144645513 | 0.000162757 | 437235 | 0.01 | Idiopathic pulmonary fibrosis \|\| id:ebi-a-GCST90018120 | Idiopathic pulmonary fibrosis | Idiopathic pulmonary fibrosis \|\| \|\| | TRUE | igd | 0.0034 | 4.42E-10 | exposure | TRUE | reported | MpDfko | textfile | 2 | TRUE |
|  | 92 | rs13279515 | C | T | C | T | -0.0314 | -2.36E-05 | 0.139982114 | 0.859221 | FALSE | FALSE | FALSE | ebi-a-GCST90018120 | 8 | 9221327 | 0.000166293 | 437235 | 0.89 | Idiopathic pulmonary fibrosis \|\| id:ebi-a-GCST90018120 | Idiopathic pulmonary fibrosis | Idiopathic pulmonary fibrosis \|\| \|\| | TRUE | igd | 0.0035 | 2.44E-19 | exposure | TRUE | reported | MpDfko | textfile | 2 | TRUE |
|  | 93 | rs13374371 | T | G | T | G | 0.0259 | -0.000166575 | 0.122418673 | 0.877963 | FALSE | FALSE | FALSE | ebi-a-GCST90018120 | 1 | 65696189 | 0.000176516 | 437235 | 0.35 | Idiopathic pulmonary fibrosis \|\| id:ebi-a-GCST90018120 | Idiopathic pulmonary fibrosis | Idiopathic pulmonary fibrosis \|\| \|\| | TRUE | igd | 0.0036 | 1.01E-12 | exposure | TRUE | reported | MpDfko | textfile | 2 | TRUE |
|  | 94 | rs1371614 | C | T | C | T | -0.0227 | 3.32E-05 | 0.245265177 | 0.754344 | FALSE | FALSE | FALSE | ebi-a-GCST90018120 | 2 | 27152874 | 0.000134311 | 437235 | 0.8 | Idiopathic pulmonary fibrosis \|\| id:ebi-a-GCST90018120 | Idiopathic pulmonary fibrosis | Idiopathic pulmonary fibrosis \|\| \|\| | TRUE | igd | 0.0028 | 2.00E-16 | exposure | TRUE | reported | MpDfko | textfile | 2 | TRUE |
|  | 95 | rs138607350 | T | G | T | G | -0.1783 | 0.00154607 | 0.009591095 | 0.991534 | FALSE | FALSE | FALSE | ebi-a-GCST90018120 | 19 | 45363820 | 0.000661342 | 437235 | 0.0189998 | Idiopathic pulmonary fibrosis \|\| id:ebi-a-GCST90018120 | Idiopathic pulmonary fibrosis | Idiopathic pulmonary fibrosis \|\| \|\| | TRUE | igd | 0.0132 | 8.06E-42 | exposure | TRUE | reported | MpDfko | textfile | 2 | TRUE |
|  | 96 | rs1386821 | T | G | T | G | -0.0619 | 7.34E-05 | 0.204518165 | 0.795221 | FALSE | FALSE | FALSE | ebi-a-GCST90018120 | 1 | 154382049 | 0.000143063 | 437235 | 0.61 | Idiopathic pulmonary fibrosis \|\| id:ebi-a-GCST90018120 | Idiopathic pulmonary fibrosis | Idiopathic pulmonary fibrosis \|\| \|\| | TRUE | igd | 0.003 | 4.00E-97 | exposure | TRUE | reported | MpDfko | textfile | 2 | TRUE |
|  | 97 | rs138914864 | C | T | C | T | -0.2652 | 0.000867646 | 0.002920615 | 0.99743 | FALSE | FALSE | FALSE | ebi-a-GCST90018120 | 19 | 45379431 | 0.00124541 | 437235 | 0.48 | Idiopathic pulmonary fibrosis \|\| id:ebi-a-GCST90018120 | Idiopathic pulmonary fibrosis | Idiopathic pulmonary fibrosis \|\| \|\| | TRUE | igd | 0.0238 | 8.44E-29 | exposure | TRUE | reported | MpDfko | textfile | 2 | TRUE |
|  | 98 | rs140526515 | A | G | A | G | -0.2805 | 0.00093828 | 0.002827542 | 0.997321 | FALSE | FALSE | FALSE | ebi-a-GCST90018120 | 19 | 45427540 | 0.00120232 | 437235 | 0.43 | Idiopathic pulmonary fibrosis \|\| id:ebi-a-GCST90018120 | Idiopathic pulmonary fibrosis | Idiopathic pulmonary fibrosis \|\| \|\| | TRUE | igd | 0.0242 | 5.71E-31 | exposure | TRUE | reported | MpDfko | textfile | 2 | TRUE |
|  | 99 | rs141622900 | G | A | G | A | 0.0921 | 0.000294439 | 0.052238408 | 0.947618 | FALSE | FALSE | FALSE | ebi-a-GCST90018120 | 19 | 45426792 | 0.000265845 | 437235 | 0.27 | Idiopathic pulmonary fibrosis \|\| id:ebi-a-GCST90018120 | Idiopathic pulmonary fibrosis | Idiopathic pulmonary fibrosis \|\| \|\| | TRUE | igd | 0.0056 | 8.62E-61 | exposure | TRUE | reported | MpDfko | textfile | 2 | TRUE |
|  | 100 | rs141739979 | G | T | G | T | 0.0787 | -0.00137555 | 0.010639255 | 0.990392 | FALSE | FALSE | FALSE | ebi-a-GCST90018120 | 19 | 45374983 | 0.000638914 | 437235 | 0.032 | Idiopathic pulmonary fibrosis \|\| id:ebi-a-GCST90018120 | Idiopathic pulmonary fibrosis | Idiopathic pulmonary fibrosis \|\| \|\| | TRUE | igd | 0.0124 | 2.45E-10 | exposure | TRUE | reported | MpDfko | textfile | 2 | TRUE |
|  | 101 | rs142042446 | G | GTAA | G | GTAA | -0.2062 | 0.000115903 | 0.14872175 | 0.851348 | FALSE | FALSE | FALSE | ebi-a-GCST90018120 | 19 | 45386467 | 0.000164998 | 437235 | 0.48 | Idiopathic pulmonary fibrosis \|\| id:ebi-a-GCST90018120 | Idiopathic pulmonary fibrosis | Idiopathic pulmonary fibrosis \|\| \|\| | TRUE | igd | 0.0035 | 1.00E-200 | exposure | TRUE | reported | MpDfko | textfile | 2 | TRUE |
|  | 102 | rs144261139 | C | A | C | A | -0.2545 | -0.00023293 | 0.012186018 | 0.987849 | FALSE | FALSE | FALSE | ebi-a-GCST90018120 | 19 | 45385356 | 0.00053359 | 437235 | 0.66 | Idiopathic pulmonary fibrosis \|\| id:ebi-a-GCST90018120 | Idiopathic pulmonary fibrosis | Idiopathic pulmonary fibrosis \|\| \|\| | TRUE | igd | 0.0114 | 9.51E-110 | exposure | TRUE | reported | MpDfko | textfile | 2 | TRUE |
|  | 103 | rs147707133 | C | G | C | G | 0.0903 | 0.000313269 | 0.017150515 | 0.017027 | FALSE | TRUE | FALSE | ebi-a-GCST90018120 | 19 | 45399165 | 0.000473001 | 437235 | 0.51 | Idiopathic pulmonary fibrosis \|\| id:ebi-a-GCST90018120 | Idiopathic pulmonary fibrosis | Idiopathic pulmonary fibrosis \|\| \|\| | TRUE | igd | 0.0098 | 3.57E-20 | exposure | TRUE | reported | MpDfko | textfile | 2 | TRUE |
|  | 104 | rs150639620 | G | T | G | T | 0.0597 | 6.85E-05 | 0.050161952 | 0.948392 | FALSE | FALSE | FALSE | ebi-a-GCST90018120 | 19 | 45381292 | 0.000263416 | 437235 | 0.79 | Idiopathic pulmonary fibrosis \|\| id:ebi-a-GCST90018120 | Idiopathic pulmonary fibrosis | Idiopathic pulmonary fibrosis \|\| \|\| | TRUE | igd | 0.0057 | 6.53E-26 | exposure | TRUE | reported | MpDfko | textfile | 2 | TRUE |
|  | 105 | rs151291132 | A | G | A | G | 0.074 | 1.97E-05 | 0.025991952 | 0.974723 | FALSE | FALSE | FALSE | ebi-a-GCST90018120 | 15 | 44842210 | 0.000376538 | 437235 | 0.96 | Idiopathic pulmonary fibrosis \|\| id:ebi-a-GCST90018120 | Idiopathic pulmonary fibrosis | Idiopathic pulmonary fibrosis \|\| \|\| | TRUE | igd | 0.008 | 1.60E-20 | exposure | TRUE | reported | MpDfko | textfile | 2 | TRUE |
|  | 106 | rs1524107 | C | T | C | T | -0.0528 | 0.000135996 | 0.043352572 | 0.955829 | FALSE | FALSE | FALSE | ebi-a-GCST90018120 | 7 | 22768219 | 0.000282145 | 437235 | 0.630001 | Idiopathic pulmonary fibrosis \|\| id:ebi-a-GCST90018120 | Idiopathic pulmonary fibrosis | Idiopathic pulmonary fibrosis \|\| \|\| | TRUE | igd | 0.0058 | 6.80E-20 | exposure | TRUE | reported | MpDfko | textfile | 2 | TRUE |
|  | 107 | rs164910 | T | C | T | C | -0.0284 | 0.000517264 | 0.085319954 | 0.914518 | FALSE | FALSE | FALSE | ebi-a-GCST90018120 | 1 | 91550535 | 0.000206979 | 437235 | 0.012 | Idiopathic pulmonary fibrosis \|\| id:ebi-a-GCST90018120 | Idiopathic pulmonary fibrosis | Idiopathic pulmonary fibrosis \|\| \|\| | TRUE | igd | 0.0042 | 1.52E-11 | exposure | TRUE | reported | MpDfko | textfile | 2 | TRUE |
|  | 108 | rs1653607 | C | T | C | T | 0.0302 | 0.00017188 | 0.108220181 | 0.107653 | FALSE | FALSE | FALSE | ebi-a-GCST90018120 | 12 | 121707036 | 0.000186728 | 437235 | 0.36 | Idiopathic pulmonary fibrosis \|\| id:ebi-a-GCST90018120 | Idiopathic pulmonary fibrosis | Idiopathic pulmonary fibrosis \|\| \|\| | TRUE | igd | 0.0039 | 1.21E-14 | exposure | TRUE | reported | MpDfko | textfile | 2 | TRUE |
|  | 109 | rs16835630 | T | C | T | C | -0.0759 | 6.84E-05 | 0.017787253 | 0.982202 | FALSE | FALSE | FALSE | ebi-a-GCST90018120 | 1 | 153824852 | 0.000437344 | 437235 | 0.87 | Idiopathic pulmonary fibrosis \|\| id:ebi-a-GCST90018120 | Idiopathic pulmonary fibrosis | Idiopathic pulmonary fibrosis \|\| \|\| | TRUE | igd | 0.0091 | 9.06E-17 | exposure | TRUE | reported | MpDfko | textfile | 2 | TRUE |
|  | 110 | rs16950101 | A | G | A | G | -0.0442 | -0.000287272 | 0.056923795 | 0.943829 | FALSE | FALSE | FALSE | ebi-a-GCST90018120 | 12 | 120815504 | 0.000251077 | 437235 | 0.25 | Idiopathic pulmonary fibrosis \|\| id:ebi-a-GCST90018120 | Idiopathic pulmonary fibrosis | Idiopathic pulmonary fibrosis \|\| \|\| | TRUE | igd | 0.0052 | 1.09E-17 | exposure | TRUE | reported | MpDfko | textfile | 2 | TRUE |
|  | 111 | rs17097193 | T | C | T | C | 0.0468 | 0.000556192 | 0.031035254 | 0.968581 | FALSE | FALSE | FALSE | ebi-a-GCST90018120 | 1 | 66067396 | 0.00033147 | 437235 | 0.0929994 | Idiopathic pulmonary fibrosis \|\| id:ebi-a-GCST90018120 | Idiopathic pulmonary fibrosis | Idiopathic pulmonary fibrosis \|\| \|\| | TRUE | igd | 0.0067 | 2.48E-12 | exposure | TRUE | reported | MpDfko | textfile | 2 | TRUE |
|  | 112 | rs17128076 | G | A | G | A | -0.046 | 0.000182604 | 0.038620884 | 0.961905 | FALSE | FALSE | FALSE | ebi-a-GCST90018120 | 1 | 66332054 | 0.00030203 | 437235 | 0.55 | Idiopathic pulmonary fibrosis \|\| id:ebi-a-GCST90018120 | Idiopathic pulmonary fibrosis | Idiopathic pulmonary fibrosis \|\| \|\| | TRUE | igd | 0.0062 | 9.28E-14 | exposure | TRUE | reported | MpDfko | textfile | 2 | TRUE |
|  | 113 | rs17138478 | C | A | C | A | 0.0329 | 0.000131051 | 0.128551168 | 0.871085 | FALSE | FALSE | FALSE | ebi-a-GCST90018120 | 17 | 36073320 | 0.000172617 | 437235 | 0.450001 | Idiopathic pulmonary fibrosis \|\| id:ebi-a-GCST90018120 | Idiopathic pulmonary fibrosis | Idiopathic pulmonary fibrosis \|\| \|\| | TRUE | igd | 0.0036 | 2.69E-20 | exposure | TRUE | reported | MpDfko | textfile | 2 | TRUE |
|  | 114 | rs17216525 | C | T | C | T | 0.035 | 0.000386357 | 0.0813804 | 0.919291 | FALSE | FALSE | FALSE | ebi-a-GCST90018120 | 19 | 19662220 | 0.000212544 | 437235 | 0.0690001 | Idiopathic pulmonary fibrosis \|\| id:ebi-a-GCST90018120 | Idiopathic pulmonary fibrosis | Idiopathic pulmonary fibrosis \|\| \|\| | TRUE | igd | 0.0044 | 9.63E-16 | exposure | TRUE | reported | MpDfko | textfile | 2 | TRUE |
|  | 115 | rs17408658 | A | C | A | C | -0.0308 | -0.000145384 | 0.320575515 | 0.679033 | FALSE | FALSE | FALSE | ebi-a-GCST90018120 | 1 | 66228851 | 0.000123762 | 437235 | 0.24 | Idiopathic pulmonary fibrosis \|\| id:ebi-a-GCST90018120 | Idiopathic pulmonary fibrosis | Idiopathic pulmonary fibrosis \|\| \|\| | TRUE | igd | 0.0026 | 3.48E-33 | exposure | TRUE | reported | MpDfko | textfile | 2 | TRUE |
|  | 116 | rs17434647 | T | G | T | G | 0.0565 | 0.000241481 | 0.123075987 | 0.877108 | FALSE | FALSE | FALSE | ebi-a-GCST90018120 | 12 | 121579024 | 0.00017602 | 437235 | 0.17 | Idiopathic pulmonary fibrosis \|\| id:ebi-a-GCST90018120 | Idiopathic pulmonary fibrosis | Idiopathic pulmonary fibrosis \|\| \|\| | TRUE | igd | 0.0037 | 9.92E-54 | exposure | TRUE | reported | MpDfko | textfile | 2 | TRUE |
|  | 117 | rs17457976 | A | G | A | G | 0.0593 | 9.18E-05 | 0.15156629 | 0.848342 | FALSE | FALSE | FALSE | ebi-a-GCST90018120 | 1 | 159580873 | 0.000161265 | 437235 | 0.57 | Idiopathic pulmonary fibrosis \|\| id:ebi-a-GCST90018120 | Idiopathic pulmonary fibrosis | Idiopathic pulmonary fibrosis \|\| \|\| | TRUE | igd | 0.0034 | 1.99E-69 | exposure | TRUE | reported | MpDfko | textfile | 2 | TRUE |
|  | 118 | rs17459069 | C | T | C | T | -0.117 | -0.000306685 | 0.03987762 | 0.9598 | FALSE | FALSE | FALSE | ebi-a-GCST90018120 | 1 | 159648693 | 0.00029431 | 437235 | 0.3 | Idiopathic pulmonary fibrosis \|\| id:ebi-a-GCST90018120 | Idiopathic pulmonary fibrosis | Idiopathic pulmonary fibrosis \|\| \|\| | TRUE | igd | 0.0062 | 1.57E-80 | exposure | TRUE | reported | MpDfko | textfile | 2 | TRUE |
|  | 119 | rs17616063 | A | G | A | G | -0.1287 | 0.000109992 | 0.076133871 | 0.924671 | FALSE | FALSE | FALSE | ebi-a-GCST90018120 | 16 | 51436882 | 0.000219246 | 437235 | 0.61 | Idiopathic pulmonary fibrosis \|\| id:ebi-a-GCST90018120 | Idiopathic pulmonary fibrosis | Idiopathic pulmonary fibrosis \|\| \|\| | TRUE | igd | 0.0046 | 7.72E-175 | exposure | TRUE | reported | MpDfko | textfile | 2 | TRUE |
|  | 120 | rs17658057 | G | A | G | A | 0.0381 | -0.000198703 | 0.044906313 | 0.95565 | FALSE | FALSE | FALSE | ebi-a-GCST90018120 | 5 | 172175327 | 0.000280604 | 437235 | 0.48 | Idiopathic pulmonary fibrosis \|\| id:ebi-a-GCST90018120 | Idiopathic pulmonary fibrosis | Idiopathic pulmonary fibrosis \|\| \|\| | TRUE | igd | 0.0059 | 7.75E-11 | exposure | TRUE | reported | MpDfko | textfile | 2 | TRUE |
|  | 121 | rs17796841 | T | C | T | C | -0.0345 | -9.77E-05 | 0.06487806 | 0.935595 | FALSE | FALSE | FALSE | ebi-a-GCST90018120 | 7 | 23353514 | 0.000235324 | 437235 | 0.68 | Idiopathic pulmonary fibrosis \|\| id:ebi-a-GCST90018120 | Idiopathic pulmonary fibrosis | Idiopathic pulmonary fibrosis \|\| \|\| | TRUE | igd | 0.0049 | 2.20E-12 | exposure | TRUE | reported | MpDfko | textfile | 2 | TRUE |
|  | 122 | rs17817449 | T | G | T | G | 0.021 | 4.38E-05 | 0.393743945 | 0.605274 | FALSE | FALSE | FALSE | ebi-a-GCST90018120 | 16 | 53813367 | 0.000118289 | 437235 | 0.709999 | Idiopathic pulmonary fibrosis \|\| id:ebi-a-GCST90018120 | Idiopathic pulmonary fibrosis | Idiopathic pulmonary fibrosis \|\| \|\| | TRUE | igd | 0.0024 | 7.34E-18 | exposure | TRUE | reported | MpDfko | textfile | 2 | TRUE |
|  | 123 | rs1855981 | G | C | G | C | -0.0159 | 1.38E-06 | 0.27014881 | 0.270365 | FALSE | TRUE | FALSE | ebi-a-GCST90018120 | 1 | 65696034 | 0.000130167 | 437235 | 0.99 | Idiopathic pulmonary fibrosis \|\| id:ebi-a-GCST90018120 | Idiopathic pulmonary fibrosis | Idiopathic pulmonary fibrosis \|\| \|\| | TRUE | igd | 0.0027 | 2.10E-09 | exposure | TRUE | reported | MpDfko | textfile | 2 | TRUE |
|  | 124 | rs187353341 | C | T | C | T | -0.0637 | 0.000571504 | 0.019259966 | 0.980766 | FALSE | FALSE | FALSE | ebi-a-GCST90018120 | 12 | 121557376 | 0.000421218 | 437235 | 0.17 | Idiopathic pulmonary fibrosis \|\| id:ebi-a-GCST90018120 | Idiopathic pulmonary fibrosis | Idiopathic pulmonary fibrosis \|\| \|\| | TRUE | igd | 0.0088 | 5.75E-13 | exposure | TRUE | reported | MpDfko | textfile | 2 | TRUE |
|  | 125 | rs191977866 | T | C | T | C | 0.0725 | 0.000686923 | 0.01926792 | 0.980778 | FALSE | FALSE | FALSE | ebi-a-GCST90018120 | 12 | 121123420 | 0.000420866 | 437235 | 0.1 | Idiopathic pulmonary fibrosis \|\| id:ebi-a-GCST90018120 | Idiopathic pulmonary fibrosis | Idiopathic pulmonary fibrosis \|\| \|\| | TRUE | igd | 0.0088 | 1.75E-16 | exposure | TRUE | reported | MpDfko | textfile | 2 | TRUE |
|  | 126 | rs201053423 | CAG | C | CAG | C | 0.0324 | 0.000174622 | 0.060106814 | 0.940269 | FALSE | FALSE | FALSE | ebi-a-GCST90018120 | 1 | 247611659 | 0.000243663 | 437235 | 0.47 | Idiopathic pulmonary fibrosis \|\| id:ebi-a-GCST90018120 | Idiopathic pulmonary fibrosis | Idiopathic pulmonary fibrosis \|\| \|\| | TRUE | igd | 0.005 | 1.03E-10 | exposure | TRUE | reported | MpDfko | textfile | 2 | TRUE |
|  | 127 | rs202228845 | AAAAAT | A | AAAAAT | A | -0.0151 | 0.000118141 | 0.375369 | 0.374349 | FALSE | FALSE | FALSE | ebi-a-GCST90018120 | 10 | 62410141 | 0.000122901 | 437235 | 0.34 | Idiopathic pulmonary fibrosis \|\| id:ebi-a-GCST90018120 | Idiopathic pulmonary fibrosis | Idiopathic pulmonary fibrosis \|\| \|\| | TRUE | igd | 0.0025 | 3.39E-09 | exposure | TRUE | reported | MpDfko | textfile | 2 | TRUE |
|  | 128 | rs2049045 | G | C | G | C | -0.0209 | -0.000161192 | 0.187838332 | 0.186679 | FALSE | TRUE | FALSE | ebi-a-GCST90018120 | 11 | 27694241 | 0.000148389 | 437235 | 0.28 | Idiopathic pulmonary fibrosis \|\| id:ebi-a-GCST90018120 | Idiopathic pulmonary fibrosis | Idiopathic pulmonary fibrosis \|\| \|\| | TRUE | igd | 0.0031 | 1.50E-11 | exposure | TRUE | reported | MpDfko | textfile | 2 | TRUE |
|  | 129 | rs204906 | C | T | C | T | 0.0619 | 9.36E-05 | 0.032799514 | 0.96686 | FALSE | FALSE | FALSE | ebi-a-GCST90018120 | 19 | 45461980 | 0.000323128 | 437235 | 0.77 | Idiopathic pulmonary fibrosis \|\| id:ebi-a-GCST90018120 | Idiopathic pulmonary fibrosis | Idiopathic pulmonary fibrosis \|\| \|\| | TRUE | igd | 0.0067 | 2.49E-20 | exposure | TRUE | reported | MpDfko | textfile | 2 | TRUE |
|  | 130 | rs2110944 | T | C | T | C | 0.0147 | 2.00E-05 | 0.481339 | 0.469445 | FALSE | FALSE | FALSE | ebi-a-GCST90018120 | 2 | 37090233 | 0.000116266 | 437235 | 0.86 | Idiopathic pulmonary fibrosis \|\| id:ebi-a-GCST90018120 | Idiopathic pulmonary fibrosis | Idiopathic pulmonary fibrosis \|\| \|\| | TRUE | igd | 0.0024 | 8.31E-10 | exposure | TRUE | reported | MpDfko | textfile | 2 | TRUE |
|  | 131 | rs2111125 | T | C | T | C | -0.0394 | 0.000171315 | 0.350518899 | 0.351344 | FALSE | FALSE | FALSE | ebi-a-GCST90018120 | 16 | 51165891 | 0.000121067 | 437235 | 0.16 | Idiopathic pulmonary fibrosis \|\| id:ebi-a-GCST90018120 | Idiopathic pulmonary fibrosis | Idiopathic pulmonary fibrosis \|\| \|\| | TRUE | igd | 0.0025 | 1.47E-55 | exposure | TRUE | reported | MpDfko | textfile | 2 | TRUE |
|  | 132 | rs2207132 | G | A | G | A | 0.044 | -7.96E-05 | 0.033101573 | 0.966821 | FALSE | FALSE | FALSE | ebi-a-GCST90018120 | 20 | 39142516 | 0.000322646 | 437235 | 0.81 | Idiopathic pulmonary fibrosis \|\| id:ebi-a-GCST90018120 | Idiopathic pulmonary fibrosis | Idiopathic pulmonary fibrosis \|\| \|\| | TRUE | igd | 0.0067 | 6.63E-11 | exposure | TRUE | reported | MpDfko | textfile | 2 | TRUE |
|  | 133 | rs2212339 | G | A | G | A | -0.0248 | 0.000234792 | 0.12964955 | 0.87041 | FALSE | FALSE | FALSE | ebi-a-GCST90018120 | 11 | 55194527 | 0.000172139 | 437235 | 0.17 | Idiopathic pulmonary fibrosis \|\| id:ebi-a-GCST90018120 | Idiopathic pulmonary fibrosis | Idiopathic pulmonary fibrosis \|\| \|\| | TRUE | igd | 0.0036 | 1.05E-11 | exposure | TRUE | reported | MpDfko | textfile | 2 | TRUE |
|  | 134 | rs2235780 | C | G | C | G | -0.0715 | 0.000887144 | 0.030389096 | 0.030629 | FALSE | TRUE | FALSE | ebi-a-GCST90018120 | 1 | 159168617 | 0.000335609 | 437235 | 0.00819993 | Idiopathic pulmonary fibrosis \|\| id:ebi-a-GCST90018120 | Idiopathic pulmonary fibrosis | Idiopathic pulmonary fibrosis \|\| \|\| | TRUE | igd | 0.007 | 2.62E-24 | exposure | TRUE | reported | MpDfko | textfile | 2 | TRUE |
|  | 135 | rs2244608 | A | G | A | G | -0.1388 | 8.87E-05 | 0.315270643 | 0.682902 | FALSE | FALSE | FALSE | ebi-a-GCST90018120 | 12 | 121416988 | 0.000124305 | 437235 | 0.48 | Idiopathic pulmonary fibrosis \|\| id:ebi-a-GCST90018120 | Idiopathic pulmonary fibrosis | Idiopathic pulmonary fibrosis \|\| \|\| | TRUE | igd | 0.0026 | 1.00E-200 | exposure | TRUE | reported | MpDfko | textfile | 2 | TRUE |
|  | 136 | rs2245407 | C | A | C | A | -0.0602 | -0.000258255 | 0.070775137 | 0.929637 | FALSE | FALSE | FALSE | ebi-a-GCST90018120 | 12 | 121423998 | 0.000226025 | 437235 | 0.25 | Idiopathic pulmonary fibrosis \|\| id:ebi-a-GCST90018120 | Idiopathic pulmonary fibrosis | Idiopathic pulmonary fibrosis \|\| \|\| | TRUE | igd | 0.0045 | 3.69E-40 | exposure | TRUE | reported | MpDfko | textfile | 2 | TRUE |
|  | 137 | rs2269434 | T | C | T | C | -0.0243 | 0.000131593 | 0.346475926 | 0.654014 | FALSE | FALSE | FALSE | ebi-a-GCST90018120 | 11 | 47360412 | 0.000121417 | 437235 | 0.28 | Idiopathic pulmonary fibrosis \|\| id:ebi-a-GCST90018120 | Idiopathic pulmonary fibrosis | Idiopathic pulmonary fibrosis \|\| \|\| | TRUE | igd | 0.0025 | 2.86E-22 | exposure | TRUE | reported | MpDfko | textfile | 2 | TRUE |
|  | 138 | rs2269841 | A | G | A | G | 0.0233 | -0.000173276 | 0.281872975 | 0.718775 | FALSE | FALSE | FALSE | ebi-a-GCST90018120 | 17 | 36059377 | 0.000128726 | 437235 | 0.18 | Idiopathic pulmonary fibrosis \|\| id:ebi-a-GCST90018120 | Idiopathic pulmonary fibrosis | Idiopathic pulmonary fibrosis \|\| \|\| | TRUE | igd | 0.0026 | 9.55E-19 | exposure | TRUE | reported | MpDfko | textfile | 2 | TRUE |
|  | 139 | rs2280405 | G | C | G | C | -0.0154 | -3.98E-05 | 0.465627246 | 0.465673 | FALSE | TRUE | TRUE | ebi-a-GCST90018120 | 3 | 49895052 | 0.000115815 | 437235 | 0.73 | Idiopathic pulmonary fibrosis \|\| id:ebi-a-GCST90018120 | Idiopathic pulmonary fibrosis | Idiopathic pulmonary fibrosis \|\| \|\| | TRUE | igd | 0.0024 | 9.50E-11 | exposure | TRUE | reported | MpDfko | textfile | 2 | FALSE |
|  | 140 | rs2526353 | G | T | G | T | -0.021 | -0.000183344 | 0.198501228 | 0.80277 | FALSE | FALSE | FALSE | ebi-a-GCST90018120 | 17 | 57988607 | 0.000145103 | 437235 | 0.2 | Idiopathic pulmonary fibrosis \|\| id:ebi-a-GCST90018120 | Idiopathic pulmonary fibrosis | Idiopathic pulmonary fibrosis \|\| \|\| | TRUE | igd | 0.003 | 1.97E-12 | exposure | TRUE | reported | MpDfko | textfile | 2 | TRUE |
|  | 141 | rs2686340 | T | C | T | C | 0.0257 | 0.000148747 | 0.197958098 | 0.198137 | FALSE | FALSE | FALSE | ebi-a-GCST90018120 | 12 | 121682051 | 0.000145051 | 437235 | 0.31 | Idiopathic pulmonary fibrosis \|\| id:ebi-a-GCST90018120 | Idiopathic pulmonary fibrosis | Idiopathic pulmonary fibrosis \|\| \|\| | TRUE | igd | 0.003 | 6.25E-18 | exposure | TRUE | reported | MpDfko | textfile | 2 | TRUE |
|  | 142 | rs2722733 | T | G | T | G | 0.0176 | 0.000156049 | 0.237290641 | 0.23747 | FALSE | FALSE | FALSE | ebi-a-GCST90018120 | 19 | 44845759 | 0.000135765 | 437235 | 0.25 | Idiopathic pulmonary fibrosis \|\| id:ebi-a-GCST90018120 | Idiopathic pulmonary fibrosis | Idiopathic pulmonary fibrosis \|\| \|\| | TRUE | igd | 0.0028 | 5.44E-10 | exposure | TRUE | reported | MpDfko | textfile | 2 | TRUE |
|  | 143 | rs2836878 | G | A | G | A | -0.0324 | 1.58E-05 | 0.267162371 | 0.733237 | FALSE | FALSE | FALSE | ebi-a-GCST90018120 | 21 | 40465534 | 0.000130705 | 437235 | 0.9 | Idiopathic pulmonary fibrosis \|\| id:ebi-a-GCST90018120 | Idiopathic pulmonary fibrosis | Idiopathic pulmonary fibrosis \|\| \|\| | TRUE | igd | 0.0027 | 5.43E-33 | exposure | TRUE | reported | MpDfko | textfile | 2 | TRUE |
|  | 144 | rs283814 | A | G | A | G | -0.0481 | 8.44E-05 | 0.07802237 | 0.078319 | FALSE | FALSE | FALSE | ebi-a-GCST90018120 | 19 | 45389224 | 0.000215097 | 437235 | 0.7 | Idiopathic pulmonary fibrosis \|\| id:ebi-a-GCST90018120 | Idiopathic pulmonary fibrosis | Idiopathic pulmonary fibrosis \|\| \|\| | TRUE | igd | 0.0045 | 7.94E-27 | exposure | TRUE | reported | MpDfko | textfile | 2 | TRUE |
|  | 145 | rs28399637 | G | A | G | A | -0.059 | 0.0001191 | 0.317274968 | 0.684222 | FALSE | FALSE | FALSE | ebi-a-GCST90018120 | 19 | 45324138 | 0.000124327 | 437235 | 0.34 | Idiopathic pulmonary fibrosis \|\| id:ebi-a-GCST90018120 | Idiopathic pulmonary fibrosis | Idiopathic pulmonary fibrosis \|\| \|\| | TRUE | igd | 0.0026 | 4.21E-116 | exposure | TRUE | reported | MpDfko | textfile | 2 | TRUE |
|  | 146 | rs28489942 | T | C | T | C | -0.0177 | -0.000134925 | 0.350315726 | 0.650594 | FALSE | FALSE | FALSE | ebi-a-GCST90018120 | 2 | 27368588 | 0.00012125 | 437235 | 0.27 | Idiopathic pulmonary fibrosis \|\| id:ebi-a-GCST90018120 | Idiopathic pulmonary fibrosis | Idiopathic pulmonary fibrosis \|\| \|\| | TRUE | igd | 0.0025 | 1.73E-12 | exposure | TRUE | reported | MpDfko | textfile | 2 | TRUE |
|  | 147 | rs2859400 | T | G | T | G | -0.0259 | -3.01E-05 | 0.163574142 | 0.164282 | FALSE | FALSE | FALSE | ebi-a-GCST90018120 | 12 | 121503136 | 0.000156053 | 437235 | 0.85 | Idiopathic pulmonary fibrosis \|\| id:ebi-a-GCST90018120 | Idiopathic pulmonary fibrosis | Idiopathic pulmonary fibrosis \|\| \|\| | TRUE | igd | 0.0032 | 5.18E-16 | exposure | TRUE | reported | MpDfko | textfile | 2 | TRUE |
|  | 148 | rs2927437 | A | G | A | G | 0.0345 | 2.59E-05 | 0.19274926 | 0.192172 | FALSE | FALSE | FALSE | ebi-a-GCST90018120 | 19 | 45241638 | 0.000146736 | 437235 | 0.86 | Idiopathic pulmonary fibrosis \|\| id:ebi-a-GCST90018120 | Idiopathic pulmonary fibrosis | Idiopathic pulmonary fibrosis \|\| \|\| | TRUE | igd | 0.003 | 5.25E-30 | exposure | TRUE | reported | MpDfko | textfile | 2 | TRUE |
|  | 149 | rs2965164 | C | T | C | T | 0.0245 | -9.87E-05 | 0.345785537 | 0.654461 | FALSE | FALSE | FALSE | ebi-a-GCST90018120 | 19 | 45202052 | 0.000121589 | 437235 | 0.42 | Idiopathic pulmonary fibrosis \|\| id:ebi-a-GCST90018120 | Idiopathic pulmonary fibrosis | Idiopathic pulmonary fibrosis \|\| \|\| | TRUE | igd | 0.0025 | 1.72E-22 | exposure | TRUE | reported | MpDfko | textfile | 2 | TRUE |
|  | 150 | rs3027067 | G | A | G | A | -0.0892 | 6.61E-05 | 0.041187179 | 0.958274 | FALSE | FALSE | FALSE | ebi-a-GCST90018120 | 1 | 159188483 | 0.000288769 | 437235 | 0.82 | Idiopathic pulmonary fibrosis \|\| id:ebi-a-GCST90018120 | Idiopathic pulmonary fibrosis | Idiopathic pulmonary fibrosis \|\| \|\| | TRUE | igd | 0.006 | 2.05E-49 | exposure | TRUE | reported | MpDfko | textfile | 2 | TRUE |
|  | 151 | rs3093070 | T | G | T | G | 0.1019 | -0.000389967 | 0.017918778 | 0.982246 | FALSE | FALSE | FALSE | ebi-a-GCST90018120 | 1 | 159680817 | 0.00043761 | 437235 | 0.37 | Idiopathic pulmonary fibrosis \|\| id:ebi-a-GCST90018120 | Idiopathic pulmonary fibrosis | Idiopathic pulmonary fibrosis \|\| \|\| | TRUE | igd | 0.0091 | 4.83E-29 | exposure | TRUE | reported | MpDfko | textfile | 2 | TRUE |
|  | 152 | rs315946 | G | A | G | A | -0.0253 | 0.000239099 | 0.144713778 | 0.854796 | FALSE | FALSE | FALSE | ebi-a-GCST90018120 | 2 | 113893864 | 0.000164234 | 437235 | 0.14 | Idiopathic pulmonary fibrosis \|\| id:ebi-a-GCST90018120 | Idiopathic pulmonary fibrosis | Idiopathic pulmonary fibrosis \|\| \|\| | TRUE | igd | 0.0034 | 1.75E-13 | exposure | TRUE | reported | MpDfko | textfile | 2 | TRUE |
|  | 153 | rs34095326 | G | A | G | A | -0.1919 | 4.99E-05 | 0.113927004 | 0.886216 | FALSE | FALSE | FALSE | ebi-a-GCST90018120 | 19 | 45395844 | 0.000182845 | 437235 | 0.79 | Idiopathic pulmonary fibrosis \|\| id:ebi-a-GCST90018120 | Idiopathic pulmonary fibrosis | Idiopathic pulmonary fibrosis \|\| \|\| | TRUE | igd | 0.004 | 1.00E-200 | exposure | TRUE | reported | MpDfko | textfile | 2 | TRUE |
|  | 154 | rs34179846 | T | C | T | C | 0.0472 | 5.18E-05 | 0.104540514 | 0.894911 | FALSE | FALSE | FALSE | ebi-a-GCST90018120 | 12 | 120917722 | 0.000188454 | 437235 | 0.780001 | Idiopathic pulmonary fibrosis \|\| id:ebi-a-GCST90018120 | Idiopathic pulmonary fibrosis | Idiopathic pulmonary fibrosis \|\| \|\| | TRUE | igd | 0.0039 | 3.52E-33 | exposure | TRUE | reported | MpDfko | textfile | 2 | TRUE |
|  | 155 | rs34473506 | C | T | C | T | 0.0268 | -6.19E-06 | 0.156676324 | 0.842813 | FALSE | FALSE | FALSE | ebi-a-GCST90018120 | 14 | 72988567 | 0.000158659 | 437235 | 0.97 | Idiopathic pulmonary fibrosis \|\| id:ebi-a-GCST90018120 | Idiopathic pulmonary fibrosis | Idiopathic pulmonary fibrosis \|\| \|\| | TRUE | igd | 0.0033 | 3.15E-16 | exposure | TRUE | reported | MpDfko | textfile | 2 | TRUE |
|  | 156 | rs34693607 | C | G | C | G | -0.0334 | -0.000181176 | 0.213577778 | 0.213155 | FALSE | TRUE | FALSE | ebi-a-GCST90018120 | 1 | 154661369 | 0.000141186 | 437235 | 0.2 | Idiopathic pulmonary fibrosis \|\| id:ebi-a-GCST90018120 | Idiopathic pulmonary fibrosis | Idiopathic pulmonary fibrosis \|\| \|\| | TRUE | igd | 0.0029 | 5.27E-30 | exposure | TRUE | reported | MpDfko | textfile | 2 | TRUE |
|  | 157 | rs34982954 | T | C | T | C | -0.0147 | -0.000189869 | 0.474337842 | 0.525366 | FALSE | FALSE | FALSE | ebi-a-GCST90018120 | 16 | 79040870 | 0.000115707 | 437235 | 0.1 | Idiopathic pulmonary fibrosis \|\| id:ebi-a-GCST90018120 | Idiopathic pulmonary fibrosis | Idiopathic pulmonary fibrosis \|\| \|\| | TRUE | igd | 0.0024 | 9.07E-10 | exposure | TRUE | reported | MpDfko | textfile | 2 | TRUE |
|  | 158 | rs35315097 | A | C | A | C | 0.0508 | 0.000354101 | 0.053496183 | 0.946323 | FALSE | FALSE | FALSE | ebi-a-GCST90018120 | 2 | 27929023 | 0.00025657 | 437235 | 0.17 | Idiopathic pulmonary fibrosis \|\| id:ebi-a-GCST90018120 | Idiopathic pulmonary fibrosis | Idiopathic pulmonary fibrosis \|\| \|\| | TRUE | igd | 0.0054 | 2.26E-21 | exposure | TRUE | reported | MpDfko | textfile | 2 | TRUE |
|  | 159 | rs35521407 | C | CT | C | CT | 0.0194 | -0.00012565 | 0.159948 | 0.841187 | FALSE | FALSE | FALSE | ebi-a-GCST90018120 | 8 | 103507581 | 0.000160614 | 437235 | 0.44 | Idiopathic pulmonary fibrosis \|\| id:ebi-a-GCST90018120 | Idiopathic pulmonary fibrosis | Idiopathic pulmonary fibrosis \|\| \|\| | TRUE | igd | 0.0033 | 3.77E-09 | exposure | TRUE | reported | MpDfko | textfile | 2 | TRUE |
|  | 160 | rs35860194 | C | A | C | A | 0.0635 | 0.000459116 | 0.015833499 | 0.984027 | FALSE | FALSE | FALSE | ebi-a-GCST90018120 | 2 | 26946788 | 0.000460659 | 437235 | 0.32 | Idiopathic pulmonary fibrosis \|\| id:ebi-a-GCST90018120 | Idiopathic pulmonary fibrosis | Idiopathic pulmonary fibrosis \|\| \|\| | TRUE | igd | 0.0096 | 4.12E-11 | exposure | TRUE | reported | MpDfko | textfile | 2 | TRUE |
|  | 161 | rs36227 | T | C | T | C | 0.02 | 4.67E-05 | 0.180224443 | 0.180163 | FALSE | FALSE | FALSE | ebi-a-GCST90018120 | 16 | 2195556 | 0.000150504 | 437235 | 0.760001 | Idiopathic pulmonary fibrosis \|\| id:ebi-a-GCST90018120 | Idiopathic pulmonary fibrosis | Idiopathic pulmonary fibrosis \|\| \|\| | TRUE | igd | 0.0031 | 1.14E-10 | exposure | TRUE | reported | MpDfko | textfile | 2 | TRUE |
|  | 162 | rs365653 | A | G | A | G | 0.0644 | -4.49E-05 | 0.114569393 | 0.884733 | FALSE | FALSE | FALSE | ebi-a-GCST90018120 | 19 | 45361646 | 0.000182886 | 437235 | 0.81 | Idiopathic pulmonary fibrosis \|\| id:ebi-a-GCST90018120 | Idiopathic pulmonary fibrosis | Idiopathic pulmonary fibrosis \|\| \|\| | TRUE | igd | 0.0039 | 6.49E-62 | exposure | TRUE | reported | MpDfko | textfile | 2 | TRUE |
|  | 163 | rs3745150 | G | C | G | C | 0.038 | 0.000151906 | 0.39392291 | 0.393282 | FALSE | TRUE | FALSE | ebi-a-GCST90018120 | 19 | 45385759 | 0.000119528 | 437235 | 0.2 | Idiopathic pulmonary fibrosis \|\| id:ebi-a-GCST90018120 | Idiopathic pulmonary fibrosis | Idiopathic pulmonary fibrosis \|\| \|\| | TRUE | igd | 0.0026 | 3.35E-49 | exposure | TRUE | reported | MpDfko | textfile | 2 | TRUE |
|  | 164 | rs3781619 | G | A | G | A | -0.0315 | 0.000195636 | 0.155155667 | 0.845245 | FALSE | FALSE | FALSE | ebi-a-GCST90018120 | 11 | 47255317 | 0.000159763 | 437235 | 0.22 | Idiopathic pulmonary fibrosis \|\| id:ebi-a-GCST90018120 | Idiopathic pulmonary fibrosis | Idiopathic pulmonary fibrosis \|\| \|\| | TRUE | igd | 0.0033 | 6.07E-22 | exposure | TRUE | reported | MpDfko | textfile | 2 | TRUE |
|  | 165 | rs3812814 | A | G | A | G | 0.0247 | -2.95E-05 | 0.122842151 | 0.877489 | FALSE | FALSE | FALSE | ebi-a-GCST90018120 | 12 | 95927037 | 0.000176633 | 437235 | 0.87 | Idiopathic pulmonary fibrosis \|\| id:ebi-a-GCST90018120 | Idiopathic pulmonary fibrosis | Idiopathic pulmonary fibrosis \|\| \|\| | TRUE | igd | 0.0037 | 1.27E-11 | exposure | TRUE | reported | MpDfko | textfile | 2 | TRUE |
|  | 166 | rs3815989 | C | T | C | T | -0.0267 | -6.79E-05 | 0.11339958 | 0.885687 | FALSE | FALSE | FALSE | ebi-a-GCST90018120 | 12 | 121666476 | 0.00018168 | 437235 | 0.709999 | Idiopathic pulmonary fibrosis \|\| id:ebi-a-GCST90018120 | Idiopathic pulmonary fibrosis | Idiopathic pulmonary fibrosis \|\| \|\| | TRUE | igd | 0.0037 | 8.56E-13 | exposure | TRUE | reported | MpDfko | textfile | 2 | TRUE |
|  | 167 | rs3850515 | C | G | C | G | -0.0654 | 0.000839333 | 0.013760228 | 0.013986 | FALSE | TRUE | FALSE | ebi-a-GCST90018120 | 12 | 121488519 | 0.000492189 | 437235 | 0.089 | Idiopathic pulmonary fibrosis \|\| id:ebi-a-GCST90018120 | Idiopathic pulmonary fibrosis | Idiopathic pulmonary fibrosis \|\| \|\| | TRUE | igd | 0.0093 | 2.08E-12 | exposure | TRUE | reported | MpDfko | textfile | 2 | TRUE |
|  | 168 | rs405697 | A | G | A | G | -0.062 | -3.28E-05 | 0.276026832 | 0.27653 | FALSE | FALSE | FALSE | ebi-a-GCST90018120 | 19 | 45404691 | 0.000130214 | 437235 | 0.8 | Idiopathic pulmonary fibrosis \|\| id:ebi-a-GCST90018120 | Idiopathic pulmonary fibrosis | Idiopathic pulmonary fibrosis \|\| \|\| | TRUE | igd | 0.0028 | 1.16E-107 | exposure | TRUE | reported | MpDfko | textfile | 2 | TRUE |
|  | 169 | rs41264481 | A | C | A | C | 0.1018 | 0.000137432 | 0.017669403 | 0.982642 | FALSE | FALSE | FALSE | ebi-a-GCST90018120 | 1 | 159436349 | 0.000442198 | 437235 | 0.75 | Idiopathic pulmonary fibrosis \|\| id:ebi-a-GCST90018120 | Idiopathic pulmonary fibrosis | Idiopathic pulmonary fibrosis \|\| \|\| | TRUE | igd | 0.009 | 2.29E-29 | exposure | TRUE | reported | MpDfko | textfile | 2 | TRUE |
|  | 170 | rs41289512 | C | G | C | G | -0.1788 | 2.36E-05 | 0.043305802 | 0.042662 | FALSE | TRUE | FALSE | ebi-a-GCST90018120 | 19 | 45351516 | 0.000285546 | 437235 | 0.93 | Idiopathic pulmonary fibrosis \|\| id:ebi-a-GCST90018120 | Idiopathic pulmonary fibrosis | Idiopathic pulmonary fibrosis \|\| \|\| | TRUE | igd | 0.0059 | 1.25E-198 | exposure | TRUE | reported | MpDfko | textfile | 2 | TRUE |
|  | 171 | rs41290100 | C | T | C | T | 0.071 | 0.000241013 | 0.029858706 | 0.971817 | FALSE | FALSE | FALSE | ebi-a-GCST90018120 | 19 | 45370941 | 0.000359425 | 437235 | 0.5 | Idiopathic pulmonary fibrosis \|\| id:ebi-a-GCST90018120 | Idiopathic pulmonary fibrosis | Idiopathic pulmonary fibrosis \|\| \|\| | TRUE | igd | 0.0075 | 2.02E-21 | exposure | TRUE | reported | MpDfko | textfile | 2 | TRUE |
|  | 172 | rs41310887 | G | A | G | A | 0.0404 | 7.64E-05 | 0.048358189 | 0.951281 | FALSE | FALSE | FALSE | ebi-a-GCST90018120 | 1 | 154321623 | 0.000268404 | 437235 | 0.77 | Idiopathic pulmonary fibrosis \|\| id:ebi-a-GCST90018120 | Idiopathic pulmonary fibrosis | Idiopathic pulmonary fibrosis \|\| \|\| | TRUE | igd | 0.0056 | 6.22E-13 | exposure | TRUE | reported | MpDfko | textfile | 2 | TRUE |
|  | 173 | rs41310893 | C | T | C | T | 0.045 | -0.000451476 | 0.049845481 | 0.95051 | FALSE | FALSE | FALSE | ebi-a-GCST90018120 | 1 | 154514267 | 0.000266856 | 437235 | 0.0909997 | Idiopathic pulmonary fibrosis \|\| id:ebi-a-GCST90018120 | Idiopathic pulmonary fibrosis | Idiopathic pulmonary fibrosis \|\| \|\| | TRUE | igd | 0.0056 | 5.78E-16 | exposure | TRUE | reported | MpDfko | textfile | 2 | TRUE |
|  | 174 | rs4131568 | C | T | C | T | 0.0915 | -8.87E-05 | 0.338026178 | 0.66124 | FALSE | FALSE | FALSE | ebi-a-GCST90018120 | 1 | 159722056 | 0.000122373 | 437235 | 0.47 | Idiopathic pulmonary fibrosis \|\| id:ebi-a-GCST90018120 | Idiopathic pulmonary fibrosis | Idiopathic pulmonary fibrosis \|\| \|\| | TRUE | igd | 0.0025 | 1.00E-200 | exposure | TRUE | reported | MpDfko | textfile | 2 | TRUE |
|  | 175 | rs416041 | G | A | G | A | 0.0361 | -2.31E-05 | 0.375985784 | 0.375311 | FALSE | FALSE | FALSE | ebi-a-GCST90018120 | 19 | 45370854 | 0.000119754 | 437235 | 0.85 | Idiopathic pulmonary fibrosis \|\| id:ebi-a-GCST90018120 | Idiopathic pulmonary fibrosis | Idiopathic pulmonary fibrosis \|\| \|\| | TRUE | igd | 0.0026 | 7.91E-44 | exposure | TRUE | reported | MpDfko | textfile | 2 | TRUE |
|  | 176 | rs4282816 | G | A | G | A | -0.0175 | -0.000136566 | 0.261392916 | 0.261765 | FALSE | FALSE | FALSE | ebi-a-GCST90018120 | 1 | 159292850 | 0.000131602 | 437235 | 0.3 | Idiopathic pulmonary fibrosis \|\| id:ebi-a-GCST90018120 | Idiopathic pulmonary fibrosis | Idiopathic pulmonary fibrosis \|\| \|\| | TRUE | igd | 0.0027 | 7.55E-11 | exposure | TRUE | reported | MpDfko | textfile | 2 | TRUE |
|  | 177 | rs4394621 | G | A | G | A | 0.1088 | 7.43E-06 | 0.184154093 | 0.183254 | FALSE | FALSE | FALSE | ebi-a-GCST90018120 | 1 | 66165836 | 0.000149433 | 437235 | 0.96 | Idiopathic pulmonary fibrosis \|\| id:ebi-a-GCST90018120 | Idiopathic pulmonary fibrosis | Idiopathic pulmonary fibrosis \|\| \|\| | TRUE | igd | 0.0031 | 1.00E-200 | exposure | TRUE | reported | MpDfko | textfile | 2 | TRUE |
|  | 178 | rs4411129 | C | G | C | G | 0.0178 | 0.000214247 | 0.292615757 | 0.293502 | FALSE | TRUE | FALSE | ebi-a-GCST90018120 | 1 | 159785284 | 0.000126993 | 437235 | 0.0920005 | Idiopathic pulmonary fibrosis \|\| id:ebi-a-GCST90018120 | Idiopathic pulmonary fibrosis | Idiopathic pulmonary fibrosis \|\| \|\| | TRUE | igd | 0.0026 | 1.38E-11 | exposure | TRUE | reported | MpDfko | textfile | 2 | TRUE |
|  | 179 | rs4420638 | A | G | A | G | -0.2217 | 0.000126015 | 0.191027615 | 0.810224 | FALSE | FALSE | FALSE | ebi-a-GCST90018120 | 19 | 45422946 | 0.000147446 | 437235 | 0.39 | Idiopathic pulmonary fibrosis \|\| id:ebi-a-GCST90018120 | Idiopathic pulmonary fibrosis | Idiopathic pulmonary fibrosis \|\| \|\| | TRUE | igd | 0.003 | 1.00E-200 | exposure | TRUE | reported | MpDfko | textfile | 2 | TRUE |
|  | 180 | rs4527079 | A | G | A | G | -0.0268 | 8.02E-05 | 0.078802039 | 0.079309 | FALSE | FALSE | FALSE | ebi-a-GCST90018120 | 17 | 76368761 | 0.000213933 | 437235 | 0.709999 | Idiopathic pulmonary fibrosis \|\| id:ebi-a-GCST90018120 | Idiopathic pulmonary fibrosis | Idiopathic pulmonary fibrosis \|\| \|\| | TRUE | igd | 0.0044 | 1.35E-09 | exposure | TRUE | reported | MpDfko | textfile | 2 | TRUE |
|  | 181 | rs45446698 | T | G | T | G | -0.0425 | -0.000295466 | 0.042780939 | 0.957842 | FALSE | FALSE | FALSE | ebi-a-GCST90018120 | 7 | 99332948 | 0.000287824 | 437235 | 0.3 | Idiopathic pulmonary fibrosis \|\| id:ebi-a-GCST90018120 | Idiopathic pulmonary fibrosis | Idiopathic pulmonary fibrosis \|\| \|\| | TRUE | igd | 0.006 | 1.11E-12 | exposure | TRUE | reported | MpDfko | textfile | 2 | TRUE |
|  | 182 | rs4714508 | A | G | A | G | 0.0198 | -6.90E-05 | 0.351713 | 0.654519 | FALSE | FALSE | FALSE | ebi-a-GCST90018120 | 6 | 41671677 | 0.000122347 | 437235 | 0.57 | Idiopathic pulmonary fibrosis \|\| id:ebi-a-GCST90018120 | Idiopathic pulmonary fibrosis | Idiopathic pulmonary fibrosis \|\| \|\| | TRUE | igd | 0.0025 | 5.40E-15 | exposure | TRUE | reported | MpDfko | textfile | 2 | TRUE |
|  | 183 | rs4767878 | A | G | A | G | 0.0518 | -0.000252046 | 0.026684643 | 0.026553 | FALSE | FALSE | FALSE | ebi-a-GCST90018120 | 12 | 120564999 | 0.000359333 | 437235 | 0.48 | Idiopathic pulmonary fibrosis \|\| id:ebi-a-GCST90018120 | Idiopathic pulmonary fibrosis | Idiopathic pulmonary fibrosis \|\| \|\| | TRUE | igd | 0.0075 | 4.90E-12 | exposure | TRUE | reported | MpDfko | textfile | 2 | TRUE |
|  | 184 | rs4767915 | G | A | G | A | -0.0347 | 0.000281389 | 0.097756958 | 0.901991 | FALSE | FALSE | FALSE | ebi-a-GCST90018120 | 12 | 120999127 | 0.000194357 | 437235 | 0.15 | Idiopathic pulmonary fibrosis \|\| id:ebi-a-GCST90018120 | Idiopathic pulmonary fibrosis | Idiopathic pulmonary fibrosis \|\| \|\| | TRUE | igd | 0.004 | 5.72E-18 | exposure | TRUE | reported | MpDfko | textfile | 2 | TRUE |
|  | 185 | rs4806073 | T | C | T | C | -0.0286 | 0.000321438 | 0.066765561 | 0.067047 | FALSE | FALSE | FALSE | ebi-a-GCST90018120 | 19 | 35555190 | 0.000230846 | 437235 | 0.16 | Idiopathic pulmonary fibrosis \|\| id:ebi-a-GCST90018120 | Idiopathic pulmonary fibrosis | Idiopathic pulmonary fibrosis \|\| \|\| | TRUE | igd | 0.0045 | 3.27E-10 | exposure | TRUE | reported | MpDfko | textfile | 2 | TRUE |
|  | 186 | rs483082 | G | T | G | T | -0.152 | 9.55E-05 | 0.237110595 | 0.76381 | FALSE | FALSE | FALSE | ebi-a-GCST90018120 | 19 | 45416178 | 0.000136437 | 437235 | 0.49 | Idiopathic pulmonary fibrosis \|\| id:ebi-a-GCST90018120 | Idiopathic pulmonary fibrosis | Idiopathic pulmonary fibrosis \|\| \|\| | TRUE | igd | 0.0029 | 1.00E-200 | exposure | TRUE | reported | MpDfko | textfile | 2 | TRUE |
|  | 187 | rs4916009 | C | T | C | T | 0.0335 | -0.000154162 | 0.091693935 | 0.908781 | FALSE | FALSE | FALSE | ebi-a-GCST90018120 | 1 | 65371418 | 0.000200652 | 437235 | 0.44 | Idiopathic pulmonary fibrosis \|\| id:ebi-a-GCST90018120 | Idiopathic pulmonary fibrosis | Idiopathic pulmonary fibrosis \|\| \|\| | TRUE | igd | 0.0042 | 1.37E-15 | exposure | TRUE | reported | MpDfko | textfile | 2 | TRUE |
|  | 188 | rs4925671 | T | C | T | C | 0.0194 | -1.69E-05 | 0.302022351 | 0.302392 | FALSE | FALSE | FALSE | ebi-a-GCST90018120 | 1 | 247622874 | 0.000125821 | 437235 | 0.89 | Idiopathic pulmonary fibrosis \|\| id:ebi-a-GCST90018120 | Idiopathic pulmonary fibrosis | Idiopathic pulmonary fibrosis \|\| \|\| | TRUE | igd | 0.0026 | 6.44E-14 | exposure | TRUE | reported | MpDfko | textfile | 2 | TRUE |
|  | 189 | rs507666 | G | A | G | A | 0.0329 | -0.000317317 | 0.184602484 | 0.815334 | FALSE | FALSE | FALSE | ebi-a-GCST90018120 | 9 | 136149399 | 0.000149067 | 437235 | 0.0329997 | Idiopathic pulmonary fibrosis \|\| id:ebi-a-GCST90018120 | Idiopathic pulmonary fibrosis | Idiopathic pulmonary fibrosis \|\| \|\| | TRUE | igd | 0.0031 | 2.95E-26 | exposure | TRUE | reported | MpDfko | textfile | 2 | TRUE |
|  | 190 | rs55688443 | G | T | G | T | -0.0973 | -0.000108727 | 0.021692872 | 0.978167 | FALSE | FALSE | FALSE | ebi-a-GCST90018120 | 1 | 159447163 | 0.000395382 | 437235 | 0.780001 | Idiopathic pulmonary fibrosis \|\| id:ebi-a-GCST90018120 | Idiopathic pulmonary fibrosis | Idiopathic pulmonary fibrosis \|\| \|\| | TRUE | igd | 0.0081 | 2.67E-33 | exposure | TRUE | reported | MpDfko | textfile | 2 | TRUE |
|  | 191 | rs55997241 | G | A | G | A | 0.0315 | -0.000151773 | 0.076439214 | 0.923175 | FALSE | FALSE | FALSE | ebi-a-GCST90018120 | 1 | 154671128 | 0.000217359 | 437235 | 0.49 | Idiopathic pulmonary fibrosis \|\| id:ebi-a-GCST90018120 | Idiopathic pulmonary fibrosis | Idiopathic pulmonary fibrosis \|\| \|\| | TRUE | igd | 0.0046 | 5.18E-12 | exposure | TRUE | reported | MpDfko | textfile | 2 | TRUE |
|  | 192 | rs56156770 | C | T | C | T | 0.0845 | 0.000212447 | 0.021813905 | 0.978434 | FALSE | FALSE | FALSE | ebi-a-GCST90018120 | 12 | 121421992 | 0.000397661 | 437235 | 0.59 | Idiopathic pulmonary fibrosis \|\| id:ebi-a-GCST90018120 | Idiopathic pulmonary fibrosis | Idiopathic pulmonary fibrosis \|\| \|\| | TRUE | igd | 0.0083 | 1.90E-24 | exposure | TRUE | reported | MpDfko | textfile | 2 | TRUE |
|  | 193 | rs56401401 | G | A | G | A | -0.0729 | -0.000118927 | 0.032838882 | 0.967041 | FALSE | FALSE | FALSE | ebi-a-GCST90018120 | 12 | 121186314 | 0.000323893 | 437235 | 0.709999 | Idiopathic pulmonary fibrosis \|\| id:ebi-a-GCST90018120 | Idiopathic pulmonary fibrosis | Idiopathic pulmonary fibrosis \|\| \|\| | TRUE | igd | 0.0067 | 1.54E-27 | exposure | TRUE | reported | MpDfko | textfile | 2 | TRUE |
|  | 194 | rs572144 | T | C | T | C | -0.014 | -0.000235105 | 0.459977524 | 0.53995 | FALSE | FALSE | FALSE | ebi-a-GCST90018120 | 1 | 66670804 | 0.000115881 | 437235 | 0.0420001 | Idiopathic pulmonary fibrosis \|\| id:ebi-a-GCST90018120 | Idiopathic pulmonary fibrosis | Idiopathic pulmonary fibrosis \|\| \|\| | TRUE | igd | 0.0024 | 4.66E-09 | exposure | TRUE | reported | MpDfko | textfile | 2 | TRUE |
|  | 195 | rs584007 | A | G | A | G | -0.0901 | -0.000247775 | 0.355942076 | 0.35866 | FALSE | FALSE | FALSE | ebi-a-GCST90018120 | 19 | 45416478 | 0.000120947 | 437235 | 0.04 | Idiopathic pulmonary fibrosis \|\| id:ebi-a-GCST90018120 | Idiopathic pulmonary fibrosis | Idiopathic pulmonary fibrosis \|\| \|\| | TRUE | igd | 0.0026 | 1.00E-200 | exposure | TRUE | reported | MpDfko | textfile | 2 | TRUE |
|  | 196 | rs59104589 | C | T | C | T | 0.0183 | 4.18E-05 | 0.355894 | 0.6402 | FALSE | FALSE | FALSE | ebi-a-GCST90018120 | 2 | 242237902 | 0.000120414 | 437235 | 0.73 | Idiopathic pulmonary fibrosis \|\| id:ebi-a-GCST90018120 | Idiopathic pulmonary fibrosis | Idiopathic pulmonary fibrosis \|\| \|\| | TRUE | igd | 0.0025 | 1.49E-13 | exposure | TRUE | reported | MpDfko | textfile | 2 | TRUE |
|  | 197 | rs60049679 | G | C | G | C | -0.1277 | 0.000138601 | 0.07720473 | 0.073048 | FALSE | TRUE | FALSE | ebi-a-GCST90018120 | 19 | 45429708 | 0.000237096 | 437235 | 0.56 | Idiopathic pulmonary fibrosis \|\| id:ebi-a-GCST90018120 | Idiopathic pulmonary fibrosis | Idiopathic pulmonary fibrosis \|\| \|\| | TRUE | igd | 0.0046 | 3.68E-169 | exposure | TRUE | reported | MpDfko | textfile | 2 | TRUE |
|  | 198 | rs60517797 | T | C | T | C | 0.0325 | -0.000252479 | 0.08025554 | 0.918826 | FALSE | FALSE | FALSE | ebi-a-GCST90018120 | 1 | 154642224 | 0.000211777 | 437235 | 0.23 | Idiopathic pulmonary fibrosis \|\| id:ebi-a-GCST90018120 | Idiopathic pulmonary fibrosis | Idiopathic pulmonary fibrosis \|\| \|\| | TRUE | igd | 0.0043 | 6.66E-14 | exposure | TRUE | reported | MpDfko | textfile | 2 | TRUE |
|  | 199 | rs61753391 | G | A | G | A | 0.0515 | 0.000582951 | 0.026332124 | 0.973965 | FALSE | FALSE | FALSE | ebi-a-GCST90018120 | 1 | 65849887 | 0.000362548 | 437235 | 0.11 | Idiopathic pulmonary fibrosis \|\| id:ebi-a-GCST90018120 | Idiopathic pulmonary fibrosis | Idiopathic pulmonary fibrosis \|\| \|\| | TRUE | igd | 0.0076 | 9.44E-12 | exposure | TRUE | reported | MpDfko | textfile | 2 | TRUE |
|  | 200 | rs61806853 | T | C | T | C | -0.0452 | -0.000207158 | 0.049732908 | 0.950244 | FALSE | FALSE | FALSE | ebi-a-GCST90018120 | 1 | 154154587 | 0.000266063 | 437235 | 0.44 | Idiopathic pulmonary fibrosis \|\| id:ebi-a-GCST90018120 | Idiopathic pulmonary fibrosis | Idiopathic pulmonary fibrosis \|\| \|\| | TRUE | igd | 0.0055 | 3.80E-16 | exposure | TRUE | reported | MpDfko | textfile | 2 | TRUE |
|  | 201 | rs61811421 | C | T | C | T | 0.0396 | -2.55E-05 | 0.22577334 | 0.773605 | FALSE | FALSE | FALSE | ebi-a-GCST90018120 | 1 | 154653951 | 0.000138201 | 437235 | 0.85 | Idiopathic pulmonary fibrosis \|\| id:ebi-a-GCST90018120 | Idiopathic pulmonary fibrosis | Idiopathic pulmonary fibrosis \|\| \|\| | TRUE | igd | 0.0029 | 1.17E-43 | exposure | TRUE | reported | MpDfko | textfile | 2 | TRUE |
|  | 202 | rs61821592 | C | T | C | T | -0.08 | 1.89E-05 | 0.021472593 | 0.978983 | FALSE | FALSE | FALSE | ebi-a-GCST90018120 | 1 | 159670262 | 0.000402979 | 437235 | 0.96 | Idiopathic pulmonary fibrosis \|\| id:ebi-a-GCST90018120 | Idiopathic pulmonary fibrosis | Idiopathic pulmonary fibrosis \|\| \|\| | TRUE | igd | 0.0084 | 1.51E-21 | exposure | TRUE | reported | MpDfko | textfile | 2 | TRUE |
|  | 203 | rs62023507 | A | G | A | G | -0.0329 | 0.000408194 | 0.065627089 | 0.934298 | FALSE | FALSE | FALSE | ebi-a-GCST90018120 | 15 | 53124819 | 0.000232955 | 437235 | 0.08 | Idiopathic pulmonary fibrosis \|\| id:ebi-a-GCST90018120 | Idiopathic pulmonary fibrosis | Idiopathic pulmonary fibrosis \|\| \|\| | TRUE | igd | 0.0048 | 9.16E-12 | exposure | TRUE | reported | MpDfko | textfile | 2 | TRUE |
|  | 204 | rs62117160 | G | A | G | A | 0.0587 | 0.000101293 | 0.04595752 | 0.954317 | FALSE | FALSE | FALSE | ebi-a-GCST90018120 | 19 | 45232161 | 0.000276684 | 437235 | 0.719999 | Idiopathic pulmonary fibrosis \|\| id:ebi-a-GCST90018120 | Idiopathic pulmonary fibrosis | Idiopathic pulmonary fibrosis \|\| \|\| | TRUE | igd | 0.0058 | 3.48E-24 | exposure | TRUE | reported | MpDfko | textfile | 2 | TRUE |
|  | 205 | rs62513191 | T | C | T | C | 0.036 | -0.000227754 | 0.043681928 | 0.956519 | FALSE | FALSE | FALSE | ebi-a-GCST90018120 | 8 | 129155362 | 0.000283114 | 437235 | 0.42 | Idiopathic pulmonary fibrosis \|\| id:ebi-a-GCST90018120 | Idiopathic pulmonary fibrosis | Idiopathic pulmonary fibrosis \|\| \|\| | TRUE | igd | 0.0059 | 1.21E-09 | exposure | TRUE | reported | MpDfko | textfile | 2 | TRUE |
|  | 206 | rs6501734 | C | T | C | T | -0.028 | 0.000154519 | 0.105524826 | 0.8948 | FALSE | FALSE | FALSE | ebi-a-GCST90018120 | 17 | 72735629 | 0.000188559 | 437235 | 0.41 | Idiopathic pulmonary fibrosis \|\| id:ebi-a-GCST90018120 | Idiopathic pulmonary fibrosis | Idiopathic pulmonary fibrosis \|\| \|\| | TRUE | igd | 0.0038 | 2.69E-13 | exposure | TRUE | reported | MpDfko | textfile | 2 | TRUE |
|  | 207 | rs6573778 | T | C | T | C | 0.0153 | -7.72E-05 | 0.472722 | 0.481484 | FALSE | FALSE | FALSE | ebi-a-GCST90018120 | 14 | 24872209 | 0.000116314 | 437235 | 0.51 | Idiopathic pulmonary fibrosis \|\| id:ebi-a-GCST90018120 | Idiopathic pulmonary fibrosis | Idiopathic pulmonary fibrosis \|\| \|\| | TRUE | igd | 0.0024 | 1.78E-10 | exposure | TRUE | reported | MpDfko | textfile | 2 | TRUE |
|  | 208 | rs6672331 | G | C | G | C | 0.1248 | -0.000104414 | 0.032780996 | 0.03301 | FALSE | TRUE | FALSE | ebi-a-GCST90018120 | 1 | 65975847 | 0.000323714 | 437235 | 0.75 | Idiopathic pulmonary fibrosis \|\| id:ebi-a-GCST90018120 | Idiopathic pulmonary fibrosis | Idiopathic pulmonary fibrosis \|\| \|\| | TRUE | igd | 0.0068 | 4.75E-76 | exposure | TRUE | reported | MpDfko | textfile | 2 | TRUE |
|  | 209 | rs6698040 | T | C | T | C | -0.0454 | -1.26E-05 | 0.223811265 | 0.225091 | FALSE | FALSE | FALSE | ebi-a-GCST90018120 | 1 | 154432948 | 0.000138362 | 437235 | 0.93 | Idiopathic pulmonary fibrosis \|\| id:ebi-a-GCST90018120 | Idiopathic pulmonary fibrosis | Idiopathic pulmonary fibrosis \|\| \|\| | TRUE | igd | 0.0028 | 8.50E-58 | exposure | TRUE | reported | MpDfko | textfile | 2 | TRUE |
|  | 210 | rs672140 | T | C | T | C | -0.0394 | -0.000306327 | 0.078364989 | 0.921775 | FALSE | FALSE | FALSE | ebi-a-GCST90018120 | 12 | 121577847 | 0.000215206 | 437235 | 0.16 | Idiopathic pulmonary fibrosis \|\| id:ebi-a-GCST90018120 | Idiopathic pulmonary fibrosis | Idiopathic pulmonary fibrosis \|\| \|\| | TRUE | igd | 0.0043 | 1.02E-19 | exposure | TRUE | reported | MpDfko | textfile | 2 | TRUE |
|  | 211 | rs6728590 | A | G | A | G | 0.0348 | 7.33E-05 | 0.485252066 | 0.515484 | FALSE | FALSE | FALSE | ebi-a-GCST90018120 | 2 | 113844600 | 0.000115691 | 437235 | 0.53 | Idiopathic pulmonary fibrosis \|\| id:ebi-a-GCST90018120 | Idiopathic pulmonary fibrosis | Idiopathic pulmonary fibrosis \|\| \|\| | TRUE | igd | 0.0025 | 1.51E-43 | exposure | TRUE | reported | MpDfko | textfile | 2 | TRUE |
|  | 212 | rs6792725 | A | G | A | G | -0.0195 | 0.000109483 | 0.30437 | 0.307264 | FALSE | FALSE | FALSE | ebi-a-GCST90018120 | 3 | 24520283 | 0.000129122 | 437235 | 0.4 | Idiopathic pulmonary fibrosis \|\| id:ebi-a-GCST90018120 | Idiopathic pulmonary fibrosis | Idiopathic pulmonary fibrosis \|\| \|\| | TRUE | igd | 0.0027 | 5.12E-13 | exposure | TRUE | reported | MpDfko | textfile | 2 | TRUE |
|  | 213 | rs6859 | A | G | A | G | 0.0548 | -0.000207361 | 0.42245825 | 0.422128 | FALSE | FALSE | FALSE | ebi-a-GCST90018120 | 19 | 45382034 | 0.000116956 | 437235 | 0.0759994 | Idiopathic pulmonary fibrosis \|\| id:ebi-a-GCST90018120 | Idiopathic pulmonary fibrosis | Idiopathic pulmonary fibrosis \|\| \|\| | TRUE | igd | 0.0024 | 6.46E-115 | exposure | TRUE | reported | MpDfko | textfile | 2 | TRUE |
|  | 214 | rs6986685 | G | A | G | A | 0.0198 | -3.15E-05 | 0.179274583 | 0.822312 | FALSE | FALSE | FALSE | ebi-a-GCST90018120 | 8 | 9233771 | 0.000151243 | 437235 | 0.84 | Idiopathic pulmonary fibrosis \|\| id:ebi-a-GCST90018120 | Idiopathic pulmonary fibrosis | Idiopathic pulmonary fibrosis \|\| \|\| | TRUE | igd | 0.0032 | 4.28E-10 | exposure | TRUE | reported | MpDfko | textfile | 2 | TRUE |
|  | 215 | rs7084062 | A | G | A | G | 0.017 | -3.12E-05 | 0.491779 | 0.509171 | FALSE | FALSE | FALSE | ebi-a-GCST90018120 | 10 | 133736636 | 0.000116978 | 437235 | 0.79 | Idiopathic pulmonary fibrosis \|\| id:ebi-a-GCST90018120 | Idiopathic pulmonary fibrosis | Idiopathic pulmonary fibrosis \|\| \|\| | TRUE | igd | 0.0024 | 1.49E-12 | exposure | TRUE | reported | MpDfko | textfile | 2 | TRUE |
|  | 216 | rs71441083 | G | A | G | A | 0.0556 | 9.50E-05 | 0.033732261 | 0.965865 | FALSE | FALSE | FALSE | ebi-a-GCST90018120 | 2 | 27747949 | 0.000318221 | 437235 | 0.77 | Idiopathic pulmonary fibrosis \|\| id:ebi-a-GCST90018120 | Idiopathic pulmonary fibrosis | Idiopathic pulmonary fibrosis \|\| \|\| | TRUE | igd | 0.0066 | 6.02E-17 | exposure | TRUE | reported | MpDfko | textfile | 2 | TRUE |
|  | 217 | rs71454665 | C | T | C | T | 0.0718 | 0.000127679 | 0.057363828 | 0.94242 | FALSE | FALSE | FALSE | ebi-a-GCST90018120 | 12 | 121198711 | 0.000248423 | 437235 | 0.61 | Idiopathic pulmonary fibrosis \|\| id:ebi-a-GCST90018120 | Idiopathic pulmonary fibrosis | Idiopathic pulmonary fibrosis \|\| \|\| | TRUE | igd | 0.0052 | 5.19E-44 | exposure | TRUE | reported | MpDfko | textfile | 2 | TRUE |
|  | 218 | rs714948 | C | A | C | A | -0.0405 | 4.59E-05 | 0.117267995 | 0.883144 | FALSE | FALSE | FALSE | ebi-a-GCST90018120 | 19 | 45165912 | 0.000179872 | 437235 | 0.8 | Idiopathic pulmonary fibrosis \|\| id:ebi-a-GCST90018120 | Idiopathic pulmonary fibrosis | Idiopathic pulmonary fibrosis \|\| \|\| | TRUE | igd | 0.0038 | 4.88E-27 | exposure | TRUE | reported | MpDfko | textfile | 2 | TRUE |
|  | 219 | rs71556711 | C | T | C | T | -0.0292 | -4.41E-05 | 0.090616444 | 0.909778 | FALSE | FALSE | FALSE | ebi-a-GCST90018120 | 7 | 72854549 | 0.00020167 | 437235 | 0.83 | Idiopathic pulmonary fibrosis \|\| id:ebi-a-GCST90018120 | Idiopathic pulmonary fibrosis | Idiopathic pulmonary fibrosis \|\| \|\| | TRUE | igd | 0.0042 | 3.39E-12 | exposure | TRUE | reported | MpDfko | textfile | 2 | TRUE |
|  | 220 | rs7223548 | G | C | G | C | 0.0282 | -1.48E-05 | 0.101461126 | 0.101936 | FALSE | TRUE | FALSE | ebi-a-GCST90018120 | 17 | 76445231 | 0.000191039 | 437235 | 0.94 | Idiopathic pulmonary fibrosis \|\| id:ebi-a-GCST90018120 | Idiopathic pulmonary fibrosis | Idiopathic pulmonary fibrosis \|\| \|\| | TRUE | igd | 0.0039 | 3.18E-13 | exposure | TRUE | reported | MpDfko | textfile | 2 | TRUE |
|  | 221 | rs7254892 | G | A | G | A | 0.0884 | 7.53E-05 | 0.031391532 | 0.968651 | FALSE | FALSE | FALSE | ebi-a-GCST90018120 | 19 | 45389596 | 0.000332055 | 437235 | 0.82 | Idiopathic pulmonary fibrosis \|\| id:ebi-a-GCST90018120 | Idiopathic pulmonary fibrosis | Idiopathic pulmonary fibrosis \|\| \|\| | TRUE | igd | 0.0066 | 1.64E-40 | exposure | TRUE | reported | MpDfko | textfile | 2 | TRUE |
|  | 222 | rs72654472 | G | T | G | T | 0.0737 | -1.76E-05 | 0.026787357 | 0.973029 | FALSE | FALSE | FALSE | ebi-a-GCST90018120 | 19 | 45414392 | 0.00035691 | 437235 | 0.96 | Idiopathic pulmonary fibrosis \|\| id:ebi-a-GCST90018120 | Idiopathic pulmonary fibrosis | Idiopathic pulmonary fibrosis \|\| \|\| | TRUE | igd | 0.0075 | 9.35E-23 | exposure | TRUE | reported | MpDfko | textfile | 2 | TRUE |
|  | 223 | rs72667426 | A | G | A | G | -0.0488 | -0.000128439 | 0.025804852 | 0.974438 | FALSE | FALSE | FALSE | ebi-a-GCST90018120 | 1 | 66481640 | 0.000366075 | 437235 | 0.73 | Idiopathic pulmonary fibrosis \|\| id:ebi-a-GCST90018120 | Idiopathic pulmonary fibrosis | Idiopathic pulmonary fibrosis \|\| \|\| | TRUE | igd | 0.0077 | 1.90E-10 | exposure | TRUE | reported | MpDfko | textfile | 2 | TRUE |
|  | 224 | rs72679119 | C | G | C | G | -0.0357 | 9.73E-05 | 0.133232921 | 0.132741 | FALSE | TRUE | FALSE | ebi-a-GCST90018120 | 1 | 66617553 | 0.00017051 | 437235 | 0.57 | Idiopathic pulmonary fibrosis \|\| id:ebi-a-GCST90018120 | Idiopathic pulmonary fibrosis | Idiopathic pulmonary fibrosis \|\| \|\| | TRUE | igd | 0.0036 | 1.06E-23 | exposure | TRUE | reported | MpDfko | textfile | 2 | TRUE |
|  | 225 | rs72685056 | G | A | G | A | -0.0506 | -0.000135119 | 0.128436018 | 0.871643 | FALSE | FALSE | FALSE | ebi-a-GCST90018120 | 1 | 66397344 | 0.000172965 | 437235 | 0.44 | Idiopathic pulmonary fibrosis \|\| id:ebi-a-GCST90018120 | Idiopathic pulmonary fibrosis | Idiopathic pulmonary fibrosis \|\| \|\| | TRUE | igd | 0.0036 | 2.76E-44 | exposure | TRUE | reported | MpDfko | textfile | 2 | TRUE |
|  | 226 | rs72698555 | C | T | C | T | -0.0538 | 0.000142761 | 0.079713651 | 0.920302 | FALSE | FALSE | FALSE | ebi-a-GCST90018120 | 1 | 159605749 | 0.000213351 | 437235 | 0.5 | Idiopathic pulmonary fibrosis \|\| id:ebi-a-GCST90018120 | Idiopathic pulmonary fibrosis | Idiopathic pulmonary fibrosis \|\| \|\| | TRUE | igd | 0.0044 | 2.44E-34 | exposure | TRUE | reported | MpDfko | textfile | 2 | TRUE |
|  | 227 | rs72698573 | T | C | T | C | -0.09 | 2.53E-06 | 0.085691292 | 0.914362 | FALSE | FALSE | FALSE | ebi-a-GCST90018120 | 1 | 159670967 | 0.000206714 | 437235 | 0.99 | Idiopathic pulmonary fibrosis \|\| id:ebi-a-GCST90018120 | Idiopathic pulmonary fibrosis | Idiopathic pulmonary fibrosis \|\| \|\| | TRUE | igd | 0.0043 | 3.55E-97 | exposure | TRUE | reported | MpDfko | textfile | 2 | TRUE |
|  | 228 | rs72743115 | A | C | A | C | -0.0479 | -0.000131101 | 0.041409958 | 0.95851 | FALSE | FALSE | FALSE | ebi-a-GCST90018120 | 15 | 53759611 | 0.000290008 | 437235 | 0.649999 | Idiopathic pulmonary fibrosis \|\| id:ebi-a-GCST90018120 | Idiopathic pulmonary fibrosis | Idiopathic pulmonary fibrosis \|\| \|\| | TRUE | igd | 0.0061 | 2.88E-15 | exposure | TRUE | reported | MpDfko | textfile | 2 | TRUE |
|  | 229 | rs72814468 | T | C | T | C | 0.0277 | -0.000160372 | 0.126974956 | 0.873747 | FALSE | FALSE | FALSE | ebi-a-GCST90018120 | 2 | 28359016 | 0.000173914 | 437235 | 0.36 | Idiopathic pulmonary fibrosis \|\| id:ebi-a-GCST90018120 | Idiopathic pulmonary fibrosis | Idiopathic pulmonary fibrosis \|\| \|\| | TRUE | igd | 0.0036 | 2.32E-14 | exposure | TRUE | reported | MpDfko | textfile | 2 | TRUE |
|  | 230 | rs72961013 | G | A | G | A | -0.0495 | 1.52E-05 | 0.067125985 | 0.933744 | FALSE | FALSE | FALSE | ebi-a-GCST90018120 | 6 | 127529780 | 0.000232431 | 437235 | 0.95 | Idiopathic pulmonary fibrosis \|\| id:ebi-a-GCST90018120 | Idiopathic pulmonary fibrosis | Idiopathic pulmonary fibrosis \|\| \|\| | TRUE | igd | 0.0049 | 2.40E-24 | exposure | TRUE | reported | MpDfko | textfile | 2 | TRUE |
|  | 231 | rs73020704 | A | G | A | G | -0.0582 | 9.97E-05 | 0.019583608 | 0.980394 | FALSE | FALSE | FALSE | ebi-a-GCST90018120 | 1 | 159559088 | 0.000416755 | 437235 | 0.81 | Idiopathic pulmonary fibrosis \|\| id:ebi-a-GCST90018120 | Idiopathic pulmonary fibrosis | Idiopathic pulmonary fibrosis \|\| \|\| | TRUE | igd | 0.0082 | 1.41E-12 | exposure | TRUE | reported | MpDfko | textfile | 2 | TRUE |
|  | 232 | rs7302482 | T | C | T | C | 0.0647 | 0.000314367 | 0.076096022 | 0.92379 | FALSE | FALSE | FALSE | ebi-a-GCST90018120 | 12 | 121052180 | 0.000217873 | 437235 | 0.15 | Idiopathic pulmonary fibrosis \|\| id:ebi-a-GCST90018120 | Idiopathic pulmonary fibrosis | Idiopathic pulmonary fibrosis \|\| \|\| | TRUE | igd | 0.0044 | 1.99E-48 | exposure | TRUE | reported | MpDfko | textfile | 2 | TRUE |
|  | 233 | rs73050293 | A | G | A | G | 0.0223 | 0.000145931 | 0.131601664 | 0.868889 | FALSE | FALSE | FALSE | ebi-a-GCST90018120 | 19 | 45379746 | 0.000173503 | 437235 | 0.4 | Idiopathic pulmonary fibrosis \|\| id:ebi-a-GCST90018120 | Idiopathic pulmonary fibrosis | Idiopathic pulmonary fibrosis \|\| \|\| | TRUE | igd | 0.0038 | 3.33E-09 | exposure | TRUE | reported | MpDfko | textfile | 2 | TRUE |
|  | 234 | rs7305618 | C | T | C | T | 0.0886 | 3.91E-05 | 0.22819264 | 0.770566 | FALSE | FALSE | FALSE | ebi-a-GCST90018120 | 12 | 121402932 | 0.000137638 | 437235 | 0.77 | Idiopathic pulmonary fibrosis \|\| id:ebi-a-GCST90018120 | Idiopathic pulmonary fibrosis | Idiopathic pulmonary fibrosis \|\| \|\| | TRUE | igd | 0.0028 | 1.00E-200 | exposure | TRUE | reported | MpDfko | textfile | 2 | TRUE |
|  | 235 | rs73157142 | G | A | G | A | -0.036 | -0.000194947 | 0.048799273 | 0.95077 | FALSE | FALSE | FALSE | ebi-a-GCST90018120 | 22 | 38933740 | 0.000267066 | 437235 | 0.46 | Idiopathic pulmonary fibrosis \|\| id:ebi-a-GCST90018120 | Idiopathic pulmonary fibrosis | Idiopathic pulmonary fibrosis \|\| \|\| | TRUE | igd | 0.0056 | 1.51E-10 | exposure | TRUE | reported | MpDfko | textfile | 2 | TRUE |
|  | 236 | rs73214144 | G | A | G | A | 0.0364 | 6.32E-05 | 0.133412172 | 0.867361 | FALSE | FALSE | FALSE | ebi-a-GCST90018120 | 12 | 121450881 | 0.000170486 | 437235 | 0.709999 | Idiopathic pulmonary fibrosis \|\| id:ebi-a-GCST90018120 | Idiopathic pulmonary fibrosis | Idiopathic pulmonary fibrosis \|\| \|\| | TRUE | igd | 0.0035 | 7.96E-25 | exposure | TRUE | reported | MpDfko | textfile | 2 | TRUE |
|  | 237 | rs73220272 | T | C | T | C | -0.0267 | -1.76E-05 | 0.087054003 | 0.913011 | FALSE | FALSE | FALSE | ebi-a-GCST90018120 | 12 | 121614227 | 0.000204915 | 437235 | 0.93 | Idiopathic pulmonary fibrosis \|\| id:ebi-a-GCST90018120 | Idiopathic pulmonary fibrosis | Idiopathic pulmonary fibrosis \|\| \|\| | TRUE | igd | 0.0043 | 3.70E-10 | exposure | TRUE | reported | MpDfko | textfile | 2 | TRUE |
|  | 238 | rs73921514 | T | C | T | C | -0.0369 | -2.58E-05 | 0.038695222 | 0.961085 | FALSE | FALSE | FALSE | ebi-a-GCST90018120 | 2 | 27579172 | 0.000299188 | 437235 | 0.93 | Idiopathic pulmonary fibrosis \|\| id:ebi-a-GCST90018120 | Idiopathic pulmonary fibrosis | Idiopathic pulmonary fibrosis \|\| \|\| | TRUE | igd | 0.0061 | 1.21E-09 | exposure | TRUE | reported | MpDfko | textfile | 2 | TRUE |
|  | 239 | rs74541405 | C | T | C | T | 0.0305 | -5.12E-05 | 0.05574267 | 0.943984 | FALSE | FALSE | FALSE | ebi-a-GCST90018120 | 6 | 127179525 | 0.000251688 | 437235 | 0.83 | Idiopathic pulmonary fibrosis \|\| id:ebi-a-GCST90018120 | Idiopathic pulmonary fibrosis | Idiopathic pulmonary fibrosis \|\| \|\| | TRUE | igd | 0.0052 | 4.11E-09 | exposure | TRUE | reported | MpDfko | textfile | 2 | TRUE |
|  | 240 | rs74580701 | A | G | A | G | -0.0383 | 0.000207886 | 0.04289069 | 0.956776 | FALSE | FALSE | FALSE | ebi-a-GCST90018120 | 6 | 127000881 | 0.000283211 | 437235 | 0.46 | Idiopathic pulmonary fibrosis \|\| id:ebi-a-GCST90018120 | Idiopathic pulmonary fibrosis | Idiopathic pulmonary fibrosis \|\| \|\| | TRUE | igd | 0.0059 | 1.16E-10 | exposure | TRUE | reported | MpDfko | textfile | 2 | TRUE |
|  | 241 | rs74587498 | T | C | T | C | -0.0356 | -0.000309189 | 0.052617916 | 0.947359 | FALSE | FALSE | FALSE | ebi-a-GCST90018120 | 17 | 76388343 | 0.00025877 | 437235 | 0.23 | Idiopathic pulmonary fibrosis \|\| id:ebi-a-GCST90018120 | Idiopathic pulmonary fibrosis | Idiopathic pulmonary fibrosis \|\| \|\| | TRUE | igd | 0.0054 | 4.02E-11 | exposure | TRUE | reported | MpDfko | textfile | 2 | TRUE |
|  | 242 | rs74607435 | T | C | T | C | 0.0447 | -0.000379183 | 0.051531678 | 0.948747 | FALSE | FALSE | FALSE | ebi-a-GCST90018120 | 19 | 45235700 | 0.000262439 | 437235 | 0.15 | Idiopathic pulmonary fibrosis \|\| id:ebi-a-GCST90018120 | Idiopathic pulmonary fibrosis | Idiopathic pulmonary fibrosis \|\| \|\| | TRUE | igd | 0.0055 | 3.42E-16 | exposure | TRUE | reported | MpDfko | textfile | 2 | TRUE |
|  | 243 | rs74663951 | G | A | G | A | 0.0507 | 0.000191199 | 0.024805932 | 0.975179 | FALSE | FALSE | FALSE | ebi-a-GCST90018120 | 12 | 121559410 | 0.000371175 | 437235 | 0.61 | Idiopathic pulmonary fibrosis \|\| id:ebi-a-GCST90018120 | Idiopathic pulmonary fibrosis | Idiopathic pulmonary fibrosis \|\| \|\| | TRUE | igd | 0.0078 | 6.19E-11 | exposure | TRUE | reported | MpDfko | textfile | 2 | TRUE |
|  | 244 | rs74876709 | G | A | G | A | 0.0795 | 0.000237468 | 0.018297218 | 0.981595 | FALSE | FALSE | FALSE | ebi-a-GCST90018120 | 12 | 121450013 | 0.000430349 | 437235 | 0.58 | Idiopathic pulmonary fibrosis \|\| id:ebi-a-GCST90018120 | Idiopathic pulmonary fibrosis | Idiopathic pulmonary fibrosis \|\| \|\| | TRUE | igd | 0.009 | 1.13E-18 | exposure | TRUE | reported | MpDfko | textfile | 2 | TRUE |
|  | 245 | rs75089355 | A | G | A | G | 0.0309 | 0.000276544 | 0.145831167 | 0.854446 | FALSE | FALSE | FALSE | ebi-a-GCST90018120 | 12 | 121673182 | 0.000163752 | 437235 | 0.0909997 | Idiopathic pulmonary fibrosis \|\| id:ebi-a-GCST90018120 | Idiopathic pulmonary fibrosis | Idiopathic pulmonary fibrosis \|\| \|\| | TRUE | igd | 0.0034 | 7.15E-20 | exposure | TRUE | reported | MpDfko | textfile | 2 | TRUE |
|  | 246 | rs75120785 | T | G | T | G | 0.0294 | 8.15E-05 | 0.101517672 | 0.897823 | FALSE | FALSE | FALSE | ebi-a-GCST90018120 | 8 | 9206342 | 0.000190949 | 437235 | 0.67 | Idiopathic pulmonary fibrosis \|\| id:ebi-a-GCST90018120 | Idiopathic pulmonary fibrosis | Idiopathic pulmonary fibrosis \|\| \|\| | TRUE | igd | 0.004 | 1.54E-13 | exposure | TRUE | reported | MpDfko | textfile | 2 | TRUE |
|  | 247 | rs75148473 | T | C | T | C | -0.0438 | -0.000715682 | 0.033310727 | 0.966913 | FALSE | FALSE | FALSE | ebi-a-GCST90018120 | 1 | 65884783 | 0.000323131 | 437235 | 0.0269998 | Idiopathic pulmonary fibrosis \|\| id:ebi-a-GCST90018120 | Idiopathic pulmonary fibrosis | Idiopathic pulmonary fibrosis \|\| \|\| | TRUE | igd | 0.0068 | 9.39E-11 | exposure | TRUE | reported | MpDfko | textfile | 2 | TRUE |
|  | 248 | rs75164063 | G | A | G | A | -0.0512 | 0.000166295 | 0.024239874 | 0.975112 | FALSE | FALSE | FALSE | ebi-a-GCST90018120 | 1 | 154036161 | 0.000370975 | 437235 | 0.66 | Idiopathic pulmonary fibrosis \|\| id:ebi-a-GCST90018120 | Idiopathic pulmonary fibrosis | Idiopathic pulmonary fibrosis \|\| \|\| | TRUE | igd | 0.0078 | 6.90E-11 | exposure | TRUE | reported | MpDfko | textfile | 2 | TRUE |
|  | 249 | rs7546735 | T | C | T | C | 0.0165 | -3.28E-05 | 0.274638164 | 0.723891 | FALSE | FALSE | FALSE | ebi-a-GCST90018120 | 1 | 39550621 | 0.000129416 | 437235 | 0.79 | Idiopathic pulmonary fibrosis \|\| id:ebi-a-GCST90018120 | Idiopathic pulmonary fibrosis | Idiopathic pulmonary fibrosis \|\| \|\| | TRUE | igd | 0.0027 | 1.19E-09 | exposure | TRUE | reported | MpDfko | textfile | 2 | TRUE |
|  | 250 | rs75564621 | G | T | G | T | -0.0274 | 9.91E-05 | 0.082354898 | 0.917723 | FALSE | FALSE | FALSE | ebi-a-GCST90018120 | 1 | 91546448 | 0.000210275 | 437235 | 0.64 | Idiopathic pulmonary fibrosis \|\| id:ebi-a-GCST90018120 | Idiopathic pulmonary fibrosis | Idiopathic pulmonary fibrosis \|\| \|\| | TRUE | igd | 0.0044 | 3.60E-10 | exposure | TRUE | reported | MpDfko | textfile | 2 | TRUE |
|  | 251 | rs7563362 | A | G | A | G | 0.0208 | -2.99E-05 | 0.15202 | 0.142447 | FALSE | FALSE | FALSE | ebi-a-GCST90018120 | 2 | 620297 | 0.000166251 | 437235 | 0.86 | Idiopathic pulmonary fibrosis \|\| id:ebi-a-GCST90018120 | Idiopathic pulmonary fibrosis | Idiopathic pulmonary fibrosis \|\| \|\| | TRUE | igd | 0.0034 | 8.23E-10 | exposure | TRUE | reported | MpDfko | textfile | 2 | TRUE |
|  | 252 | rs7573232 | C | T | C | T | 0.0202 | -4.20E-05 | 0.326391925 | 0.673247 | FALSE | FALSE | FALSE | ebi-a-GCST90018120 | 2 | 214040425 | 0.000123199 | 437235 | 0.73 | Idiopathic pulmonary fibrosis \|\| id:ebi-a-GCST90018120 | Idiopathic pulmonary fibrosis | Idiopathic pulmonary fibrosis \|\| \|\| | TRUE | igd | 0.0026 | 2.67E-15 | exposure | TRUE | reported | MpDfko | textfile | 2 | TRUE |
|  | 253 | rs75880130 | C | T | C | T | -0.0264 | 2.19E-05 | 0.082400592 | 0.917076 | FALSE | FALSE | FALSE | ebi-a-GCST90018120 | 18 | 55097852 | 0.000209662 | 437235 | 0.92 | Idiopathic pulmonary fibrosis \|\| id:ebi-a-GCST90018120 | Idiopathic pulmonary fibrosis | Idiopathic pulmonary fibrosis \|\| \|\| | TRUE | igd | 0.0043 | 6.98E-10 | exposure | TRUE | reported | MpDfko | textfile | 2 | TRUE |
|  | 254 | rs75976622 | T | A | T | A | -0.0386 | 0.000409505 | 0.0407901 | 0.039135 | FALSE | TRUE | FALSE | ebi-a-GCST90018120 | 5 | 156842034 | 0.000299853 | 437235 | 0.17 | Idiopathic pulmonary fibrosis \|\| id:ebi-a-GCST90018120 | Idiopathic pulmonary fibrosis | Idiopathic pulmonary fibrosis \|\| \|\| | TRUE | igd | 0.0061 | 2.52E-10 | exposure | TRUE | reported | MpDfko | textfile | 2 | TRUE |
|  | 255 | rs7602171 | A | G | A | G | -0.0155 | 0.00013394 | 0.328445913 | 0.327605 | FALSE | FALSE | FALSE | ebi-a-GCST90018120 | 2 | 169880178 | 0.000123234 | 437235 | 0.28 | Idiopathic pulmonary fibrosis \|\| id:ebi-a-GCST90018120 | Idiopathic pulmonary fibrosis | Idiopathic pulmonary fibrosis \|\| \|\| | TRUE | igd | 0.0025 | 1.10E-09 | exposure | TRUE | reported | MpDfko | textfile | 2 | TRUE |
|  | 256 | rs76164683 | G | C | G | C | 0.0592 | 3.27E-05 | 0.018856074 | 0.018745 | FALSE | TRUE | FALSE | ebi-a-GCST90018120 | 2 | 27547247 | 0.000426322 | 437235 | 0.94 | Idiopathic pulmonary fibrosis \|\| id:ebi-a-GCST90018120 | Idiopathic pulmonary fibrosis | Idiopathic pulmonary fibrosis \|\| \|\| | TRUE | igd | 0.0089 | 3.06E-11 | exposure | TRUE | reported | MpDfko | textfile | 2 | TRUE |
|  | 257 | rs76263497 | G | A | G | A | 0.0238 | 0.000290267 | 0.157955604 | 0.843547 | FALSE | FALSE | FALSE | ebi-a-GCST90018120 | 15 | 60878804 | 0.000159167 | 437235 | 0.0680002 | Idiopathic pulmonary fibrosis \|\| id:ebi-a-GCST90018120 | Idiopathic pulmonary fibrosis | Idiopathic pulmonary fibrosis \|\| \|\| | TRUE | igd | 0.0033 | 8.90E-13 | exposure | TRUE | reported | MpDfko | textfile | 2 | TRUE |
|  | 258 | rs76365233 | C | G | C | G | 0.0247 | -3.49E-05 | 0.090740113 | 0.090611 | FALSE | TRUE | FALSE | ebi-a-GCST90018120 | 12 | 12885173 | 0.00020134 | 437235 | 0.86 | Idiopathic pulmonary fibrosis \|\| id:ebi-a-GCST90018120 | Idiopathic pulmonary fibrosis | Idiopathic pulmonary fibrosis \|\| \|\| | TRUE | igd | 0.0042 | 4.28E-09 | exposure | TRUE | reported | MpDfko | textfile | 2 | TRUE |
|  | 259 | rs76366838 | G | A | G | A | -0.2294 | 0.000233471 | 0.026210309 | 0.973973 | FALSE | FALSE | FALSE | ebi-a-GCST90018120 | 19 | 45399896 | 0.000364628 | 437235 | 0.52 | Idiopathic pulmonary fibrosis \|\| id:ebi-a-GCST90018120 | Idiopathic pulmonary fibrosis | Idiopathic pulmonary fibrosis \|\| \|\| | TRUE | igd | 0.0078 | 2.17E-191 | exposure | TRUE | reported | MpDfko | textfile | 2 | TRUE |
|  | 260 | rs76419583 | T | C | T | C | -0.0229 | -0.000258312 | 0.154182215 | 0.846526 | FALSE | FALSE | FALSE | ebi-a-GCST90018120 | 19 | 45217859 | 0.000160547 | 437235 | 0.11 | Idiopathic pulmonary fibrosis \|\| id:ebi-a-GCST90018120 | Idiopathic pulmonary fibrosis | Idiopathic pulmonary fibrosis \|\| \|\| | TRUE | igd | 0.0033 | 4.66E-12 | exposure | TRUE | reported | MpDfko | textfile | 2 | TRUE |
|  | 261 | rs76475417 | G | A | G | A | -0.05 | 0.000572195 | 0.028800541 | 0.971089 | FALSE | FALSE | FALSE | ebi-a-GCST90018120 | 12 | 120870774 | 0.00034456 | 437235 | 0.0969996 | Idiopathic pulmonary fibrosis \|\| id:ebi-a-GCST90018120 | Idiopathic pulmonary fibrosis | Idiopathic pulmonary fibrosis \|\| \|\| | TRUE | igd | 0.0071 | 1.50E-12 | exposure | TRUE | reported | MpDfko | textfile | 2 | TRUE |
|  | 262 | rs76476582 | C | T | C | T | 0.0347 | -6.40E-07 | 0.055557205 | 0.944278 | FALSE | FALSE | FALSE | ebi-a-GCST90018120 | 2 | 27594397 | 0.000251871 | 437235 | 1 | Idiopathic pulmonary fibrosis \|\| id:ebi-a-GCST90018120 | Idiopathic pulmonary fibrosis | Idiopathic pulmonary fibrosis \|\| \|\| | TRUE | igd | 0.0053 | 5.51E-11 | exposure | TRUE | reported | MpDfko | textfile | 2 | TRUE |
|  | 263 | rs76769546 | G | A | G | A | -0.0342 | 0.000196024 | 0.048256831 | 0.95153 | FALSE | FALSE | FALSE | ebi-a-GCST90018120 | 1 | 159623545 | 0.000269085 | 437235 | 0.46 | Idiopathic pulmonary fibrosis \|\| id:ebi-a-GCST90018120 | Idiopathic pulmonary fibrosis | Idiopathic pulmonary fibrosis \|\| \|\| | TRUE | igd | 0.0056 | 1.19E-09 | exposure | TRUE | reported | MpDfko | textfile | 2 | TRUE |
|  | 264 | rs76841017 | T | C | T | C | -0.0561 | 4.65E-05 | 0.028522511 | 0.971491 | FALSE | FALSE | FALSE | ebi-a-GCST90018120 | 12 | 121090195 | 0.000346587 | 437235 | 0.9 | Idiopathic pulmonary fibrosis \|\| id:ebi-a-GCST90018120 | Idiopathic pulmonary fibrosis | Idiopathic pulmonary fibrosis \|\| \|\| | TRUE | igd | 0.0073 | 1.12E-14 | exposure | TRUE | reported | MpDfko | textfile | 2 | TRUE |
|  | 265 | rs76856627 | A | G | A | G | -0.0567 | -0.000865719 | 0.033602586 | 0.966731 | FALSE | FALSE | FALSE | ebi-a-GCST90018120 | 19 | 45543755 | 0.000322636 | 437235 | 0.00729995 | Idiopathic pulmonary fibrosis \|\| id:ebi-a-GCST90018120 | Idiopathic pulmonary fibrosis | Idiopathic pulmonary fibrosis \|\| \|\| | TRUE | igd | 0.0068 | 5.07E-17 | exposure | TRUE | reported | MpDfko | textfile | 2 | TRUE |
|  | 266 | rs76870318 | G | A | G | A | 0.056 | 0.00025827 | 0.020628264 | 0.979619 | FALSE | FALSE | FALSE | ebi-a-GCST90018120 | 2 | 27933601 | 0.000409154 | 437235 | 0.53 | Idiopathic pulmonary fibrosis \|\| id:ebi-a-GCST90018120 | Idiopathic pulmonary fibrosis | Idiopathic pulmonary fibrosis \|\| \|\| | TRUE | igd | 0.0085 | 5.49E-11 | exposure | TRUE | reported | MpDfko | textfile | 2 | TRUE |
|  | 267 | rs76898938 | G | A | G | A | 0.0447 | 0.000414142 | 0.02608937 | 0.974186 | FALSE | FALSE | FALSE | ebi-a-GCST90018120 | 2 | 27539201 | 0.000363726 | 437235 | 0.25 | Idiopathic pulmonary fibrosis \|\| id:ebi-a-GCST90018120 | Idiopathic pulmonary fibrosis | Idiopathic pulmonary fibrosis \|\| \|\| | TRUE | igd | 0.0076 | 3.78E-09 | exposure | TRUE | reported | MpDfko | textfile | 2 | TRUE |
|  | 268 | rs76912411 | T | A | T | A | -0.0655 | -0.000271602 | 0.079515009 | 0.079023 | FALSE | TRUE | FALSE | ebi-a-GCST90018120 | 1 | 66040934 | 0.000214555 | 437235 | 0.21 | Idiopathic pulmonary fibrosis \|\| id:ebi-a-GCST90018120 | Idiopathic pulmonary fibrosis | Idiopathic pulmonary fibrosis \|\| \|\| | TRUE | igd | 0.0045 | 1.58E-48 | exposure | TRUE | reported | MpDfko | textfile | 2 | TRUE |
|  | 269 | rs769449 | G | A | G | A | -0.2593 | 6.07E-05 | 0.126903251 | 0.873953 | FALSE | FALSE | FALSE | ebi-a-GCST90018120 | 19 | 45410002 | 0.000174214 | 437235 | 0.73 | Idiopathic pulmonary fibrosis \|\| id:ebi-a-GCST90018120 | Idiopathic pulmonary fibrosis | Idiopathic pulmonary fibrosis \|\| \|\| | TRUE | igd | 0.0036 | 1.00E-200 | exposure | TRUE | reported | MpDfko | textfile | 2 | TRUE |
|  | 270 | rs769450 | G | A | G | A | 0.0449 | -0.000151338 | 0.39615452 | 0.603798 | FALSE | FALSE | FALSE | ebi-a-GCST90018120 | 19 | 45410444 | 0.000118171 | 437235 | 0.2 | Idiopathic pulmonary fibrosis \|\| id:ebi-a-GCST90018120 | Idiopathic pulmonary fibrosis | Idiopathic pulmonary fibrosis \|\| \|\| | TRUE | igd | 0.0024 | 4.33E-76 | exposure | TRUE | reported | MpDfko | textfile | 2 | TRUE |
|  | 271 | rs76962533 | C | T | C | T | -0.067 | 0.000257318 | 0.037851964 | 0.962026 | FALSE | FALSE | FALSE | ebi-a-GCST90018120 | 1 | 66194165 | 0.000302448 | 437235 | 0.4 | Idiopathic pulmonary fibrosis \|\| id:ebi-a-GCST90018120 | Idiopathic pulmonary fibrosis | Idiopathic pulmonary fibrosis \|\| \|\| | TRUE | igd | 0.0063 | 2.60E-26 | exposure | TRUE | reported | MpDfko | textfile | 2 | TRUE |
|  | 272 | rs77273543 | C | G | C | G | 0.0497 | -0.000352343 | 0.05010843 | 0.050466 | FALSE | TRUE | FALSE | ebi-a-GCST90018120 | 1 | 65976098 | 0.000264184 | 437235 | 0.18 | Idiopathic pulmonary fibrosis \|\| id:ebi-a-GCST90018120 | Idiopathic pulmonary fibrosis | Idiopathic pulmonary fibrosis \|\| \|\| | TRUE | igd | 0.0055 | 2.79E-19 | exposure | TRUE | reported | MpDfko | textfile | 2 | TRUE |
|  | 273 | rs77383163 | T | C | T | C | 0.0375 | -0.000272682 | 0.084650964 | 0.91551 | FALSE | FALSE | FALSE | ebi-a-GCST90018120 | 1 | 159282664 | 0.00020785 | 437235 | 0.19 | Idiopathic pulmonary fibrosis \|\| id:ebi-a-GCST90018120 | Idiopathic pulmonary fibrosis | Idiopathic pulmonary fibrosis \|\| \|\| | TRUE | igd | 0.0043 | 4.92E-18 | exposure | TRUE | reported | MpDfko | textfile | 2 | TRUE |
|  | 274 | rs77451629 | G | A | G | A | 0.0445 | 6.81E-05 | 0.028935364 | 0.970784 | FALSE | FALSE | FALSE | ebi-a-GCST90018120 | 1 | 65911519 | 0.000342693 | 437235 | 0.84 | Idiopathic pulmonary fibrosis \|\| id:ebi-a-GCST90018120 | Idiopathic pulmonary fibrosis | Idiopathic pulmonary fibrosis \|\| \|\| | TRUE | igd | 0.0072 | 7.04E-10 | exposure | TRUE | reported | MpDfko | textfile | 2 | TRUE |
|  | 275 | rs77509028 | G | T | G | T | 0.0615 | -0.000389249 | 0.015364326 | 0.984273 | FALSE | FALSE | FALSE | ebi-a-GCST90018120 | 1 | 159740174 | 0.000464202 | 437235 | 0.4 | Idiopathic pulmonary fibrosis \|\| id:ebi-a-GCST90018120 | Idiopathic pulmonary fibrosis | Idiopathic pulmonary fibrosis \|\| \|\| | TRUE | igd | 0.0097 | 2.67E-10 | exposure | TRUE | reported | MpDfko | textfile | 2 | TRUE |
|  | 276 | rs77532628 | T | C | T | C | -0.0366 | -0.000339334 | 0.051094369 | 0.948753 | FALSE | FALSE | FALSE | ebi-a-GCST90018120 | 2 | 27907444 | 0.000262278 | 437235 | 0.2 | Idiopathic pulmonary fibrosis \|\| id:ebi-a-GCST90018120 | Idiopathic pulmonary fibrosis | Idiopathic pulmonary fibrosis \|\| \|\| | TRUE | igd | 0.0054 | 1.55E-11 | exposure | TRUE | reported | MpDfko | textfile | 2 | TRUE |
|  | 277 | rs77617917 | G | A | G | A | -0.0284 | 7.53E-05 | 0.079561641 | 0.920569 | FALSE | FALSE | FALSE | ebi-a-GCST90018120 | 20 | 44563217 | 0.000213643 | 437235 | 0.719999 | Idiopathic pulmonary fibrosis \|\| id:ebi-a-GCST90018120 | Idiopathic pulmonary fibrosis | Idiopathic pulmonary fibrosis \|\| \|\| | TRUE | igd | 0.0045 | 1.80E-10 | exposure | TRUE | reported | MpDfko | textfile | 2 | TRUE |
|  | 278 | rs77704739 | T | C | T | C | -0.0532 | -0.000372281 | 0.041979102 | 0.957831 | FALSE | FALSE | FALSE | ebi-a-GCST90018120 | 5 | 52080909 | 0.000288379 | 437235 | 0.2 | Idiopathic pulmonary fibrosis \|\| id:ebi-a-GCST90018120 | Idiopathic pulmonary fibrosis | Idiopathic pulmonary fibrosis \|\| \|\| | TRUE | igd | 0.006 | 7.27E-19 | exposure | TRUE | reported | MpDfko | textfile | 2 | TRUE |
|  | 279 | rs77801962 | T | C | T | C | -0.0643 | -0.000211548 | 0.029667388 | 0.970569 | FALSE | FALSE | FALSE | ebi-a-GCST90018120 | 1 | 154566932 | 0.000342092 | 437235 | 0.54 | Idiopathic pulmonary fibrosis \|\| id:ebi-a-GCST90018120 | Idiopathic pulmonary fibrosis | Idiopathic pulmonary fibrosis \|\| \|\| | TRUE | igd | 0.0071 | 1.96E-19 | exposure | TRUE | reported | MpDfko | textfile | 2 | TRUE |
|  | 280 | rs77828979 | T | C | T | C | -0.0252 | 0.000295618 | 0.133298607 | 0.867461 | FALSE | FALSE | FALSE | ebi-a-GCST90018120 | 11 | 49855634 | 0.000170452 | 437235 | 0.0830004 | Idiopathic pulmonary fibrosis \|\| id:ebi-a-GCST90018120 | Idiopathic pulmonary fibrosis | Idiopathic pulmonary fibrosis \|\| \|\| | TRUE | igd | 0.0036 | 2.40E-12 | exposure | TRUE | reported | MpDfko | textfile | 2 | TRUE |
|  | 281 | rs77993403 | G | A | G | A | -0.0415 | 7.79E-07 | 0.048200386 | 0.952378 | FALSE | FALSE | FALSE | ebi-a-GCST90018120 | 1 | 154316996 | 0.000271527 | 437235 | 1 | Idiopathic pulmonary fibrosis \|\| id:ebi-a-GCST90018120 | Idiopathic pulmonary fibrosis | Idiopathic pulmonary fibrosis \|\| \|\| | TRUE | igd | 0.0057 | 2.16E-13 | exposure | TRUE | reported | MpDfko | textfile | 2 | TRUE |
|  | 282 | rs77994054 | C | T | C | T | -0.0522 | 0.000311514 | 0.01924427 | 0.981156 | FALSE | FALSE | FALSE | ebi-a-GCST90018120 | 1 | 154699197 | 0.000424585 | 437235 | 0.46 | Idiopathic pulmonary fibrosis \|\| id:ebi-a-GCST90018120 | Idiopathic pulmonary fibrosis | Idiopathic pulmonary fibrosis \|\| \|\| | TRUE | igd | 0.0088 | 3.51E-09 | exposure | TRUE | reported | MpDfko | textfile | 2 | TRUE |
|  | 283 | rs77994623 | C | T | C | T | 0.0476 | -0.000346804 | 0.166217429 | 0.833097 | FALSE | FALSE | FALSE | ebi-a-GCST90018120 | 1 | 154505106 | 0.000154952 | 437235 | 0.025 | Idiopathic pulmonary fibrosis \|\| id:ebi-a-GCST90018120 | Idiopathic pulmonary fibrosis | Idiopathic pulmonary fibrosis \|\| \|\| | TRUE | igd | 0.0032 | 2.90E-50 | exposure | TRUE | reported | MpDfko | textfile | 2 | TRUE |
|  | 284 | rs780094 | T | C | T | C | -0.0734 | -4.46E-05 | 0.381034641 | 0.383266 | FALSE | FALSE | FALSE | ebi-a-GCST90018120 | 2 | 27741237 | 0.000118872 | 437235 | 0.709999 | Idiopathic pulmonary fibrosis \|\| id:ebi-a-GCST90018120 | Idiopathic pulmonary fibrosis | Idiopathic pulmonary fibrosis \|\| \|\| | TRUE | igd | 0.0025 | 1.88E-196 | exposure | TRUE | reported | MpDfko | textfile | 2 | TRUE |
|  | 285 | rs78038982 | G | T | G | T | 0.0609 | 0.00069894 | 0.017091099 | 0.982381 | FALSE | FALSE | FALSE | ebi-a-GCST90018120 | 1 | 154539156 | 0.000439494 | 437235 | 0.11 | Idiopathic pulmonary fibrosis \|\| id:ebi-a-GCST90018120 | Idiopathic pulmonary fibrosis | Idiopathic pulmonary fibrosis \|\| \|\| | TRUE | igd | 0.0092 | 3.78E-11 | exposure | TRUE | reported | MpDfko | textfile | 2 | TRUE |
|  | 286 | rs78248443 | C | T | C | T | 0.0363 | -0.000519034 | 0.047297819 | 0.952943 | FALSE | FALSE | FALSE | ebi-a-GCST90018120 | 1 | 65155172 | 0.000272686 | 437235 | 0.0569994 | Idiopathic pulmonary fibrosis \|\| id:ebi-a-GCST90018120 | Idiopathic pulmonary fibrosis | Idiopathic pulmonary fibrosis \|\| \|\| | TRUE | igd | 0.0057 | 1.93E-10 | exposure | TRUE | reported | MpDfko | textfile | 2 | TRUE |
|  | 287 | rs7845566 | T | C | T | C | -0.0191 | -7.29E-05 | 0.169097267 | 0.830916 | FALSE | FALSE | FALSE | ebi-a-GCST90018120 | 8 | 11653150 | 0.000154153 | 437235 | 0.64 | Idiopathic pulmonary fibrosis \|\| id:ebi-a-GCST90018120 | Idiopathic pulmonary fibrosis | Idiopathic pulmonary fibrosis \|\| \|\| | TRUE | igd | 0.0032 | 2.67E-09 | exposure | TRUE | reported | MpDfko | textfile | 2 | TRUE |
|  | 288 | rs78494072 | G | A | G | A | -0.0498 | 8.29E-05 | 0.02647568 | 0.973699 | FALSE | FALSE | FALSE | ebi-a-GCST90018120 | 1 | 66281595 | 0.000360651 | 437235 | 0.82 | Idiopathic pulmonary fibrosis \|\| id:ebi-a-GCST90018120 | Idiopathic pulmonary fibrosis | Idiopathic pulmonary fibrosis \|\| \|\| | TRUE | igd | 0.0076 | 4.53E-11 | exposure | TRUE | reported | MpDfko | textfile | 2 | TRUE |
|  | 289 | rs79018068 | A | G | A | G | -0.0413 | -0.000663609 | 0.034421322 | 0.96556 | FALSE | FALSE | FALSE | ebi-a-GCST90018120 | 19 | 45634854 | 0.000317123 | 437235 | 0.0369999 | Idiopathic pulmonary fibrosis \|\| id:ebi-a-GCST90018120 | Idiopathic pulmonary fibrosis | Idiopathic pulmonary fibrosis \|\| \|\| | TRUE | igd | 0.0065 | 2.16E-10 | exposure | TRUE | reported | MpDfko | textfile | 2 | TRUE |
|  | 290 | rs79219014 | G | T | G | T | -0.0847 | 2.67E-05 | 0.027236149 | 0.972646 | FALSE | FALSE | FALSE | ebi-a-GCST90018120 | 1 | 154415675 | 0.000355187 | 437235 | 0.94 | Idiopathic pulmonary fibrosis \|\| id:ebi-a-GCST90018120 | Idiopathic pulmonary fibrosis | Idiopathic pulmonary fibrosis \|\| \|\| | TRUE | igd | 0.0075 | 7.34E-30 | exposure | TRUE | reported | MpDfko | textfile | 2 | TRUE |
|  | 291 | rs79429216 | G | A | G | A | -0.0769 | -0.000324378 | 0.012359157 | 0.987675 | FALSE | FALSE | FALSE | ebi-a-GCST90018120 | 19 | 45445517 | 0.000523346 | 437235 | 0.54 | Idiopathic pulmonary fibrosis \|\| id:ebi-a-GCST90018120 | Idiopathic pulmonary fibrosis | Idiopathic pulmonary fibrosis \|\| \|\| | TRUE | igd | 0.0106 | 3.20E-13 | exposure | TRUE | reported | MpDfko | textfile | 2 | TRUE |
|  | 292 | rs79468673 | A | G | A | G | 0.0556 | 0.000236742 | 0.034749483 | 0.964639 | FALSE | FALSE | FALSE | ebi-a-GCST90018120 | 2 | 28254894 | 0.00031315 | 437235 | 0.450001 | Idiopathic pulmonary fibrosis \|\| id:ebi-a-GCST90018120 | Idiopathic pulmonary fibrosis | Idiopathic pulmonary fibrosis \|\| \|\| | TRUE | igd | 0.0066 | 2.77E-17 | exposure | TRUE | reported | MpDfko | textfile | 2 | TRUE |
|  | 293 | rs79634415 | C | T | C | T | -0.026 | -0.000202761 | 0.088161986 | 0.91143 | FALSE | FALSE | FALSE | ebi-a-GCST90018120 | 8 | 126344793 | 0.000203462 | 437235 | 0.32 | Idiopathic pulmonary fibrosis \|\| id:ebi-a-GCST90018120 | Idiopathic pulmonary fibrosis | Idiopathic pulmonary fibrosis \|\| \|\| | TRUE | igd | 0.0042 | 4.17E-10 | exposure | TRUE | reported | MpDfko | textfile | 2 | TRUE |
|  | 294 | rs7976660 | C | T | C | T | 0.0209 | -8.75E-05 | 0.269084996 | 0.730561 | FALSE | FALSE | FALSE | ebi-a-GCST90018120 | 12 | 121070011 | 0.000130401 | 437235 | 0.5 | Idiopathic pulmonary fibrosis \|\| id:ebi-a-GCST90018120 | Idiopathic pulmonary fibrosis | Idiopathic pulmonary fibrosis \|\| \|\| | TRUE | igd | 0.0027 | 6.21E-15 | exposure | TRUE | reported | MpDfko | textfile | 2 | TRUE |
|  | 295 | rs80051818 | T | C | T | C | -0.0401 | -5.12E-05 | 0.326054618 | 0.676747 | FALSE | FALSE | FALSE | ebi-a-GCST90018120 | 2 | 27591804 | 0.000123533 | 437235 | 0.68 | Idiopathic pulmonary fibrosis \|\| id:ebi-a-GCST90018120 | Idiopathic pulmonary fibrosis | Idiopathic pulmonary fibrosis \|\| \|\| | TRUE | igd | 0.0026 | 4.62E-55 | exposure | TRUE | reported | MpDfko | textfile | 2 | TRUE |
|  | 296 | rs800980 | C | T | C | T | -0.0211 | 0.000139592 | 0.161434 | 0.8351 | FALSE | FALSE | FALSE | ebi-a-GCST90018120 | 7 | 74196244 | 0.000159252 | 437235 | 0.38 | Idiopathic pulmonary fibrosis \|\| id:ebi-a-GCST90018120 | Idiopathic pulmonary fibrosis | Idiopathic pulmonary fibrosis \|\| \|\| | TRUE | igd | 0.0033 | 1.35E-10 | exposure | TRUE | reported | MpDfko | textfile | 2 | TRUE |
|  | 297 | rs80255322 | A | G | A | G | 0.0707 | -0.00021482 | 0.036670209 | 0.963246 | FALSE | FALSE | FALSE | ebi-a-GCST90018120 | 1 | 66519663 | 0.000306995 | 437235 | 0.48 | Idiopathic pulmonary fibrosis \|\| id:ebi-a-GCST90018120 | Idiopathic pulmonary fibrosis | Idiopathic pulmonary fibrosis \|\| \|\| | TRUE | igd | 0.0064 | 3.71E-28 | exposure | TRUE | reported | MpDfko | textfile | 2 | TRUE |
|  | 298 | rs80257887 | G | A | G | A | -0.0476 | 0.000205597 | 0.03585206 | 0.964084 | FALSE | FALSE | FALSE | ebi-a-GCST90018120 | 19 | 45020859 | 0.000310553 | 437235 | 0.51 | Idiopathic pulmonary fibrosis \|\| id:ebi-a-GCST90018120 | Idiopathic pulmonary fibrosis | Idiopathic pulmonary fibrosis \|\| \|\| | TRUE | igd | 0.0065 | 2.33E-13 | exposure | TRUE | reported | MpDfko | textfile | 2 | TRUE |
|  | 299 | rs80341247 | G | A | G | A | 0.0397 | 0.000285609 | 0.09516357 | 0.905245 | FALSE | FALSE | FALSE | ebi-a-GCST90018120 | 12 | 121704150 | 0.000197178 | 437235 | 0.15 | Idiopathic pulmonary fibrosis \|\| id:ebi-a-GCST90018120 | Idiopathic pulmonary fibrosis | Idiopathic pulmonary fibrosis \|\| \|\| | TRUE | igd | 0.0041 | 6.54E-22 | exposure | TRUE | reported | MpDfko | textfile | 2 | TRUE |
|  | 300 | rs8060025 | T | G | T | G | -0.0187 | 4.74E-06 | 0.389349 | 0.38841 | FALSE | FALSE | FALSE | ebi-a-GCST90018120 | 16 | 27327214 | 0.00011869 | 437235 | 0.97 | Idiopathic pulmonary fibrosis \|\| id:ebi-a-GCST90018120 | Idiopathic pulmonary fibrosis | Idiopathic pulmonary fibrosis \|\| \|\| | TRUE | igd | 0.0024 | 1.59E-14 | exposure | TRUE | reported | MpDfko | textfile | 2 | TRUE |
|  | 301 | rs8180849 | G | A | G | A | -0.0226 | 0.000258131 | 0.13288036 | 0.866474 | FALSE | FALSE | FALSE | ebi-a-GCST90018120 | 7 | 22859259 | 0.000169863 | 437235 | 0.13 | Idiopathic pulmonary fibrosis \|\| id:ebi-a-GCST90018120 | Idiopathic pulmonary fibrosis | Idiopathic pulmonary fibrosis \|\| \|\| | TRUE | igd | 0.0035 | 1.28E-10 | exposure | TRUE | reported | MpDfko | textfile | 2 | TRUE |
|  | 302 | rs862994 | G | A | G | A | -0.0353 | 8.65E-06 | 0.268826573 | 0.268623 | FALSE | FALSE | FALSE | ebi-a-GCST90018120 | 1 | 159165336 | 0.000130453 | 437235 | 0.95 | Idiopathic pulmonary fibrosis \|\| id:ebi-a-GCST90018120 | Idiopathic pulmonary fibrosis | Idiopathic pulmonary fibrosis \|\| \|\| | TRUE | igd | 0.0027 | 1.73E-39 | exposure | TRUE | reported | MpDfko | textfile | 2 | TRUE |
|  | 303 | rs880632 | C | A | C | A | -0.0171 | -7.74E-05 | 0.294027156 | 0.707978 | FALSE | FALSE | FALSE | ebi-a-GCST90018120 | 8 | 11735939 | 0.000127322 | 437235 | 0.54 | Idiopathic pulmonary fibrosis \|\| id:ebi-a-GCST90018120 | Idiopathic pulmonary fibrosis | Idiopathic pulmonary fibrosis \|\| \|\| | TRUE | igd | 0.0027 | 1.24E-10 | exposure | TRUE | reported | MpDfko | textfile | 2 | TRUE |
|  | 304 | rs9521499 | T | C | T | C | 0.0143 | 0.000104764 | 0.415988 | 0.584798 | FALSE | FALSE | FALSE | ebi-a-GCST90018120 | 13 | 110393185 | 0.000118182 | 437235 | 0.37 | Idiopathic pulmonary fibrosis \|\| id:ebi-a-GCST90018120 | Idiopathic pulmonary fibrosis | Idiopathic pulmonary fibrosis \|\| \|\| | TRUE | igd | 0.0024 | 3.63E-09 | exposure | TRUE | reported | MpDfko | textfile | 2 | TRUE |
|  | 305 | rs9604045 | G | T | G | T | -0.0229 | 0.000101622 | 0.264431 | 0.748649 | FALSE | FALSE | FALSE | ebi-a-GCST90018120 | 13 | 113927208 | 0.00013894 | 437235 | 0.47 | Idiopathic pulmonary fibrosis \|\| id:ebi-a-GCST90018120 | Idiopathic pulmonary fibrosis | Idiopathic pulmonary fibrosis \|\| \|\| | TRUE | igd | 0.0029 | 3.85E-15 | exposure | TRUE | reported | MpDfko | textfile | 2 | TRUE |
|  | 306 | rs9738365 | C | A | C | A | 0.0203 | -0.000199505 | 0.266574 | 0.733827 | FALSE | FALSE | FALSE | ebi-a-GCST90018120 | 12 | 31997635 | 0.000131148 | 437235 | 0.13 | Idiopathic pulmonary fibrosis \|\| id:ebi-a-GCST90018120 | Idiopathic pulmonary fibrosis | Idiopathic pulmonary fibrosis \|\| \|\| | TRUE | igd | 0.0027 | 4.93E-14 | exposure | TRUE | reported | MpDfko | textfile | 2 | TRUE |
|  | 307 | rs983309 | T | G | T | G | 0.0606 | -0.000153797 | 0.118058296 | 0.117136 | FALSE | FALSE | FALSE | ebi-a-GCST90018120 | 8 | 9177732 | 0.000179925 | 437235 | 0.39 | Idiopathic pulmonary fibrosis \|\| id:ebi-a-GCST90018120 | Idiopathic pulmonary fibrosis | Idiopathic pulmonary fibrosis \|\| \|\| | TRUE | igd | 0.0037 | 5.16E-61 | exposure | TRUE | reported | MpDfko | textfile | 2 | TRUE |
|  |  |  |  |  |  |  |  |  |  |  |  |  |  |  |  |  |  |  |  |  |  |  |  |  |  |  |  |  |  |  |  |  |  |
| CXCL9 |  | SNP | effect_allele.exposure | other_allele.exposure | effect_allele.outcome | other_allele.outcome | beta.exposure | beta.outcome | eaf.exposure | eaf.outcome | remove | palindromic | ambiguous | id.outcome | chr | pos | se.outcome | samplesize.outcome | pval.outcome | outcome | originalname.outcome | outcome.deprecated | mr_keep.outcome | data_source.outcome | se.exposure | pval.exposure | exposure | mr_keep.exposure | pval_origin.exposure | id.exposure | data_source.exposure | action | mr_keep |
|  | 1 | rs111607343 | G | A | G | A | 0.521 | 3.08E-06 | 0.9622 | 0.967177 | FALSE | FALSE | FALSE | ebi-a-GCST90018120 | 19 | 897855 | 0.000333947 | 437235 | 0.99 | Idiopathic pulmonary fibrosis \|\| id:ebi-a-GCST90018120 | Idiopathic pulmonary fibrosis | Idiopathic pulmonary fibrosis \|\| \|\| | TRUE | igd | 0.1119 | 2.83E-06 | exposure | TRUE | reported | 60Iuhy | textfile | 2 | TRUE |
|  | 2 | rs11177248 | G | A | G | A | -0.3073 | -9.76E-05 | 0.9394 | 0.934542 | FALSE | FALSE | FALSE | ebi-a-GCST90018120 | 12 | 68875886 | 0.00023505 | 437235 | 0.68 | Idiopathic pulmonary fibrosis \|\| id:ebi-a-GCST90018120 | Idiopathic pulmonary fibrosis | Idiopathic pulmonary fibrosis \|\| \|\| | TRUE | igd | 0.067 | 4.45E-06 | exposure | TRUE | reported | 60Iuhy | textfile | 2 | TRUE |
|  | 3 | rs112337562 | G | T | G | T | 0.37 | -0.000511293 | 0.0169 | 0.011926 | FALSE | FALSE | FALSE | ebi-a-GCST90018120 | 14 | 93131570 | 0.000565811 | 437235 | 0.37 | Idiopathic pulmonary fibrosis \|\| id:ebi-a-GCST90018120 | Idiopathic pulmonary fibrosis | Idiopathic pulmonary fibrosis \|\| \|\| | TRUE | igd | 0.0796 | 2.98E-06 | exposure | TRUE | reported | 60Iuhy | textfile | 2 | TRUE |
|  | 4 | rs112861654 | G | A | G | A | 0.2765 | 0.000105676 | 0.0865 | 0.086618 | FALSE | FALSE | FALSE | ebi-a-GCST90018120 | 21 | 43599172 | 0.000206012 | 437235 | 0.61 | Idiopathic pulmonary fibrosis \|\| id:ebi-a-GCST90018120 | Idiopathic pulmonary fibrosis | Idiopathic pulmonary fibrosis \|\| \|\| | TRUE | igd | 0.0529 | 1.81E-07 | exposure | TRUE | reported | 60Iuhy | textfile | 2 | TRUE |
|  | 5 | rs117831247 | C | T | C | T | 0.8334 | -0.00049459 | 0.9811 | 0.988369 | FALSE | FALSE | FALSE | ebi-a-GCST90018120 | 10 | 68501839 | 0.000542727 | 437235 | 0.36 | Idiopathic pulmonary fibrosis \|\| id:ebi-a-GCST90018120 | Idiopathic pulmonary fibrosis | Idiopathic pulmonary fibrosis \|\| \|\| | TRUE | igd | 0.1754 | 2.16E-06 | exposure | TRUE | reported | 60Iuhy | textfile | 2 | TRUE |
|  | 6 | rs139010077 | C | T | C | T | -0.4322 | 0.00018372 | 0.9891 | 0.98612 | FALSE | FALSE | FALSE | ebi-a-GCST90018120 | 3 | 170336148 | 0.0005034 | 437235 | 0.709999 | Idiopathic pulmonary fibrosis \|\| id:ebi-a-GCST90018120 | Idiopathic pulmonary fibrosis | Idiopathic pulmonary fibrosis \|\| \|\| | TRUE | igd | 0.095 | 3.55E-06 | exposure | TRUE | reported | 60Iuhy | textfile | 2 | TRUE |
|  | 7 | rs1796086 | C | T | C | T | 0.2096 | -0.000394426 | 0.0944 | 0.085586 | FALSE | FALSE | FALSE | ebi-a-GCST90018120 | 7 | 70648715 | 0.00020731 | 437235 | 0.0569994 | Idiopathic pulmonary fibrosis \|\| id:ebi-a-GCST90018120 | Idiopathic pulmonary fibrosis | Idiopathic pulmonary fibrosis \|\| \|\| | TRUE | igd | 0.0403 | 2.23E-07 | exposure | TRUE | reported | 60Iuhy | textfile | 2 | TRUE |
|  | 8 | rs41272086 | G | A | G | A | 0.2226 | -4.57E-05 | 0.9145 | 0.893601 | FALSE | FALSE | FALSE | ebi-a-GCST90018120 | 6 | 161008646 | 0.000187949 | 437235 | 0.81 | Idiopathic pulmonary fibrosis \|\| id:ebi-a-GCST90018120 | Idiopathic pulmonary fibrosis | Idiopathic pulmonary fibrosis \|\| \|\| | TRUE | igd | 0.0415 | 7.43E-08 | exposure | TRUE | reported | 60Iuhy | textfile | 2 | TRUE |
|  | 9 | rs55876513 | G | T | G | T | -0.166 | 6.77E-05 | 0.2475 | 0.243278 | FALSE | FALSE | FALSE | ebi-a-GCST90018120 | 4 | 76883698 | 0.000134721 | 437235 | 0.61 | Idiopathic pulmonary fibrosis \|\| id:ebi-a-GCST90018120 | Idiopathic pulmonary fibrosis | Idiopathic pulmonary fibrosis \|\| \|\| | TRUE | igd | 0.0255 | 8.23E-11 | exposure | TRUE | reported | 60Iuhy | textfile | 2 | TRUE |
|  | 10 | rs5752128 | C | T | C | T | 0.1685 | -0.000160227 | 0.0954 | 0.093295 | FALSE | FALSE | FALSE | ebi-a-GCST90018120 | 22 | 25718623 | 0.000198524 | 437235 | 0.42 | Idiopathic pulmonary fibrosis \|\| id:ebi-a-GCST90018120 | Idiopathic pulmonary fibrosis | Idiopathic pulmonary fibrosis \|\| \|\| | TRUE | igd | 0.0369 | 4.34E-06 | exposure | TRUE | reported | 60Iuhy | textfile | 2 | TRUE |
|  | 11 | rs62562991 | G | A | G | A | -0.6236 | -1.69E-05 | 0.9801 | 0.980905 | FALSE | FALSE | FALSE | ebi-a-GCST90018120 | 9 | 98736059 | 0.000422737 | 437235 | 0.97 | Idiopathic pulmonary fibrosis \|\| id:ebi-a-GCST90018120 | Idiopathic pulmonary fibrosis | Idiopathic pulmonary fibrosis \|\| \|\| | TRUE | igd | 0.126 | 8.40E-07 | exposure | TRUE | reported | 60Iuhy | textfile | 2 | TRUE |
|  | 12 | rs6679677 | C | A | C | A | -0.162 | -0.000288677 | 0.9085 | 0.898171 | FALSE | FALSE | FALSE | ebi-a-GCST90018120 | 1 | 114303808 | 0.00019096 | 437235 | 0.13 | Idiopathic pulmonary fibrosis \|\| id:ebi-a-GCST90018120 | Idiopathic pulmonary fibrosis | Idiopathic pulmonary fibrosis \|\| \|\| | TRUE | igd | 0.0329 | 8.86E-07 | exposure | TRUE | reported | 60Iuhy | textfile | 2 | TRUE |
|  | 13 | rs77086208 | C | T | C | T | -0.3226 | -0.000407671 | 0.9811 | 0.982372 | FALSE | FALSE | FALSE | ebi-a-GCST90018120 | 14 | 70619491 | 0.000444242 | 437235 | 0.36 | Idiopathic pulmonary fibrosis \|\| id:ebi-a-GCST90018120 | Idiopathic pulmonary fibrosis | Idiopathic pulmonary fibrosis \|\| \|\| | TRUE | igd | 0.0698 | 3.83E-06 | exposure | TRUE | reported | 60Iuhy | textfile | 2 | TRUE |
|  | 14 | rs816960 | C | T | C | T | 0.1224 | -6.27E-05 | 0.7406 | 0.775672 | FALSE | FALSE | FALSE | ebi-a-GCST90018120 | 13 | 108522521 | 0.000140982 | 437235 | 0.66 | Idiopathic pulmonary fibrosis \|\| id:ebi-a-GCST90018120 | Idiopathic pulmonary fibrosis | Idiopathic pulmonary fibrosis \|\| \|\| | TRUE | igd | 0.0244 | 5.01E-07 | exposure | TRUE | reported | 60Iuhy | textfile | 2 | TRUE |
|  |  |  |  |  |  |  |  |  |  |  |  |  |  |  |  |  |  |  |  |  |  |  |  |  |  |  |  |  |  |  |  |  |  |
| Eotaxin |  | SNP | effect_allele.exposure | other_allele.exposure | effect_allele.outcome | other_allele.outcome | beta.exposure | beta.outcome | eaf.exposure | eaf.outcome | remove | palindromic | ambiguous | id.outcome | chr | pos | se.outcome | samplesize.outcome | pval.outcome | outcome | originalname.outcome | outcome.deprecated | mr_keep.outcome | data_source.outcome | se.exposure | pval.exposure | exposure | mr_keep.exposure | pval_origin.exposure | id.exposure | data_source.exposure | action | mr_keep |
|  | 1 | rs11087905 | C | A | C | A | -0.0941 | 0.000231218 | 0.6581 | 0.652118 | FALSE | FALSE | FALSE | ebi-a-GCST90018120 | 21 | 25505329 | 0.000137884 | 437235 | 0.0940005 | Idiopathic pulmonary fibrosis \|\| id:ebi-a-GCST90018120 | Idiopathic pulmonary fibrosis | Idiopathic pulmonary fibrosis \|\| \|\| | TRUE | igd | 0.0189 | 5.48E-07 | exposure | TRUE | reported | podQwP | textfile | 2 | TRUE |
|  | 2 | rs112347425 | C | T | C | T | -0.158 | -0.000446383 | 0.8926 | 0.90025 | FALSE | FALSE | FALSE | ebi-a-GCST90018120 | 3 | 46460888 | 0.000195799 | 437235 | 0.0230001 | Idiopathic pulmonary fibrosis \|\| id:ebi-a-GCST90018120 | Idiopathic pulmonary fibrosis | Idiopathic pulmonary fibrosis \|\| \|\| | TRUE | igd | 0.0277 | 8.65E-09 | exposure | TRUE | reported | podQwP | textfile | 2 | TRUE |
|  | 3 | rs12075 | G | A | G | A | -0.1671 | -0.000204697 | 0.3976 | 0.420187 | FALSE | FALSE | FALSE | ebi-a-GCST90018120 | 1 | 159175354 | 0.000117093 | 437235 | 0.08 | Idiopathic pulmonary fibrosis \|\| id:ebi-a-GCST90018120 | Idiopathic pulmonary fibrosis | Idiopathic pulmonary fibrosis \|\| \|\| | TRUE | igd | 0.0156 | 1.33E-26 | exposure | TRUE | reported | podQwP | textfile | 2 | TRUE |
|  | 4 | rs138125931 | C | T | C | T | -0.1292 | -8.27E-05 | 0.8678 | 0.123966 | FALSE | FALSE | FALSE | ebi-a-GCST90018120 | 1 | 91505950 | 0.00017555 | 437235 | 0.64 | Idiopathic pulmonary fibrosis \|\| id:ebi-a-GCST90018120 | Idiopathic pulmonary fibrosis | Idiopathic pulmonary fibrosis \|\| \|\| | TRUE | igd | 0.0254 | 3.59E-07 | exposure | TRUE | reported | podQwP | textfile | 2 | TRUE |
|  | 5 | rs1476670 | C | A | C | A | 0.1007 | 0.000145997 | 0.7932 | 0.789191 | FALSE | FALSE | FALSE | ebi-a-GCST90018120 | 1 | 44508195 | 0.000143182 | 437235 | 0.31 | Idiopathic pulmonary fibrosis \|\| id:ebi-a-GCST90018120 | Idiopathic pulmonary fibrosis | Idiopathic pulmonary fibrosis \|\| \|\| | TRUE | igd | 0.0217 | 3.51E-06 | exposure | TRUE | reported | podQwP | textfile | 2 | TRUE |
|  | 6 | rs2024050 | G | A | G | A | -0.1728 | -0.000254361 | 0.8996 | 0.89555 | FALSE | FALSE | FALSE | ebi-a-GCST90018120 | 7 | 75460393 | 0.000189178 | 437235 | 0.18 | Idiopathic pulmonary fibrosis \|\| id:ebi-a-GCST90018120 | Idiopathic pulmonary fibrosis | Idiopathic pulmonary fibrosis \|\| \|\| | TRUE | igd | 0.0303 | 1.10E-08 | exposure | TRUE | reported | podQwP | textfile | 2 | TRUE |
|  | 7 | rs2210755 | C | T | C | T | 0.1104 | 6.28E-05 | 0.0805 | 0.079718 | FALSE | FALSE | FALSE | ebi-a-GCST90018120 | 9 | 80223823 | 0.000222904 | 437235 | 0.780001 | Idiopathic pulmonary fibrosis \|\| id:ebi-a-GCST90018120 | Idiopathic pulmonary fibrosis | Idiopathic pulmonary fibrosis \|\| \|\| | TRUE | igd | 0.0242 | 4.85E-06 | exposure | TRUE | reported | podQwP | textfile | 2 | TRUE |
|  | 8 | rs2211994 | C | T | C | T | -0.0885 | 8.72E-05 | 0.7495 | 0.735654 | FALSE | FALSE | FALSE | ebi-a-GCST90018120 | 21 | 18047593 | 0.000131316 | 437235 | 0.51 | Idiopathic pulmonary fibrosis \|\| id:ebi-a-GCST90018120 | Idiopathic pulmonary fibrosis | Idiopathic pulmonary fibrosis \|\| \|\| | TRUE | igd | 0.0177 | 6.08E-07 | exposure | TRUE | reported | podQwP | textfile | 2 | TRUE |
|  | 9 | rs2228467 | C | T | C | T | 0.4163 | -4.05E-05 | 0.0696 | 0.061321 | FALSE | FALSE | FALSE | ebi-a-GCST90018120 | 3 | 42906116 | 0.000240582 | 437235 | 0.86 | Idiopathic pulmonary fibrosis \|\| id:ebi-a-GCST90018120 | Idiopathic pulmonary fibrosis | Idiopathic pulmonary fibrosis \|\| \|\| | TRUE | igd | 0.0292 | 2.27E-46 | exposure | TRUE | reported | podQwP | textfile | 2 | TRUE |
|  | 10 | rs2419841 | C | T | C | T | 0.1277 | -4.70E-05 | 0.1193 | 0.102895 | FALSE | FALSE | FALSE | ebi-a-GCST90018120 | 10 | 115335983 | 0.000191309 | 437235 | 0.81 | Idiopathic pulmonary fibrosis \|\| id:ebi-a-GCST90018120 | Idiopathic pulmonary fibrosis | Idiopathic pulmonary fibrosis \|\| \|\| | TRUE | igd | 0.0279 | 4.98E-06 | exposure | TRUE | reported | podQwP | textfile | 2 | TRUE |
|  | 11 | rs5746492 | G | A | G | A | -0.0954 | -0.000124237 | 0.1809 | 0.180113 | FALSE | FALSE | FALSE | ebi-a-GCST90018120 | 22 | 18393933 | 0.00015069 | 437235 | 0.41 | Idiopathic pulmonary fibrosis \|\| id:ebi-a-GCST90018120 | Idiopathic pulmonary fibrosis | Idiopathic pulmonary fibrosis \|\| \|\| | TRUE | igd | 0.0207 | 3.96E-06 | exposure | TRUE | reported | podQwP | textfile | 2 | TRUE |
|  | 12 | rs5754733 | C | A | C | A | 0.1042 | 4.71E-05 | 0.2117 | 0.238188 | FALSE | FALSE | FALSE | ebi-a-GCST90018120 | 22 | 34269594 | 0.000137661 | 437235 | 0.73 | Idiopathic pulmonary fibrosis \|\| id:ebi-a-GCST90018120 | Idiopathic pulmonary fibrosis | Idiopathic pulmonary fibrosis \|\| \|\| | TRUE | igd | 0.0214 | 1.06E-06 | exposure | TRUE | reported | podQwP | textfile | 2 | TRUE |
|  | 13 | rs59808887 | C | T | C | T | 0.1673 | -7.52E-05 | 0.9225 | 0.92165 | FALSE | FALSE | FALSE | ebi-a-GCST90018120 | 5 | 31846520 | 0.000218754 | 437235 | 0.73 | Idiopathic pulmonary fibrosis \|\| id:ebi-a-GCST90018120 | Idiopathic pulmonary fibrosis | Idiopathic pulmonary fibrosis \|\| \|\| | TRUE | igd | 0.0358 | 2.91E-06 | exposure | TRUE | reported | podQwP | textfile | 2 | TRUE |
|  | 14 | rs75426604 | C | A | C | A | 0.1366 | -0.000235221 | 0.8688 | 0.868364 | FALSE | FALSE | FALSE | ebi-a-GCST90018120 | 14 | 35857714 | 0.000171063 | 437235 | 0.17 | Idiopathic pulmonary fibrosis \|\| id:ebi-a-GCST90018120 | Idiopathic pulmonary fibrosis | Idiopathic pulmonary fibrosis \|\| \|\| | TRUE | igd | 0.0291 | 2.53E-06 | exposure | TRUE | reported | podQwP | textfile | 2 | TRUE |
|  | 15 | rs79722574 | C | T | C | T | 0.1113 | -0.000131652 | 0.84 | 0.836573 | FALSE | FALSE | FALSE | ebi-a-GCST90018120 | 17 | 32619052 | 0.000158358 | 437235 | 0.41 | Idiopathic pulmonary fibrosis \|\| id:ebi-a-GCST90018120 | Idiopathic pulmonary fibrosis | Idiopathic pulmonary fibrosis \|\| \|\| | TRUE | igd | 0.0228 | 1.06E-06 | exposure | TRUE | reported | podQwP | textfile | 2 | TRUE |
|  | 16 | rs80341932 | G | A | G | A | -0.1016 | 0.000220309 | 0.2922 | 0.296005 | FALSE | FALSE | FALSE | ebi-a-GCST90018120 | 18 | 56915928 | 0.000127468 | 437235 | 0.0840001 | Idiopathic pulmonary fibrosis \|\| id:ebi-a-GCST90018120 | Idiopathic pulmonary fibrosis | Idiopathic pulmonary fibrosis \|\| \|\| | TRUE | igd | 0.0205 | 6.69E-07 | exposure | TRUE | reported | podQwP | textfile | 2 | TRUE |
|  | 17 | rs9317045 | C | A | C | A | -0.1182 | 0.000551153 | 0.1461 | 0.163189 | FALSE | FALSE | FALSE | ebi-a-GCST90018120 | 13 | 59630038 | 0.000157892 | 437235 | 0.000470002 | Idiopathic pulmonary fibrosis \|\| id:ebi-a-GCST90018120 | Idiopathic pulmonary fibrosis | Idiopathic pulmonary fibrosis \|\| \|\| | TRUE | igd | 0.0237 | 5.82E-07 | exposure | TRUE | reported | podQwP | textfile | 2 | TRUE |
|  |  |  |  |  |  |  |  |  |  |  |  |  |  |  |  |  |  |  |  |  |  |  |  |  |  |  |  |  |  |  |  |  |  |
| GROa |  | SNP | effect_allele.exposure | other_allele.exposure | effect_allele.outcome | other_allele.outcome | beta.exposure | beta.outcome | eaf.exposure | eaf.outcome | remove | palindromic | ambiguous | id.outcome | chr | pos | se.outcome | samplesize.outcome | pval.outcome | outcome | originalname.outcome | outcome.deprecated | mr_keep.outcome | data_source.outcome | se.exposure | pval.exposure | exposure | mr_keep.exposure | pval_origin.exposure | id.exposure | data_source.exposure | action | mr_keep |
|  | 1 | rs1113500 | G | T | G | T | -0.1174 | 0.000132166 | 0.3698 | 0.366549 | FALSE | FALSE | FALSE | ebi-a-GCST90018120 | 1 | 108595442 | 0.000120395 | 437235 | 0.27 | Idiopathic pulmonary fibrosis \|\| id:ebi-a-GCST90018120 | Idiopathic pulmonary fibrosis | Idiopathic pulmonary fibrosis \|\| \|\| | TRUE | igd | 0.0244 | 1.57E-06 | exposure | TRUE | reported | xmxOCr | textfile | 2 | TRUE |
|  | 2 | rs118158560 | G | A | G | A | -0.2703 | -1.63E-05 | 0.9374 | 0.941509 | FALSE | FALSE | FALSE | ebi-a-GCST90018120 | 7 | 38146610 | 0.000246904 | 437235 | 0.95 | Idiopathic pulmonary fibrosis \|\| id:ebi-a-GCST90018120 | Idiopathic pulmonary fibrosis | Idiopathic pulmonary fibrosis \|\| \|\| | TRUE | igd | 0.0594 | 3.42E-06 | exposure | TRUE | reported | xmxOCr | textfile | 2 | TRUE |
|  | 3 | rs12075 | G | A | G | A | -0.3751 | -0.000204697 | 0.3976 | 0.420187 | FALSE | FALSE | FALSE | ebi-a-GCST90018120 | 1 | 159175354 | 0.000117093 | 437235 | 0.08 | Idiopathic pulmonary fibrosis \|\| id:ebi-a-GCST90018120 | Idiopathic pulmonary fibrosis | Idiopathic pulmonary fibrosis \|\| \|\| | TRUE | igd | 0.0237 | 1.24E-55 | exposure | TRUE | reported | xmxOCr | textfile | 2 | TRUE |
|  | 4 | rs140734053 | G | A | G | A | -0.7257 | 0.000107591 | 0.9751 | 0.982456 | FALSE | FALSE | FALSE | ebi-a-GCST90018120 | 10 | 5401459 | 0.00044624 | 437235 | 0.81 | Idiopathic pulmonary fibrosis \|\| id:ebi-a-GCST90018120 | Idiopathic pulmonary fibrosis | Idiopathic pulmonary fibrosis \|\| \|\| | TRUE | igd | 0.1561 | 3.58E-06 | exposure | TRUE | reported | xmxOCr | textfile | 2 | TRUE |
|  | 5 | rs185768063 | G | A | G | A | -0.3998 | -0.000646791 | 0.0139 | 0.009302 | FALSE | FALSE | FALSE | ebi-a-GCST90018120 | 6 | 16494983 | 0.0006243 | 437235 | 0.3 | Idiopathic pulmonary fibrosis \|\| id:ebi-a-GCST90018120 | Idiopathic pulmonary fibrosis | Idiopathic pulmonary fibrosis \|\| \|\| | TRUE | igd | 0.076 | 1.46E-07 | exposure | TRUE | reported | xmxOCr | textfile | 2 | TRUE |
|  | 6 | rs188345231 | C | T | C | T | -0.623 | 0.000281224 | 0.9881 | 0.980392 | FALSE | FALSE | FALSE | ebi-a-GCST90018120 | 8 | 41437350 | 0.000423811 | 437235 | 0.51 | Idiopathic pulmonary fibrosis \|\| id:ebi-a-GCST90018120 | Idiopathic pulmonary fibrosis | Idiopathic pulmonary fibrosis \|\| \|\| | TRUE | igd | 0.1323 | 4.34E-06 | exposure | TRUE | reported | xmxOCr | textfile | 2 | TRUE |
|  | 7 | rs508977 | G | T | G | T | 0.3802 | -5.00E-05 | 0.2336 | 0.237285 | FALSE | FALSE | FALSE | ebi-a-GCST90018120 | 4 | 74762383 | 0.00013591 | 437235 | 0.709999 | Idiopathic pulmonary fibrosis \|\| id:ebi-a-GCST90018120 | Idiopathic pulmonary fibrosis | Idiopathic pulmonary fibrosis \|\| \|\| | TRUE | igd | 0.028 | 7.56E-42 | exposure | TRUE | reported | xmxOCr | textfile | 2 | TRUE |
|  | 8 | rs62024303 | G | A | G | A | 0.3053 | 5.32E-05 | 0.0457 | 0.043528 | FALSE | FALSE | FALSE | ebi-a-GCST90018120 | 15 | 88871162 | 0.000283549 | 437235 | 0.85 | Idiopathic pulmonary fibrosis \|\| id:ebi-a-GCST90018120 | Idiopathic pulmonary fibrosis | Idiopathic pulmonary fibrosis \|\| \|\| | TRUE | igd | 0.0666 | 4.41E-06 | exposure | TRUE | reported | xmxOCr | textfile | 2 | TRUE |
|  | 9 | rs78653452 | G | T | G | T | 0.7362 | -0.000244747 | 0.9881 | 0.987248 | FALSE | FALSE | FALSE | ebi-a-GCST90018120 | 20 | 9762055 | 0.000517646 | 437235 | 0.64 | Idiopathic pulmonary fibrosis \|\| id:ebi-a-GCST90018120 | Idiopathic pulmonary fibrosis | Idiopathic pulmonary fibrosis \|\| \|\| | TRUE | igd | 0.1558 | 1.21E-06 | exposure | TRUE | reported | xmxOCr | textfile | 2 | TRUE |
|  |  |  |  |  |  |  |  |  |  |  |  |  |  |  |  |  |  |  |  |  |  |  |  |  |  |  |  |  |  |  |  |  |  |
| MCP1 |  | SNP | effect_allele.exposure | other_allele.exposure | effect_allele.outcome | other_allele.outcome | beta.exposure | beta.outcome | eaf.exposure | eaf.outcome | remove | palindromic | ambiguous | id.outcome | chr | pos | se.outcome | samplesize.outcome | pval.outcome | outcome | originalname.outcome | outcome.deprecated | mr_keep.outcome | data_source.outcome | se.exposure | pval.exposure | exposure | mr_keep.exposure | pval_origin.exposure | id.exposure | data_source.exposure | action | mr_keep |
|  | 1 | rs10145849 | G | A | G | A | 0.0755 | 3.59E-06 | 0.6511 | 0.626834 | FALSE | FALSE | FALSE | ebi-a-GCST90018120 | 14 | 82941991 | 0.000118867 | 437235 | 0.98 | Idiopathic pulmonary fibrosis \|\| id:ebi-a-GCST90018120 | Idiopathic pulmonary fibrosis | Idiopathic pulmonary fibrosis \|\| \|\| | TRUE | igd | 0.0162 | 3.41E-06 | exposure | TRUE | reported | 5ovFYV | textfile | 2 | TRUE |
|  | 2 | rs10744620 | C | T | C | T | -0.0788 | -6.72E-05 | 0.6392 | 0.610413 | FALSE | FALSE | FALSE | ebi-a-GCST90018120 | 12 | 3739094 | 0.000119738 | 437235 | 0.57 | Idiopathic pulmonary fibrosis \|\| id:ebi-a-GCST90018120 | Idiopathic pulmonary fibrosis | Idiopathic pulmonary fibrosis \|\| \|\| | TRUE | igd | 0.0161 | 9.91E-07 | exposure | TRUE | reported | 5ovFYV | textfile | 2 | TRUE |
|  | 3 | rs111995966 | G | T | G | T | -0.1452 | -0.000132138 | 0.0258 | 0.020152 | FALSE | FALSE | FALSE | ebi-a-GCST90018120 | 2 | 109174969 | 0.000428317 | 437235 | 0.760001 | Idiopathic pulmonary fibrosis \|\| id:ebi-a-GCST90018120 | Idiopathic pulmonary fibrosis | Idiopathic pulmonary fibrosis \|\| \|\| | TRUE | igd | 0.031 | 2.53E-06 | exposure | TRUE | reported | 5ovFYV | textfile | 2 | TRUE |
|  | 4 | rs12073356 | G | A | G | A | 0.1426 | 4.37E-05 | 0.9145 | 0.937427 | FALSE | FALSE | FALSE | ebi-a-GCST90018120 | 1 | 208007848 | 0.000239008 | 437235 | 0.85 | Idiopathic pulmonary fibrosis \|\| id:ebi-a-GCST90018120 | Idiopathic pulmonary fibrosis | Idiopathic pulmonary fibrosis \|\| \|\| | TRUE | igd | 0.0311 | 4.17E-06 | exposure | TRUE | reported | 5ovFYV | textfile | 2 | TRUE |
|  | 5 | rs12075 | G | A | G | A | -0.2185 | -0.000204697 | 0.3976 | 0.420187 | FALSE | FALSE | FALSE | ebi-a-GCST90018120 | 1 | 159175354 | 0.000117093 | 437235 | 0.08 | Idiopathic pulmonary fibrosis \|\| id:ebi-a-GCST90018120 | Idiopathic pulmonary fibrosis | Idiopathic pulmonary fibrosis \|\| \|\| | TRUE | igd | 0.0155 | 1.44E-44 | exposure | TRUE | reported | 5ovFYV | textfile | 2 | TRUE |
|  | 6 | rs146522229 | C | T | C | T | 0.5976 | -0.000307939 | 0.9761 | 0.98786 | FALSE | FALSE | FALSE | ebi-a-GCST90018120 | 19 | 47798480 | 0.000531206 | 437235 | 0.56 | Idiopathic pulmonary fibrosis \|\| id:ebi-a-GCST90018120 | Idiopathic pulmonary fibrosis | Idiopathic pulmonary fibrosis \|\| \|\| | TRUE | igd | 0.1177 | 3.56E-07 | exposure | TRUE | reported | 5ovFYV | textfile | 2 | TRUE |
|  | 7 | rs2036297 | G | A | G | A | -0.119 | 6.96E-05 | 0.6571 | 0.64713 | FALSE | FALSE | FALSE | ebi-a-GCST90018120 | 3 | 46172903 | 0.000121638 | 437235 | 0.57 | Idiopathic pulmonary fibrosis \|\| id:ebi-a-GCST90018120 | Idiopathic pulmonary fibrosis | Idiopathic pulmonary fibrosis \|\| \|\| | TRUE | igd | 0.016 | 1.09E-13 | exposure | TRUE | reported | 5ovFYV | textfile | 2 | TRUE |
|  | 8 | rs2288370 | C | T | C | T | 0.1031 | -0.00013958 | 0.3956 | 0.370584 | FALSE | FALSE | FALSE | ebi-a-GCST90018120 | 3 | 42977436 | 0.000119933 | 437235 | 0.24 | Idiopathic pulmonary fibrosis \|\| id:ebi-a-GCST90018120 | Idiopathic pulmonary fibrosis | Idiopathic pulmonary fibrosis \|\| \|\| | TRUE | igd | 0.0163 | 2.25E-10 | exposure | TRUE | reported | 5ovFYV | textfile | 2 | TRUE |
|  | 9 | rs2712431 | C | A | C | A | 0.0787 | -8.22E-05 | 0.2853 | 0.315442 | FALSE | FALSE | FALSE | ebi-a-GCST90018120 | 3 | 128316890 | 0.000124201 | 437235 | 0.51 | Idiopathic pulmonary fibrosis \|\| id:ebi-a-GCST90018120 | Idiopathic pulmonary fibrosis | Idiopathic pulmonary fibrosis \|\| \|\| | TRUE | igd | 0.0172 | 4.76E-06 | exposure | TRUE | reported | 5ovFYV | textfile | 2 | TRUE |
|  | 10 | rs56212190 | C | T | C | T | -0.181 | -0.000328981 | 0.9453 | 0.954918 | FALSE | FALSE | FALSE | ebi-a-GCST90018120 | 1 | 42168539 | 0.000282701 | 437235 | 0.24 | Idiopathic pulmonary fibrosis \|\| id:ebi-a-GCST90018120 | Idiopathic pulmonary fibrosis | Idiopathic pulmonary fibrosis \|\| \|\| | TRUE | igd | 0.0373 | 9.85E-07 | exposure | TRUE | reported | 5ovFYV | textfile | 2 | TRUE |
|  | 11 | rs7197349 | G | A | G | A | -0.0968 | 0.000222989 | 0.1372 | 0.127702 | FALSE | FALSE | FALSE | ebi-a-GCST90018120 | 16 | 78687219 | 0.000175308 | 437235 | 0.2 | Idiopathic pulmonary fibrosis \|\| id:ebi-a-GCST90018120 | Idiopathic pulmonary fibrosis | Idiopathic pulmonary fibrosis \|\| \|\| | TRUE | igd | 0.0206 | 2.62E-06 | exposure | TRUE | reported | 5ovFYV | textfile | 2 | TRUE |
|  | 12 | rs7517040 | G | A | G | A | 0.0987 | 0.000309068 | 0.7177 | 0.735833 | FALSE | FALSE | FALSE | ebi-a-GCST90018120 | 1 | 158859133 | 0.000132493 | 437235 | 0.0189998 | Idiopathic pulmonary fibrosis \|\| id:ebi-a-GCST90018120 | Idiopathic pulmonary fibrosis | Idiopathic pulmonary fibrosis \|\| \|\| | TRUE | igd | 0.0191 | 2.44E-07 | exposure | TRUE | reported | 5ovFYV | textfile | 2 | TRUE |
|  | 13 | rs7632755 | G | A | G | A | -0.2938 | -0.000206003 | 0.9195 | 0.930844 | FALSE | FALSE | FALSE | ebi-a-GCST90018120 | 3 | 46332382 | 0.000228455 | 437235 | 0.37 | Idiopathic pulmonary fibrosis \|\| id:ebi-a-GCST90018120 | Idiopathic pulmonary fibrosis | Idiopathic pulmonary fibrosis \|\| \|\| | TRUE | igd | 0.0316 | 1.18E-20 | exposure | TRUE | reported | 5ovFYV | textfile | 2 | TRUE |
|  | 14 | rs9317045 | C | A | C | A | -0.1134 | 0.000551153 | 0.1461 | 0.163189 | FALSE | FALSE | FALSE | ebi-a-GCST90018120 | 13 | 59630038 | 0.000157892 | 437235 | 0.000470002 | Idiopathic pulmonary fibrosis \|\| id:ebi-a-GCST90018120 | Idiopathic pulmonary fibrosis | Idiopathic pulmonary fibrosis \|\| \|\| | TRUE | igd | 0.0236 | 1.52E-06 | exposure | TRUE | reported | 5ovFYV | textfile | 2 | TRUE |
|  |  |  |  |  |  |  |  |  |  |  |  |  |  |  |  |  |  |  |  |  |  |  |  |  |  |  |  |  |  |  |  |  |  |
| MIF |  | SNP | effect_allele.exposure | other_allele.exposure | effect_allele.outcome | other_allele.outcome | beta.exposure | beta.outcome | eaf.exposure | eaf.outcome | remove | palindromic | ambiguous | id.outcome | chr | pos | se.outcome | samplesize.outcome | pval.outcome | outcome | originalname.outcome | outcome.deprecated | mr_keep.outcome | data_source.outcome | se.exposure | pval.exposure | exposure | mr_keep.exposure | pval_origin.exposure | id.exposure | data_source.exposure | action | mr_keep |
|  | 1 | rs113218956 | G | A | G | A | 0.8948 | -2.11E-05 | 0.9891 | 0.995735 | FALSE | FALSE | FALSE | ebi-a-GCST90018120 | 22 | 25224834 | 0.000918424 | 437235 | 0.98 | Idiopathic pulmonary fibrosis \|\| id:ebi-a-GCST90018120 | Idiopathic pulmonary fibrosis | Idiopathic pulmonary fibrosis \|\| \|\| | TRUE | igd | 0.1879 | 2.26E-06 | exposure | TRUE | reported | jqNI68 | textfile | 2 | TRUE |
|  | 2 | rs118055855 | C | T | C | T | -0.6907 | 0.0006322 | 0.0129 | 0.015369 | FALSE | FALSE | FALSE | ebi-a-GCST90018120 | 11 | 29888572 | 0.000471801 | 437235 | 0.18 | Idiopathic pulmonary fibrosis \|\| id:ebi-a-GCST90018120 | Idiopathic pulmonary fibrosis | Idiopathic pulmonary fibrosis \|\| \|\| | TRUE | igd | 0.15 | 4.13E-06 | exposure | TRUE | reported | jqNI68 | textfile | 2 | TRUE |
|  | 3 | rs12594190 | G | A | G | A | -0.1355 | 4.58E-05 | 0.2465 | 0.222736 | FALSE | FALSE | FALSE | ebi-a-GCST90018120 | 15 | 25036455 | 0.000138735 | 437235 | 0.74 | Idiopathic pulmonary fibrosis \|\| id:ebi-a-GCST90018120 | Idiopathic pulmonary fibrosis | Idiopathic pulmonary fibrosis \|\| \|\| | TRUE | igd | 0.0267 | 3.70E-07 | exposure | TRUE | reported | jqNI68 | textfile | 2 | TRUE |
|  | 4 | rs13142904 | C | T | C | T | 0.223 | 0.000100861 | 0.9314 | 0.930409 | FALSE | FALSE | FALSE | ebi-a-GCST90018120 | 4 | 54318414 | 0.000231147 | 437235 | 0.66 | Idiopathic pulmonary fibrosis \|\| id:ebi-a-GCST90018120 | Idiopathic pulmonary fibrosis | Idiopathic pulmonary fibrosis \|\| \|\| | TRUE | igd | 0.0425 | 2.56E-07 | exposure | TRUE | reported | jqNI68 | textfile | 2 | TRUE |
|  | 5 | rs141009259 | C | T | C | T | 0.6178 | 0.000595916 | 0.0129 | 0.011843 | FALSE | FALSE | FALSE | ebi-a-GCST90018120 | 2 | 207976283 | 0.000554025 | 437235 | 0.28 | Idiopathic pulmonary fibrosis \|\| id:ebi-a-GCST90018120 | Idiopathic pulmonary fibrosis | Idiopathic pulmonary fibrosis \|\| \|\| | TRUE | igd | 0.1322 | 2.47E-06 | exposure | TRUE | reported | jqNI68 | textfile | 2 | TRUE |
|  | 6 | rs5751777 | C | T | C | T | 0.1342 | -0.000119302 | 0.3887 | 0.413076 | FALSE | FALSE | FALSE | ebi-a-GCST90018120 | 22 | 24267047 | 0.000117398 | 437235 | 0.31 | Idiopathic pulmonary fibrosis \|\| id:ebi-a-GCST90018120 | Idiopathic pulmonary fibrosis | Idiopathic pulmonary fibrosis \|\| \|\| | TRUE | igd | 0.0249 | 7.09E-08 | exposure | TRUE | reported | jqNI68 | textfile | 2 | TRUE |
|  | 7 | rs78098071 | C | T | C | T | 0.4867 | 0.000277787 | 0.0189 | 0.012 | FALSE | FALSE | FALSE | ebi-a-GCST90018120 | 5 | 163309739 | 0.000536318 | 437235 | 0.6 | Idiopathic pulmonary fibrosis \|\| id:ebi-a-GCST90018120 | Idiopathic pulmonary fibrosis | Idiopathic pulmonary fibrosis \|\| \|\| | TRUE | igd | 0.0918 | 1.78E-07 | exposure | TRUE | reported | jqNI68 | textfile | 2 | TRUE |
|  |  |  |  |  |  |  |  |  |  |  |  |  |  |  |  |  |  |  |  |  |  |  |  |  |  |  |  |  |  |  |  |  |  |
| MIP1a |  | SNP | effect_allele.exposure | other_allele.exposure | effect_allele.outcome | other_allele.outcome | beta.exposure | beta.outcome | eaf.exposure | eaf.outcome | remove | palindromic | ambiguous | id.outcome | chr | pos | se.outcome | samplesize.outcome | pval.outcome | outcome | originalname.outcome | outcome.deprecated | mr_keep.outcome | data_source.outcome | se.exposure | pval.exposure | exposure | mr_keep.exposure | pval_origin.exposure | id.exposure | data_source.exposure | action | mr_keep |
|  | 1 | rs10835056 | G | T | G | T | -0.1194 | 4.25E-05 | 0.7555 | 0.740949 | FALSE | FALSE | FALSE | ebi-a-GCST90018120 | 11 | 26697017 | 0.000132649 | 437235 | 0.75 | Idiopathic pulmonary fibrosis \|\| id:ebi-a-GCST90018120 | Idiopathic pulmonary fibrosis | Idiopathic pulmonary fibrosis \|\| \|\| | TRUE | igd | 0.0254 | 2.60E-06 | exposure | TRUE | reported | lEqYIj | textfile | 2 | TRUE |
|  | 2 | rs116615337 | G | A | G | A | -0.1278 | 6.98E-06 | 0.2913 | 0.283829 | FALSE | FALSE | FALSE | ebi-a-GCST90018120 | 1 | 193533132 | 0.000135396 | 437235 | 0.96 | Idiopathic pulmonary fibrosis \|\| id:ebi-a-GCST90018120 | Idiopathic pulmonary fibrosis | Idiopathic pulmonary fibrosis \|\| \|\| | TRUE | igd | 0.0279 | 4.82E-06 | exposure | TRUE | reported | lEqYIj | textfile | 2 | TRUE |
|  | 3 | rs12690897 | G | A | G | A | -0.1248 | -8.99E-06 | 0.7187 | 0.740452 | FALSE | FALSE | FALSE | ebi-a-GCST90018120 | 7 | 85346177 | 0.000132913 | 437235 | 0.94 | Idiopathic pulmonary fibrosis \|\| id:ebi-a-GCST90018120 | Idiopathic pulmonary fibrosis | Idiopathic pulmonary fibrosis \|\| \|\| | TRUE | igd | 0.0262 | 2.11E-06 | exposure | TRUE | reported | lEqYIj | textfile | 2 | TRUE |
|  | 4 | rs184154340 | G | A | G | A | -0.331 | -9.32E-05 | 0.9592 | 0.955913 | FALSE | FALSE | FALSE | ebi-a-GCST90018120 | 11 | 80502036 | 0.000283267 | 437235 | 0.74 | Idiopathic pulmonary fibrosis \|\| id:ebi-a-GCST90018120 | Idiopathic pulmonary fibrosis | Idiopathic pulmonary fibrosis \|\| \|\| | TRUE | igd | 0.0693 | 1.86E-06 | exposure | TRUE | reported | lEqYIj | textfile | 2 | TRUE |
|  | 5 | rs34771762 | G | A | G | A | -0.249 | -1.79E-05 | 0.0736 | 0.067784 | FALSE | FALSE | FALSE | ebi-a-GCST90018120 | 2 | 201412655 | 0.000229862 | 437235 | 0.94 | Idiopathic pulmonary fibrosis \|\| id:ebi-a-GCST90018120 | Idiopathic pulmonary fibrosis | Idiopathic pulmonary fibrosis \|\| \|\| | TRUE | igd | 0.0523 | 2.13E-06 | exposure | TRUE | reported | lEqYIj | textfile | 2 | TRUE |
|  | 6 | rs57786342 | G | A | G | A | -0.1314 | 0.000198898 | 0.7843 | 0.796534 | FALSE | FALSE | FALSE | ebi-a-GCST90018120 | 14 | 69260028 | 0.000144085 | 437235 | 0.17 | Idiopathic pulmonary fibrosis \|\| id:ebi-a-GCST90018120 | Idiopathic pulmonary fibrosis | Idiopathic pulmonary fibrosis \|\| \|\| | TRUE | igd | 0.0285 | 4.06E-06 | exposure | TRUE | reported | lEqYIj | textfile | 2 | TRUE |
|  | 7 | rs60198979 | G | A | G | A | 0.2146 | 0.000166208 | 0.9135 | 0.914745 | FALSE | FALSE | FALSE | ebi-a-GCST90018120 | 22 | 43646704 | 0.000212229 | 437235 | 0.43 | Idiopathic pulmonary fibrosis \|\| id:ebi-a-GCST90018120 | Idiopathic pulmonary fibrosis | Idiopathic pulmonary fibrosis \|\| \|\| | TRUE | igd | 0.0458 | 2.62E-06 | exposure | TRUE | reported | lEqYIj | textfile | 2 | TRUE |
|  | 8 | rs6900267 | C | A | C | A | 0.2429 | -0.000289682 | 0.0746 | 0.055015 | FALSE | FALSE | FALSE | ebi-a-GCST90018120 | 6 | 380341 | 0.000271943 | 437235 | 0.29 | Idiopathic pulmonary fibrosis \|\| id:ebi-a-GCST90018120 | Idiopathic pulmonary fibrosis | Idiopathic pulmonary fibrosis \|\| \|\| | TRUE | igd | 0.0519 | 2.89E-06 | exposure | TRUE | reported | lEqYIj | textfile | 2 | TRUE |
|  | 9 | rs7232268 | G | A | G | A | -0.2821 | -0.000302712 | 0.9523 | 0.96476 | FALSE | FALSE | FALSE | ebi-a-GCST90018120 | 18 | 67768914 | 0.000313759 | 437235 | 0.33 | Idiopathic pulmonary fibrosis \|\| id:ebi-a-GCST90018120 | Idiopathic pulmonary fibrosis | Idiopathic pulmonary fibrosis \|\| \|\| | TRUE | igd | 0.0599 | 2.55E-06 | exposure | TRUE | reported | lEqYIj | textfile | 2 | TRUE |
|  |  |  |  |  |  |  |  |  |  |  |  |  |  |  |  |  |  |  |  |  |  |  |  |  |  |  |  |  |  |  |  |  |  |
| MIP1b |  | SNP | effect_allele.exposure | other_allele.exposure | effect_allele.outcome | other_allele.outcome | beta.exposure | beta.outcome | eaf.exposure | eaf.outcome | remove | palindromic | ambiguous | id.outcome | chr | pos | se.outcome | samplesize.outcome | pval.outcome | outcome | originalname.outcome | outcome.deprecated | mr_keep.outcome | data_source.outcome | se.exposure | pval.exposure | exposure | mr_keep.exposure | pval_origin.exposure | id.exposure | data_source.exposure | action | mr_keep |
|  | 1 | rs113010081 | C | T | C | T | 0.5954 | 9.86E-05 | 0.1083 | 0.117309 | FALSE | FALSE | FALSE | ebi-a-GCST90018120 | 3 | 46457412 | 0.00018271 | 437235 | 0.59 | Idiopathic pulmonary fibrosis \|\| id:ebi-a-GCST90018120 | Idiopathic pulmonary fibrosis | Idiopathic pulmonary fibrosis \|\| \|\| | TRUE | igd | 0.0236 | 3.85E-140 | exposure | TRUE | reported | nd6n1L | textfile | 2 | TRUE |
|  | 2 | rs113877493 | C | T | C | T | 0.6124 | 1.05E-05 | 0.8777 | 0.920005 | FALSE | FALSE | FALSE | ebi-a-GCST90018120 | 17 | 34812273 | 0.000214228 | 437235 | 0.96 | Idiopathic pulmonary fibrosis \|\| id:ebi-a-GCST90018120 | Idiopathic pulmonary fibrosis | Idiopathic pulmonary fibrosis \|\| \|\| | TRUE | igd | 0.0218 | 1.62E-173 | exposure | TRUE | reported | nd6n1L | textfile | 2 | TRUE |
|  | 3 | rs116237296 | G | A | G | A | -0.5437 | -0.000256235 | 0.9841 | 0.98854 | FALSE | FALSE | FALSE | ebi-a-GCST90018120 | 1 | 87045516 | 0.000557175 | 437235 | 0.649999 | Idiopathic pulmonary fibrosis \|\| id:ebi-a-GCST90018120 | Idiopathic pulmonary fibrosis | Idiopathic pulmonary fibrosis \|\| \|\| | TRUE | igd | 0.1115 | 7.23E-07 | exposure | TRUE | reported | nd6n1L | textfile | 2 | TRUE |
|  | 4 | rs117453826 | G | A | G | A | 0.5774 | -0.000340164 | 0.0159 | 0.01714 | FALSE | FALSE | FALSE | ebi-a-GCST90018120 | 17 | 35132809 | 0.000455526 | 437235 | 0.450001 | Idiopathic pulmonary fibrosis \|\| id:ebi-a-GCST90018120 | Idiopathic pulmonary fibrosis | Idiopathic pulmonary fibrosis \|\| \|\| | TRUE | igd | 0.0593 | 5.07E-22 | exposure | TRUE | reported | nd6n1L | textfile | 2 | TRUE |
|  | 5 | rs12490293 | C | T | C | T | -0.0866 | -0.000101512 | 0.3917 | 0.428259 | FALSE | FALSE | FALSE | ebi-a-GCST90018120 | 3 | 45788127 | 0.000117108 | 437235 | 0.39 | Idiopathic pulmonary fibrosis \|\| id:ebi-a-GCST90018120 | Idiopathic pulmonary fibrosis | Idiopathic pulmonary fibrosis \|\| \|\| | TRUE | igd | 0.0168 | 2.12E-07 | exposure | TRUE | reported | nd6n1L | textfile | 2 | TRUE |
|  | 6 | rs141102180 | G | T | G | T | -0.3225 | -0.000515263 | 0.9821 | 0.982008 | FALSE | FALSE | FALSE | ebi-a-GCST90018120 | 17 | 34436204 | 0.000449328 | 437235 | 0.25 | Idiopathic pulmonary fibrosis \|\| id:ebi-a-GCST90018120 | Idiopathic pulmonary fibrosis | Idiopathic pulmonary fibrosis \|\| \|\| | TRUE | igd | 0.0393 | 1.08E-16 | exposure | TRUE | reported | nd6n1L | textfile | 2 | TRUE |
|  | 7 | rs1437220 | C | T | C | T | -0.1478 | -7.32E-05 | 0.0497 | 0.055358 | FALSE | FALSE | FALSE | ebi-a-GCST90018120 | 17 | 32791974 | 0.000260004 | 437235 | 0.780001 | Idiopathic pulmonary fibrosis \|\| id:ebi-a-GCST90018120 | Idiopathic pulmonary fibrosis | Idiopathic pulmonary fibrosis \|\| \|\| | TRUE | igd | 0.0315 | 3.53E-06 | exposure | TRUE | reported | nd6n1L | textfile | 2 | TRUE |
|  | 8 | rs17138331 | G | A | G | A | 0.1391 | -8.61E-05 | 0.0944 | 0.119926 | FALSE | FALSE | FALSE | ebi-a-GCST90018120 | 7 | 7866368 | 0.000179225 | 437235 | 0.630001 | Idiopathic pulmonary fibrosis \|\| id:ebi-a-GCST90018120 | Idiopathic pulmonary fibrosis | Idiopathic pulmonary fibrosis \|\| \|\| | TRUE | igd | 0.0295 | 2.26E-06 | exposure | TRUE | reported | nd6n1L | textfile | 2 | TRUE |
|  | 9 | rs2411161 | C | T | C | T | -0.1714 | 0.000237832 | 0.0507 | 0.055637 | FALSE | FALSE | FALSE | ebi-a-GCST90018120 | 17 | 35501799 | 0.000254071 | 437235 | 0.35 | Idiopathic pulmonary fibrosis \|\| id:ebi-a-GCST90018120 | Idiopathic pulmonary fibrosis | Idiopathic pulmonary fibrosis \|\| \|\| | TRUE | igd | 0.0367 | 3.14E-06 | exposure | TRUE | reported | nd6n1L | textfile | 2 | TRUE |
|  | 10 | rs281749 | C | T | C | T | -0.0799 | -0.000163788 | 0.7083 | 0.683123 | FALSE | FALSE | FALSE | ebi-a-GCST90018120 | 8 | 108638645 | 0.000124519 | 437235 | 0.19 | Idiopathic pulmonary fibrosis \|\| id:ebi-a-GCST90018120 | Idiopathic pulmonary fibrosis | Idiopathic pulmonary fibrosis \|\| \|\| | TRUE | igd | 0.0171 | 3.17E-06 | exposure | TRUE | reported | nd6n1L | textfile | 2 | TRUE |
|  | 11 | rs3760440 | G | A | G | A | -0.1236 | -0.000126976 | 0.331 | 0.341973 | FALSE | FALSE | FALSE | ebi-a-GCST90018120 | 17 | 35228301 | 0.000122217 | 437235 | 0.3 | Idiopathic pulmonary fibrosis \|\| id:ebi-a-GCST90018120 | Idiopathic pulmonary fibrosis | Idiopathic pulmonary fibrosis \|\| \|\| | TRUE | igd | 0.0162 | 2.75E-14 | exposure | TRUE | reported | nd6n1L | textfile | 2 | TRUE |
|  | 12 | rs62242409 | C | T | C | T | -0.1282 | 0.000289289 | 0.7425 | 0.755025 | FALSE | FALSE | FALSE | ebi-a-GCST90018120 | 3 | 45349210 | 0.000134613 | 437235 | 0.0309999 | Idiopathic pulmonary fibrosis \|\| id:ebi-a-GCST90018120 | Idiopathic pulmonary fibrosis | Idiopathic pulmonary fibrosis \|\| \|\| | TRUE | igd | 0.0193 | 3.43E-11 | exposure | TRUE | reported | nd6n1L | textfile | 2 | TRUE |
|  | 13 | rs72791296 | C | T | C | T | -0.2369 | 0.00022202 | 0.9414 | 0.953968 | FALSE | FALSE | FALSE | ebi-a-GCST90018120 | 5 | 120950050 | 0.00028186 | 437235 | 0.43 | Idiopathic pulmonary fibrosis \|\| id:ebi-a-GCST90018120 | Idiopathic pulmonary fibrosis | Idiopathic pulmonary fibrosis \|\| \|\| | TRUE | igd | 0.0466 | 3.78E-07 | exposure | TRUE | reported | nd6n1L | textfile | 2 | TRUE |
|  | 14 | rs72799710 | C | T | C | T | 0.1014 | -0.000177607 | 0.834 | 0.814972 | FALSE | FALSE | FALSE | ebi-a-GCST90018120 | 5 | 123161665 | 0.000149559 | 437235 | 0.23 | Idiopathic pulmonary fibrosis \|\| id:ebi-a-GCST90018120 | Idiopathic pulmonary fibrosis | Idiopathic pulmonary fibrosis \|\| \|\| | TRUE | igd | 0.0218 | 3.21E-06 | exposure | TRUE | reported | nd6n1L | textfile | 2 | TRUE |
|  | 15 | rs74810984 | C | T | C | T | -0.2206 | 0.00015075 | 0.0219 | 0.02042 | FALSE | FALSE | FALSE | ebi-a-GCST90018120 | 10 | 129674466 | 0.000428282 | 437235 | 0.73 | Idiopathic pulmonary fibrosis \|\| id:ebi-a-GCST90018120 | Idiopathic pulmonary fibrosis | Idiopathic pulmonary fibrosis \|\| \|\| | TRUE | igd | 0.0474 | 1.96E-06 | exposure | TRUE | reported | nd6n1L | textfile | 2 | TRUE |
|  | 16 | rs76582507 | G | A | G | A | -0.3175 | 0.000430793 | 0.9732 | 0.982144 | FALSE | FALSE | FALSE | ebi-a-GCST90018120 | 9 | 37510072 | 0.000453819 | 437235 | 0.34 | Idiopathic pulmonary fibrosis \|\| id:ebi-a-GCST90018120 | Idiopathic pulmonary fibrosis | Idiopathic pulmonary fibrosis \|\| \|\| | TRUE | igd | 0.0677 | 3.26E-06 | exposure | TRUE | reported | nd6n1L | textfile | 2 | TRUE |
|  | 17 | rs76583883 | G | T | G | T | 0.2317 | 0.000643417 | 0.9682 | 0.958524 | FALSE | FALSE | FALSE | ebi-a-GCST90018120 | 21 | 47356359 | 0.000291346 | 437235 | 0.0269998 | Idiopathic pulmonary fibrosis \|\| id:ebi-a-GCST90018120 | Idiopathic pulmonary fibrosis | Idiopathic pulmonary fibrosis \|\| \|\| | TRUE | igd | 0.0511 | 4.99E-06 | exposure | TRUE | reported | nd6n1L | textfile | 2 | TRUE |
|  | 18 | rs76776296 | G | A | G | A | -0.2997 | 0.000453505 | 0.0368 | 0.038675 | FALSE | FALSE | FALSE | ebi-a-GCST90018120 | 7 | 115128487 | 0.000299778 | 437235 | 0.13 | Idiopathic pulmonary fibrosis \|\| id:ebi-a-GCST90018120 | Idiopathic pulmonary fibrosis | Idiopathic pulmonary fibrosis \|\| \|\| | TRUE | igd | 0.0598 | 5.55E-07 | exposure | TRUE | reported | nd6n1L | textfile | 2 | TRUE |
|  | 19 | rs9793308 | G | A | G | A | -0.0835 | 0.000208844 | 0.3718 | 0.393019 | FALSE | FALSE | FALSE | ebi-a-GCST90018120 | 1 | 6604585 | 0.00013633 | 437235 | 0.13 | Idiopathic pulmonary fibrosis \|\| id:ebi-a-GCST90018120 | Idiopathic pulmonary fibrosis | Idiopathic pulmonary fibrosis \|\| \|\| | TRUE | igd | 0.0178 | 2.52E-06 | exposure | TRUE | reported | nd6n1L | textfile | 2 | TRUE |
|  | 20 | rs9850846 | G | A | G | A | -0.072 | -9.06E-05 | 0.4443 | 0.465846 | FALSE | FALSE | FALSE | ebi-a-GCST90018120 | 3 | 45114682 | 0.000116405 | 437235 | 0.44 | Idiopathic pulmonary fibrosis \|\| id:ebi-a-GCST90018120 | Idiopathic pulmonary fibrosis | Idiopathic pulmonary fibrosis \|\| \|\| | TRUE | igd | 0.0157 | 4.48E-06 | exposure | TRUE | reported | nd6n1L | textfile | 2 | TRUE |
|  |  |  |  |  |  |  |  |  |  |  |  |  |  |  |  |  |  |  |  |  |  |  |  |  |  |  |  |  |  |  |  |  |  |
| RANTES |  | SNP | effect_allele.exposure | other_allele.exposure | effect_allele.outcome | other_allele.outcome | beta.exposure | beta.outcome | eaf.exposure | eaf.outcome | remove | palindromic | ambiguous | id.outcome | chr | pos | se.outcome | samplesize.outcome | pval.outcome | outcome | originalname.outcome | outcome.deprecated | mr_keep.outcome | data_source.outcome | se.exposure | pval.exposure | exposure | mr_keep.exposure | pval_origin.exposure | id.exposure | data_source.exposure | action | mr_keep |
|  | 1 | rs112072646 | G | A | G | A | -0.4286 | 0.000129638 | 0.9692 | 0.971862 | FALSE | FALSE | FALSE | ebi-a-GCST90018120 | 2 | 53444393 | 0.000351304 | 437235 | 0.709999 | Idiopathic pulmonary fibrosis \|\| id:ebi-a-GCST90018120 | Idiopathic pulmonary fibrosis | Idiopathic pulmonary fibrosis \|\| \|\| | TRUE | igd | 0.0862 | 6.48E-07 | exposure | TRUE | reported | T50WQ0 | textfile | 2 | TRUE |
|  | 2 | rs147509526 | C | T | C | T | 0.358 | 0.000248844 | 0.9871 | 0.984312 | FALSE | FALSE | FALSE | ebi-a-GCST90018120 | 19 | 15776330 | 0.000483432 | 437235 | 0.61 | Idiopathic pulmonary fibrosis \|\| id:ebi-a-GCST90018120 | Idiopathic pulmonary fibrosis | Idiopathic pulmonary fibrosis \|\| \|\| | TRUE | igd | 0.0717 | 6.93E-07 | exposure | TRUE | reported | T50WQ0 | textfile | 2 | TRUE |
|  | 3 | rs2251660 | C | A | C | A | -0.1829 | -0.00034988 | 0.1451 | 0.148694 | FALSE | FALSE | FALSE | ebi-a-GCST90018120 | 17 | 34252537 | 0.00016279 | 437235 | 0.032 | Idiopathic pulmonary fibrosis \|\| id:ebi-a-GCST90018120 | Idiopathic pulmonary fibrosis | Idiopathic pulmonary fibrosis \|\| \|\| | TRUE | igd | 0.0359 | 3.83E-07 | exposure | TRUE | reported | T50WQ0 | textfile | 2 | TRUE |
|  | 4 | rs4940620 | G | A | G | A | 0.2494 | -0.000172382 | 0.0636 | 0.061281 | FALSE | FALSE | FALSE | ebi-a-GCST90018120 | 18 | 61971111 | 0.000243862 | 437235 | 0.48 | Idiopathic pulmonary fibrosis \|\| id:ebi-a-GCST90018120 | Idiopathic pulmonary fibrosis | Idiopathic pulmonary fibrosis \|\| \|\| | TRUE | igd | 0.054 | 3.54E-06 | exposure | TRUE | reported | T50WQ0 | textfile | 2 | TRUE |
|  | 5 | rs62438851 | G | A | G | A | 0.1957 | -5.87E-05 | 0.1352 | 0.14099 | FALSE | FALSE | FALSE | ebi-a-GCST90018120 | 6 | 145230309 | 0.000169435 | 437235 | 0.73 | Idiopathic pulmonary fibrosis \|\| id:ebi-a-GCST90018120 | Idiopathic pulmonary fibrosis | Idiopathic pulmonary fibrosis \|\| \|\| | TRUE | igd | 0.0414 | 2.33E-06 | exposure | TRUE | reported | T50WQ0 | textfile | 2 | TRUE |
|  | 6 | rs7000423 | C | T | C | T | 0.1318 | 0.000286411 | 0.674 | 0.321008 | FALSE | FALSE | FALSE | ebi-a-GCST90018120 | 8 | 111053649 | 0.000123879 | 437235 | 0.021 | Idiopathic pulmonary fibrosis \|\| id:ebi-a-GCST90018120 | Idiopathic pulmonary fibrosis | Idiopathic pulmonary fibrosis \|\| \|\| | TRUE | igd | 0.0253 | 1.82E-07 | exposure | TRUE | reported | T50WQ0 | textfile | 2 | TRUE |
|  | 7 | rs72793342 | G | A | G | A | 0.1487 | 0.000166128 | 0.7922 | 0.800069 | FALSE | FALSE | FALSE | ebi-a-GCST90018120 | 16 | 30548352 | 0.000144898 | 437235 | 0.25 | Idiopathic pulmonary fibrosis \|\| id:ebi-a-GCST90018120 | Idiopathic pulmonary fibrosis | Idiopathic pulmonary fibrosis \|\| \|\| | TRUE | igd | 0.0308 | 1.48E-06 | exposure | TRUE | reported | T50WQ0 | textfile | 2 | TRUE |
|  | 8 | rs74472919 | C | T | C | T | -0.3313 | -0.000564997 | 0.9811 | 0.96638 | FALSE | FALSE | FALSE | ebi-a-GCST90018120 | 13 | 82200650 | 0.00033162 | 437235 | 0.0879995 | Idiopathic pulmonary fibrosis \|\| id:ebi-a-GCST90018120 | Idiopathic pulmonary fibrosis | Idiopathic pulmonary fibrosis \|\| \|\| | TRUE | igd | 0.0605 | 3.97E-08 | exposure | TRUE | reported | T50WQ0 | textfile | 2 | TRUE |
|  | 9 | rs75613039 | C | T | C | T | -0.37 | 0.000473231 | 0.9692 | 0.968999 | FALSE | FALSE | FALSE | ebi-a-GCST90018120 | 11 | 129576583 | 0.000337784 | 437235 | 0.16 | Idiopathic pulmonary fibrosis \|\| id:ebi-a-GCST90018120 | Idiopathic pulmonary fibrosis | Idiopathic pulmonary fibrosis \|\| \|\| | TRUE | igd | 0.081 | 4.81E-06 | exposure | TRUE | reported | T50WQ0 | textfile | 2 | TRUE |
|  | 10 | rs818452 | C | T | C | T | -0.2381 | 0.000284629 | 0.9484 | 0.926086 | FALSE | FALSE | FALSE | ebi-a-GCST90018120 | 6 | 152915796 | 0.000222112 | 437235 | 0.2 | Idiopathic pulmonary fibrosis \|\| id:ebi-a-GCST90018120 | Idiopathic pulmonary fibrosis | Idiopathic pulmonary fibrosis \|\| \|\| | TRUE | igd | 0.0505 | 2.36E-06 | exposure | TRUE | reported | T50WQ0 | textfile | 2 | TRUE |
|  |  |  |  |  |  |  |  |  |  |  |  |  |  |  |  |  |  |  |  |  |  |  |  |  |  |  |  |  |  |  |  |  |  |
| TNFa |  | SNP | effect_allele.exposure | other_allele.exposure | effect_allele.outcome | other_allele.outcome | beta.exposure | beta.outcome | eaf.exposure | eaf.outcome | remove | palindromic | ambiguous | id.outcome | chr | pos | se.outcome | samplesize.outcome | pval.outcome | outcome | originalname.outcome | outcome.deprecated | mr_keep.outcome | data_source.outcome | se.exposure | pval.exposure | exposure | mr_keep.exposure | pval_origin.exposure | id.exposure | data_source.exposure | action | mr_keep |
|  | 1 | rs10834997 | G | A | G | A | 0.1247 | -0.000200622 | 0.3598 | 0.307773 | FALSE | FALSE | FALSE | ebi-a-GCST90018120 | 11 | 26526948 | 0.00012536 | 437235 | 0.11 | Idiopathic pulmonary fibrosis \|\| id:ebi-a-GCST90018120 | Idiopathic pulmonary fibrosis | Idiopathic pulmonary fibrosis \|\| \|\| | TRUE | igd | 0.0258 | 1.33E-06 | exposure | TRUE | reported | j0q75B | textfile | 2 | TRUE |
|  | 2 | rs111332265 | G | A | G | A | 0.3766 | -0.000229674 | 0.0716 | 0.057354 | FALSE | FALSE | FALSE | ebi-a-GCST90018120 | 5 | 150393107 | 0.000253114 | 437235 | 0.36 | Idiopathic pulmonary fibrosis \|\| id:ebi-a-GCST90018120 | Idiopathic pulmonary fibrosis | Idiopathic pulmonary fibrosis \|\| \|\| | TRUE | igd | 0.0754 | 6.63E-07 | exposure | TRUE | reported | j0q75B | textfile | 2 | TRUE |
|  | 3 | rs8121916 | C | A | C | A | -0.1306 | 0.000196061 | 0.7744 | 0.753301 | FALSE | FALSE | FALSE | ebi-a-GCST90018120 | 20 | 12401325 | 0.000134013 | 437235 | 0.14 | Idiopathic pulmonary fibrosis \|\| id:ebi-a-GCST90018120 | Idiopathic pulmonary fibrosis | Idiopathic pulmonary fibrosis \|\| \|\| | TRUE | igd | 0.0278 | 2.72E-06 | exposure | TRUE | reported | j0q75B | textfile | 2 | TRUE |
|  |  |  |  |  |  |  |  |  |  |  |  |  |  |  |  |  |  |  |  |  |  |  |  |  |  |  |  |  |  |  |  |  |  |
| TNFb |  | SNP | effect_allele.exposure | other_allele.exposure | effect_allele.outcome | other_allele.outcome | beta.exposure | beta.outcome | eaf.exposure | eaf.outcome | remove | palindromic | ambiguous | id.outcome | chr | pos | se.outcome | samplesize.outcome | pval.outcome | outcome | originalname.outcome | outcome.deprecated | mr_keep.outcome | data_source.outcome | se.exposure | pval.exposure | exposure | mr_keep.exposure | pval_origin.exposure | id.exposure | data_source.exposure | action | mr_keep |
|  | 1 | rs10925040 | C | T | C | T | -0.1755 | 0.000145996 | 0.6113 | 0.622741 | FALSE | FALSE | FALSE | ebi-a-GCST90018120 | 1 | 247622698 | 0.000120082 | 437235 | 0.22 | Idiopathic pulmonary fibrosis \|\| id:ebi-a-GCST90018120 | Idiopathic pulmonary fibrosis | Idiopathic pulmonary fibrosis \|\| \|\| | TRUE | igd | 0.0373 | 2.67E-06 | exposure | TRUE | reported | Bkx7Et | textfile | 2 | TRUE |
|  | 2 | rs753274 | C | T | C | T | 0.1736 | 4.05E-05 | 0.4642 | 0.452928 | FALSE | FALSE | FALSE | ebi-a-GCST90018120 | 19 | 14436462 | 0.000116427 | 437235 | 0.73 | Idiopathic pulmonary fibrosis \|\| id:ebi-a-GCST90018120 | Idiopathic pulmonary fibrosis | Idiopathic pulmonary fibrosis \|\| \|\| | TRUE | igd | 0.0371 | 2.77E-06 | exposure | TRUE | reported | Bkx7Et | textfile | 2 | TRUE |
|  | 3 | rs7629875 | G | A | G | A | -0.3766 | 0.000174331 | 0.0577 | 0.050598 | FALSE | FALSE | FALSE | ebi-a-GCST90018120 | 3 | 174385622 | 0.000264465 | 437235 | 0.51 | Idiopathic pulmonary fibrosis \|\| id:ebi-a-GCST90018120 | Idiopathic pulmonary fibrosis | Idiopathic pulmonary fibrosis \|\| \|\| | TRUE | igd | 0.0774 | 1.37E-06 | exposure | TRUE | reported | Bkx7Et | textfile | 2 | TRUE |
|  | 4 | rs78296352 | G | T | G | T | -1.2215 | -0.000455665 | 0.9712 | 0.961226 | FALSE | FALSE | FALSE | ebi-a-GCST90018120 | 1 | 22821844 | 0.000298985 | 437235 | 0.13 | Idiopathic pulmonary fibrosis \|\| id:ebi-a-GCST90018120 | Idiopathic pulmonary fibrosis | Idiopathic pulmonary fibrosis \|\| \|\| | TRUE | igd | 0.1366 | 4.76E-21 | exposure | TRUE | reported | Bkx7Et | textfile | 2 | TRUE |
|  |  |  |  |  |  |  |  |  |  |  |  |  |  |  |  |  |  |  |  |  |  |  |  |  |  |  |  |  |  |  |  |  |  |
| TRAIL |  | SNP | effect_allele.exposure | other_allele.exposure | effect_allele.outcome | other_allele.outcome | beta.exposure | beta.outcome | eaf.exposure | eaf.outcome | remove | palindromic | ambiguous | id.outcome | chr | pos | se.outcome | samplesize.outcome | pval.outcome | outcome | originalname.outcome | outcome.deprecated | mr_keep.outcome | data_source.outcome | se.exposure | pval.exposure | exposure | mr_keep.exposure | pval_origin.exposure | id.exposure | data_source.exposure | action | mr_keep |
|  | 1 | rs113010081 | C | T | C | T | 0.5954 | 9.86E-05 | 0.1083 | 0.117309 | FALSE | FALSE | FALSE | ebi-a-GCST90018120 | 3 | 46457412 | 0.00018271 | 437235 | 0.59 | Idiopathic pulmonary fibrosis \|\| id:ebi-a-GCST90018120 | Idiopathic pulmonary fibrosis | Idiopathic pulmonary fibrosis \|\| \|\| | TRUE | igd | 0.0236 | 3.85E-140 | exposure | TRUE | reported | OzMjFw | textfile | 2 | TRUE |
|  | 2 | rs113877493 | C | T | C | T | 0.6124 | 1.05E-05 | 0.8777 | 0.920005 | FALSE | FALSE | FALSE | ebi-a-GCST90018120 | 17 | 34812273 | 0.000214228 | 437235 | 0.96 | Idiopathic pulmonary fibrosis \|\| id:ebi-a-GCST90018120 | Idiopathic pulmonary fibrosis | Idiopathic pulmonary fibrosis \|\| \|\| | TRUE | igd | 0.0218 | 1.62E-173 | exposure | TRUE | reported | OzMjFw | textfile | 2 | TRUE |
|  | 3 | rs116237296 | G | A | G | A | -0.5437 | -0.000256235 | 0.9841 | 0.98854 | FALSE | FALSE | FALSE | ebi-a-GCST90018120 | 1 | 87045516 | 0.000557175 | 437235 | 0.649999 | Idiopathic pulmonary fibrosis \|\| id:ebi-a-GCST90018120 | Idiopathic pulmonary fibrosis | Idiopathic pulmonary fibrosis \|\| \|\| | TRUE | igd | 0.1115 | 7.23E-07 | exposure | TRUE | reported | OzMjFw | textfile | 2 | TRUE |
|  | 4 | rs117453826 | G | A | G | A | 0.5774 | -0.000340164 | 0.0159 | 0.01714 | FALSE | FALSE | FALSE | ebi-a-GCST90018120 | 17 | 35132809 | 0.000455526 | 437235 | 0.450001 | Idiopathic pulmonary fibrosis \|\| id:ebi-a-GCST90018120 | Idiopathic pulmonary fibrosis | Idiopathic pulmonary fibrosis \|\| \|\| | TRUE | igd | 0.0593 | 5.07E-22 | exposure | TRUE | reported | OzMjFw | textfile | 2 | TRUE |
|  | 5 | rs12490293 | C | T | C | T | -0.0866 | -0.000101512 | 0.3917 | 0.428259 | FALSE | FALSE | FALSE | ebi-a-GCST90018120 | 3 | 45788127 | 0.000117108 | 437235 | 0.39 | Idiopathic pulmonary fibrosis \|\| id:ebi-a-GCST90018120 | Idiopathic pulmonary fibrosis | Idiopathic pulmonary fibrosis \|\| \|\| | TRUE | igd | 0.0168 | 2.12E-07 | exposure | TRUE | reported | OzMjFw | textfile | 2 | TRUE |
|  | 6 | rs141102180 | G | T | G | T | -0.3225 | -0.000515263 | 0.9821 | 0.982008 | FALSE | FALSE | FALSE | ebi-a-GCST90018120 | 17 | 34436204 | 0.000449328 | 437235 | 0.25 | Idiopathic pulmonary fibrosis \|\| id:ebi-a-GCST90018120 | Idiopathic pulmonary fibrosis | Idiopathic pulmonary fibrosis \|\| \|\| | TRUE | igd | 0.0393 | 1.08E-16 | exposure | TRUE | reported | OzMjFw | textfile | 2 | TRUE |
|  | 7 | rs1437220 | C | T | C | T | -0.1478 | -7.32E-05 | 0.0497 | 0.055358 | FALSE | FALSE | FALSE | ebi-a-GCST90018120 | 17 | 32791974 | 0.000260004 | 437235 | 0.780001 | Idiopathic pulmonary fibrosis \|\| id:ebi-a-GCST90018120 | Idiopathic pulmonary fibrosis | Idiopathic pulmonary fibrosis \|\| \|\| | TRUE | igd | 0.0315 | 3.53E-06 | exposure | TRUE | reported | OzMjFw | textfile | 2 | TRUE |
|  | 8 | rs17138331 | G | A | G | A | 0.1391 | -8.61E-05 | 0.0944 | 0.119926 | FALSE | FALSE | FALSE | ebi-a-GCST90018120 | 7 | 7866368 | 0.000179225 | 437235 | 0.630001 | Idiopathic pulmonary fibrosis \|\| id:ebi-a-GCST90018120 | Idiopathic pulmonary fibrosis | Idiopathic pulmonary fibrosis \|\| \|\| | TRUE | igd | 0.0295 | 2.26E-06 | exposure | TRUE | reported | OzMjFw | textfile | 2 | TRUE |
|  | 9 | rs2411161 | C | T | C | T | -0.1714 | 0.000237832 | 0.0507 | 0.055637 | FALSE | FALSE | FALSE | ebi-a-GCST90018120 | 17 | 35501799 | 0.000254071 | 437235 | 0.35 | Idiopathic pulmonary fibrosis \|\| id:ebi-a-GCST90018120 | Idiopathic pulmonary fibrosis | Idiopathic pulmonary fibrosis \|\| \|\| | TRUE | igd | 0.0367 | 3.14E-06 | exposure | TRUE | reported | OzMjFw | textfile | 2 | TRUE |
|  | 10 | rs281749 | C | T | C | T | -0.0799 | -0.000163788 | 0.7083 | 0.683123 | FALSE | FALSE | FALSE | ebi-a-GCST90018120 | 8 | 108638645 | 0.000124519 | 437235 | 0.19 | Idiopathic pulmonary fibrosis \|\| id:ebi-a-GCST90018120 | Idiopathic pulmonary fibrosis | Idiopathic pulmonary fibrosis \|\| \|\| | TRUE | igd | 0.0171 | 3.17E-06 | exposure | TRUE | reported | OzMjFw | textfile | 2 | TRUE |
|  | 11 | rs3760440 | G | A | G | A | -0.1236 | -0.000126976 | 0.331 | 0.341973 | FALSE | FALSE | FALSE | ebi-a-GCST90018120 | 17 | 35228301 | 0.000122217 | 437235 | 0.3 | Idiopathic pulmonary fibrosis \|\| id:ebi-a-GCST90018120 | Idiopathic pulmonary fibrosis | Idiopathic pulmonary fibrosis \|\| \|\| | TRUE | igd | 0.0162 | 2.75E-14 | exposure | TRUE | reported | OzMjFw | textfile | 2 | TRUE |
|  | 12 | rs62242409 | C | T | C | T | -0.1282 | 0.000289289 | 0.7425 | 0.755025 | FALSE | FALSE | FALSE | ebi-a-GCST90018120 | 3 | 45349210 | 0.000134613 | 437235 | 0.0309999 | Idiopathic pulmonary fibrosis \|\| id:ebi-a-GCST90018120 | Idiopathic pulmonary fibrosis | Idiopathic pulmonary fibrosis \|\| \|\| | TRUE | igd | 0.0193 | 3.43E-11 | exposure | TRUE | reported | OzMjFw | textfile | 2 | TRUE |
|  | 13 | rs72791296 | C | T | C | T | -0.2369 | 0.00022202 | 0.9414 | 0.953968 | FALSE | FALSE | FALSE | ebi-a-GCST90018120 | 5 | 120950050 | 0.00028186 | 437235 | 0.43 | Idiopathic pulmonary fibrosis \|\| id:ebi-a-GCST90018120 | Idiopathic pulmonary fibrosis | Idiopathic pulmonary fibrosis \|\| \|\| | TRUE | igd | 0.0466 | 3.78E-07 | exposure | TRUE | reported | OzMjFw | textfile | 2 | TRUE |
|  | 14 | rs72799710 | C | T | C | T | 0.1014 | -0.000177607 | 0.834 | 0.814972 | FALSE | FALSE | FALSE | ebi-a-GCST90018120 | 5 | 123161665 | 0.000149559 | 437235 | 0.23 | Idiopathic pulmonary fibrosis \|\| id:ebi-a-GCST90018120 | Idiopathic pulmonary fibrosis | Idiopathic pulmonary fibrosis \|\| \|\| | TRUE | igd | 0.0218 | 3.21E-06 | exposure | TRUE | reported | OzMjFw | textfile | 2 | TRUE |
|  | 15 | rs74810984 | C | T | C | T | -0.2206 | 0.00015075 | 0.0219 | 0.02042 | FALSE | FALSE | FALSE | ebi-a-GCST90018120 | 10 | 129674466 | 0.000428282 | 437235 | 0.73 | Idiopathic pulmonary fibrosis \|\| id:ebi-a-GCST90018120 | Idiopathic pulmonary fibrosis | Idiopathic pulmonary fibrosis \|\| \|\| | TRUE | igd | 0.0474 | 1.96E-06 | exposure | TRUE | reported | OzMjFw | textfile | 2 | TRUE |
|  | 16 | rs76582507 | G | A | G | A | -0.3175 | 0.000430793 | 0.9732 | 0.982144 | FALSE | FALSE | FALSE | ebi-a-GCST90018120 | 9 | 37510072 | 0.000453819 | 437235 | 0.34 | Idiopathic pulmonary fibrosis \|\| id:ebi-a-GCST90018120 | Idiopathic pulmonary fibrosis | Idiopathic pulmonary fibrosis \|\| \|\| | TRUE | igd | 0.0677 | 3.26E-06 | exposure | TRUE | reported | OzMjFw | textfile | 2 | TRUE |
|  | 17 | rs76583883 | G | T | G | T | 0.2317 | 0.000643417 | 0.9682 | 0.958524 | FALSE | FALSE | FALSE | ebi-a-GCST90018120 | 21 | 47356359 | 0.000291346 | 437235 | 0.0269998 | Idiopathic pulmonary fibrosis \|\| id:ebi-a-GCST90018120 | Idiopathic pulmonary fibrosis | Idiopathic pulmonary fibrosis \|\| \|\| | TRUE | igd | 0.0511 | 4.99E-06 | exposure | TRUE | reported | OzMjFw | textfile | 2 | TRUE |
|  | 18 | rs76776296 | G | A | G | A | -0.2997 | 0.000453505 | 0.0368 | 0.038675 | FALSE | FALSE | FALSE | ebi-a-GCST90018120 | 7 | 115128487 | 0.000299778 | 437235 | 0.13 | Idiopathic pulmonary fibrosis \|\| id:ebi-a-GCST90018120 | Idiopathic pulmonary fibrosis | Idiopathic pulmonary fibrosis \|\| \|\| | TRUE | igd | 0.0598 | 5.55E-07 | exposure | TRUE | reported | OzMjFw | textfile | 2 | TRUE |
|  | 19 | rs9793308 | G | A | G | A | -0.0835 | 0.000208844 | 0.3718 | 0.393019 | FALSE | FALSE | FALSE | ebi-a-GCST90018120 | 1 | 6604585 | 0.00013633 | 437235 | 0.13 | Idiopathic pulmonary fibrosis \|\| id:ebi-a-GCST90018120 | Idiopathic pulmonary fibrosis | Idiopathic pulmonary fibrosis \|\| \|\| | TRUE | igd | 0.0178 | 2.52E-06 | exposure | TRUE | reported | OzMjFw | textfile | 2 | TRUE |
|  | 20 | rs9850846 | G | A | G | A | -0.072 | -9.06E-05 | 0.4443 | 0.465846 | FALSE | FALSE | FALSE | ebi-a-GCST90018120 | 3 | 45114682 | 0.000116405 | 437235 | 0.44 | Idiopathic pulmonary fibrosis \|\| id:ebi-a-GCST90018120 | Idiopathic pulmonary fibrosis | Idiopathic pulmonary fibrosis \|\| \|\| | TRUE | igd | 0.0157 | 4.48E-06 | exposure | TRUE | reported | OzMjFw | textfile | 2 | TRUE |
