# Supplementary material for: Circulating inflammatory cytokines and risk of idiopathic pulmonary fibrosis: a Mendelian randomization study
Source: BMC Pulm Med. 2023 Oct 3;23:369. doi: 10.1186/s12890-023-02658-3 (PMC10548733; doi:10.1186/s12890-023-02658-3)
Supplement: Supplementary file 3 — Supplementary Material 3 [file 12890_2023_2658_MOESM3_ESM.docx]

**Supplementary Table 2. Effect estimates for association of genetically predicted inflammatory cytokines with idiopathic pulmonary fibrosis using 7 Mendelian randomization methods.**

| **Diseases** | **inflammatory cytokines** | **Methods** | **No. of SNPs** | **OR** | **95%CI** | **P-value** | **Cochran’s Q** | **MR-Egger Intercept** |
| --- | --- | --- | --- | --- | --- | --- | --- | --- |
| IPF | *IL2* | MR Egger |  | 1.000 | 0.998-1.001 | 0.397 | 2.970, 0.965 | 0.333 |
|  |  | IVW (random effects) |  | 1.000 | 1.000-1.000 | 0.979 | 4.015, 0.947 |  |
|  |  | IVW (fixed effects) |  | 1.000 | 1.000-1.001 | 0.987 |  |  |
|  |  | MR Egger (bootstrap) |  | 1.000 | 1.000-1.000 | 0.347 |  |  |
|  |  | Simple median |  | 1.000 | 1.000-1.001 | 0.760 |  |  |
|  |  | Weighted median |  | 1.000 | 1.000-1.001 | 0.947 |  |  |
|  |  | Penalised weighted median |  | 1.000 | 1.000-1.001 | 0.947 |  |  |
|  | *IL6* | MR Egger |  | 0.999 | 0.996-1.001 | 0.271 | 8.684, 0.124 | 0.483 |
|  |  | IVW (random effects) |  | 0.999 | 0.998-1.000 | 0.246 | 9.642, 0.141 |  |
|  |  | IVW (fixed effects) |  | 0.999 | 0.998-1.000 | 0.142 |  |  |
|  |  | MR Egger (bootstrap) |  | 1.002 | 0.999-1.005 | 0.125 |  |  |
|  |  | Simple median |  | 0.999 | 0.998-1.001 | 0.329 |  |  |
|  |  | Weighted median |  | 0.999 | 0.998-1.001 | 0.260 |  |  |
|  |  | Penalised weighted median |  | 0.999 | 0.998-1.001 | 0.293 |  |  |
|  | IL-8 | MR Egger |  | 0.999 | 0.998-1.001 | 0.541 | 3.609, 0.165 | 0.556 |
|  |  | IVW (random effects) |  | 1.000 | 0.999-1.001 | 0.758 | 4.495, 0.213 |  |
|  |  | IVW (fixed effects) |  | 1.000 | 0.999-1.001 | 0.706 |  |  |
|  |  | MR Egger (bootstrap) |  | 1.001 | 0.990-1.012 | 0.392 |  |  |
|  |  | Simple median |  | 1.000 | 0.999-1.001 | 0.604 |  |  |
|  |  | Weighted median |  | 1.000 | 0.999-1.001 | 0.417 |  |  |
|  |  | Penalised weighted median |  | 1.000 | 0.999-1.000 | 0.425 |  |  |
|  | IL-10 | MR Egger |  | 1.000 | 0.999-1.001 | 0.545 | 30.654, 0.103 | 0.731 |
|  |  | IVW (random effects) |  | 1.000 | 0.999-1.000 | 0.541 | 30.822, 0.127 |  |
|  |  | IVW (fixed effects) |  | 1.000 | 0.999-1.000 | 0.479 |  |  |
|  |  | MR Egger (bootstrap) |  | 1.000 | 0.999-1.000 | 0.203 |  |  |
|  |  | Simple median |  | 1.000 | 0.999-1.000 | 0.565 |  |  |
|  |  | Weighted median |  | 1.000 | 0.999-1.000 | 0.475 |  |  |
|  |  | Penalised weighted median |  | 1.000 | 0.999-1.000 | 0.456 |  |  |
|  | IL-13 | MR Egger |  | 1.000 | 0.999-1.000 | 0.850 | 9.635, 0.210 | 0.371 |
|  |  | IVW (random effects) |  | 1.000 | 0.999-1.000 | 0.092 | 10.892, 0.208 |  |
|  |  | IVW (fixed effects) |  | 1.000 | 0.999-1.000 | 0.050 |  |  |
|  |  | MR Egger (bootstrap) |  | 1.000 | 0.999-1.000 | 0.423 |  |  |
|  |  | Simple median |  | 0.999 | 0.998-1.000 | 0.113 |  |  |
|  |  | Weighted median |  | 1.000 | 0.999-1.000 | 0.198 |  |  |
|  |  | Penalised weighted median |  | 1.000 | 0.999-1.000 | 0.210 |  |  |
|  | IL-14 | MR Egger |  | 1.000 | 0.999-1.001 | 0.713 | 5.694, 0.770 | 0.444 |
|  |  | IVW (random effects) |  | 1.001 | 1.000-1.001 | 0.026 | 6.337, 0.786 |  |
|  |  | IVW (fixed effects) |  | 1.001 | 1.000-1.001 | 0.076 |  |  |
|  |  | MR Egger (bootstrap) |  | 1.001 | 0.999-1.002 | 0.158 |  |  |
|  |  | Simple median |  | 1.000 | 0.999-1.001 | 0.535 |  |  |
|  |  | Weighted median |  | 1.000 | 0.999-1.001 | 0.767 |  |  |
|  |  | Penalised weighted median |  | 1.000 | 0.999-1.001 | 0.766 |  |  |
|  | IL-16 | MR Egger |  | 1.000 | 1.000-1.001 | 0.218 | 9.601, 0.384 | 0.307 |
|  |  | IVW (random effects) |  | 1.000 | 1.000-1.001 | 0.449 | 10.853, 0.369 |  |
|  |  | IVW (fixed effects) |  | 1.000 | 1.000-1.001 | 0.430 |  |  |
|  |  | MR Egger (bootstrap) |  | 1.000 | 1.000-1.001 | 0.385 |  |  |
|  |  | Simple median |  | 1.000 | 1.000-1.001 | 0.619 |  |  |
|  |  | Weighted median |  | 1.000 | 1.000-1.001 | 0.477 |  |  |
|  |  | Penalised weighted median |  | 1.000 | 1.000-1.001 | 0.463 |  |  |
|  | IL-17 | MR Egger |  | 1.001 | 0.999-1.002 | 0.398 | 5.543, 0.852 | 0.333 |
|  |  | IVW (random effects) |  | 1.000 | 0.999-1.001 | 0.990 | 6.580, 0.832 |  |
|  |  | IVW (fixed effects) |  | 1.000 | 0.999-1.001 | 0.992 |  |  |
|  |  | MR Egger (bootstrap) |  | 0.999 | 0.998-1.001 | 0.238 |  |  |
|  |  | Simple median |  | 1.000 | 0.999-1.001 | 0.749 |  |  |
|  |  | Weighted median |  | 1.000 | 0.999-1.001 | 0.479 |  |  |
|  |  | Penalised weighted median |  | 1.000 | 0.999-1.001 | 0.454 |  |  |
|  | IL-18 | MR Egger |  | 1.000 | 1.000-1.001 | 0.328 | 8.004, 0.889 | 0.648 |
|  |  | IVW (random effects) |  | 1.000 | 1.000-1.001 | 0.112 | 8.222, 0.915 |  |
|  |  | IVW (fixed effects) |  | 1.000 | 1.000-1.001 | 0.240 |  |  |
|  |  | MR Egger (bootstrap) |  | 1.001 | 1.000-1.001 | 0.064 |  |  |
|  |  | Simple median |  | 1.000 | 0.999-1.001 | 0.848 |  |  |
|  |  | Weighted median |  | 1.000 | 1.000-1.001 | 0.197 |  |  |
|  |  | Penalised weighted median |  | 1.000 | 1.000-1.001 | 0.182 |  |  |
|  | IL1ra | MR Egger |  | 1.001 | 0.999-1.003 | 0.392 | 2.486, 0.870 | 0.456 |
|  |  | IVW (random effects) |  | 1.000 | 1.000-1.001 | 0.451 | 3.119, 0.874 |  |
|  |  | IVW (fixed effects) |  | 1.000 | 0.999-1.001 | 0.614 |  |  |
|  |  | MR Egger (bootstrap) |  | 1.000 | 0.998-1.002 | 0.444 |  |  |
|  |  | Simple median |  | 1.000 | 0.999-1.001 | 0.815 |  |  |
|  |  | Weighted median |  | 1.000 | 0.999-1.001 | 0.746 |  |  |
|  |  | Penalised weighted median |  | 1.000 | 0.999-1.001 | 0.745 |  |  |
|  | IL2ra | MR Egger |  | 1.000 | 0.999-1.001 | 0.720 | 10.293, 0.113 | 0.410 |
|  |  | IVW (random effects) |  | 0.999 | 0.999-1.000 | 0.076 | 11.640, 0.113 |  |
|  |  | IVW (fixed effects) |  | 0.999 | 0.999-1.000 | 0.022 |  |  |
|  |  | MR Egger (bootstrap) |  | 1.000 | 0.999-1.001 | 0.335 |  |  |
|  |  | Simple median |  | 0.999 | 0.999-1.000 | 0.180 |  |  |
|  |  | Weighted median |  | 1.000 | 0.999-1.000 | 0.463 |  |  |
|  |  | Penalised weighted median |  | 1.000 | 0.999-1.000 | 0.482 |  |  |
|  | CRP | MR Egger |  | 1.000 | 0.999-1.000 | 0.220 | 298.711, 0.543 | 0.586 |
|  |  | IVW (random effects) |  | 1.000 | 0.999-1.000 | 0.214 | 299.008, 0.554 |  |
|  |  | IVW (fixed effects) |  | 1.000 | 0.999-1.000 | 0.217 |  |  |
|  |  | MR Egger (bootstrap) |  | 1.000 | 0.999-1.000 | 0.086 |  |  |
|  |  | Simple median |  | 1.000 | 0.999-1.000 | 0.497 |  |  |
|  |  | Weighted median |  | 0.999 | 0.999-1.000 | 0.084 |  |  |
|  |  | Penalised weighted median |  | 0.999 | 0.999-1.000 | 0.088 |  |  |
|  | CXCL9 | MR Egger | 14 | 1.000 | 0.999-1.001 | 0.819 | 9.636, 0.648 |  |
|  |  | IVW (random effects) | 14 | 1.000 | 0.999-1.000 | 0.423 | 9.650, 0.722 | 0.910 |
|  |  | IVW (fixed effects) | 14 | 1.000 | 0.999-1.000 | 0.490 |  |  |
|  |  | MR Egger (bootstrap) | 14 | 1.000 | 0.999-1.000 | 0.343 |  |  |
|  |  | Simple median | 14 | 1.000 | 0.999-1.000 | 0.330 |  |  |
|  |  | Weighted median | 14 | 1.000 | 0.999-1.001 | 0.754 |  |  |
|  |  | Penalised weighted median | 14 | 1.000 | 0.999-1.001 | 0.752 |  |  |
|  | Eotaxin | MR Egger |  | 1.001 | 0.999-1.002 | 0.408 | 31.465, 0.008 | 0.352 |
|  |  | IVW (random effects) |  | 1.000 | 0.999-1.001 | 0.933 | 33.399, 0.007 |  |
|  |  | IVW (fixed effects) |  | 1.000 | 0.999-1.001 | 0.904 |  |  |
|  |  | MR Egger (bootstrap) |  | 1.001 | 0.999-1.003 | 0.160 |  |  |
|  |  | Simple median |  | 1.000 | 0.999-1.001 | 0.837 |  |  |
|  |  | Weighted median |  | 1.000 | 0.999-1.001 | 0.719 |  |  |
|  |  | Penalised weighted median |  | 1.000 | 0.999-1.001 | 0.645 |  |  |
|  | GROa | MR Egger |  | 1.000 | 0.999-1.001 | 0.517 | 5.736, 0.571 | 0.604 |
|  |  | IVW (random effects) |  | 1.000 | 1.000-1.000 | 0.606 | 6.630, 0.644 |  |
|  |  | IVW (fixed effects) |  | 1.000 | 1.000-1.000 | 0.654 |  |  |
|  |  | MR Egger (bootstrap) |  | 1.000 | 1.000-1.001 | 0.087 |  |  |
|  |  | Simple median |  | 1.000 | 0.999-1.000 | 0.671 |  |  |
|  |  | Weighted median |  | 1.000 | 0.999-1.000 | 0.936 |  |  |
|  |  | Penalised weighted median |  | 1.000 | 0.999-1.001 | 0.940 |  |  |
|  | MCP1 | MR Egger |  | 1.001 | 0.999-1.002 | 0.400 | 25.723, 0.012 | 0.434 |
|  |  | IVW (random effects) |  | 1.000 | 0.999-1.001 | 0.735 | 27.129, 0.012 |  |
|  |  | IVW (fixed effects) |  | 1.000 | 1.000-1.001 | 0.625 |  |  |
|  |  | MR Egger (bootstrap) |  | 1.001 | 1.000-1.002 | 0.071 |  |  |
|  |  | Simple median |  | 1.000 | 0.999-1.001 | 0.721 |  |  |
|  |  | Weighted median |  | 1.001 | 1.000-1.002 | 0.110 |  |  |
|  |  | Penalised weighted median |  | 1.001 | 1.000-1.002 | 0.072 |  |  |
|  | MIF | MR Egger |  | 1.000 | 0.999-1.001 | 0.866 | 3.989, 0.551 | 0.587 |
|  |  | IVW (random effects) |  | 1.000 | 0.999-1.000 | 0.574 | 4.326, 0.633 |  |
|  |  | IVW (fixed effects) |  | 1.000 | 0.999-1.001 | 0.634 |  |  |
|  |  | MR Egger (bootstrap) |  | 0.999 | 0.997-1.001 | 0.133 |  |  |
|  |  | Simple median |  | 1.000 | 0.999-1.001 | 0.962 |  |  |
|  |  | Weighted median |  | 1.000 | 0.999-1.001 | 0.609 |  |  |
|  |  | Penalised weighted median |  | 1.000 | 0.999-1.001 | 0.596 |  |  |
|  | MIP1a | MR Egger |  | 1.001 | 0.999-1.002 | 0.443 | 3.956, 0.785 | 0.390 |
|  |  | IVW (random effects) |  | 1.000 | 0.999-1.000 | 0.875 | 4.794, 0.779 |  |
|  |  | IVW (fixed effects) |  | 1.000 | 0.999-1.001 | 0.903 |  |  |
|  |  | MR Egger (bootstrap) |  | 0.999 | 0.997-1.001 | 0.318 |  |  |
|  |  | Simple median |  | 1.000 | 0.999-1.001 | 0.879 |  |  |
|  |  | Weighted median |  | 1.000 | 0.999-1.001 | 0.871 |  |  |
|  |  | Penalised weighted median |  | 1.000 | 0.999-1.001 | 0.870 |  |  |
|  | MIP1b | MR Egger |  | 1.000 | 0.999-1.001 | 0.955 | 24.863, 0.129 | 0.865 |
|  |  | IVW (random effects) |  | 1.000 | 1.000-1.000 | 0.917 | 24.904, 0.164 |  |
|  |  | IVW (fixed effects) |  | 1.000 | 1.000-1.000 | 0.905 |  |  |
|  |  | MR Egger (bootstrap) |  | 1.000 | 1.000-1.001 | 0.261 |  |  |
|  |  | Simple median |  | 1.000 | 0.999-1.000 | 0.373 |  |  |
|  |  | Weighted median |  | 1.000 | 1.000-1.001 | 0.719 |  |  |
|  |  | Penalised weighted median |  | 1.000 | 1.000-1.001 | 0.708 |  |  |
|  | RANTES | MR Egger |  | 0.998 | 0.996-1.000 | 0.159 | 11.708, 0.165 | 0.079 |
|  |  | IVW (random effects) |  | 1.000 | 1.000-1.001 | 0.436 | 17.618, 0.040 |  |
|  |  | IVW (fixed effects) |  | 1.000 | 1.000-1.001 | 0.275 |  |  |
|  |  | MR Egger (bootstrap) |  | 1.001 | 1.000-1.004 | 0.012 |  |  |
|  |  | Simple median |  | 1.000 | 0.999-1.001 | 0.652 |  |  |
|  |  | Weighted median |  | 1.000 | 0.999-1.001 | 0.532 |  |  |
|  |  | Penalised weighted median |  | 1.000 | 0.999-1.001 | 0.512 |  |  |
|  | TNFa | MR Egger |  | 1.000 | 0.998-1.002 | 0.927 | 0.000, 0.977 | 0.511 |
|  |  | IVW (random effects) |  | 0.999 | 0.998-1.000 | 0.002 | 0.931, 0.628 |  |
|  |  | IVW (fixed effects) |  | 0.999 | 0.998-1.000 | 0.032 |  |  |
|  |  | MR Egger (bootstrap) |  | 0.998 | 0.984-1.012 | 0.268 |  |  |
|  |  | Simple median |  | 0.998 | 0.997-1.000 | 0.033 |  |  |
|  |  | Weighted median |  | 0.999 | 0.998-1.000 | 0.051 |  |  |
|  |  | Penalised weighted median |  | 0.999 | 0.998-1.000 | 0.046 |  |  |
|  | TNFb | MR Egger |  | 1.000 | 1.000-1.001 | 0.265 | 1.888, 0.389 | 0.315 |
|  |  | IVW (random effects) |  | 1.000 | 1.000-1.001 | 0.447 | 3.653, 0.301 |  |
|  |  | IVW (fixed effects) |  | 1.000 | 1.000-1.001 | 0.402 |  |  |
|  |  | MR Egger (bootstrap) |  | 1.000 | 0.999-1.002 | 0.315 |  |  |
|  |  | Simple median |  | 1.000 | 0.999-1.001 | 0.745 |  |  |
|  |  | Weighted median |  | 1.000 | 1.000-1.001 | 0.142 |  |  |
|  |  | Penalised weighted median |  | 1.000 | 1.000-1.001 | 0.146 |  |  |
|  | TRAIL | MR Egger |  | 1.000 | 0.999-1.000 | 0.290 | 21.717, 0.196 | 0.474 |
|  |  | IVW (random effects) |  | 1.000 | 0.999-1.000 | 0.409 | 22.401, 0.215 |  |
|  |  | IVW (fixed effects) |  | 1.000 | 1.000-1.000 | 0.357 |  |  |
|  |  | MR Egger (bootstrap) |  | 0.999 | 0.998-1.000 | 0.133 |  |  |
|  |  | Simple median |  | 1.000 | 1.000-1.001 | 0.687 |  |  |
|  |  | Weighted median |  | 1.000 | 0.999-1.000 | 0.175 |  |  |
|  |  | Penalised weighted median |  | 1.000 | 0.999-1.000 | 0.176 |  |  |

SNP, single-nucleotide polymorphisms; OR, odds ratio; CI, confidence internal; IVW, inverse variance weighting.
